# Replacement of Dietary Fishmeal with *Clostridium autoethanogenum* Protein on Lipidomics and Lipid Metabolism in Muscle of Pearl Gentian Grouper

**DOI:** 10.1155/2023/6723677

**Published:** 2023-06-30

**Authors:** Jia Xu, Bocheng Huang, Shuyan Chi, Shuang Zhang, Junming Cao, Beiping Tan, Shiwei Xie

**Affiliations:** ^1^Laboratory of Aquatic Animal Nutrition and Feed, Fisheries College, Guangdong Ocean University, Zhanjiang, China; ^2^Guangxi Key Laboratory of Marine Environmental Science, Beibu Gulf Marine Research Center, Guangxi Academy of Sciences, Nanning, China; ^3^Aquatic Animals Precision Nutrition and High Efficiency Feed Engineering Research Center of Guangdong Province, Zhanjiang, China; ^4^Guangdong Provincial Key Lab of Pathogenic Biology and Epidemiology for Aquatic Economic Animals, Zhanjiang 524088, China

## Abstract

*Clostridium autoethanogenum* protein (CAP) is an economical and alternative protein source. Here, three experimental diets were formulated with CAP replacing 0% (CAP-0), 30% (CAP-30), and 60% (CAP-60) of fishmeal to investigate the alterations of structure integrity, fatty acids profiles, and lipid metabolism in the muscle of pearl gentian grouper. With increasing levels of CAP substitution, the percentages of 16 : 0 or 18 : 0 were decreased in triglycerides (TG) and diacylglycerols (DG); 18 : 1 or 18 : 2 was increased at the *sn*−*1* and *sn*−*2* positions in phosphatidylethanolamines; 20 : 5*n*−3 was increased in TG and DG. The phosphatidylcholines (PC) (18 : 3/20 : 5), PC(22 : 6/17 : 1), and sphingomyelins (d19 : 0/24 : 4) were identified as potential lipid biomarkers between CAP treatments. The CAP-30 treatment enhanced lipolysis and lipogenesis, while the CAP-60 treatment inhibited lipogenesis. In conclusion, fishmeal replacement with CAP affected the lipid characteristics and lipid metabolism, whereas it did not affect the structural integrity and fatty acids profiles in the muscle of pearl gentian grouper.

## 1. Introduction

The pearl gentian grouper (*Epinephelus lanceolatus*♂×*Epinephelus fuscoguttatus*♀) is a hybrid seawater fish created by the Golden Seed Project to cultivate high-quality species with fast growth, strong disease resistance, and delicious meat [1, 2]. As a carnivorous fish, the protein level in the feed of pearl gentian grouper is typically as high as 50% [3, 4]. Fishmeal, which contains balanced essential amino acids and high growth-promoting factors and exhibits high digestibility, has been used as a major source of protein for marine fish feed [5, 6]. However, with an increase in demand and the shortage of production, the price of fishmeal is constantly rising, which greatly affects the feeding cost [7]. Therefore, many enterprises and research scholars urgently need to look for cheaper and more sustainable alternative protein sources to replace fishmeal [8, 9] so as to promote the healthy development of grouper aquaculture.

*Clostridium autoethanogenum* is a nonpathogenic and Gram-positive bacterium. It can effectively utilize carbon monoxide in steelmaking waste gas as carbon source and ammonia as nitrogen source to biosynthesize bacterial protein, *C. autoethanogenum* protein (CAP) [6, 10]. Recently, CAP was listed in the list of feed additives in China (Announcement no. 465 of the Ministry of Agriculture of China). The content of protein in CAP is high, accounting for about 85% of dry matter weight, and it contains a lot of essential amino acids, vitamins, and other nutrients, and the amino acid composition is similar to that of fishmeal [11]. Studies in aquatic animals have shown that the replacement of fishmeal by CAP could improve the growth performance, inflammatory response, intestinal health, intestinal microflora, and flesh quality of largemouth bass (*Micropterus salmoides*) [6, 11, 12]; changed the growth performance, intestinal structure, and meat quality of Pacific white shrimp (*Litopenaeus vannamei*) [13–15]; altered the growth performance and antioxidant properties of black sea bream (*Acanthopagrus schlegelii*) [16]. However, up to now, there are few research reports on CAP in pearl gentian grouper.

Lipidomics is a scientific method based on mass spectrometry, which can identify and quantify the types, composition, and structure of thousands of cellular lipid molecules in organisms so as to accurately discover and clarify the changes in lipid types and lipid metabolism [17, 18]. At the same time, the fatty acid profile is the final reflection of the metabolic processes of all substances and fatty acids occurring in tissues, such as muscle, liver, and intestine [5, 19]. However, to date, few people have studied the characteristics of lipidomic and fatty acid composition in the muscle of grouper. Here, we hypothesized that replacing fishmeal with CAP would affect the structural integrity, fatty acids composition, lipidomics, and lipid metabolism in the muscle of pearl gentian grouper. Therefore, CAP was used to replace different proportions (0%, 30%, and 60%) of fishmeal to feed juvenile pearl gentian grouper, and then the effects of dietary CAP on muscle were evaluated from the aspects of structural changes, fatty acids compositions, lipid molecular alterations, potential biomarkers identification, lipid, and fatty acids metabolism. This study will provide new information and direction for evaluating the nutritional values of CAP inclusions in aquatic animals.

## 2. Materials and Methods

### 2.1. Chemicals

The antibody against PPAR*α* (peroxisome proliferator-activated receptor alpha, 66836-1-Ig) was purchased from Proteintech Co. (Chicago, USA). The antibodies against SREBP1 (sterol regulator element-binding protein 1, ab28481) and P-PPAR*α* (phosphor-PPAR*α*, S12, ab3484) were purchased from Abcam Co. (Cambridge, UK). The antibody against GAPDH (2118S) was purchased from Cell Signaling Technology Co. (MA, USA). The animal treatment and experimental procedures are carried out according to the “Guidelines for the Care and Use of Experimental Animals” issued by the National Research Council. All animal care and use procedures have been approved by the Animal Ethics and Welfare Committee of Guangdong Ocean University (license number: DOU-AEWC-20180063), and all authors have clearly stated that they have followed these guidelines.

### 2.2. Experimental Diets and Fish

The basic diet contained 50% fishmeal, and add CAP to the basic diet instead of 0%, 30%, and 60% of fishmeal to form CAP-0, CAP-30, and CAP-60 diets, respectively (all diets were isoenergetic and isonitrogenous). At the same time, methionine and arginine were added to the feeds of CAP-30 and CAP-60 to reach the same level as that of CAP-0 so as to meet the needs of pearl gentian grouper [3]. For the preparation and manufacturing of feed (*Supplementary 1*), please refer to our previous experiment [20].

Juvenile pearl gentian grouper (*E. fuscoguttatus*♀×*E. lanceolatus*♂, *n* = 300, bodyweight of 18.01 ± 0.82 g) were distributed into 12 plastic barrels (300 L) reinforced with glass fiber, with 25 fish per barrel, ensuring four replicates in CAP-0, CAP-30, and CAP-60 treatments, respectively. The acclimation, feeding, and rearing conditions of fish are described in our previous study [20, 21]. One day (24 hr) before the end of the 8-week feeding experiment, all the fish were anesthetized with MS-222 (Sigma Aldrich, USA). Five fish per barrel (each group of 20) were randomly selected to obtain the white muscle under the last bundle of the dorsal fin. Then, three muscle samples were exposed to 2.5% (v/v) glutaraldehyde in phosphate buffer solution for microstructure analysis. The remaining muscle samples were frozen in liquid nitrogen and stored at −80°C, which were used for the analysis of lipidomics, fatty acid, gene expression, and protein expression analyses.

### 2.3. Structural Change in Muscle Analyses

The scanning electron microscopy (SEM) analysis was used to compare and evaluate the possible microstructure changes. SEM analysis refers to the previous experiments [22, 23], including prefixation, fixation with osmium tetroxide, ethanol dehydration, drying dehydration, and finally, treatment with a gold-coating machine (HITACHI MC1000, Hitachi, Tokyo, Japan), and observation with an SEM (HITACHI Regulus 8100, Hitachi, Tokyo, Japan).

### 2.4. Fatty Acids in Muscle Analyses

The quantification of fatty acids in muscle was determined in accordance with the previous methods [18]. The standards of fatty acids methyl ester are shown in *Supplementary 2* and *3*. The precision and stability of each fatty acid are shown in *Supplementary 4*. The sample was ground and the supernatant was collected; then, hexane was added after esterification, anhydrous sodium sulfate was added to the supernatant, and the supernatant was centrifuged again and hexane was added. After methyl salicylate was added, part of the supernatant was determined by gas chromatography–mass spectrometry. Processed samples were determined on a Trance 1310 (Thermo Scientific, Delaware, USA) equipped with a Thermo TG-FAME capillary column (50 m × 0.25 mm, ID × 0.20 *μ*m). The mass spectrometer ISQ 7000 (Thermo Scientific, Delaware, USA) was used to perform this operation in full scanning mode (mass range *m*/*z* 40–500). The concentrations of each fatty acid were calculated according to the peak area of fatty acid to the peak area of internal standard substance.

### 2.5. Lipidomics Analyses

Quantification of lipid molecules in muscle was performed according to the previous methods [24, 25]. Add an appropriate amount of samples to chloroform–methanol mixture, vortex on ice, add water and then take the lower solution and add chloroform–methanol mixture again. The lower solution was concentrated in vacuum and dissolved in isopropanol, and then detected by lipid chromatography (LC)–MS. Chromatographic separations were performed on a Thermo Ultimate 1290 system equipped with a Phenomenex Kinetex C18 column (100 × 2.1 mm, 1.7 *μ*m). After analysis by the parameters of the mass spectrometry (AB 6600, AB SCIEX), the lipid molecules were identified and quantified using Thermo Scientific™ and LipidSearch™ 4.1 SP2 software. The raw lipidomics data (accession number: MTBLS4807) were deposited in the MetaboLights database [26].

### 2.6. The qPCR Analyses

The details of total RNA extraction using 1 ml of Trizol (TRI Reagent solution, Invitrogen, Carlsbad, CA, USA), cDNA preparation using an Evo M-MLV reagent Kit with gDNA Eraser (Accurate Biotechnology (Hunan) Co., Ltd), and qPCR assays using SYBR® Green Pro Taq HS (Accurate Biotechnology (Hunan) Co., Ltd) are provided in our previous study [2, 27]. Primers used in this study included in *Supplementary 5*: fatty acid synthase (*fas*), *srebp1*, *pparγ*, adipose triglyceride lipase (*atgl*), *pparα*, acyl-CoA oxidase 1 (*aco*), delta-6 fatty acyl desaturase (*fad6*), elongase of very long-chain fatty acid 4 (*elovl4*), *elovl8*, fatty acid binding protein (*fabp*), uncoupling protein 2 (*ucp2*), and liver X receptor alpha (*lxr*). *β-actin* and *18s rRNA* were used as reference genes to normalize the genes expression. The results of gene expression were calculated using the 2^−*ΔΔ*CT^ method [28, 29].

### 2.7. Western Blot Analyses

The total protein extraction, quantification, SDS-PAGE gel electrophoresis, transferring, blocking, incubation, and visualization assay were based on our published methods [4, 30]. Western bands were quantified using Image J (version 1.42, National Institutes of Health). The primary antibodies used in this study included SREBP1 (1 : 800), PPAR*α* (1 : 1,000), P-PPAR*α* (1 : 800), and GAPDH (1 : 1,000).

### 2.8. Statistical Analysis

Shapiro–Wilk test and Levene test were used for the normality and homogeneity test of results, respectively [31]. One-way analysis of variance was used for evaluation, and Duncan's multiple range test was used for significance analysis. SPSS 23.0 (IBM, Armonk, NY, USA) was used for the previous analysis. The final results were expressed as mean ± standard deviation (SD), where the threshold of statistical significance was <0.05.

## 3. Results and Discussion

### 3.1. The Structural Change in Muscle

**Figure 1:**
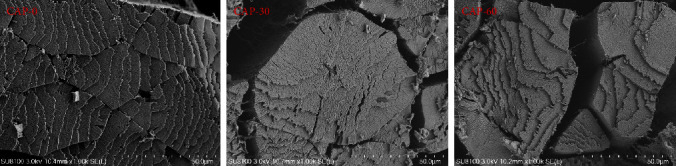
Scanning electron microscopy microstructures in the muscle of pearl gentian grouper. CAP-0, CAP replacing 0% of fishmeal; CAP-30, CAP replacing 30% of fishmeal; CAP-60, CAP replacing 60% of fishmeal.

The SEM sections of muscle tissue of the pearl gentian grouper were obtained and compared. As shown in Figure 1, there were no significant differences in the myofibrils, bundles, space, and structural connectivity between the CAP-0, CAP-30, and CAP-60 groups. These groups showed tight and clear myofibrils and muscle bundles, with smaller gaps between bundles and well-organized structure. Compared to CAP-0 treatment, CAP-30 treatment has no adverse effects on the flesh quality of Pacific white shrimp, including texture characteristics, shear force, and water-holding capacity [14], while CAP-14 treatment did not affect the texture characteristics and water-holding capacity of the muscle of largemouth bass [11]. These observations are consistent with our results to some extent; that is, CAP inclusions (0%–60%) did not change the integrity of the muscle structure of pearl gentian grouper.

### 3.2. The Composition of Lipid in Muscle

In this experiment, the method based on untargeted lipidomics was used to identify and identify the characteristics of total lipid composition and distribution in the muscles of pearl gentian grouper with different levels of CAP. As shown in *Supplementary 6*, the pooled quality control samples were closely clustered and separated from the treatment groups (CAP-0, CAP-30, and CAP-60 samples), which demonstrating the high stability of the system and reliability of the data. The OPLS-DA scores in positive and negative ion modes were *R*^2^*X* = 0.767, *R*^2^*Y* = 0.993, and *Q*^2^ = 0.874, while the validation plots provided intercept parameters of *R*^2^ (0.0, 0.54) and *Q*^2^ (0.0, −0.48) (*Supplementary 6*). The results of OPLS-DA showed that the lipid composition and structure of the three treatment groups were obviously separated. At the same time, CAP-30 and CAP-60 groups showed small intragroup variations, while CAP-0 group showed large intragroup variations. Based on these findings, we concluded that the addition of CAP resulted in a dramatic change in lipid profiles in the muscle of pearl gentian grouper.

**Figure 2:**
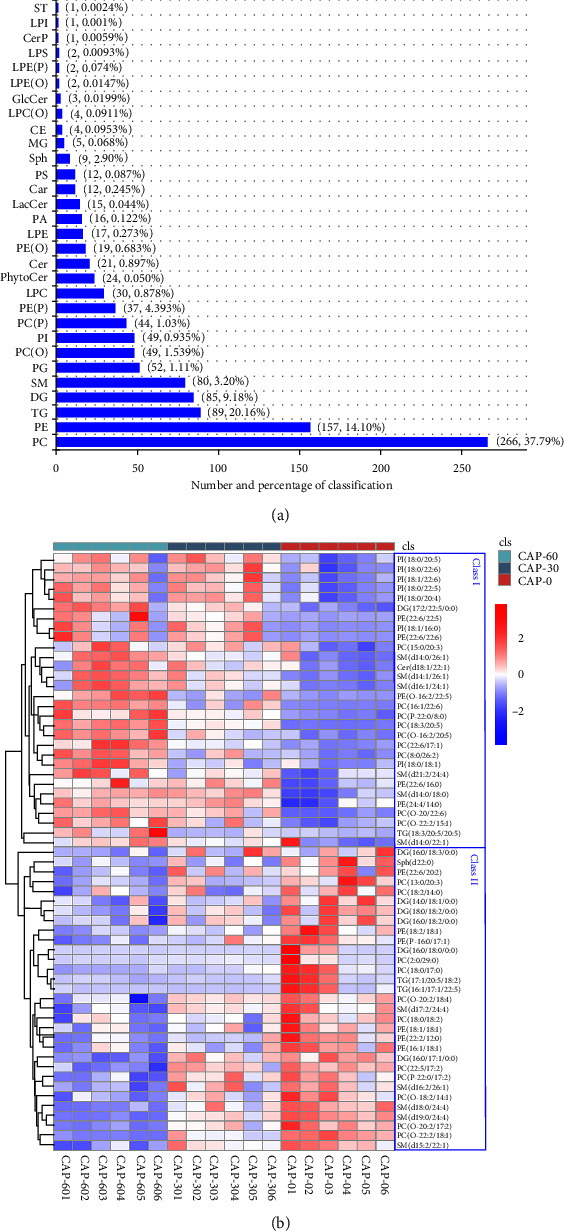
The lipid composition and dierential lipid molecules in the muscle of pearl gentian grouper. (a) The numbers and percentages of lipid metabolites. (b) The heatmap of dierential lipid molecules. CAP-0, CAP replacing 0% of fishmeal; CAP-30, CAP replacing 30% of fishmeal; CAP-60, CAP replacing 60% of fishmeal; PC, phosphatidylcholines; PE, phosphatidylethanolamines; TG, triglycerides; DG, diacylglycerols; SM, sphingomyelins; CE, cholesterol ester; Cer, ceramides; LPC, lysophosphatidylcholine; LPE, lysophosphatidylethanolamine; LPG, lysophosphatidylglycerol; PC(O), alkylphosphatidylcholine; PC(P), alkenylphosphatidylcholine; PE, phosphatidylethanolamines; PE(P), alkenylphosphatidylethanolamine; PG, phosphatidylglycerol; PhytoCer, phytoceramides; PI, phosphatidylinositol.

A total of 1,108 lipid molecules of 30 classes were identified in the muscle of pearl gentian grouper across different groups. It was mainly composed of phosphatidylcholines (PC, 37.79%), triglycerides (TG, 20.16%), phosphatidylethanolamines (PE, 14.10%), diacylglycerols (DG, 9.18%), and sphingomyelins (SM, 3.20%) (*Supplementary 7* and Figure 2(a)). Our results were consistent with the findings from Nile tilapia (*Oreochromis niloticus*) that lipid class mainly included TG, DG, PC, PE, phosphatidylserine (PS), and phosphatidylinositol (PI) [19]. PC and PE belong to a kind of widely distributed polar lipids, mainly as one of the components of cell membrane, and play a key role in maintaining the basic structure of cell membrane, bidirectional fluidity, and regulation of internal and external signal transduction [19]. The PC and PE were abundantly detected in caramote prawn (*Penaeus kerathurus*), mantis shrimp (*Squilla mantis*), and Pacific white shrimp [32, 33], while the SM was a component of phosphosphingolipid and widely present in the muscle of Pacific white shrimp [33]. TG is a neutral lipid that plays a key role as the central molecule in cell biology, organ function, and lipid metabolism [18]. Unlike our study, TG was the predominant fraction in muscle, accounting for more than 83% in Nile tilapia and 77.5% in swimming crabs (*Portunus trituberculatus*) [18, 19, 34]. In any case, it should be noted that there is considerable variation in the composition and abundance of lipids in different species and/or their tissues [33]. These observations and our findings indicated that the major lipid classes were similar in aquatic animals.

Compared to CAP-0 group, the CAP-30 treatment significantly increased the levels of PI, PS, and sulfatide (ST); the CAP-60 treatment significantly decreased the levels of phytoceramides (PhytoCer) and monoglyceride (MG) while significantly increased the levels of PS, lysophosphatidylserine (LPS), and ST (*Supplementary 6*). Consistent with the present study, the level of TG was not affected in CAP treatments (0%–58.2%) in serum of black sea bream [16]. However, with increasing levels of CAP substitution (0%–75% or 0%–93%), the content of TG was significantly increased in serum of largemouth bass or Jian carp (*Cyprinus carpio* var. Jian), respectively [6, 35]. Due to the lack of researches on the correlation between CAP and lipid composition in the muscle of animals, more studies are needed to confirm and explain our results. Overall, the levels of PS and ST classes in pearl gentian muscle increased significantly with increasing levels of CAP substitution (0%–60%).

### 3.3. The Alteration of Lipid Molecules and Biomarkers in Muscle

In the present study, the lipid species with *p* < 0.05 and VIP > 1 were defined as dierential lipid molecules (DLM). There were 61 DLM among CAP-0, CAP-30, and CAP-60 groups, comprising by 11 PC, 11 SM, 9 PE, 7 PI, 7 DG, 7 alkylphosphatidylcholine (PC(O)), 3 TG, 2 alkenylphosphatidylcholine PC(P), 1 Cer, 1 PE(O), 1 PE(P): alkenylphosphatidylethanolamine, and 1 Sph (*Supplementary 8*). Curiously, these DLM were largely inconsistent with the differential lipids classes that we found earlier (such as PS and ST). The heatmap was used to provide an intuitive visualization of the relative levels of lipids molecules, which showing their abundance in comparison groups. As shown in Figure 2(b), the level of lipid class I was low in CAP-0, intermediate in CAP-30, and high in CAP-60, consisting mainly of PI and PC classes. Meanwhile, the level of lipid class II was low in CAP-60, intermediate in CAP-30, and high in CAP-0, consisting mainly of DG and TG classes. In addition, the CAP-70 treatment significantly reduced the level of PC(15 : 0/16 : 0) in Pacific white shrimp [14]. However, the CAP-30 and CAP-60 treatment did not affect the level of PC(15 : 0/16 : 0) in this study. Overall, the replacement of fishmeal with CAP directly altered the abundance of some specific lipid molecules in the muscle of pearl gentian grouper, mainly concentrating on PI, PC, DG, and TG classes.

**Figure 3:**
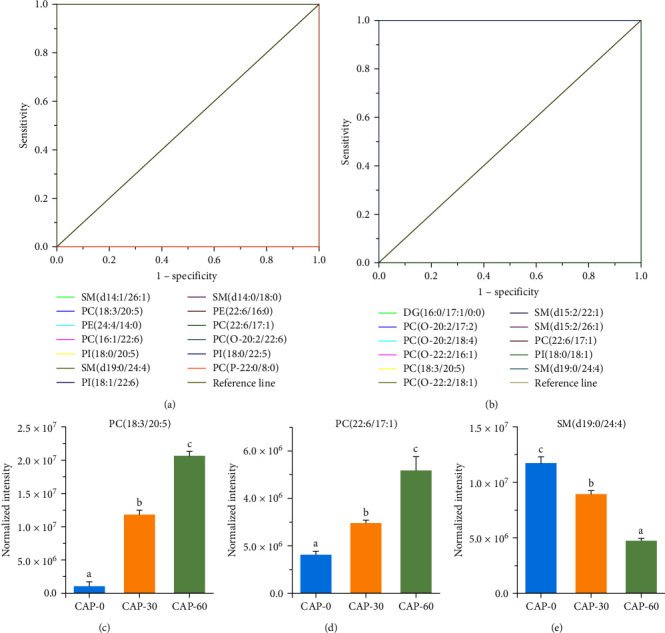
Receiver operating characteristic curve and normalized intensity for potential lipid biomarkers in the muscle of pearl gentian grouper. (a) The receiver operating characteristic curve between CAP-0 and CAP-30 groups. (b) The receiver operating characteristic curve between CAP-30 and CAP-60 groups. (c–e) The normalized intensity of PC(18 : 3/20 : 5), PC(22 : 6/17 : 1), and SM(d19 : 0/24 : 4). Values are presented as means with SD, where significant (*p* < 0.05) differences between groups are indicated by different letters.

Biomarker is a defined characteristic that is measured as an indicator of normal biological processes, pathogenic processes, or responses to an exposure or intervention [36, 37]; we used this method to discriminate the target samples from different treatments. The receiver operating characteristic (ROC) curves of DLM were used to identify the potential biomarkers between different treatment groups [33], with an area under the ROC curve (AUC) of 1 or 0. The area under AUC, specificity, and sensitivity values of target DLM were also calculated and presented in Figure 3. In the CAP-0 and CAP-30 groups, the 13 lipid molecules were identified as potential lipid biomarkers, including SM(d14 : 1/26 : 1), PC(18 : 3/20 : 5), PE(24 : 4/14 : 0), PC(16 : 1/22 : 6), PI(18 : 0/20 : 5), SM(d19 : 0/24 : 4), PI(18 : 1/22 : 6), SM(d14 : 0/18 : 0), PE(22 : 6/16 : 0), PC(22 : 6/17 : 1), PC(O-20 : 2/22 : 6), PI(18 : 0/22 : 5), and PC(P-22 : 0/8 : 0) (Figure 3(a)). In the CAP-30 and CAP-60 groups, the 12 potential lipid biomarkers included DG(16 : 0/17 : 1/0 : 0), PC(O-20 : 2/17 : 2), PC(O-20 : 2/18 : 4), PC(O-22 : 2/16 : 1), PC(18 : 3/20 : 5), PC(O-22 : 2/18 : 1), SM(d15 : 2/22 : 1), SM(d15 : 2/26 : 1), PC(22 : 6/17 : 1), PI(18 : 0/18 : 1), and SM(d19 : 0/24 : 4) (Figure 3(b)). Thus, 3 lipid molecules, PC(18 : 3/20 : 5), PC(22 : 6/17 : 1), and SM(d19 : 0/24 : 4), were identified as potential lipid biomarkers among the CAP-0, CAP-30 and CAP-60 groups. In details, the levels of PC(18 : 3/20 : 5) and PC(22 : 6/17 : 1) were significantly increased in CAP-30 group (compared to CAP-0 group) and CAP-60 group (compared to CAP-30 group), whereas the level of SM(d19 : 0/24 : 4) was significantly decreased in CAP-30 group and CAP-60 group (Figure 3(c)–3(e)). Clearly, replacing of fishmeal by CAP-induced significant alterations in these three biomarkers, which in turn may affect the composition of lipids.

### 3.4. The Distribution of Fatty Acids in Muscle

The positional distribution of fatty acids in the side chains of lipid molecules greatly aects its nutritional values and role in energy metabolism [38]. To further investigate the distributions of seven key fatty acids (such as 16 : 0, 18 : 0, 18 : 1*n*−9, 18 : 2*n*−6, 20 : 5*n*−3, 22 : 5*n*−3, and 22 : 6*n*−3), the percentages of fatty acids in PC, PE, TG, and DG (the four most abundant classes) were calculated and compared.

**Figure 4:**
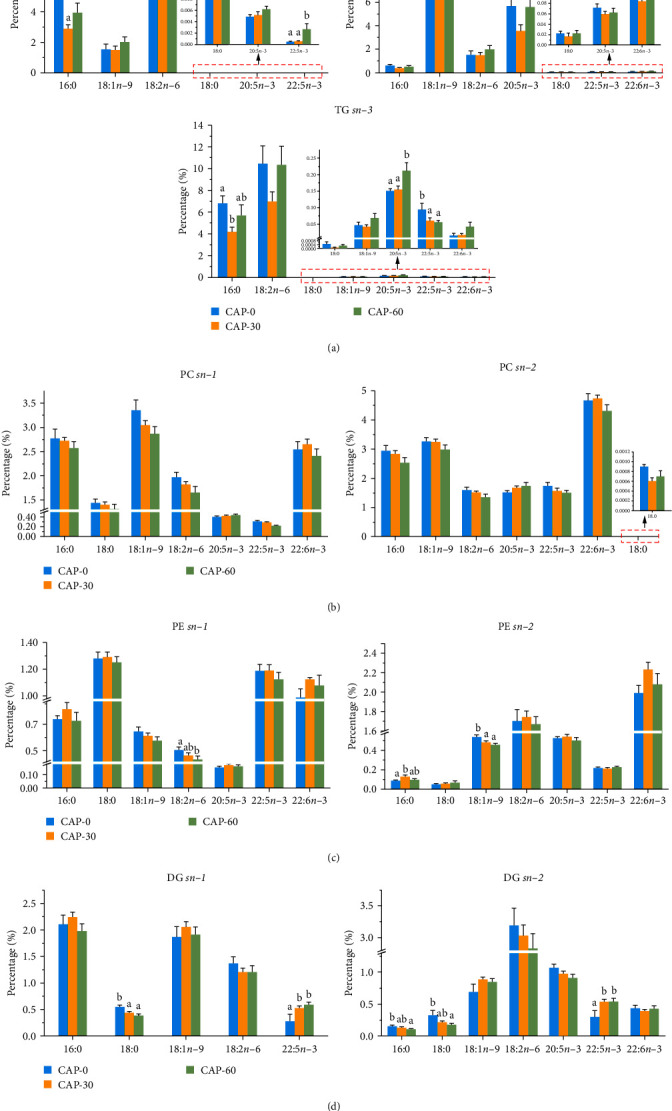
Positional distributions (*sn*−*1*, *sn*−*2*, and *sn*−*3*) of key fatty acids in TG (a), PC (b), PE (c), and DG (d) in the muscle of pearl gentian grouper. CAP-0, CAP replacing 0% of fishmeal; CAP-30, CAP replacing 30% of fishmeal; CAP-60, CAP replacing 60% of fishmeal. Values are presented as means with SD, where significant (*p* < 0.05) differences between groups are indicated by different letters.

In general, in most TG molecules, fatty acids are esterified to three stereoscopic positions on the glycerol skeleton such that their steric number (sn) is *sn*−*1*, *sn*−*2*, and *sn*−*3*, respectively [19]. For the TG classes, the saturated fatty acids (SFA) predominantly occupied the *sn*−*2* positions, while the monounsaturated fatty acids (MUFA) and polyunsaturated fatty acids (PUFA) predominantly occupied the *sn*−*1* or *sn*−*3* positions in muscle, head, and viscera of tilapia [34]. In contrast, the SFA predominantly occupied the *sn*−*1* and *sn*−*3* position, whereas the MUFA tended to be located at *sn*−*1* and *sn*−*2* positions in the present study (Figure 4(a)). The previous study found that SFA at the *sn*−*1* or *sn*−*3* position of TG is preferentially decomposed by lipase, which further confirmed that the positional distribution of fatty acids could affect their role in energy metabolism [39]. Compared to CAP-0, the percentages of 16 : 0 at *sn*−*1* and *sn*−*3* in TG were significantly decreased in CAP-30, suggesting that CAP inclusions (30%) increased the catabolism of SFA and decreased their deposition in pearl gentian grouper (Figure 4(a)). In addition, an increased level of 18 : 2*n*−6 at the *sn*−*1* or *sn*−*3* positions of TG might enhance the risk of inflammation [19, 39]. In the present study, the CAP treatments were unaffected by the percentages of 18 : 2*n*−6 at the *sn*−*1* and *sn*−*3* positions, suggesting that the addition of CAP did not affect the inflammatory status of pearl gentian grouper. In contrast to the finding that the 20 : 5*n*−3 tended to distribute at *sn*−*1* and *sn*−*3* positions in TG of Nile tilapia [19], we found the 20 : 5*n*−3 was predominantly distributed at *sn*−*2* position in the present study. In addition, 22 : 6*n*−3 at the *sn*−*2* position in TG was more stabilize than at the *sn*−*1* and *sn*−*3* positions in the muscle of Atlantic salmon (*Salmo salar*) [40]. In partial agreement with this, we found that 22 : 6*n*−3 was only located at the *sn*−*2* and *sn*−*3* position in pearl gentian grouper (Figure 4(a)). In the present study, the percentage of 22 : 5*n*−3 in TG increased significantly at the *sn*−*1* position and decreased significantly at the *sn*−*3* position as CAP levels increased. As there is no information available on the impacts of dietary CAP on the distribution of key fatty acids in specific lipid class in the muscle of fish, more research is needed to interpret our results.

Although the number, length, and double bond positions of side-chain fatty acids of PC and PE are quite different, there is usually an SFA at the *sn*−*1* position [41]. In the present study (Figure 4(b)), the percentages of 16 : 0 and 18 : 0 in PC, PE, and DG classes were much higher at the *sn*−*1* or *sn*−*3* positions than at the *sn*−*2* position in pearl gentian grouper, which is similar to the finding in Nile tilapia [39]. Consistent with our results, the 20 : 5*n*−3 and 22 : 6*n*−3 were inclined to deposit at the *sn*−*2* position in PC and PE classes in swimming crab and Nile tilapia [18, 19]. In mammals, 22 : 6*n*−3 is most preferentially binds to the PE skeleton, followed by the PC skeleton [38]. This may be due to the fact that 22 : 6*n*−3 in PE has a more important physiological function [33]. Thus, in pearl gentian grouper, the PE was the major lipid species for 22 : 6*n*−3 deposition, compared to TG, PC, and DG classes (Figure 4(b)–4(d)).

To sum up, replacing of fishmeal by CAP (0%–60%) influenced the positional distributions of fatty acids in muscle of pearl gentian grouper (Figure 4(a)–4(d)). With increasing levels of dietary CAP: (1) the percentages of 16 : 0 or 18 : 0 were decreased in TG and DG, while increased in PE; (2) more 18 : 1 or 18 : 2 was accumulated at the *sn*−*1* and *sn*−*2* positions in PE; (3) the percentage of 20 : 5*n*−3 was increased in TG and DG; (4) 22 : 5*n*−3 was accumulated at *sn*−*1* in PC and at *sn*−*3* in TG, while reduced at *sn*−*1* in TG.

### 3.5. The Fatty Acids Profiles in Muscle

In general terms, SFA are used as an available energy sources, while PUFA are used in the synthesis of eicosanoids in kinds of fish tissues [34]. In the present study, concentrations of SFA across the CAP-0, CAP-30 and CAP-60 groups were 850.88 *μ*g/g (on average), dominated by 16 : 0 (482.67 *μ*g/g) and 18 : 0 (310.51 *μ*g/g) (Table 1). The concentrations of n-3 PUFA were 386.71 *μ*g/g, predominating by 20 : 5*n*−3 (128.04 *μ*g/g) and 22 : 6*n*−3 (184.81 *μ*g/g). The concentrations of *n*−6 PUFA were 202.15 *μ*g/g, predominating by 18 : 2*n*−6 (169.20 *μ*g/g). MUFA, especially 18 : 1*n*−9, is a “healthy” fatty acid for human body, which can remove bad cholesterol and protect cardiovascular and cerebrovascular health, etc. [42]. The concentrations of MUFA were 361.46 *μ*g/g, dominated by 18 : 1*n*−9c (146.34 *μ*g/g). Compared to the CAP-0 group, the concentrations of C18 : 1*n*−9T was significantly decreased in the CAP-30 and CAP-60 groups; the concentration of C22 : 1*n*−9T was significantly decreased in the CAP-60 group. These findings suggested that the muscle of pearl gentian grouper maybe a good source of 18 : 1 *n*−9 for human consumption, while dietary CAP inclusions weaken this benefit.

Basing on the AUC (1 or 0), none of fatty acid was identified as potential biomarkers between CAP-0 and CAP-30 groups or CAP-30 and CAP-60 groups (*Supplementary 9* and *10*). The PCA, PLS-DA and OPLS-DA score plots, as well as the hierarchical cluster, all showed no significant separation or clustering between the CAP-0, CAP-30 and CAP-60 groups (*Supplementary 6*), suggesting that fatty acids compositions were not affected by the CAP substitution. Similar to our study, the composition of all fatty acids did not differ from the control diet at low CAP levels (0%–30%), and the composition of SFA and MUFA did not differ at all dietary CAP levels (0%–100%) in Pacific white shrimp [14]. However, the levels of *n*−3 PUFA was significantly increased, while the level of *n*−6 PUFA was significantly decreased at high CAP levels (more than 70%) [14]. Overall, in this study, dietary fishmeal can be replaced by CAP up to 60% without adversely affecting the fatty acids composition in the muscle of pearl gentian grouper.

### 3.6. The Lipid Metabolism in Muscle

**Figure 5:**
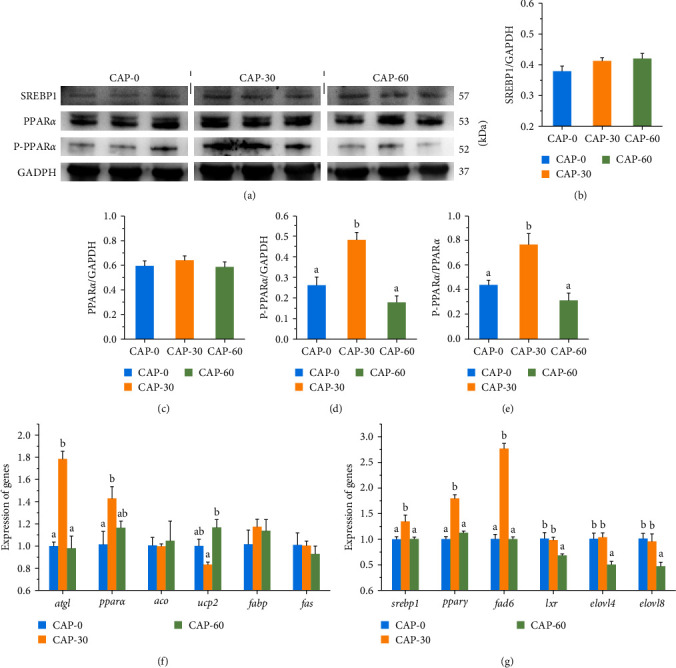
The expression of proteins and genes associated with lipid metabolism in muscle of pearl gentian grouper. (a) Western blot analysis of SREBP1, PPARA, P-PPARA, and GAPDH. (b–d) The relative quantification of SREBP1, PPARA, and P-PPARA proteins normalized to the GAPDH. (e) The relative quantification of P-PPARA protein normalized to the PPARA. (f and g) Gene expression of lipid metabolism. PPARA, peroxisome proliferator-activated receptor alpha; SREBP1, sterol-regulator element-binding protein 1; *fas*, fatty acid synthase; *atgl*, adipose triglyceride lipase; *aco*, acyl-CoA oxidase 1; *fad6*, delta-6 fatty acyl desaturase; *elovl4*, elongase of very long-chain fatty acid 4; *fabp*, fatty acid binding protein; *ucp2*, uncoupling protein 2; *lxr*, liver X receptor alpha; CAP-0, CAP replacing 0% of fishmeal; CAP-30, CAP replacing 30% of fishmeal; CAP-60, CAP replacing 60% of fishmeal. Values are presented as means with SD, where significant (*p* < 0.05) differences between groups are indicated by different letters.

The enrichment analysis of metabolites in CAP-30 and CAP-70 groups showed that amino acid metabolism and lipid metabolism pathways were greatly affected between CAP-treated group and control group in the muscles of Pacific white shrimp and largemouth bass [11, 14]. Therefore, we further evaluated the alterations of lipid metabolism and measured the expression of related proteins and genes in the muscle of pearl gentian grouper. The CAP treatment (0%–50%) significantly decreased the mRNA levels of *pparα* in largemouth bass [12]. In contrast, in the present study, the CAP-30 treatment significantly increased the expression of PPAR*α* protein and gene and the ratio of p-PPAR*α* to PPAR*α* protein (Figure 5(a)–5(e)). In addition, the mRNA level of *atgl* was downregulated in the CAP-25 or CAP-37.5 groups but upregulated in the CAP50 group in largemouth bass [12]. Similarly, in this study, CAP-30 treatment significantly increased the expression of *atgl* (Figure 5(f)). The SREBP1 is a transcriptional regulator that binds to cell membranes and regulates a series of genes required for TG synthesis, which in turn promotes the biosynthesis of sterols and fatty acids [43]. Our results showed that the expression of *srebp1* and *pparr* genes was significantly increased in CAP-30 treatment (Figure 5(f)–5(g)).

It was reported that the biosynthesis of LC-PUFA from 18 : 2*n*−6 to 18 : 3*n*−3 is activated by *fad6* [44]. Meanwhile, ELOVL4 enzyme plays an important role in the biosynthesis of LC-PUFA by extending EPA and DPA to 24 : 5*n*−3, which is a key intermediate in the production of DHA [44]. In addition, *elovl8*, an elongation enzyme discovered in recent years, has been shown to have elongation effects on C18 and C20 PUFA in teleost fish [45]. In the present study, the expression level of *fad6* was significantly increased in the CAP-30 group, whereas *elovl8* and *elovl4* were significantly decreased in the CAP-60 group. Our results indicated that CAP-30 treatment enhanced the activity of fatty acids desaturation, while the CAP-60 treatment inhibited the activity of fatty acids elongation. Although the expression of genes related to fatty acids metabolism was altered by the addition of CAP, the fatty acids composition was not affected in this study. Further researches are needed to explain this incongruity. Overall, a low level of CAP inclusion (30%) could enhance the lipolysis, lipogenesis, and fatty acids desaturation, while a high level (60%) could inhibit lipogenesis and fatty acids elongation in the muscle of pearl gentian grouper.

## 4. Conclusion

In summary, we found that: (1) fishmeal replacement with CAP (0%, 30%, and 60%) did not alter the structural integrity of muscle; (2) CAP inclusion resulted in large alterations in lipid composition and positional distributions of fatty acids; (3) the levels of PS and ST classes increased significantly with increasing levels of CAP substitution; (4) PC(18 : 3/20 : 5), PC(22 : 6/17 : 1), and SM(d19 : 0/24 : 4) were identified as the potential lipid biomarkers between the CAP treatments; (5) CAP replacement did not affect the fatty acids compositions; (6) 30% CAP inclusion enhanced lipolysis, lipogenesis, and fatty acids desaturation, while 60% CAP inclusion inhibited lipogenesis and fatty acids elongation. These findings provided scientific evidences and novel insights into the nutritional values of dietary CAP in aquatic animals.

## Figures and Tables

**Table 1:** Composition of fatty acids in muscle of pearl gentian grouper in three groups.

Fatty acids	Concentrations (*μ*g/g)		
	CAP-0	CAP-30	CAP-60
6 : 0	0.79 ± 0.11	0.81 ± 0.03	0.73 ± 0.04
8 : 0	0.2 ± 0.05	0.17 ± 0.03	0.14 ± 0.01
10 : 0	0.17 ± 0.05	0.15 ± 0.04	0.11 ± 0.01
11 : 0	0.09 ± 0.02	0.09 ± 0.01	0.08 ± 0.00
12 : 0	0.75 ± 0.30	0.72 ± 0.12	0.77 ± 0.11
13 : 0	1.20 ± 0.50	0.89 ± 0.38	0.48 ± 0.04
14 : 0	24.26 ± 12.22	22.98 ± 5.21	27.50 ± 5.30
15 : 0	6.07 ± 1.33	6.32 ± 0.64	6.69 ± 0.53
16 : 0	540.13 ± 151.10	482.08 ± 98.94	425.79 ± 26.96
17 : 0	11.03 ± 1.93	11.71 ± 0.98	12.54 ± 1.05
18 : 0	363.04 ± 101.58	313.49 ± 72.27	255.01 ± 9.66
20 : 0	6.19 ± 1.92	6.18 ± 0.88	6.87 ± 0.89
21 : 0	0.80 ± 0.29	0.93 ± 0.16	1.06 ± 0.16
22 : 0	1.90 ± 0.97	2.03 ± 0.46	2.49 ± 0.53
23 : 0	0.58 ± 0.26	0.62 ± 0.13	0.68 ± 0.13
24 : 0	1.92 ± 0.80	1.66 ± 0.33	1.74 ± 0.27
SFA	959.1 ± 268.84	850.85 ± 173.62	742.68 ± 45.4
14 : 1T	5.92 ± 2.06	3.48 ± 1.48	1.54 ± 0.23
14 : 1	17.44 ± 1.37	13.04 ± 4.07	8.86 ± 3.01
15 : 1T	2.48 ± 0.5	2.88 ± 0.27	2.84 ± 0.05
15 : 1	5.20 ± 1.07	6.03 ± 0.64	6.19 ± 0.15
16 : 1T	3.05 ± 0.88	3.57 ± 0.32	4.79 ± 0.96
16 : 1	25.25 ± 12.97	24.00 ± 5.08	29.28 ± 5.43
17 : 1T	4.42 ± 0.71	4.76 ± 0.24	5.16 ± 0.41
17 : 1	4.39 ± 0.80	4.27 ± 0.26	3.90 ± 0.22
18 : 1*n*−12T	1.87 ± 0.44	1.50 ± 0.23	1.31 ± 0.11
18 : 1*n*−9T	1.97 ± 0.32^b^	1.30 ± 0.17^a^	0.9 ± 0.13^a^
18 : 1*n*−7T	19.48 ± 3.88	22.66 ± 2.66	22.55 ± 0.63
18 : 1*n*−12	34.82 ± 8.64	51.82 ± 9.65	48.23 ± 6.92
18 : 1*n*−9C	130.38 ± 53.62	138.7 ± 24.92	169.95 ± 30.87
18 : 1*n*−7	32.83 ± 12.34	33.84 ± 5.75	39.33 ± 6.64
19 : 1*n*−12T	4.45 ± 1.66	3.03 ± 0.90	2.00 ± 0.32
19 : 1*n*−9T	3.04 ± 0.93	3.94 ± 0.92	2.79 ± 0.35
20 : 1T	4.10 ± 0.64	4.06 ± 0.24	3.23 ± 0.18
20 : 1	15.75 ± 6.47	16.7 ± 3.12	20.02 ± 3.87
22 : 1*n*−9T	3.24 ± 0.58^b^	2.62 ± 0.37^ab^	1.80 ± 0.10^a^
22 : 1*n*−9	10.75 ± 3.76	13.83 ± 1.99	17.81 ± 3.13
24 : 1	7.03 ± 3.23	6.80 ± 1.28	6.15 ± 0.84
MUFA	329.49 ± 107.25	360.67 ± 56.12	394.21 ± 60.72
20 : 2	7.75 ± 2.14	7.26 ± 0.75	8.07 ± 1.29
22 : 2	1.86 ± 0.55	1.46 ± 0.23	1.23 ± 0.14
22 : 4	2.03 ± 0.74	1.80 ± 0.26	2.10 ± 0.34
18 : 3*n*−3	23.84 ± 12.67	26.02 ± 6.02	32.36 ± 7.37
20 : 3*n*−3	9.48 ± 2.51	10.00 ± 1.11	10.44 ± 1.46
20 : 5*n*−3	97.89 ± 41.1	120.68 ± 23.59	165.56 ± 32.98
22 : 5*n*−3	30.12 ± 10.26	32.99 ± 4.38	46.35 ± 8.93
22 : 6*n*−3	172.85 ± 47.83	183.99 ± 20.05	197.58 ± 27.71
*n*−3 PUFA	334.17 ± 114.02	373.67 ± 54.19	452.3 ± 78.20
18 : 2*n*−6T	0.58 ± 0.26	0.37 ± 0.25	0.04 ± 0.01
18 : 2*n*−6	152.09 ± 65.09	165.26 ± 33.36	190.26 ± 36.37
18 : 3*n*−6	1.04 ± 0.67	0.94 ± 0.28	1.19 ± 0.31
20 : 3*n*−6	2.31 ± 0.75	2.58 ± 0.29	3.02 ± 0.50
20 : 4*n*−6	14.75 ± 3.61	15.50 ± 1.55	17.72 ± 2.01
22 : 5*n*−6	14.21 ± 6.29	12.97 ± 1.08	12.03 ± 0.64
*n*−6 PUFA	184.71 ± 71.51	197.5 ± 36.11	224.24 ± 38.86
*n*−3 PUFA/ *n*−6 PUFA	1.93 ± 0.10	1.95 ± 0.08	2.02 ± 0.03

CAP-0, CAP replacing 0% of fishmeal; CAP-30, CAP replacing 30% of fishmeal; CAP-60, CAP replacing 60% of fishmeal. Values are presented as means with SD, where significant (*p* < 0.05) differences between groups are indicated by different letters.

## Data Availability

The data that support the findings of this study are available from the corresponding author upon reasonable request.

## Abbreviations

*aco*: Acyl-CoA oxidase 1
*atgl*: Adipose triglyceride lipase;
CAP: *Clostridium autoethanogenum* protein
DG: Diacylglycerols
*elovl4*: Elongase of very long-chain fatty acid 4
*fabp*: Fatty acid binding protein
*fad6*: Delta-6 fatty acyl desaturase
*fas*: Fatty acid synthase
*lxr*: Liver X receptor alpha
PC: Phosphatidylcholines
P-PPAR*α*: Phosphor-PPAR*α*
SEM: Scanning electron microscopy
SREBP1: Sterol regulator element-binding protein 1
TG: Triglycerides
*ucp2*: Uncoupling protein 2.

## Supplementary Materials

Supplementary 1 Composition and concentration of nutrients in diets.

Supplementary 2 Informations and percentages of each fatty acids.

Supplementary 3 Calibration curves of each fatty acid.

Supplementary 4 The precision and stability of each fatty acid.

Supplementary 5 Primers designed for qPCR.

Supplementary 6 Quality control and structure of lipidomics in the muscle of pearl gentian grouper. Fold change of lipid classes in the muscle of pearl gentian grouper. The PCA (a), PLS-DA (b), OPLS-DA (c), and hierarchical cluster (d) in the muscle of pearl gentian grouper.

Supplementary 7 The identified lipid species and composition in the muscle of pearl gentian grouper in three groups.

Supplementary 8 Dierential lipid molecules in the muscle of pearl gentian grouper in three groups.

Supplementary 9 Receiver operating characteristic curve of fatty acids in CAP-0 and CAP-30 groups.

Supplementary 10 Receiver operating characteristic curve of fatty acids in CAP-30 and CAP-60 groups.
