# Supplementary material for: Replacement of Dietary Fishmeal with Clostridium autoethanogenum Protein on Lipidomics and Lipid Metabolism in Muscle of Pearl Gentian Grouper
Source: Aquac Nutr. 2023 Jun 30;2023:6723677. doi: 10.1155/2023/6723677 (PMC10328730; doi:10.1155/2023/6723677)
Supplement: Supplementary 1 — Composition and concentration of nutrients in diets. [file 6723677.f1.pdf]

Table S1 Composition and concentration of nutrients in diets

Ingredients (%)	Diets		
	CAP-0	CAP-30	CAP-60
Brown fish meal	50	35	20
Clostridium autoethanogenum protein	0	12	24
Gluten	4	4	4
Soybean meal	13	13	13
Wheat flour	21	21	21
Fish oil	2.5	3.82	5.15
Soybean oil	2	2	2
Soybean lecithin	1.5	1.5	1.5
Choline chloride	0.5	0.5	0.5
Vitamin C	0.1	0.1	0.1
CaH_2PO_4	1.5	1.5	1.5
Compound premixa	1	1	1
Ethoxyquin	0.05	0.05	0.05
Microcrystalline cellulose	2.85	4.29	5.73
Methionine	0	0.03	0.06
Arginine	0	0.21	0.41
Total	100	100	100
Proximate composition (%)			
Crude protein	48.45	48.94	48.31
Crude fat	12.71	12.61	11.94
Moisture	9.97	9.2	9.1
