# Supplementary material for: Replacement of Dietary Fishmeal with Clostridium autoethanogenum Protein on Lipidomics and Lipid Metabolism in Muscle of Pearl Gentian Grouper
Source: Aquac Nutr. 2023 Jun 30;2023:6723677. doi: 10.1155/2023/6723677 (PMC10328730; doi:10.1155/2023/6723677)
Supplement: Supplementary 2 — Informations and percentages of each fatty acids. [file 6723677.f2.pdf]

Table S2 Informations and percentages of each fatty acids

Abbreviations	Full names	Composition in standard solutions (%)
C6:0	Caproate	2
C8:0	Caprylate	2
C10:0	Caprate	1
C11:0	Unndecanoate	2
C12:0	Laurate	1
C13:0	Tridecanoate	2
C14:0	Myristate	2
C14:1T	Myristelaidate	1
C14:1	Myristoleate	2
C15:0	Pentadecanoate	2
C15:1T	10-Transpentadecenoate	1
C15:1	10-Pentadecenoate	2
C16:0	Palmitate	3
C16:1T	Palmitelaidate	1
C16:1	Palmitoleate	2
C17:0	Heptadecanoate	3
C17:1T	10-Transsheptadecenoate	2
C17:1	10-Heptadecenoate	2
C18:0	Stearate	2
C18:1N12T	Petroselaidate	2
C18:1N9T	Elaidate	3
C18:1N7T	Transvaccenate	1
C18:1N12	Petroselinate	2
C18:1N9C	Oleate	2
C18:1N7	Vaccenate	2
C18:2N6T	Linoelaidate	2
C19:1N12T	7-Transnonadecenoate	1
C19:1N9T	10-Transnonadecenoate	2
C18:2N6	Linoleate	3
C20:0	Arachidate	2
C18:3N6	Gamma Linolenate	3
C20:1T	Trans 11-Eicosenoate	1
C20:1	11-Eicosenoate	2
C18:3N3	Alpha Linolenate	3
C21:0	Heneicosanoate	2
C20:2	11-14 Eicosadienoate	3
C22:0	Behenate	1
C20:3N6	Homogamma Linolenate	2
C22:1N9T	Brassidate	1
C22:1N9	Erucate	2
C20:3N3	11-14-17 Eicosatrienoate	2
C20:4N6	Arachidonate	3
C23:0	Tricosanoate	2
C22:2	Docosadienoate	2
C20:5N3	Eicosapentaenoate	2
C24:0	Lignocerate	1
C24:1	Nervonoate	1

C22:4	Docosatetraenoate	2
C22:5N6	Docosapentaenoate	2
C22:5N3	Docosapentaenoate	2
C22:6N3	Docosahexaenoate	2
