# Supplementary material for: Replacement of Dietary Fishmeal with Clostridium autoethanogenum Protein on Lipidomics and Lipid Metabolism in Muscle of Pearl Gentian Grouper
Source: Aquac Nutr. 2023 Jun 30;2023:6723677. doi: 10.1155/2023/6723677 (PMC10328730; doi:10.1155/2023/6723677)
Supplement: Supplementary 3 — Calibration curves of each fatty acid. [file 6723677.f3.pdf]

**Table S3 Calibration curves of each fatty acids**

| Fatty acids | Linear equation | Correlation coefficient | Linear range |
|---|---|---|---|
| C6:0 | y=0.0416x + 0.000208 | 0.999 | 0.02-40 |
| C8:0 | y=0.082x + 0.000211 | 0.999 | 0.02-40 |
| C10:0 | y=0.116x + 0.000149 | 1 | 0.01-20 |
| C11:0 | y=0.116x + 0.000281 | 0.998 | 0.02-40 |
| C12:0 | y=0.113x + 0.00016 | 0.999 | 0.01-20 |
| C13:0 | y=0.105x + 7.82e-05 | 0.999 | 0.02-40 |
| C14:0 | y=0.0944x + 0.000273 | 0.999 | 0.02-40 |
| C14:1T | y=0.0404x + 0.00568 | 0.992 | 0.01-20 |
| C14:1 | y=0.0332x + 0.0823 | 0.992 | 0.02-40 |
| C15:0 | y=0.0945x + 0.000251 | 0.999 | 0.02-40 |
| C15:1T | y=0.041x + 0.00193 | 0.998 | 0.01-20 |
| C15:1 | y=0.0384x + 0.00199 | 0.999 | 0.02-40 |
| C16:0 | y=0.0839x + 0.00694 | 0.997 | 0.03-60 |
| C16:1T | y=0.0296x + 0.00228 | 0.998 | 0.01-20 |
| C16:1 | y=0.0296x + 0.00279 | 0.999 | 0.02-40 |
| C17:0 | y=0.0774x + 0.00185 | 0.998 | 0.03-60 |
| C17:1T | y=0.0298x + 0.00228 | 0.998 | 0.02-40 |
| C17:1 | y=0.03x + 0.0023 | 0.998 | 0.02-40 |
| C18:0 | y=0.0722x + 0.00767 | 0.998 | 0.02-40 |
| C18:1N12T | y=0.0374x + 0.00283 | 0.996 | 0.02-10 |
| C18:1N9T | y=0.0773x + 0.00275 | 0.995 | 0.01-20 |
| C18:1N7T | y=0.0102x + 0.00219 | 0.997 | 0.03-60 |
| C18:1N12 | y=0.0183x + 0.00718 | 0.996 | 0.02-40 |
| C18:1N9C | y=0.0278x + 0.00925 | 0.998 | 0.02-40 |
| C18:1N7 | y=0.0294x + 0.00366 | 0.998 | 0.02-40 |
| C18:2N6T | y=0.0312x + 0.00181 | 0.998 | 0.02-40 |
| C19:1N12T | y=0.0278x + 0.00277 | 0.998 | 0.02-20 |
| C19:1N9T | y=0.055x + 0.00279 | 0.998 | 0.01-20 |
| C18:2N6 | y=0.045x + 0.0061 | 0.997 | 0.02-40 |
| C20:0 | y=0.0609x + 0.000305 | 0.999 | 0.02-40 |
| C18:3N6 | y=0.0251x + 0.00167 | 0.999 | 0.03-60 |
| C20:1T | y=0.0233x + 0.00187 | 0.995 | 0.01-20 |
| C20:1 | y=0.0323x + 0.00245 | 0.996 | 0.02-20 |
| C18:3N3 | y=0.0315x + 0.00215 | 0.998 | 0.03-60 |
| C21:0 | y=0.0481x + 0.00014 | 0.996 | 0.02-40 |
| C20:2 | y=0.0221x + 0.00137 | 0.996 | 0.03-60 |
| C22:0 | y=0.0379x + 0.000177 | 0.995 | 0.01-20 |
| C20:3N6 | y=0.0182x + 0.000931 | 0.995 | 0.02-40 |
| C22:1N9T | y=0.0166x + 0.00186 | 0.99 | 0.01-20 |
| C22:1N9 | y=0.00914x + 0.00181 | 0.993 | 0.02-40 |
| C20:3N3 | y=0.0216x + 0.00117 | 0.996 | 0.02-10 |

| | | | |
|---|---|---|---|
| C20:4N6 | y=0.0178x + 0.000988 | 0.996 | 0.03-60 |
| C23:0 | y=0.0309x + 3.36e-05 | 0.992 | 0.02-40 |
| C22:2 | y=0.0154x + 0.00119 | 0.991 | 0.02-40 |
| C20:5N3 | y=0.0176x + 0.000471 | 0.994 | 0.1-40 |
| C24:0 | y=0.0412x + 0.000326 | 0.991 | 0.01-20 |
| C24:1 | y=0.0297x + 0.00329 | 0.99 | 0.05-20 |
| C22:4 | y=0.0327x + 0.00249 | 0.993 | 0.02-40 |
| C22:5N6 | y=0.0351x + 0.00308 | 0.996 | 0.1-40 |
| C22:5N3 | y=0.0417x + 0.00492 | 0.995 | 0.1-40 |
| C22:6N3 | y=0.0393x + 0.00723 | 0.993 | 0.02-40 |
