# Supplementary material for: Replacement of Dietary Fishmeal with Clostridium autoethanogenum Protein on Lipidomics and Lipid Metabolism in Muscle of Pearl Gentian Grouper
Source: Aquac Nutr. 2023 Jun 30;2023:6723677. doi: 10.1155/2023/6723677 (PMC10328730; doi:10.1155/2023/6723677)
Supplement: Supplementary 4 — The precision and stability of each fatty acid. [file 6723677.f4.pdf]

Table S4 The precision and stability of each fatty acids

Fatty acids	Sample concentration (μg/mL)	Inter-day precision (%)	Intra-day precision (%)	Stability (%)
	0.2	7.29	13. 11	
C6:0	1	0.8	13. 10	93.43
	10	0.47	8.27	
	0.2	7.08	9.54	
C8:0	1	1.08	11.33	98.31
	10	0.69	3.98	
	0.1	7. 16	5.43	
C10:0	0.5	0.56	10.57	96.84
	5	0.65	3.44	
	0.2	6.5	7.82	
C11:0	1	0.35	10. 13	94.62
	10	1.02	4.26	
	0.1	7.03	8.02	
C12:0	0.5	0.48	10.62	98.48
	5	0.57	3. 18	
	0.2	7. 13	8.7	
C13:0	1	0.51	10.76	96.76
	10	1.02	3.8	
	0.2	5.58	8.69	
C14:0	1	0.39	10.06	97.49
	10	1.26	3.68	
	0.1	4.41	5.49	
C14:1T	0.5	1. 10	4.2	97.75
	5	0.75	3.61	
	0.2	7.74	12.76	
C14:1	1	2.07	11. 15	89.23
	10	1.25	4. 14	
	0.2	6.2	6.57	
C15:0	1	0.37	9.74	98.52
	10	1.37	3.71	
	0.1	12.47	10.87	
C15:1T	0.5	3.05	13.5	98.1
	5	1. 13	2.48	
	0.2	7.91	12.33	
C15:1	1	1.25	10.76	97.95
	10	1.21	3.31	
	0.3	7.59	9.4	
C16:0	1.5	0.37	10.75	98.33
	15	1.79	1.9	
	0.1	11.91	14.69	
C16:1T	0.5	4.41	13.73	90.83
	5	0.99	3.78	
	0.2	7.03	7.2	
C16:1	1	2. 10	7.34	98.98
	10	1.32	2.71	
	0.3	8.93	2.73	
C17:0	1.5	0.33	9.4	97.87
	15	1.65	1.9	

	0.2	6.6	3.75	
C17:1T	1	1.47	9.37	98.54
	10	1.41	3. 11	
	0.2	10.81	6.64	
C17:1	1	1.43	10.93	95.49
	10	1.37	3.53	
	0.2	7.99	11.41	
C18:0	1	0.49	14.24	97.95
	10	1.48	3.66	
	0.2	9.49	13.69	
C18:1N12T	1	5.94	8.89	95.86
	10	11.26	9. 11	
	0.3	12.94	8.36	
C18:1N9T	1.5	2.82	8.4	98.36
	15	8. 15	5.69	
	0.1	6.25	6.89	
C18:1N7T	0.5	5	8.03	97.84
	5	2.2	4.24	
	0.2	11.77	11.51	
C18:1N12	1	2.34	7.96	94.27
	10	2.22	2.56	
	0.2	5.49	1.93	
C18:1N9C	1	4. 10	12.71	97.34
	10	3. 19	2.88	
	0.2	4.89	9.41	
C18:1N7	1	3	4.81	97.82
	10	2.66	2. 12	
	0.2	6.41	7.8	
C18:2N6T	1	1.01	11.32	97.61
	10	1.44	3. 19	
	0.1	12.94	3.6	
C19:1N12T	0.5	5.27	7.96	98.02
	5	1.67	2.55	
	0.2	13.25	11.08	
C19:1N9T	1	1.96	9.08	93.5
	10	2.29	1.8	
	0.3	8. 10	7. 15	
C18:2N6	1.5	1.77	10.85	98.6
	15	1.73	1.77	
	0.2	7. 15	7.63	
C20:0	1	0.88	8.94	97.77
	10	1.84	2.25	
	0.3	7.74	4.86	
C18:3N6	1.5	1.3	8.31	98.28
	15	1.55	1.83	
	0.1	7.73	8.26	
C20:1T	0.5	3.68	10.85	98.11
	5	0.92	1.88	
	0.2	13.32	5.53	
C20:1	1	2.87	6.86	96.62
	10	1.97	1.85	
	0.3	11.61	11.72	
C18:3N3	1.5	1.45	10.52	98.89

	15	1.94	2.35	
	0.2	4.03	1. 17	
C21:0	1	0.93	5.85	97.95
	10	2. 14	2.02	
	0.3	8.7	3.06	
C20:2	1.5	0.52	10.08	98.43
	15	2.02	1. 16	
	0.1	9. 19	6.38	
C22:0	0.5	2.72	5.63	97.45
	5	0.91	3.09	
	0.2	9.69	12. 10	
C20:3N6	1	2.74	8.46	96.59
	10	1.5	3.64	
	0.1	13.24	13.33	
C22:1N9T	0.5	3.59	3.45	97.69
	5	1.92	1.44	
	0.2	9.78	11.94	
C22:1N9	1	4. 15	11.5	97.16
	10	1.98	1. 15	
	0.2	14.38	14.02	
C20:3N3	1	3.04	8.89	92.18
	10	2.26	5.08	
	0.3	5.5	8.44	
C20:4N6	1.5	3.31	8.97	98.63
	15	1.73	1.84	
	0.2	4.2	6.41	
C23:0	1	2.6	8.72	97.56
	10	1.97	2.91	
	0.2	5.64	7.53	
C22:2	1	2. 16	10.84	97.74
	10	1.38	2.54	
	0.2	6.7	8.08	
C20:5N3	1	0.5	11.72	97.81
	10	1.5	3.71	
	0.1	12.39	4.88	
C24:0	0.5	1. 19	4.63	98.77
	5	1.83	1.78	
	0.1	12.08	3.45	
C24:1	0.5	9.39	9.77	97.52
	5	1.56	1.75	
	0.2	9.08	14.89	
C22:4	1	3.22	5.39	96.61
	10	1.97	1.26	
	0.2	5. 11	5.77	
C22:5N6	1	2.37	9.2	93.51
	10	1.84	0.99	
	0.2	6.92	6.06	
C22:5N3	1	2. 11	11.4	96.51
	10	1.51	0.87	
	0.2	11.81	9.34	
C22:6N3	1	1.45	7.65	97.67
	10	1.3	1.88	
