# Supplementary material for: Replacement of Dietary Fishmeal with Clostridium autoethanogenum Protein on Lipidomics and Lipid Metabolism in Muscle of Pearl Gentian Grouper
Source: Aquac Nutr. 2023 Jun 30;2023:6723677. doi: 10.1155/2023/6723677 (PMC10328730; doi:10.1155/2023/6723677)
Supplement: Supplementary 5 — Primers designed for qPCR. [file 6723677.f5.pdf]

Table S5 Primers designed for qPCR

Target	Sequences	Primer efficiency (%)
elovl4	F: ACAACTTTGCCATGGTCGGC<br>R: ATCATGGTGCCGTGGTGGTA	96.41
elovl8	F: CAGATGATCCAGTTCCACGTCA<br>R: GCGGTAGGTCTGGTAGTAGAAG	96.03
fabp	F: GTTGTCACCTCCAAGACTCCG<br>R: GACCATTTTGCCACCCTCC	105.68
fas	F: CGGGTGTCTACATTGGGGTG<br>R: GAATAGCGTGGAAGGCGTTT	99.77
srebp1	F: TGTATCCAACTGTTGAGCACCTG<br>R: CTGTGGCAGTGTGGTCCTAG	99.85
pparr	F: GCGCCAGACACACACAATTT<br>R: CACTCGATGTTTAGCGCTGC	101.34
atgl	F: ATTGAGCACCTTCCACCCA<br>R: CCGAATCCATCCCACATCTT	99.41
ppara	F: CATCGACAATGACGCCCTC<br>R: GCCGCTATCCCGTAAACAAC	99.51
aco	F: CGGCATGGACTTCCTGTATG<br>R: CCTGGTGTGCGTGTTGTGTT	98.48
fad6	F: GGACTTCGCCCCAGCTTTATT<br>R: TGTTGAACCGGTCAGTTCGGA	95.95
ucp2	F: TACGATTTCATCAAGGATACAC<br>R: GTGGTCCCTCTTTGGTCAT	101.95
lxr	F: CAGAAGCAATGCAACAAAAGG<br>R: TCAGTGAAGTGGGCGAACC	92.44
