# Supplementary material for: Replacement of Dietary Fishmeal with Clostridium autoethanogenum Protein on Lipidomics and Lipid Metabolism in Muscle of Pearl Gentian Grouper
Source: Aquac Nutr. 2023 Jun 30;2023:6723677. doi: 10.1155/2023/6723677 (PMC10328730; doi:10.1155/2023/6723677)
Supplement: Supplementary 6 — Quality control and structure of lipidomics in the muscle of pearl gentian grouper. Fold change of lipid classes in the muscle of pearl gentian grouper. The PCA (a), PLS-DA (b), OPLS-DA (c), and hierarchical cluster (d) in the muscle of pearl gentian grouper. [file 6723677.f6.docx]

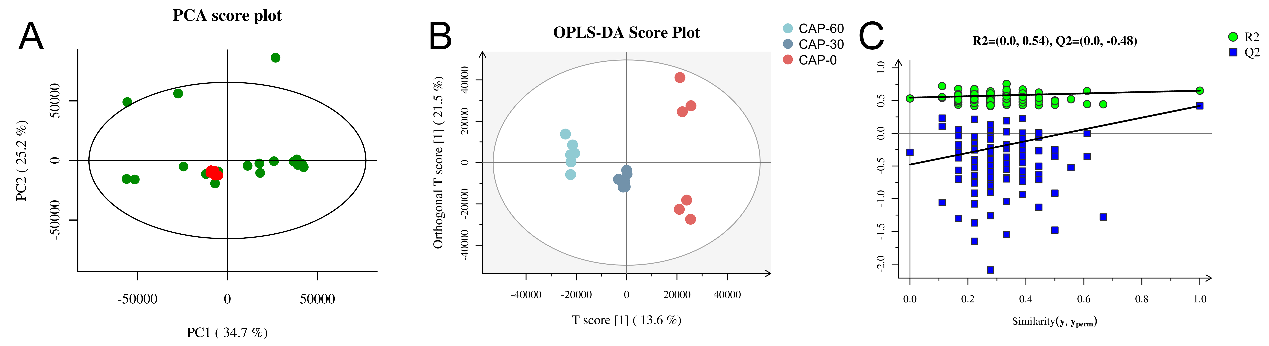


**Fig. S1** Quality control and structure of lipidomics in muscle of pearl gentian grouper. A: In PCA plot, the quality control sample is red and the tested sample is green. A smaller difference in quality control sample means a more stable system. B: the OPLS-DA scores in positive and negative ion modes. C: the validation plots of OPLS-DA analysis. CAP-0: CAP replacing 0% of fishmeal; CAP-30: CAP replacing 30% of fishmeal; CAP-60: CAP replacing 60% of fishmeal.


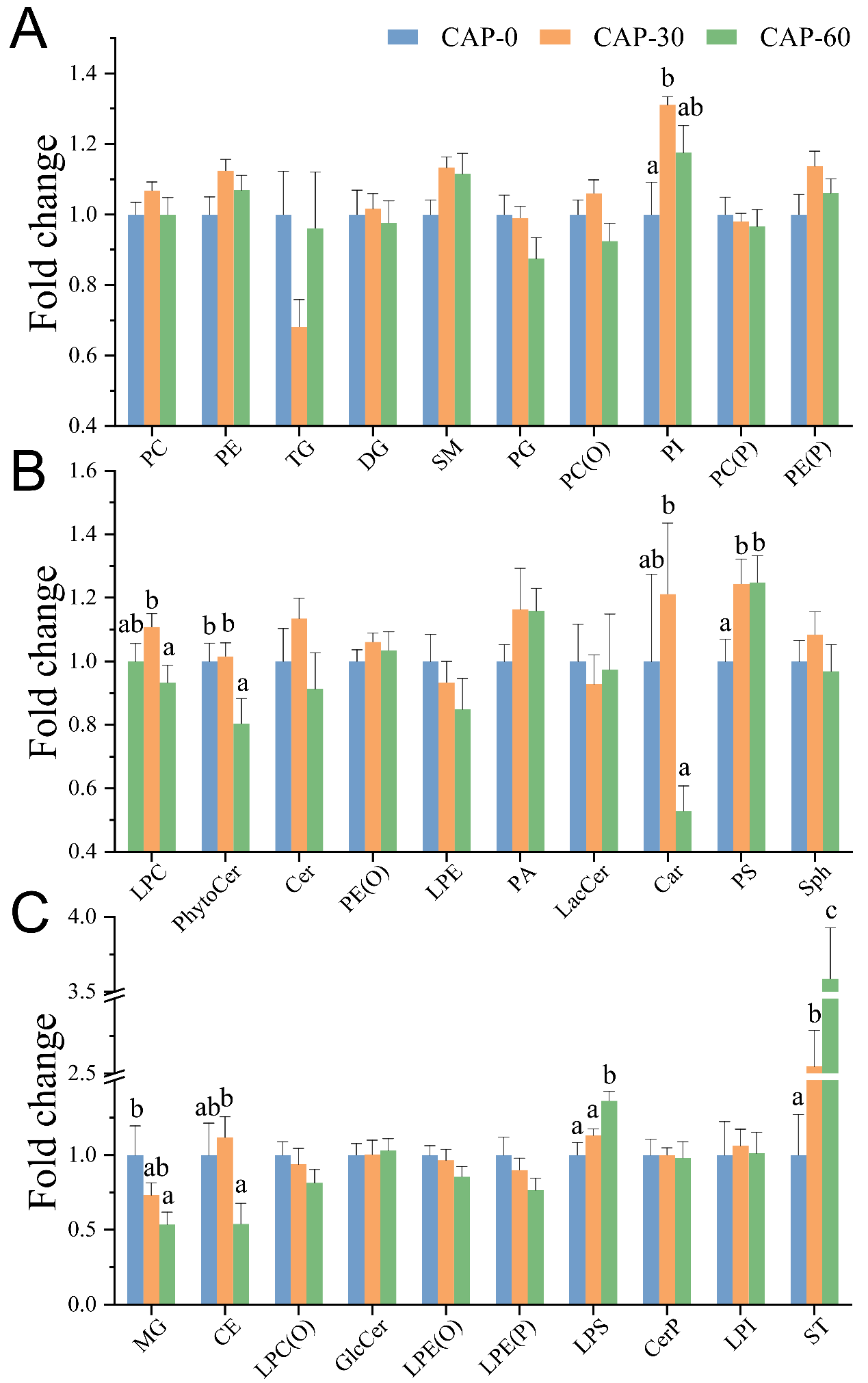


**Fig. S2** Fold change of lipid classes in muscle of pearl gentian grouper. CE: cholesterol ester, Cer: ceramides, LPC: lysophosphatidylcholine, LPE: lysophosphatidylethanolamine, LPG: lysophosphatidylglycerol, PC(O): alkylphosphatidylcholine, PC(P): alkenylphosphatidylcholine, PE: phosphatidylethanolamines, PE(P): alkenylphosphatidylethanolamine, PG: phosphatidylglycerol, PhytoCer: phytoceramides, PI: phosphatidylinositol. CAP-0: CAP replacing 0% of fishmeal; CAP-30: CAP replacing 30% of fishmeal; CAP-60: CAP replacing 60% of fishmeal. Values are presented as means with SD, where signiﬁcant (*p* < 0.05) differences between groups are indicated by different letters.


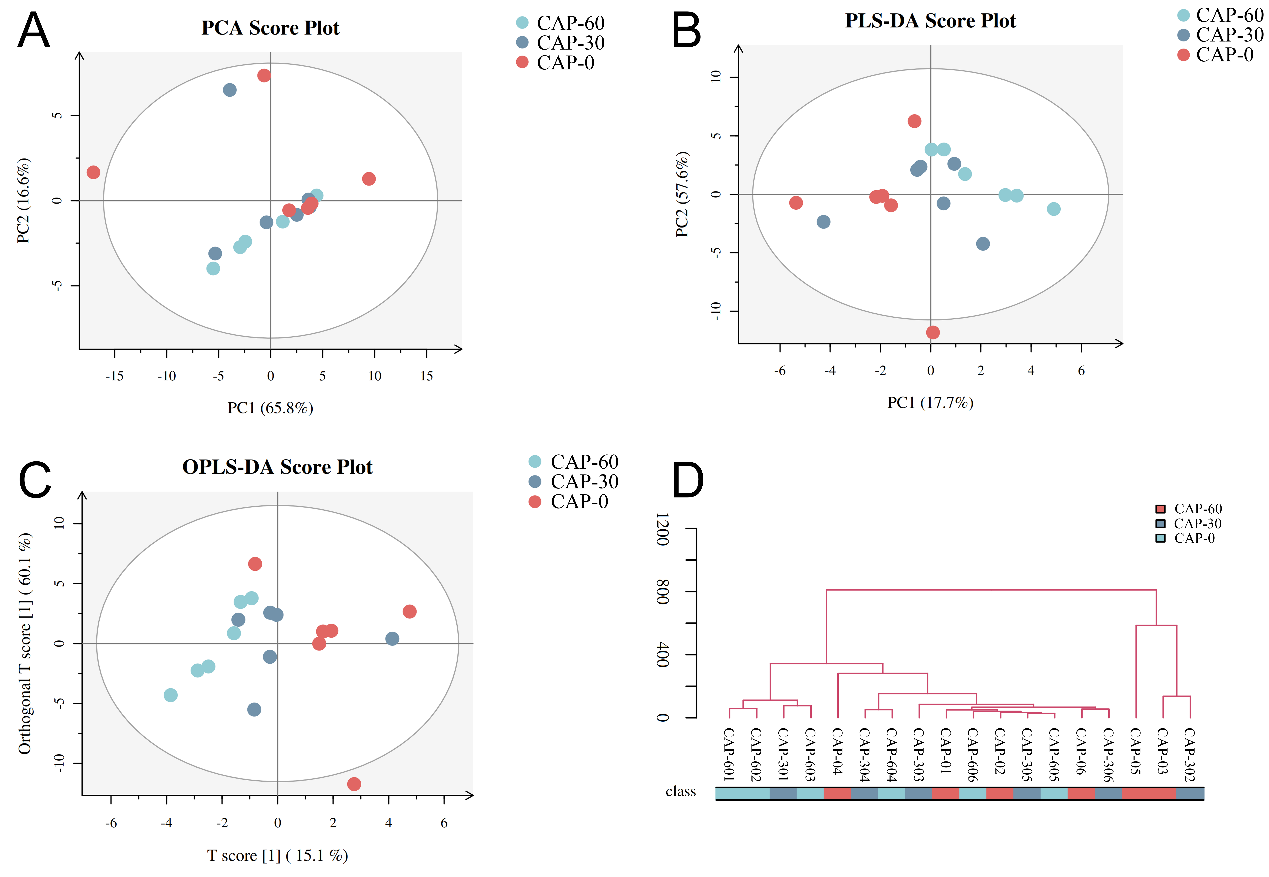


**Fig. S3** The PCA (A), PLS-DA (B), OPLS-DA (C), and hierarchical cluster (D) in muscle of pearl gentian grouper. CAP-0: CAP replacing 0% of fishmeal; CAP-30: CAP replacing 30% of fishmeal; CAP-60: CAP replacing 60% of fishmeal.
