# Supplementary material for: Replacement of Dietary Fishmeal with Clostridium autoethanogenum Protein on Lipidomics and Lipid Metabolism in Muscle of Pearl Gentian Grouper
Source: Aquac Nutr. 2023 Jun 30;2023:6723677. doi: 10.1155/2023/6723677 (PMC10328730; doi:10.1155/2023/6723677)
Supplement: Supplementary 7 — The identified lipid species and composition in the muscle of pearl gentian grouper in three groups. [file 6723677.f7.pdf]

Table S6 The identified lipid species and composition in muscle of pearl gentian grouper

name	Class	MainIon	mz	rt	CAP-01	CAP-02	CAP-03
Car(6:0)	Car	Car+H	260.1924	45.177	3260.98	9318.170583	4188.517364
Sph(d16:1	Sph	Sph+H	272.2651	87.312	24561.01747	16417.143	32244.37107
Sph(d16:0	Sph	Sph+H	274.2814	63.0975	1300002.201	1084122.629	1375566.769
Sph(d17:0	Sph	Sph+H	288.2969	74.999	10182611.58	10067428.93	12637200.02
Sph(d18:1	Sph	Sph+H	300.2949	67.445	8653.951158	8841.491053	11066.66263
Sph(d18:0	Sph	Sph+H	302.3109	78.4805	344565.3637	274019.6427	308763.112
Sph(d19:0	Sph	Sph+H	316.3276	102.1365	9413375.66	9800435.667	18241868.28
Sph(d20:1	Sph	Sph+H	328.3255	90.186	8722.637415	12232.88544	17823.69136
Car(12:0)	Car	Car+H	344.2834	58.2905	54713.64	105536.4538	40924.10974
MG(18:3/(	MG	MG+H	353.2731	117.334	131780.2053	167492.1233	843894.5756
MG(18:2/(	MG	MG+H	355.2879	108.1625	132241.9328	70607.37459	234055.2351
Car(13:1)	Car	Car+H	356.2827	57.162	33854.61469	11922.6049	23885.12033
Sph(d22:1	Sph	Sph+H	356.356	111.788	229078.157	180140.5278	247618.2304
Sph(d22:0	Sph	Sph+H	358.3726	149.259	589976.2853	511480.5041	904732.4487
Car(14:1)	Car	Car+H	370.2979	61.547	55394.02157	108187.261	40615.85014
DG(7:0/11	DG	DG+NH4	390.3234	76.1355	13677.13568	8354.320227	18360.58258
Car(16:1)	Car	Car+H	398.3283	73.6965	153605.648	225913.342	97148.52393
Car(16:0)	Car	Car+H	400.3442	91.475	845444.994	1454311.642	299868.1397
MG(22:6/(	MG	MG+H	403.2837	118.626	58661.01162	40547.43984	114423.5098
MG(22:5/(	MG	MG+H	405.2915	94.1645	32944.0363	17707.38567	14728.08333
LPC(11:0/	LPC	LPC+H	426.2708	96.47	254859.6804	60766.801	526431.7404
Car(18:1)	Car	Car+H	426.348	78.2305	29368.35147	157968.9176	31862.79595
Car(18:0)	Car	Car+H	428.3746	126.872	734290.3049	889881.5481	280978.9309
Car(20:5)	Car	Car+H	446.327	65.0985	413499.5092	764664.1231	354899.7741
PC(2:0/10:	PC	PC+H	454.2595	77.9065	81293.62574	60442.76811	66839.92033
LPC(13:0/	LPC	LPC+H	454.299	165.396	63434.52385	75494.873	43994.46505
Car(20:1)	Car	Car+H	454.3867	130.822	67307.34019	72063.00531	42244.03182
Car(20:0)	Car	Car+H	456.4027	183.7435	34996.48625	40333.45005	32974.22935
LPE(P-18:	LPE(P)	LPE(P)+H	466.3284	137.6515	138544.2576	119073.6458	204755.6544
Car(22:5)	Car	Car+H	474.3564	77.6005	287785.1242	432894.3787	140239.8351
LPC(O-16:	LPC(O)	LPC(O)+H	482.3585	103.9735	852596.8824	775949.4768	853026.7748
SM(d17:0/	SM	SM+H	495.3597	98.011	5311.862769	3353.33109	4830.639819
LPC(17:2/	LPC	LPC+H	506.3208	69.1165	20392.14319	19917.05907	25211.09717
Cer(d18:1/	Cer	Cer+H	510.4823	389.389	34621.28716	37833.17262	47725.92
LPC(18:3/	LPC	LPC+H	518.3202	64.898	376747.4331	307728.779	370622.3162
SM(d19:2/	SM	SM+H	519.3663	137.075	7642.825	5023.8606	8067.890208
LPC(18:0/	LPC	LPC+H	524.3687	119.61	1356150.996	940836.9501	1075667.335
LPC(O-18:	LPC(O)	LPC(O)+N	530.3531	109.044	10650.97882	8517.49925	12930.842
PC(O-16:2	PC(O)	PC(O)+H	534.3489	88.529	14443.89405	55926.24458	88810.29496
PC(2:0/16:	PC	PC+H	536.3289	51.9695	23343.74463	12995.66107	9653.358086
PC(P-16:0/	PC(P)	PC(P)+H	536.3665	106.582	28812.33	26659.83206	36424.75536
LPC(19:0/	LPC	LPC+H	538.3816	141.973	17065.31767	15560.96253	21907.53882
LPC(18:1/	LPC	LPC+Na	544.3321	88.431	436029.3121	418366.5131	435515.1771
PC(O-18:2	PC(O)	PC(O)+H	548.3654	96.813	246108.6313	187607.3196	299277.4454
LPC(20:1/	LPC	LPC+H	550.3826	124.853	232469.1368	210568.5496	248832.1189
DG(12:0/1	DG	DG+NH4	552.4542	367.744	10509.93905	11314.56272	19430.10701

Cer(d18:1/	Cer	Cer+H	552.5268	464.127	16468.2	9185.619692	13449.76093
PE(18:3/3:	PE	PE+Na	554.2819	69.854	4089.868526	14261.37779	10981.00008
PC(2:0/18:	PC	PC+H	560.3293	71.211	9059.502444	13084.71362	12584.41871
PC(2:0/18:	PC	PC+H	562.344	74.8385	4509.898319	27924.32813	2785.419882
LPC(20:5/	LPC	LPC+Na	564.3005	63.151	128218.4914	163807.4313	200692.2786
PC(2:0/18:	PC	PC+H	566.3741	101.2195	8002.982016	7551.095199	6674.989216
Cer(d18:1/	Cer	Cer+H	566.5421	486.112	113341.7682	9218.234884	33258.33554
DG(2:0/29	DG	DG+NH4	572.5146	497.349	50180.90041	50180.5887	90934.71917
LPC(20:0/	LPC	LPC+Na	574.3765	171.103	4667.828316	4118.447721	7396.015647
PC(3:0/18:	PC	PC+H	576.3598	87.465	7816.3756	16920.35443	18565.956
DG(14:1/1	DG	DG+NH4	576.4503	257.831	13546.04663	11645.7728	14982.76096
SM(d15:1/	SM	SM+H	577.4411	167.456	4168.121804	2158.742846	3816.783
PC(4:0/17:	PC	PC+H	578.3854	73.793	11354.52333	8120.643	10297.37287
PC(P-20:0/	PC(P)	PC(P)+H	578.4103	175.239	25553.69052	21071.72557	27106.92552
LPC(22:0/	LPC	LPC+H	580.4267	235.0615	7445.0902	6045.468	10642.34255
DG(14:0/1	DG	DG+NH4	584.515	481.563	981207.0858	978446.3587	1802058.231
DG(16:0/1	DG	DG+NH4	586.5307	517.342	1365757.592	894701.7477	1311658.477
LPC(22:6/	LPC	LPC+Na	590.3151	68.062	376845.5441	402225.1498	423478.0583
PC(2:0/20:	PC	PC+H	590.373	96.561	11552.92005	39014.17637	47808.61343
Cer(d18:1/	Cer	Cer+H	592.5556	488.752	12787.60269	13557.85276	15574.01044
PC(6:0/16:	PC	PC+H	594.4062	107.588	9805.428	6226.544381	8859.92985
LPC(23:0/	LPC	LPC+H	594.439	187.284	3734.685791	2048.508856	2779.77
DG(18:3/1	DG	DG+NH4	594.4989	438.074	27230.60776	21218.06456	42788.35222
Cer(d20:1/	Cer	Cer+H	594.571	526.123	124844.1188	17100.12667	33810.075
DG(15:0/1	DG	DG+NH4	596.5137	468.381	195455.3654	202131.206	375125.9738
DG(18:2/1	DG	DG+NH4	596.5216	520.89	121989.9873	115994.035	184854.9493
DG(16:0/1	DG	DG+NH4	598.5303	502.149	245970.1096	241039.8977	438537.5505
DG(17:0/1	DG	DG+NH4	600.5581	522.578	67298.28233	61859.82844	111141.1685
DG(12:0/2	DG	DG+NH4	602.4675	355.624	167297.1523	170640.8559	356008.0751
DG(12:0/2	DG	DG+NH4	604.4836	402.299	213814.4801	243139.6479	519258.0587
DG(16:1/1	DG	DG+NH4	606.4983	418.266	271227.843	297275.3778	620695.9253
DG(14:0/2	DG	DG+NH4	606.4982	434.101	199418.0341	205739.5234	285778.5086
Cer(d17:1/	Cer	Cer+H	606.5694	508.416	7723.840684	8051.456077	10100.24412
LPC(24:0/	LPC	LPC+H	608.4558	301.945	39525.91842	40934.32368	62177.09876
DG(16:0/1	DG	DG+NH4	608.5144	457.3075	1165509.9	1116994.54	2316022.155
Cer(d18:1/	Cer	Cer+H	608.5852	544.2	17360.37369	10874.48301	21268.58526
PC(2:0/22:	PC	PC+H	610.3625	82.451	20960.60449	6871.69693	8446.535722
DG(16:0/1	DG	DG+NH4	610.5307	490.0585	8981623.091	8865209.12	15879329.03
DG(16:0/1	DG	DG+NH4	612.5452	520.873	0	522.440094	1199.698079
DG(16:0/1	DG	DG+NH4	614.5738	537.578	2537798.556	876447.4768	1122335.711
DG(17:1/1	DG	DG+NH4	618.4975	420.073	55484.44355	55943.84863	127005.412
DG(15:0/2	DG	DG+NH4	618.5068	461.292	188131.4335	173511.3064	410220.0432
DG(18:3/1	DG	DG+NH4	620.5143	449.838	42151.801	99492.50286	104470.3707
Cer(d18:1/	Cer	Cer+H	620.5844	526.979	267235.0281	238554.5655	345645.0827
DG(17:1/1	DG	DG+NH4	622.5284	479.949	235018.1629	230689.4755	412786.436
DG(17:0/1	DG	DG+NH4	622.5378	525.7635	32043.03016	21223.79989	62643.64158
Cer(t15:1/	PhytoCer	PhytoCer+	622.5631	529.762	47188.22985	57295.85775	69581.40534
Cer(d18:1/	Cer	Cer+H	622.6011	561.431	249622.0098	91542.40766	185455.6043

DG(18:1/1	DG	DG+NH4	624.5441	510.208	12309.48606	5213.904873	26094.98097
Cer(t14:1/2	PhytoCer	PhytoCer+	624.5797	563.269	38669.69979	41132.22949	57434.98896
DG(18:4/1	DG	DG+NH4	626.4681	340.2455	88766.93769	114090.675	121732.4229
DG(18:1/1	DG	DG+NH4	626.5592	538.752	164504.6925	167221.6047	291817.4959
DG(18:3/1	DG	DG+NH4	628.482	365.0765	108222.1515	130901.3555	163301.2234
DG(18:2/1	DG	DG+NH4	630.4978	401.6845	203400.0766	215180.8306	418874.6258
DG(18:4/1	DG	DG+NH4	630.4969	422.477	636021.4388	612562.0672	1327022.121
Cer(d17:0/	Cer	Cer+H	630.5703	500.098	91482.74977	93259.58421	128488.3444
PC(5:0/20:	PC	PC+H	632.4178	111.173	37849.07938	17545.26457	25492.68896
DG(18:2/1	DG	DG+NH4	632.5123	427.965	645689.9902	633433.7598	1128259.957
DG(18:4/1	DG	DG+NH4	632.5134	451.584	2133492.12	2170210.164	4099303.073
PC(P-22:0/	PC(P)	PC(P)+H	634.4685	301.915	10820.46055	9074.191455	14855.99383
DG(18:2/1	DG	DG+NH4	634.5304	461.2785	5975603.006	5957958.498	12572252.6
Cer(d17:1/	Cer	Cer+H	634.6004	544.0675	88324.37007	79734.99049	112191.3184
DG(18:1/1	DG	DG+NH4	636.545	493.911	2254.11954	1102.114685	1352.700961
Cer(d18:1/	Cer	Cer+H	636.6157	577.77	234856.6875	67922.06726	150168.7734
PC(4:0/22:	PC	PC+H	638.3915	89.393	17382.60423	8730.45056	9759.934289
DG(18:0/1	DG	DG+NH4	638.5594	527.93	3972380.569	3152931.018	6408425.288
Cer(d18:0/	Cer	Cer+H	638.6298	589.329	25015.35615	8109.983826	19435.57638
Cer(d18:2/	Cer	Cer+H	640.5652	527.832	395098.7204	292340.3911	533564.72
DG(18:1/1	DG	DG+NH4	640.575	555.758	1546208.385	1122100.103	1885505.715
DG(18:0/1	DG	DG+NH4	642.5891	585.694	856301.2085	218727.3728	268707.6004
DG(15:0/2	DG	DG+NH4	644.5116	444.623	145654.4686	150745.7162	181854.7991
DG(17:1/2	DG	DG+NH4	644.5137	426.166	37626.264	33675.33206	56414.8605
DG(17:0/2	DG	DG+NH4	646.5281	465.9115	167825.1404	154532.895	351117.8549
PC(P-16:0/	PC(P)	PC(P)+H	648.4978	261.99	8823.811207	10222.45557	11211.18825
Cer(d18:1/	Cer	Cer+H	648.617	560.654	6489204.637	5516757.154	5489899.797
PC(2:0/24:	PC	PC+H	650.4624	262.035	3063.536784	2155.497559	3574.148763
DG(17:1/2	DG	DG+NH4	650.5581	512.96	49799.29575	39727.8405	57609.78516
Cer(d18:1/	Cer	Cer+H	650.6325	593.177	1050271.041	302348.4258	599407.68
Cer(d18:0/	Cer	Cer+H	650.6324	573.385	166265.6914	119560.623	156860.3736
DG(18:4/2	DG	DG+NH4	652.4804	355.636	91975.07295	116056.8435	184732.3384
MG(39:0/(	MG	MG+H	653.6368	592.986	3579.347368	5060.580276	6646.104292
DG(18:4/2	DG	DG+NH4	654.496	384.979	203294.6969	225175.7118	452801.6229
DG(18:1/1	DG	DG+NH4	654.5888	571.872	40251.76514	37685.42683	70915.99208
Cer(t14:0/2	PhytoCer	PhytoCer+	654.6466	545.268	9548.348294	19250.2032	25935.18031
DG(16:1/2	DG	DG+NH4	656.5134	420.024	2021953.254	2180886.471	4913844.499
DG(18:0/1	DG	DG+NH4	656.6184	564.94	8499.648571	6259.593846	11222.59542
DG(18:1/2	DG	DG+NH4	658.527	468.364	7597592.436	7279598.877	13203116.8
DG(18:2/2	DG	DG+NH4	658.5279	455.987	1615108.909	1633038.19	3637205.412
DG(16:0/2	DG	DG+NH4	660.5428	494.65	3581.624176	34684.60048	2183.258855
SM(d14:0/	SM	SM+H	661.5152	281.86	56541.19775	55628.74385	84700.43128
PC(P-22:0/	PC(P)	PC(P)+H	662.498	349.581	13521.48637	13869.58146	18869.27608
PC(P-16:0/	PC(P)	PC(P)+H	662.5177	281.8815	26474.5619	22651.24316	35920.40509
DG(18:1/2	DG	DG+NH4	662.5577	521.736	1903058.298	1259998.215	1715219.447
Cer(d19:1/	Cer	Cer+H	662.6296	576.07	91530.78905	67029.24837	107461.1449
PE(14:0/1(	PE	PE+H	664.4784	383.2925	28836.3864	29660.57588	46693.06226
PE(28:0/2:	PE	PE+H	664.4854	456.097	44573.86414	43073.7639	67740.47914

LPC(28:0/(	LPC	LPC+H	664.5152	358.728	144427.4586	138569.3393	217591.7275
Cer(t18:1/2	PhytoCer	PhytoCer+	664.6094	511.144	37001.63511	35621.0863	66699.23223
CE(18:3)	CE	CE+NH4	664.6161	640.6095	7260.669006	2999.705783	2678.924377
Cer(d18:1/	Cer	Cer+H	664.6454	607.6825	5924.184265	4057.211975	23815.16427
DG(18:1/2	DG	DG+NH4	666.5882	558.621	384180.4718	322564.71	640756.7549
CE(18:2)	CE	CE+NH4	666.6023	700.049	258804.8568	54268.01877	38390.78857
DG(17:2/2	DG	DG+NH4	668.5183	454.233	47676.01511	42613.8122	17029.68011
DG(16:0/2	DG	DG+NH4	668.6042	586.416	240691.6922	208621.0877	363094.3247
DG(17:2/2	DG	DG+NH4	670.5268	456.893	283886.1363	264630.9017	424590.6777
DG(17:1/2	DG	DG+NH4	670.5267	436.277	78424.84972	71406.41042	148631.7976
DG(17:1/2	DG	DG+NH4	672.5413	470.2085	3814.072528	39686.54074	78426.07315
PC(2:0/26:	PC	PC+H	674.4641	311.684	12996.14337	13879.3187	20598.396
DG(17:0/2	DG	DG+NH4	674.5605	586.416	55908.70015	21369.76104	24243.54273
DG(17:1/2	DG	DG+NH4	674.5576	504.761	100159.9843	84926.9165	75149.3602
PE(P-16:0/	PE(P)	PE(P)+H	676.5121	458.5945	146383.9454	149593.1048	242982.168
DG(22:4/1	DG	DG+NH4	676.5783	580.0955	7801.819036	13896.70394	14139.43156
Cer(d18:1/	Cer	Cer+H	676.645	591.475	3600.539871	3260.347595	13584.04495
SM(d14:0/	SM	SM+H	677.545	334.686	6428.039398	1607.571084	20802.2858
PE(29:0/2:	PE	PE+H	678.4979	490.3035	17693.47223	13791.14651	19939.59875
PC(2:0/26:	PC	PC+H	678.4944	321.1715	377672.5528	325186.6653	670189.338
DG(20:5/2	DG	DG+NH4	678.5202	458.224	20009.83727	18900.37354	29123.13783
LPC(29:0/(	LPC	LPC+H	678.5297	386.812	71804.81526	70234.15919	103544.4821
DG(22:2/1	DG	DG+NH4	678.587	549.498	29721.56873	16702.12043	35758.56465
Cer(t17:1/2	PhytoCer	PhytoCer+	678.6453	591.8835	2684.689412	2969.012113	5486.43631
Cer(d18:1/	Cer	Cer+H	678.6608	621.195	66193.44164	36527.18938	74408
DG(18:3/2	DG	DG+NH4	680.5099	405.228	397321.7251	393224.4692	750523.7
DG(20:4/2	DG	DG+NH4	680.5137	503.8455	56820.427	59099.06764	64533.14846
DG(19:0/2	DG	DG+NH4	680.6025	578.4925	25489.82072	21756.34854	43708.97365
DG(18:4/2	DG	DG+NH4	682.5256	438.532	3062398.777	3055861.425	5737899.877
Cer(t17:0/2	PhytoCer	PhytoCer+	682.6555	591.9335	8372.076162	7399.601349	9386.928584
SM(d15:2/	SM	SM+H	683.4984	280.97	7596.508097	7082.319481	10529.14059
DG(20:5/2	DG	DG+NH4	684.5418	472.739	6120.481314	8597.358652	8957.172664
DG(18:2/2	DG	DG+NH4	684.5421	454.307	17472.90922	91863.79558	112606.1395
DG(20:4/2	DG	DG+NH4	686.5584	509.326	8801175.7	6535313.954	9837966.989
DG(18:4/2	DG	DG+NH4	686.5584	487.648	452726.7482	690956.9525	1464606.826
PE(18:2/14	PE	PE+H	688.4751	347.3285	94515.11921	89694.58003	137235.3107
PE(P-16:0/	PE(P)	PE(P)+H	688.512	445.476	423819.5123	507411.2342	607820.7212
DG(18:4/2	DG	DG+NH4	688.5717	522.577	1031592.251	777980.988	1324583.849
DG(18:0/2	DG	DG+NH4	688.5832	592.9465	31414.27863	5288.516235	11152.46307
DG(20:1/2	DG	DG+NH4	688.5985	542.8115	16868.00317	182622.5566	15130.7948
CE(20:5)	CE	CE+NH4	688.6053	756.578	7311.458978	21363.71946	38732.7113
PE(18:1/14	PE	PE+H	690.4919	389.4565	172392.615	155705.2453	237286.1123
PE(26:1/6:	PE	PE+H	690.4983	471.9465	112187.7933	118963.9328	166106.5713
PC(P-22:0/	PC(P)	PC(P)+H	690.5271	405.184	102668.4008	110512.4468	137121.9041
PC(P-20:0/	PC(P)	PC(P)+H	690.5495	341.935	191355.1386	192782.7864	222517.1813
DG(22:1/1	DG	DG+NH4	690.5868	535.889	79891.33976	70565.31079	139285.5037
PC(3:0/26:	PC	PC+H	692.5081	349.914	164161.407	152722.2367	217295.0048
PE(2:0/30:	PE	PE+H	692.5146	487.728	38078.53995	30505.32968	51685.80907

LPC(30:0/(	LPC	LPC+H	692.544	415.6685	1441.949739	442.3460026	50428.10645
DG(22:1/1	DG	DG+NH4	692.6034	564.1415	130419.4596	117086.3501	246742.8611
DG(24:1/1	DG	DG+NH4	696.6333	613.951	137785.1876	118488.7908	150484.8516
Cer(t20:0/2	PhytoCer	PhytoCer+	696.6703	605.49	7138.752631	7934.991718	10950.34443
PE(18:4/1:	PE	PE+H	698.4929	390.9715	60230.06875	56897.65468	66096.04692
DG(24:0/1	DG	DG+NH4	698.6505	640.705	39823.6442	21664.33988	24807.8949
SM(d14:2/	SM	SM+H	699.5263	294.0805	25223.16102	25756.08412	33331.07655
PC(10:0/2(	PC	PC+H	700.4748	254.42	14121.2029	12817.18555	23606.75948
PC(O-16:2	PC(O)	PC(O)+H	700.5356	309.864	24781.779	28212.36	97552.14664
GlcCer(d1	GlcCer	GlcCer+H	700.5564	385.305	49769.16399	37445.24525	61756.62148
DG(17:2/2	DG	DG+NH4	700.5706	527.893	122018.8121	131028.0422	171487.1229
PC(14:1/1(	PC	PC+H	702.4914	288.9075	107139.8808	93884.88007	179475.4589
PC(4:0/26:	PC	PC+H	702.4917	376.249	29178.60476	28912.72589	34693.36681
PE(P-16:0/	PE(P)	PE(P)+H	702.5282	462.991	3977424.562	3535627.039	4551883.092
DG(19:0/2	DG	DG+NH4	702.5883	614.008	12602.15087	4120.303101	4603.486176
PC(4:0/26:	PC	PC+H	704.5079	329.8905	957986.0375	779483.2937	1443674.245
PE(16:0/1	PE	PE+H	704.5079	420.049	224077.7095	234965.8444	358890.2194
PE(P-18:0/	PE(P)	PE(P)+H	704.5441	500.9575	2908.655337	2212.736718	2956.694613
PC(2:0/28:	PC	PC+H	706.5235	414.768	439292.9955	442333.6581	1006931.12
LPC(31:0/(	LPC	LPC+H	706.5579	441.138	225581.3266	215006.8848	297951.7896
PE(20:5/1	PE	PE+H	710.4606	302	37473.93096	28308.07551	59779.33651
TG(12:0/1	TG	TG+NH4	710.6146	666.679	4034.153992	0	7774.599784
PC(7:0/24:	PC	PC+H	712.5074	308.1	60574.25196	57099.76225	94349.80779
TG(12:0/1	TG	TG+NH4	712.6295	629.373	12824.02039	11499.01539	13066.63073
PC(11:0/2(	PC	PC+H	714.4916	362.268	84069.14426	78131.29483	114155.616
PC(O-18:2	PC(O)	PC(O)+H	714.5321	345.929	325750.7147	305276.5774	463949.7857
DG(18:2/2	DG	DG+NH4	714.5866	545.208	193002.427	185784.7029	321721.9269
CE(22:6)	CE	CE+NH4	714.6017	676.3705	1028880.677	605231.9718	565364.2416
PE(22:2/1	PE	PE+H	716.5066	400.8035	1278026.901	1329055.456	1706259.04
PC(O-22:2	PC(O)	PC(O)+H	716.5433	378.896	1294.885803	4606.552	6672.184904
DG(24:1/1	DG	DG+NH4	716.6342	575.114	4088.54895	2470.092	4455.914808
SM(d14:1/	SM	SM+H	717.5727	397.311	239082.8109	209105.9418	298995.8923
PE(24:1/1(	PE	PE+H	718.5203	438.522	1617052.213	1630778.177	2264381.577
PE(26:1/8:	PE	PE+H	718.5275	510.27	63710.02614	3204.08	52243.69402
PC(5:0/26:	PC	PC+H	718.5554	420.958	2907204.097	2572741.166	3411353.694
PC(P-16:0/	PC(P)	PC(P)+H	718.5762	397.764	117966.3934	101933.4318	148953.5721
PE(32:0/2:	PE	PE+H	720.5433	545.846	3830.753418	1785.009257	3610.541397
PC(15:0/1(	PC	PC+H	720.5559	348.1915	221332.4364	199212.2777	244618.9841
PC(2:0/29:	PC	PC+H	720.5594	421.826	1861250.586	405890.8308	550357.2123
LPC(32:0/(	LPC	LPC+H	720.5732	468.596	2131100.25	1977226.489	3423137.863
DG(18:1/2	DG	DG+NH4	722.6523	616.077	66340.191	67349.67136	72677.11518
PE(P-18:0/	PE(P)	PE(P)+Na	724.5118	463.239	52055.84953	58727.96626	47254.31721
SM(d14:0/	SM	SM+Na	725.5395	368.437	16238.20089	12684.42415	14479.74097
PC(14:1/1	PC	PC+H	726.4926	278.695	262390.822	268311.0741	454287.4381
PE(O-18:2	PE(O)	PE(O)+H	726.5254	431.504	438820.5027	90545.14	520407.1374
PC(O-16:2	PC(O)	PC(O)+H	726.5397	368.233	14018.22961	7524.57029	14225.082
PC(15:1/1	PC	PC+H	728.5082	301.9655	1083004.069	930420.6624	1728721.504
PC(O-18:2	PC(O)	PC(O)+H	728.552	367.571	208402.9179	214960.0497	278100.8901

CerP(d18:[illegible]	CerP	CerP+H	728.5839	509.343	64433.51892	47122.01028	52120.13934
PC(6:0/26:	PC	PC+H	730.5249	414.7955	1541123.597	1543217.931	3590453.851
PE(22:2/1[illegible]	PE	PE+H	730.5482	471.827	263700.6157	319128.4172	412833.751
SM(d14:0/	SM	SM+H	731.5893	425.3515	1469468.762	781884.5424	1114710.136
PC(6:0/26:	PC	PC+H	732.537	385.012	3791.584656	3716396.038	2398926.316
PE(24:1/1[illegible]	PE	PE+H	732.5637	504.7755	472607.9815	537632.701	814826.8985
PC(P-22:0/	PC(P)	PC(P)+H	732.5728	447.2295	1034191.589	933844.9513	1119055.332
PC(11:0/2[illegible]	PC	PC+H	736.4995	378.806	36704.30461	33105.15	43497.28406
PS(30:0/2:	PS	PS+H	736.5098	409.285	125188.203	132583.0776	163784.27
PE(22:4/1[illegible]	PE	PE+H	738.4902	358.939	1932780.308	1645346.931	2475545.152
PC(15:1/1[illegible]	PC	PC+H	738.4898	266.916	42806.91008	37314.92492	68908.03415
SM(d19:2/	SM	SM+H	739.5661	429.7555	18969.17412	16031.37757	18123.37781
PE(18:2/1[illegible]	PE	PE+H	740.5066	366.683	550040.4353	510451.2531	251678.796
PE(16:0/2[illegible]	PE	PE+H	740.5069	393.801	396677.1282	421243.2253	561391.7565
PE(20:3/1[illegible]	PE	PE+H	740.5322	330.789	160966.7651	198816.9679	328932.08
PE(24:4/1[illegible]	PE	PE+H	740.54	457.741	34060.51109	25612.9251	44897.97353
PC(13:0/2[illegible]	PC	PC+H	742.5278	331.635	470481.0885	511130.334	576254.6181
PE(18:0/1[illegible]	PE	PE+H	742.555	468.5815	69260.96924	70179.16944	83084.02205
PC(7:0/26:	PC	PC+H	744.5363	368.353	656311.2483	542333.9173	1533456.667
PE(26:2/1[illegible]	PE	PE+H	744.536	448.9595	153591.1097	1061749.299	1209.460876
PC(11:0/2[illegible]	PC	PC+H	744.5723	430.6195	4492575.685	5384459.444	4765174.9
PE(P-16:0/	PE(P)	PE(P)+H	748.5106	404.247	20416659.94	18779461.54	21853061.92
PC(2:0/31:	PC	PC+H	748.6008	515.586	252634.8104	186852.8861	264808.5658
PC(14:1/2[illegible]	PC	PC+H	750.49	242.3865	169304.096	182142.7556	256225.1695
PC(12:0/2[illegible]	PC	PC+H	750.489	302.6595	58890.3003	55442.40491	81994.13488
TG(13:0/1[illegible]	TG	TG+NH4	750.644	629.944	7804.528667	17561.50769	44467.18671
PC(12:0/2[illegible]	PC	PC+H	752.5066	294.114	3541042.261	3114666.245	6868112.254
PC(14:1/2[illegible]	PC	PC+H	752.5054	277.584	834858.1374	754608.8398	1271959.183
PE(22:4/1[illegible]	PE	PE+H	752.5092	385.899	229206.3552	199447.612	288653.3307
DG(18:1/2	DG	DG+NH4	752.7257	655.4555	5741.045369	6675.325792	3595.68
PC(10:0/2[illegible]	PC	PC+H	754.5251	329.466	8031397.42	7569039.189	10044562.57
PC(14:0/2[illegible]	PC	PC+H	756.5368	357.795	1334205.276	10116088.66	3222126.365
PC(20:3/1[illegible]	PC	PC+H	756.5352	428.023	130297.4595	110312.1718	132460.2791
PE(20:3/1[illegible]	PE	PE+H	756.5637	508.448	215796.4771	232427.0273	362185.3575
PE(P-18:0/	PE(P)	PE(P)+H	756.5719	508.436	209473.1812	225050.9172	353706.4995
DG(23:0/2	DG	DG+NH4	756.6322	593.116	11004.84554	11000.15768	25195.30588
SM(d18:2/	SM	SM+H	757.6304	509.768	77973.65744	78033.87585	122513.9239
PC(8:0/26:	PC	PC+H	758.5828	460.412	482611.3319	502104.3995	535947.2146
PE(20:4/1[illegible]	PE	PE+Na	760.4719	285.4385	76615.50793	64924.84668	114394.0323
PS(20:2/1[illegible]	PS	PS+H	760.5003	384.533	48087.53407	49832.2706	64804.27336
PE(22:6/1[illegible]	PE	PE+H	762.4902	324.516	1425418.272	1248761.256	1841265.22
PC(2:0/32:	PC	PC+H	762.6027	548.111	29087.28241	30146.47957	53620.43186
PE(22:5/1[illegible]	PE	PE+H	764.5081	380.619	8355188.625	8018282.883	11620821.37
PC(13:0/2[illegible]	PC	PC+H	764.5394	352.5445	523.8626709	508.0782623	156527.8676
PE(22:6/1[illegible]	PE	PE+H	764.5375	444.595	16320.58876	16386.36447	62154.82521
TG(12:0/1[illegible]	TG	TG+NH4	764.658	635.459	75849.24768	132279.0867	175716.2623
PC(13:0/2[illegible]	PC	PC+H	766.5192	323.619	1516299.073	1474726.575	2163261.251
PE(20:1/1[illegible]	PE	PE+Na	766.5179	410.3585	5991585.186	5675873.575	7901582.681

PC(15:1/2(	PC	PC+H	766.5192	295.861	120023.854	100240.0504	123654.9512
PE(24:4/1<	PE	PE+H	766.5286	410.779	6249211.117	5797708.063	7943795.147
PC(11:0/2<	PC	PC+H	768.5346	344.6725	322482.9103	302636.1766	695303.2246
PE(24:4/1<	PE	PE+H	768.5346	441.993	734708.8829	907287.618	1525024.843
DG(20:5/2	DG	DG+NH4	768.6356	616.567	4420.420532	6312.609791	5789.662063
PE(O-16:2	PE(O)	PE(O)+Na	770.4961	405.161	66110.91169	58932.53333	62399.22706
PC(15:0/2(	PC	PC+H	770.5527	383.245	5287724.159	4708488.979	4924201.8
PE(22:2/1(	PE	PE+H	770.5821	468.0375	973483.9556	1172270.664	1484972.151
DG(24:1/2	DG	DG+NH4	770.6485	587.192	20279.445	18912.285	28945.45027
PC(9:0/26:	PC	PC+H	772.5734	420.967	16000947.96	14916491.92	18257225.71
PC(13:0/2:	PC	PC+H	772.6007	511.1395	869638.4974	1017491.934	1257783.024
TG(12:0/2(	TG	TG+NH4	772.6567	606.873	10850.31213	10669.74978	17658.69568
PC(16:1/1{	PC	PC+Na	774.4859	278.454	74483.04326	66863.52765	83277.01696
PE(O-18:2	PE(O)	PE(O)+H	774.527	409.515	3497816.713	4566284.355	5182305.351
PC(9:0/26:	PC	PC+H	774.6167	520.0565	3757292.398	4783975.826	6033993.151
PC(P-20:0/	PC(P)	PC(P)+H	774.6546	607.1845	12179.71076	6085.932129	40839.63458
PC(18:3/1{	PC	PC+H	776.5074	261.7	581001.7285	469475.7666	928464.5483
PE(P-18:0/	PE(P)	PE(P)+H	776.5399	450.798	3468.584787	1348.747603	2314.782192
PC(14:1/2:	PC	PC+H	776.5395	342.265	3163.51593	2770.830892	3655.580345
PC(18:0/1'	PC	PC+H	776.5976	436.277	558098.2609	606984.6817	674434.4608
PC(2:0/33:	PC	PC+H	776.6211	520.854	525616.0579	625556.0074	789549.1613
PE(17:1/2(	PE	PE+Na	778.5185	405.4245	643855.8695	653348.3044	848426.4586
PC(14:0/2:	PC	PC+H	778.5284	302	11883153.55	21424286.04	18183361.58
TG(14:1/1(	TG	TG+NH4	778.6818	656.848	40216.82	76937.33531	177619.1184
PC(14:0/2:	PC	PC+H	780.5641	413.693	359506.5007	355757.098	482275.9179
PE(24:4/1:	PE	PE+H	780.568	465.7935	266014.8538	535265.0363	414224.12
PC(12:0/2<	PC	PC+H	782.5524	386.798	12954913.44	15815586.66	16939250.11
PC(26:1/8:	PC	PC+Na	782.5836	511.123	135755.0474	128521.6467	64507.9716
PC(O-20:2	PC(O)	PC(O)+H	782.5953	425.354	292018.6076	273754.3728	273776.8482
TG(15:0/1	TG	TG+NH4	782.7398	732.632	2787.430691	2472.400289	1941.489041
PE(22:6/1{	PE	PE+H	784.4731	274.809	181806.1913	156915.8093	271687.077
PC(O-22:2	PC(O)	PC(O)+H	784.6356	516.467	97691.96052	81837.36342	86372.07755
SM(d14:1/	SM	SM+H	785.6359	476.2555	6190561.019	6858246.072	10139131.67
PE(22:5/1{	PE	PE+H	786.4898	308.109	495252.3569	450553.9776	719998.9026
PC(O-20:2	PC(O)	PC(O)+H	786.6301	603.521	42477.42246	51894.62048	202298.451
PE(22:4/1{	PE	PE+H	788.5078	345.5545	11938216.35	10024157.32	14078796.04
PC(P-22:0/	PC(P)	PC(P)+H	788.6375	603.114	33686.3668	37960.57682	22801.45636
PC(P-18:0/	PC(P)	PC(P)+H	788.6505	626.7395	159778.06	450178.1557	746601.4226
TG(14:0/1(	TG	TG+NH4	788.6585	626.5815	159371.3196	531756.362	709360.0419
PE(20:2/1{	PE	PE+Na	790.518	385.58	9083577.378	7827550.35	10417780.37
PC(15:1/2:	PC	PC+H	790.5188	295.868	529176.6296	508866.5816	690696.4315
PE(22:4/1{	PE	PE+H	790.5261	385.899	9044482.186	8144132.693	10478418.77
LacCer(d1	LacCer	LacCer+H	790.541	236.736	600.2707626	565.845988	4931.347667
PE(22:5/1{	PE	PE+H	790.5393	444.618	8190.147816	6374.693881	11214.85321
PS(21:0/1:	PS	PS+H	790.5456	471.8805	3198.283296	1946.386408	1459.039953
PC(O-18:2	PC(O)	PC(O)+H	790.5548	471.8595	451620.6441	524339.8074	569555.1591
SM(d17:2/	SM	SM+H	791.6045	488.9755	2317037.481	2603437.273	2726104.154
PE(22:2/1{	PE	PE+H	792.5359	428.009	3097.790871	3210748.194	6214397.225

PC(15:0/2:	PC	PC+H	792.5635	390.11	2177996.509	2487762.864	2886472.631
PC(O-20:2	PC(O)	PC(O)+H	792.6073	488.9755	453684.2287	500070.3198	540024.4373
SM(d17:1/	SM	SM+H	793.6159	488.9335	108677.5714	113442.5121	124421.5084
PC(17:0/2(	PC	PC+H	794.5535	458.754	1355126.794	1674804.689	1974814.217
PC(17:1/2(	PC	PC+H	794.5516	333.3655	499180.5643	444404.1992	586225.1329
PE(24:4/1(	PE	PE+H	794.5563	443.729	907560.5114	1496589.787	1126391.013
PC(15:0/2:	PC	PC+H	794.5821	424.482	6607487.395	6284103.738	6889051.718
PE(26:1/1:	PE	PE+Na	796.5644	481.874	175989.2123	195790.3515	248696.3792
PC(13:0/2	PC	PC+H	796.5693	413.891	694332.4808	786763.25	819318.0676
PE(24:4/1(	PE	PE+H	796.6037	471.288	540463.3204	558135.2167	796353.319
PC(P-16:0/	PC(P)	PC(P)+Na	796.6164	612.1215	352276.5467	534584.9444	394492.4889
PC(P-20:0/	PC(P)	PC(P)+H	796.637	497.4585	35281.1589	27890.15809	49927.07367
TG(14:0/1	TG	TG+NH4	796.7219	696.509	1880284.681	1552732.304	2549522.864
PC(22:6/1	PC	PC+Na	798.4917	295.02	32768.57092	31980.81491	42491.22923
PC(P-20:0/	PC(P)	PC(P)+H	798.6184	612.257	23311.10385	39465.50983	35243.81084
PC(O-22:2	PC(O)	PC(O)+H	798.6332	490.4085	566484.852	136810.1129	795004.2131
GlcCer(d1	GlcCer	GlcCer+H	798.6649	534.329	65701.6	64487.36008	132470.4409
PC(11:0/2(	PC	PC+H	800.5972	470.346	2900449.155	3100122.606	3503000.008
PC(P-22:0/	PC(P)	PC(P)+H	800.6518	621.144	42727.61775	123065.8111	197578.2329
TG(15:1/1	TG	TG+NH4	800.661	676.43	22549.40255	12753.85978	9728.19003
PC(22:4/1	PC	PC+Na	802.5177	350.794	366346.6972	358721.6679	324852.2277
PC(18:4/2(	PC	PC+H	802.5274	275.741	8639825.127	6720247.938	15645485.71
PC(18:3/2(	PC	PC+H	802.5536	384.772	58328.82344	130042.978	99649.07157
PC(11:0/2(	PC	PC+H	802.6171	507.4995	1460103.886	1556826.047	1760091.31
TG(13:0/1	TG	TG+NH4	802.6771	696.2705	31418.47882	15800.11655	17184.23
PC(16:1/2:	PC	PC+H	804.5664	392.5115	142102.5732	153354.7144	156781.9462
PE(24:4/1:	PE	PE+Na	804.5681	493.015	267838.5811	366625.7021	384360.226
PC(18:0/1	PC	PC+H	804.6334	471.353	80318.25333	163048.112	93231.84447
PC(16:0/2:	PC	PC+H	806.5836	427.1115	1683714.928	1825456.724	1618645.746
TG(14:0/1	TG	TG+NH4	806.705	669.225	277460.6701	454772.4424	590212.7488
PC(16:1/2:	PC	PC+H	808.5651	356.1185	6775947.413	6865743.83	7825835.945
TG(15:0/1	TG	TG+NH4	808.7474	733.516	11599.88629	8095.497116	7603.908403
PE(22:6/2(	PE	PE+H	810.49	298.4325	1616163.858	1834019.085	2265179.791
PC(18:1/2(	PC	PC+H	810.5817	412.167	17049220.5	15292066.41	18437406.41
PC(14:0/2	PC	PC+H	810.5812	438.5015	3345584.042	3365146.622	3354677.368
PC(O-22:2	PC(O)	PC(O)+H	810.6561	515.587	582727.3983	654550.8447	923411.2689
TG(15:0/1	TG	TG+NH4	810.7611	760.278	21490.87324	7747.554368	6464.291607
SM(d16:2/	SM	SM+H	811.654	513.834	999229.038	1084802.282	1575499.387
PC(18:1/2(	PC	PC+H	812.5977	449.904	13975874.62	13492676.76	14718547.94
PC(P-22:0/	PC(P)	PC(P)+H	812.6513	513.82	494342.0354	511765.7757	722759.9561
PE(22:4/2(	PE	PE+H	814.5555	361.694	418576.6148	509639.8848	490250.4203
PC(O-20:2	PC(O)	PC(O)+H	814.6679	627.663	456818.4888	1243684.875	548801.6292
PC(P-22:0/	PC(P)	PC(P)+H	814.6655	551.699	933550.5139	965572.5668	1355738.319
PC(17:2/2:	PC	PC+H	816.5656	400.7975	603745.1489	852759.0443	805935.7949
PC(22:5/1	PC	PC+H	818.5544	333.3655	1196027.729	1141193.596	1349159.281
PC(17:1/2:	PC	PC+H	818.5839	411.265	5299956.948	8470551.155	7603193.361
PC(2:0/36:	PC	PC+H	818.6453	570.507	384129.4653	314294.9903	457177.8181
PC(5:0/33:	PC	PC+H	818.6735	516.465	27068.74473	21899.6001	25372.16669

TG(16:1/1	TG	TG+NH4	818.7051	660.411	2197344.327	4478566.807	6236802.534
SM(d19:2/	SM	SM+H	819.6311	492.957	137740.4263	130351.3477	135369.076
PC(17:0/2	PC	PC+H	820.6008	457.83	6362297.839	9132980.329	8626668.193
PC(O-22:2	PC(O)	PC(O)+H	820.6361	534.133	14608.01508	12332.45278	18060.32068
GlcCer(d1	GlcCer	GlcCer+H	820.6476	570.465	58384.97763	50955.30454	78208.1424
TG(14:0/1	TG	TG+NH4	820.7213	679.5635	7356668.203	11152840.19	16759301.78
PC(20:5/1	PC	PC+Na	822.4886	243.347	46170.26842	47540.94438	82750.80015
PC(15:1/2	PC	PC+H	822.6102	457.829	864059.4689	1151455.039	1281585.202
PC(17:0/2	PC	PC+H	822.6095	489.46	225133.8918	276433.3268	308596.9787
PC(19:0/2	PC	PC+H	822.6139	505.702	64040.8643	69483.54525	74999.73213
PC(P-22:0	PC(P)	PC(P)+H	822.6531	527.915	60081.0719	68332.60048	110519.3505
PC(15:0/2	PC	PC+H	824.6283	472.8215	70793.53142	86824.86451	98041.03624
PC(P-22:0	PC(P)	PC(P)+H	824.6513	602.799	36279.32979	105818.3114	198146.071
TG(13:0/1	TG	TG+NH4	824.6569	660.415	39444.73145	45639.79349	36538.15229
TG(16:0/1	TG	TG+NH4	824.7506	718.5125	3938490.016	3556361.051	6835560.939
PC(P-22:0	PC(P)	PC(P)+H	826.6583	622.005	110678.0821	339771.9102	526429.0423
TG(16:1/1	TG	TG+NH4	826.6753	679.558	93861.08816	78416.97143	71909.13382
PC(O-22:2	PC(O)	PC(O)+H	826.6695	535.048	362099.1623	435405.8749	649948.4402
PC(O-22:2	PC(O)	PC(O)+H	828.6685	541.3135	315360.2559	268997.8562	509737.5195
TG(17:1/1	TG	TG+NH4	828.6882	642.714	240759.912	638314.4607	824716.198
TG(15:0/1	TG	TG+NH4	828.6935	663.348	51721.85745	108059.5801	121393.2061
LacCer(d1	LacCer	LacCer+H	830.5682	233.214	156630.8438	97689.74388	76048.35207
PC(P-22:0	PC(P)	PC(P)+H	830.6953	657.6245	317528.0129	673364.1542	915250.815
TG(15:1/1	TG	TG+NH4	830.7039	682.253	14702.67333	64025.95304	75745.0279
TG(14:0/1	TG	TG+NH4	832.7204	699.908	48512.8271	59982.36033	81078.35009
SM(d20:2/	SM	SM+H	833.6403	513.828	72197.66806	41109.52475	37302.84037
PS(20:3/2	PS	PS+H	834.5266	294.134	17644.71873	41734.56507	32875.36296
PC(O-22:2	PC(O)	PC(O)+Na	834.6552	550.788	402417.1399	398709.6881	415058.1284
TG(17:1/1	TG	TG+NH4	834.7602	735.311	33275.29724	24728.60739	24821.76045
SM(d20:1/	SM	SM+H	835.6538	550.816	295070.6291	302239.0185	282830.2804
SM(d15:2/	SM	SM+Na	835.6479	517.353	2764417.354	2830757.906	2332833.018
PC(16:1/2	PC	PC+H	836.5994	456.077	2967481.247	3207086.031	3130717.181
PC(O-16:2	PC(O)	PC(O)+Na	836.644	589.1025	5920.113566	3441.869832	4882.338691
TG(15:0/1	TG	TG+NH4	836.7678	760.314	78545.58891	26638.87071	24388.08896
SM(d15:1/	SM	SM+Na	837.6642	565.197	388509.3116	143610.7378	257337.4809
SM(d20:0/	SM	SM+H	837.7046	606.038	20450.37851	60058.08679	134519.6704
PE(22:5/2	PE	PE+H	838.5186	339.526	616891.8164	679297.8806	931454.9755
PS(24:4/1	PS	PS+H	838.5697	387.65	128873.8574	430975.946	334240.8314
PC(21:0/1	PC	PC+Na	838.6094	471.9155	608625.7965	610676.5989	629702.1105
PC(16:0/2	PC	PC+H	838.6112	527.8955	231220.022	220337.5509	187558.228
TG(16:0/1	TG	TG+NH4	838.7955	793.631	30235.64529	9649.887858	9456.028163
PC(14:1/2	PC	PC+H	840.6271	495.67	1751967.486	1655736.212	1848317.074
TG(14:0/2	TG	TG+NH4	840.7141	701.658	10178.95924	14038.3374	14769.3051
PS(22:0/1	PS	PS+H	842.5816	458.2375	107722.9707	138794.9749	105615.0406
PS(26:2/1	PS	PS+H	842.5908	425.33	29529.78343	77936.18273	60020.16243
TG(14:0/1	TG	TG+NH4	842.7028	710.681	53906.22007	42537.38079	54318.6674
TG(14:0/2	TG	TG+NH4	842.7052	656.682	4010497.766	9426348.471	11423461.13
PC(O-20:2	PC(O)	PC(O)+H	844.6367	529.703	8254.2085	5940.275116	8483.486576

PC(O-22:2	PC(O)	PC(O)+H	846.6419	633.836	93836.48267	99515.13966	92864.79288
PC(2:0/38:	PC	PC+H	846.7002	645.747	48550.63412	92337.31755	136167.1283
PC(17:2/2	PC	PC+H	848.6272	504.7805	134278.9287	237155.5288	238142.8077
PC(P-22:0	PC(P)	PC(P)+H	848.6513	593.943	32055.89687	107751.2028	232254.5541
TG(15:1/1	TG	TG+NH4	848.6582	656.899	73461.04037	97989.49865	109705.0278
TG(16:0/1	TG	TG+NH4	848.7506	699.7415	37634103.72	40593815.57	63439844.93
PC(22:5/1	PC	PC+Na	850.5203	301.974	652670.7458	652986.7136	633115.0045
PC(17:1/2	PC	PC+H	850.6227	550.981	34321.4624	41668.05183	42781.27619
PC(P-20:0	PC(P)	PC(P)+H	850.6641	638.8905	57663.902	146139.2561	217778.083
TG(18:4/1	TG	TG+NH4	850.676	639.165	56859.33785	142988.2754	212103.3237
TG(18:3/1	TG	TG+NH4	852.687	626.5355	205600.4365	643152.2284	1034770.346
PC(20:4/2	PC	PC+H	854.586	417.432	42444.82093	71335.79544	78808.5152
TG(14:0/2	TG	TG+NH4	854.7041	699.4865	421750.4805	296067.0039	252895.7786
SM(d18:2/	SM	SM+H	855.7169	614.4	63657.71193	95403.47037	241044.6115
PC(20:3/2	PC	PC+H	856.5986	441.119	33282.29285	156400.6341	113377.1429
TG(18:2/1	TG	TG+NH4	856.7216	719.358	52522.42296	319997.9335	272176.608
TG(15:1/1	TG	TG+NH4	856.7416	709.671	527175.054	23680.89481	29886.92683
PC(20:4/2	PC	PC+H	858.5801	386.7785	4846041.702	4552270.838	4411215.289
PS(24:1/1	PS	PS+H	858.6298	557.4705	15357.10234	50974.13931	67974.58421
PC(14:1/2	PC	PC+H	858.7033	641.473	41788.79055	79790.58739	90227.93506
PC(18:1/2	PC	PC+H	858.7081	657.335	213820.4602	422692.4109	570373.526
PC(2:0/39:	PC	PC+H	860.7002	602.276	66357.48702	209435.0882	558028.9381
TG(17:1/1	TG	TG+NH4	860.7661	737.177	10577.8472	26263.68866	28068.02542
TG(17:2/1	TG	TG+NH4	860.7781	737.05	38865.86782	25062.1349	26146.73866
SM(d22:2/	SM	SM+H	861.665	549.1075	11613.91132	11522.27539	14111.67398
TG(16:1/1	TG	TG+NH4	862.6764	665.721	36523.4505	52997.46854	52054.29008
TG(15:1/1	TG	TG+NH4	862.7895	763.5915	88325.38139	38434.9952	37167.90844
PI(15:0/20	PI	PI+NH4	864.5423	304.578	2831.574829	6044.363604	7407.854976
PI(17:1/18	PI	PI+NH4	864.5422	265.18	222681.3251	210944.9503	269168.3024
TG(16:0/1	TG	TG+NH4	864.7277	638.7925	128169.2598	348630.8821	479751.615
TG(17:0/1	TG	TG+NH4	864.805	794.085	66784.63173	22453.84898	20757.2999
TG(18:3/1	TG	TG+NH4	866.7414	658.5665	94107.31078	231071.113	306097.9458
PC(16:1/2	PC	PC+H	868.6946	515.5865	22210.21734	22010.09123	33294.42111
TG(16:0/1	TG	TG+NH4	868.7515	622.9255	40182.24941	85013.66973	131385.6785
SM(d21:2/	SM	SM+Na	869.6356	619.69	28947.69446	15389.54366	54946.15803
PC(16:0/2	PC	PC+H	870.7122	550.381	18025.48938	16921.03189	30256.55804
PC(O-22:2	PC(O)	PC(O)+H	870.7231	550.871	23011.73645	27194.40582	51516.29928
TG(16:1/1	TG	TG+NH4	870.7338	666.594	14467420.51	29657943.47	42091952.53
PC(O-22:2	PC(O)	PC(O)+H	872.6468	639.7295	166688.2825	206601.0887	200712.8192
PC(P-22:0	PC(P)	PC(P)+H	874.6586	601.568	47933.12483	141554.5133	258166.7856
PC(3:0/39:	PC	PC+H	874.7097	586.127	9616.130829	24033.40714	48426.31531
PC(14:0/2	PC	PC+H	874.7294	665.648	66255.42974	106845.6265	65590.57557
PC(21:0/2	PC	PC+H	876.6286	546.7015	39473.35513	21506.35909	42644.56
TG(17:1/1	TG	TG+NH4	876.6869	666.329	184961.1188	205087.74	145261.254
PC(P-22:0	PC(P)	PC(P)+H	876.6863	616.676	160758.3706	548245.5903	950090.0116
PC(21:0/2	PC	PC+H	878.6426	521.76	70206.82928	86415.51686	138351.9288
TG(17:1/1	TG	TG+NH4	878.7066	683.589	433339.3045	383180.0601	363228.2325
PC(22:5/2	PC	PC+H	880.6052	448.94	29456.26808	84096.72546	71155.74918

TG(15:0/2 TG	TG+NH4	880.7361	693.732	591420.5514	0	21765.12622
PC(17:1/2 PC	PC+H	882.6809	558.072	79193.4364	226965.0631	525230.515
PC(P-22:0, PC(P)	PC(P)+H	882.7369	586.923	7618.737686	21814.10767	51809.0918
SM(d20:2/ SM	SM+H	883.7424	585.879	10907.39099	17586.6215	39708.05859
PC(20:5/2 PC	PC+H	884.6365	545.282	3948.148262	1932.74498	2738.470025
TG(18:4/1 TG	TG+NH4	884.656	633.69	14719.80083	32083.00209	39231.52419
PC(17:0/2 PC	PC+H	884.6978	557.074	44102.55569	134040.383	308081.9153
PC(20:2/2 PC	PC+H	884.7261	657.6195	74714.57322	244591.6484	353707.5287
TG(17:2/1 TG	TG+NH4	884.752	704.395	131656.9546	217498.3058	371378.7027
TG(14:1/2 TG	TG+NH4	886.6727	650.68	32434.04052	75479.97788	70598.96707
PC(14:1/2 PC	PC+H	886.7206	607.084	165149.9841	548886.8079	1356949.596
PC(20:1/2 PC	PC+H	886.745	662.9965	5336.939348	270129.2173	269897.8509
TG(17:1/1 TG	TG+NH4	886.7659	723.94	200719.0032	337929.777	478065.4008
PI(17:1/20 PI	PI+NH4	888.5427	266.2885	15329.21397	10389.78398	13761.74244
TG(18:4/1 TG	TG+NH4	888.7281	626.5285	92007.62107	273821.5256	464520.0755
TG(17:0/1 TG	TG+NH4	888.7952	765.61	16707.67462	8336.561198	10513.91815
TG(18:3/1 TG	TG+NH4	890.7032	678.258	30771.08057	41221.30938	44810.95215
TG(17:0/1 TG	TG+NH4	890.8163	796.468	19573.39914	9841.087092	14104.83383
Sulfatide(ST	ST+H	892.6362	488.4925	5200.151001	5553.422913	18874.28503
TG(18:2/1 TG	TG+NH4	892.7562	659.924	200827.9485	345321.7859	434140.8308
TG(17:0/1 TG	TG+NH4	892.8411	831.4635	11176.04506	4194.702795	5445.2575
PC(18:2/2 PC	PC+H	894.6988	594.97	53642.96738	102581.9746	142097.1961
TG(18:2/1 TG	TG+NH4	894.7627	626.434	14180.51017	28106.72654	61072.86652
TG(18:2/1 TG	TG+NH4	896.7493	679.407	4163346.171	35751543	47078851.34
TG(15:1/2 TG	TG+NH4	900.6861	661.3145	419854.8782	557169.7242	408592.5275
PC(22:0/2 PC	PC+H	900.7414	667.8135	196504.3213	261389.8894	194409.6277
TG(15:0/2 TG	TG+NH4	902.703	679.765	266830.4754	304091.2882	251759.7229
PI(14:0/24 PI	PI+NH4	904.5985	396.431	6886.264537	30119.36709	25343.11554
TG(17:1/2 TG	TG+NH4	904.7219	667.747	136938.3646	257823.4128	346412.1547
PC(24:4/2 PC	PC+Na	906.5983	536.4255	10448.11096	10039.10795	6913.37176
PC(20:5/2 PC	PC+H	906.7006	621.117	68774.50948	182579.8337	294280.0624
TG(17:1/2 TG	TG+NH4	906.7509	696.872	410873.0364	399687.3059	367561.597
PC(24:4/2 PC	PC+Na	908.5988	586.661	17918.8444	36724.96176	32814.68982
PC(20:4/2 PC	PC+H	908.7043	566.163	88135.57861	268281.8246	683641.9102
PC(18:4/2 PC	PC+H	908.727	639.11	191810.4565	431797.2592	486366.274
TG(16:1/1 TG	TG+NH4	908.7634	716.7895	559041.123	545459.7063	533296.6524
PC(22:6/2 PC	PC+H	910.6523	557.284	36593.9632	117039.1633	250684.6954
PC(18:3/2 PC	PC+H	910.7065	552.947	39758.42004	102504.3431	232290.4084
TG(12:0/2 TG	TG+NH4	912.7257	634.3785	24130.82778	42877.96173	167904.8908
SM(d22:1/ SM	SM+H	913.7894	550.871	2545.737305	1875.626221	2151.367294
TG(14:0/2 TG	TG+NH4	914.7457	652.7975	93568.46857	118625.3457	191477.0171
TG(18:3/1 TG	TG+NH4	914.7954	808.822	37772.08521	54784.46488	73662.88319
TG(18:2/2 TG	TG+NH4	916.7521	648.553	117198.6202	271552.9665	336189.9694
TG(18:2/2 TG	TG+NH4	918.7323	644.965	18028977.96	54333022.97	84537916.06
PC(20:3/2 PC	PC+H	920.7198	625.806	134504.7605	339809.6917	616240.6482
TG(16:1/2 TG	TG+NH4	920.777	629.082	27722.17896	27805.84142	54153.32607
PC(18:4/2 PC	PC+H	922.7357	644.442	58502.27959	94255.08659	121393.2814
TG(18:1/1 TG	TG+NH4	924.7792	694.288	3394380.269	6790082.505	31510008.13

PI(20:3/20	PI	PI+NH4	926.5538	301.997	173510.0372	220139.3315	188071.928
TG(17:2/2(	TG	TG+NH4	926.702	669.151	340138.875	463503.6093	348281.8848
PI(18:0/22	PI	PI+NH4	928.5684	347.389	478137.3279	836013.1565	410161.3802
TG(17:0/2(	TG	TG+NH4	930.7329	671.4405	48447.32859	116136.2642	242103.9935
PC(22:6/2:	PC	PC+H	932.7019	560.2215	98640.52867	325274.8148	794714.4193
TG(18:3/1	TG	TG+NH4	932.7487	714.025	281821.7738	319642.5887	424387.8972
TG(18:3/2(	TG	TG+NH4	938.7384	619.957	109900.1131	143863.3588	127977.8988
PC(22:6/2(	PC	PC+H	942.6905	588.611	24036.23652	58326.84959	71091.9931
PC(22:5/2(	PC	PC+H	944.7194	619.41	117073.5371	312752.1966	582495.4494
PC(22:4/2(	PC	PC+H	946.7151	585.4365	24438.68652	70633.36584	220389.4563
TG(18:0/2:	TG	TG+NH4	952.8552	746.4995	49889.68048	116144.7388	166302.9941
TG(15:1/2:	TG	TG+NH4	956.7471	696.296	128972.1722	207622.3933	232201.3125
PC(22:6/2	PC	PC+H	960.724	592.965	6385.2165	57213.68113	305712.9493
TG(20:5/2(	TG	TG+NH4	962.7003	654.964	37422.98765	54878.37561	52230.57543
TG(18:1/2:	TG	TG+NH4	966.8255	808.0885	179894.1463	371730.5961	517398.8797
TG(18:2/2:	TG	TG+NH4	974.8317	702.872	34379.083	76745.0929	119881.1352
TG(18:1/2:	TG	TG+NH4	976.8454	723.885	25590.4931	79106.86925	156445.8511
TG(20:1/2(	TG	TG+NH4	978.861	746.8905	19095.14053	15217.19402	9826.387678
PC(18:3/3:	PC	PC+H	980.8044	650.271	47985.35982	118313.276	147686.75
PC(31:0/18	PC	PC+Na	986.769	675.253	104497.8232	216889.3175	313019.3632
TG(20:1/2:	TG	TG+NH4	1002.866	725.184	14736.75759	18533.52956	25381.62145
TG(19:0/2:	TG	TG+NH4	1012.856	766.514	19870.6893	43880.04816	67632.44021
TG(20:1/2:	TG	TG+NH4	1022.843	746.097	2347.166705	4451.955229	4638.597552
TG(20:0/2:	TG	TG+NH4	1026.873	779.414	17377.03702	36546.41673	66004.3709
TG(22:5/2:	TG	TG+NH4	1046.84	730.05	1015.395711	6995.682886	5551.215985
PE(39:0/18	PE	PE+Na	1056.845	674.409	34107.98627	38782.14355	35899.9914
TG(22:1/2:	TG	TG+NH4	1058.912	777.729	3840.762922	8273.489345	18903.9459
LPE(P-16:	LPE(P)	LPE(P)-H	436.2839	98.931	466323.7096	336207.3001	650567.2526
LPE(16:1/(	LPE	LPE-H	450.2626	71.3515	15336.35227	11171.80208	15749.88828
LPE(16:0/(	LPE	LPE-H	452.2792	87.41	213074.195	137890.3013	226645.174
LPE(O-18:	LPE(O)	LPE(O)-H	462.299	103.695	106890.2004	104107.1441	168683.3444
LPE(17:1/(	LPE	LPE-H	464.2779	78.769	24903.354	20994.17022	24677.61765
LPE(17:0/(	LPE	LPE-H	466.2936	101.0835	22055.49132	15236.24358	33100.83895
LPE(18:3/(	LPE	LPE-H	474.2626	65.34	11496.22256	9616.620067	13341.57465
LPE(18:2/(	LPE	LPE-H	476.2785	75.1615	239354.7214	204515.9082	261655.2019
LPE(18:1/(	LPE	LPE-H	478.2947	91.147	271022.9051	182426.657	310735.1226
LPE(18:0/(	LPE	LPE-H	480.3091	119.7095	536961.2904	331830.1144	575624.591
LPE(O-20:	LPE(O)	LPE(O)-H	490.3295	144.1585	5513.976	6691.766	9392.24325
LPE(19:0/(	LPE	LPE-H	494.3246	142.044	14521.77662	9538.1826	22129.38646
LPE(20:5/(	LPE	LPE-H	498.2624	62.971	86189.33824	78545.59906	122246.0474
LPE(20:4/(	LPE	LPE-H	500.2778	71.028	57926.253	38359.4895	58960.17537
LPE(20:3/(	LPE	LPE-H	502.293	81.625	9367.790167	6249.156882	10880.51067
LPE(20:2/(	LPE	LPE-H	504.3089	98.476	27628.816	20980.56	41771.18167
LPE(20:1/(	LPE	LPE-H	506.3245	124.5225	47754.45733	32070.2026	75905.57055
LPE(20:0/(	LPE	LPE-H	508.3396	121.0385	24093.08118	20330.27319	22524.2775
LPC(14:0/(	LPC	LPC+HCC	512.2989	66.907	32324.8065	31772.733	46383.09483
LPS(18:2/(	LPS	LPS-H	520.2645	87.417	5843.998111	4121.544778	5894.983111
PE(2:0/18:	PE	PE-H	522.3191	91.073	5417.089895	2053.056377	4817.237333

LPE(22:6/(	LPE	LPE-H	524.279	68.5355	736268.2218	705765.5897	809049.4015
LPC(O-16:	LPC(O)	LPC(O)+H	524.3351	99.348	35354.09511	35169.81918	36073.40224
LPE(22:5/(	LPE	LPE-H	526.2916	73.09	118352.8154	128104.6481	167490.4025
LPC(15:0/(	LPC	LPC+HCC	526.3205	50.859	1453.6916	1469.955636	1724.265214
LPC(16:1/(	LPC	LPC+HCC	538.3142	70.8615	84222.44879	72327.11647	98424.70958
LPC(16:0/(	LPC	LPC+HCC	540.3316	87.217	1337558.197	1140756.185	1334383.728
PE(6:0/16:	PE	PE-H	550.3502	87.345	7579.229833	7607.257765	8457.338625
LPC(17:1/(	LPC	LPC+HCC	552.3295	80.773	39908.814	36183.77357	41989.20844
SM(d18:0/	SM	SM+HCO(	553.3682	109.605	28861.65445	5617.437278	5300.474786
LPC(17:0/(	LPC	LPC+HCC	554.3454	101.086	29946.608	22728.41335	33201.33445
LPC(O-18:	LPC(O)	LPC(O)+H	554.3817	148.258	14102.9978	16957.89459	17979.71065
LPC(18:4/(	LPC	LPC+HCC	560.2982	58.8545	21683.04029	20972.871	26052.82341
PC(O-16:2	PC(O)	PC(O)+HC	564.3311	74.736	1064857.167	1040015.886	1051414.528
PC(P-16:0,	PC(P)	PC(P)+HC	566.3471	90.114	857414.8818	877557.9514	1092154.14
PC(16:1/2:	PC	PC+HCOC	580.3239	50.9295	18195.13406	12927.915	9819.503938
LPC(20:4/(	LPC	LPC+HCC	588.3286	70.293	101145.5002	82027.55644	97083.10313
LPC(20:3/(	LPC	LPC+HCC	590.3444	79.4625	26858.50767	23962.904	29207.6505
PC(O-16:2	PC(O)	PC(O)+HC	592.3611	96.748	48749.02613	47522.84149	59237.652
PC(P-16:0,	PC(P)	PC(P)+HC	594.3762	124.533	52841.698	45341.30552	53751.501
LPI(18:0/0	LPI	LPI-H	599.3157	83.634	4088.304	6328.157577	2628.74165
PC(18:4/2:	PC	PC+HCOC	602.3085	49.2865	45698.35838	31578.47394	34123.24782
PC(17:2/3:	PC	PC+HCOC	606.3395	75.5525	5951.76622	7226.591882	7923.040904
LPC(22:5/(	LPC	LPC+HCC	614.3453	73.208	180270.7852	183673.3953	233211.5812
LPC(22:4/(	LPC	LPC+HCC	616.36	86.409	15069.515	12743.905	15860.68
PC(17:2/4:	PC	PC+HCOC	620.355	88.167	4996.457109	5341.548	4783.216941
PC(P-18:0,	PC(P)	PC(P)+HC	622.4072	176.661	7168.207111	5471.829556	5951.616
PC(20:5/2:	PC	PC+HCOC	628.3243	50.9295	61763.7515	44478.91576	44849.92
PE(O-16:2	PE(O)	PE(O)-H	644.4636	369.641	3158.707824	4507.243826	6522.768
PE(15:1/1	PE	PE-H	646.4506	340.487	213.8006388	996.4883898	676.6644444
PE(P-16:0,	PE(P)	PE(P)-H	646.4792	411.837	6021.4952	5739.265563	7906.809625
PC(P-22:0,	PC(P)	PC(P)+HC	650.4369	238.538	8014.64366	8897.591455	6637.468133
PC(P-18:0,	PC(P)	PC(P)+HC	650.4437	131.738	6429.9096	5893.190471	9148.426941
PE(14:0/1(	PE	PE-H	660.4583	336.8995	4595.964	3329.537667	6507.836273
PE(16:0/1	PE	PE-H	662.4741	384.2105	8634.447083	9267.2671	12764.16333
PE(O-16:2	PE(O)	PE(O)-H	670.4786	374.949	24342.02621	25607.98872	36368.43068
PE(14:1/1	PE	PE-H	672.4662	341.205	646.2373657	2245.841941	1216.583615
PE(O-18:2	PE(O)	PE(O)-H	672.4951	417.266	47266.20684	53284.22117	66102.52114
PE(15:0/1(	PE	PE-H	674.475	369.6645	6056.490529	6832.050187	11082.13194
PE(P-18:0,	PE(P)	PE(P)-H	674.5109	458.944	54976.69076	54305.54206	91186.47635
PE(18:3/1	PE	PE-H	684.4599	309.9245	6687.482136	5914.202761	9168.17093
PE(P-16:0,	PE(P)	PE(P)-H	684.4909	420.076	31018.84	34079.21388	65573.4255
PE(O-16:2	PE(O)	PE(O)-H	684.4944	400.439	13262.86175	18328.81322	18098.214
PA(18:2/1	PA	PA-H	685.4785	357.157	7541.905824	8505.07625	14340.94618
PE(15:1/1	PE	PE-H	686.4742	370.471	7362.7176	7807.64785	12209.964
PA(18:1/1	PA	PA-H	687.4939	395.257	7859.240318	11821.76654	13502.64256
PE(P-16:0,	PE(P)	PE(P)-H	688.5242	482.366	23470.92817	23015.69164	33406.57359
PE(16:0/1(	PE	PE-H	690.5048	434.2455	40196.1405	39273.04792	53851.37429
SM(d14:0/	SM	SM+HCO(	691.5006	250.9615	4329.882952	4584.10176	5316.311

PE(15:1/18	PE	PE-H	694.4544	271.272	1694.101839	1764.751349	6756.243
PE(P-16:0/	PE(P)	PE(P)-H	694.4797	368.727	47031.23384	50636.48035	72647.79806
PE(P-16:0/	PE(P)	PE(P)-H	696.4941	394.35	76067.58029	64108.16942	73364.1986
Cer(t14:1/2	PhytoCer	PhytoCer+	696.6039	576.7755	42206.114	32213.216	37037.90147
Cer(t19:1/2	PhytoCer	PhytoCer+	696.6119	523.093	10527.05754	7447.8938	8617.925437
PE(P-16:0/	PE(P)	PE(P)-H	698.5106	426.32	325618.8699	363494.3891	425363.5295
Cer(t19:0/2	PhytoCer	PhytoCer+	698.6271	560.901	9947.668282	6805.827368	7619.779588
PA(18:1/18	PA	PA-H	699.4932	369.6405	15161.9835	115360.5244	31043.39853
PE(18:2/15	PE	PE-H	700.491	371.46	26114.6819	67845.17847	45566.26914
PA(18:1/18	PA	PA-H	701.5089	406.548	6930.507088	39032.32941	9383.728278
PE(15:0/18	PE	PE-H	702.5004	370.52	5989.389414	11336.96634	8138.554579
PE(P-16:0/	PE(P)	PE(P)-H	702.5333	463.624	150999.844	137253.2915	187075.2692
PA(16:0/20	PA	PA-H	703.5447	501.065	57313.21185	61360.29817	107167.3749
PE(17:0/16	PE	PE-H	704.5118	420.107	9818.579204	6905.049389	8405.717737
PE(18:0/15	PE	PE-H	704.5201	456.2515	7460.340227	6996.471737	11380.40583
Cer(t16:1/2	PhytoCer	PhytoCer+	708.6108	512.157	10317.7065	8073.751206	10382.14178
PE(14:0/20	PE	PE-H	710.4737	338.708	18312.32	20370.81542	27627.88889
PE(16:1/18	PE	PE-H	710.4744	317.122	11313.88313	10207.03579	17329.75478
Cer(t18:0/2	PhytoCer	PhytoCer+	710.6195	591.049	58639.75819	45444.72706	98154.17133
Cer(t15:1/2	PhytoCer	PhytoCer+	710.6273	542.6535	53972.75833	56857.983	61301.9145
PI(16:0/9:(	PI	PI-H	711.4109	131.9195	12977.61413	13365.37871	17928.77623
PE(18:2/16	PE	PE-H	712.4895	368.73	122203.7438	138277.9976	206646.6904
PE(16:1/18	PE	PE-H	712.4895	356.0255	49538.10536	48932.30713	59670.53885
PE(O-18:2	PE(O)	PE(O)-H	712.5255	450.99	166257.3801	194479.275	289890.4626
PE(16:0/18	PE	PE-H	714.4969	368.757	18499.59079	19896.51883	29147.36245
PE(18:1/16	PE	PE-H	714.5042	343.016	26856.74348	16997.698	25778.81321
PE(16:1/18	PE	PE-H	714.5051	400.447	555062.5657	636161.5028	769385.3137
PE(P-18:0/	PE(P)	PE(P)-H	714.5429	489.6095	706566.8014	877692.726	1381654.524
SM(d14:2/	SM	SM+HCOO	715.5	249.06	6392.176167	7045.899353	9743.546647
SM(d14:1/	SM	SM+HCOO	715.5087	342.992	11188.65186	8774.970893	11067.21165
PE(16:0/18	PE	PE-H	716.5213	438.926	812760.5011	817434.16	1059058.892
PE(P-18:0/	PE(P)	PE(P)-H	716.5537	522.197	24283.92376	25180.89856	37622.79956
PG(18:2/14	PG	PG-H	717.4729	277.7565	5692.675393	5246.932499	6060.608235
PG(14:0/18	PG	PG-H	717.4828	236.6	5201.328206	5228.484913	5903.7624
PE(18:0/16	PE	PE-H	718.5364	477.6305	63594.29206	62347.33165	99705.28941
PG(18:1/14	PG	PG-H	719.4825	352.7685	69741.84678	62803.45988	86163.61359
SM(d14:0/	SM	SM+HCOO	719.5331	312.304	548748.8664	523586.516	692554.6297
PE(P-16:0/	PE(P)	PE(P)-H	720.4945	384.635	550508.7338	589328.5768	681028.3653
SM(d22:0/	SM	SM+HCOO	721.5439	431.5275	1722.073077	1237.530009	2649.053333
PE(15:0/20	PE	PE-H	722.4591	396.57	1329.298769	146682.188	167371.8044
PC(24:0/4:	PC	PC+HCOO	722.4945	323.165	30128.82867	45522.13216	52141.3732
PE(P-16:0/	PE(P)	PE(P)-H	722.5114	418.277	235729.8	277393.4531	324680.4399
PE(O-18:2	PE(O)	PE(O)-H	722.5094	395.219	167190.4954	194668.9584	223522.2836
PA(18:2/20	PA	PA-H	723.5127	395.222	75742.38019	91871.17393	98630.03581
PE(15:0/20	PE	PE-H	724.4888	369.653	15609.76571	17812.09906	23758.07429
Cer(t16:1/2	PhytoCer	PhytoCer+	724.6407	559.125	8020.636105	8112.957263	7052.403167
PA(18:2/20	PA	PA-H	725.5285	433.368	114757.1757	131548.2319	153205.7868
PE(17:2/18	PE	PE-H	726.4916	439.929	6210.375882	5643.638467	5369.57975

PE(18:2/1	PE	PE-H	726.5047	389.912	38230.35933	64961.1072	37810.458
PE(18:3/1	PE	PE-H	726.5035	356.258	10935.38994	3786.445076	8862.907529
PE(17:1/1	PE	PE-H	726.5032	336.023	4473.424	4358.223195	5903.456364
PE(O-18:2	PE(O)	PE(O)-H	726.532	434.076	39618.73236	53188.03163	60156.65993
PE(P-18:0/	PE(P)	PE(P)-H	726.5435	472.498	997345.0262	1188706.215	1745922.945
PE(P-16:0/	PE(P)	PE(P)-H	726.541	561.776	5895.829526	6154.105263	9941.989047
Cer(t16:0/	PhytoCer	PhytoCer+	726.6576	590.7375	5700.817273	5059.616412	6422.494812
PA(18:1/2	PA	PA-H	727.5381	436.3485	9150.381864	11204.33074	13444.56409
PE(18:1/1	PE	PE-H	728.5111	455.366	2883.76324	2802.155163	3500.802
PE(17:0/1	PE	PE-H	728.5205	425.405	73892.94856	73783.20176	92709.37694
PE(P-18:0/	PE(P)	PE(P)-H	728.5431	561.771	10078.53423	7053.496471	12326.6822
SM(d16:2/	SM	SM+HCO	729.5163	272.1645	25681.61011	32525.71776	36948.47056
SM(d15:1/	SM	SM+HCO	729.5247	370.7425	7248.529645	12916.8261	8546.112
PA(17:1/2	PA	PA-H	729.5613	504.9315	504992.817	628967.1266	921949.2081
PE(18:0/1	PE	PE-H	730.5369	465.4535	126344.8793	129933.0636	221146.0097
PE(P-16:0/	PE(P)	PE(P)-H	730.5631	505.832	216394.6145	284140.852	204160.9503
PE(P-18:0/	PE(P)	PE(P)-H	730.5721	539.1025	39554.22537	48436.33653	75597.20693
PG(15:1/1	PG	PG-H	731.4995	371.4285	4484.409882	16610.77175	8070.392511
PA(18:0/2	PA	PA-H	731.5765	538.807	15347.89442	15949.28771	27393.68335
PE(14:1/2	PE	PE-H	732.4647	279.5925	4893.060778	3710.860714	6503.9664
PE(17:0/1	PE	PE-H	732.5428	465.4095	20056.87729	19181.10204	29525.37913
PA(22:6/1	PA	PA-H	733.4792	336.805	16110.12729	10397.82973	16100.2075
PA(17:1/2	PA	PA-H	733.4926	396.095	12220.07504	13523.07798	24163.81343
SM(d14:1/	SM	SM+HCO	733.5472	342.148	33513.48986	38795.64862	43963.03625
PE(22:6/1	PE	PE-H	734.4743	326.915	43928.53125	37207.94719	72417.29238
PE(18:4/1	PE	PE-H	734.474	311.5465	34054.18353	27824.34375	50044.27069
PE(16:0/2	PE	PE-H	736.491	359.3525	1065077.615	876632.3013	1394466.244
Cer(t17:1/	PhytoCer	PhytoCer+	738.644	590.672	21966.42706	27889.45819	76180.98316
PE(18:1/1	PE	PE-H	740.5118	369.412	172566.6667	190298.2091	245197.6085
PE(18:2/1	PE	PE-H	740.5207	408.273	360698.4163	544824.8967	622729.2821
PE(O-20:2	PE(O)	PE(O)-H	740.5562	492.3125	44626.18226	53782.92196	116360.6386
Cer(t17:0/	PhytoCer	PhytoCer+	740.6731	605.152	8095.5	6834.544333	9668.455688
PE(18:1/1	PE	PE-H	742.5375	449.0925	1541549.841	1511364.603	1775994.118
PE(P-20:0/	PE(P)	PE(P)-H	742.5715	524.8755	42027.612	55440.92712	80388.36704
PA(18:2/2	PA	PA-H	743.4836	396.9585	14664.36079	14399.68417	15322.14141
PG(18:2/1	PG	PG-H	743.4846	287.758	4816.578462	9029.599792	7113.414
PG(16:1/1	PG	PG-H	743.4812	312.116	7842.363742	6089.95208	11123.7089
PE(O-16:2	PE(O)	PE(O)-H	744.4942	370.53	150935.8342	150442.7353	216050.9653
PE(18:0/1	PE	PE-H	744.5537	482.335	961785.3731	1009878.758	1378730.879
PA(18:2/2	PA	PA-H	745.4994	436.871	14630.97558	13295.51337	50712.55356
PG(18:2/1	PG	PG-H	745.5	332.332	38715.27455	37444.3887	47597.31922
PC(12:0/1	PC	PC+HCOO	746.4908	291.25	12853.37346	24941.09338	18457.01192
PE(O-18:2	PE(O)	PE(O)-H	746.5098	388.129	326991.4867	388029.1171	451700.6442
PE(18:0/1	PE	PE-H	746.5659	517.7525	41341.81311	29796.49337	20458.23565
PG(18:1/1	PG	PG-H	747.5017	332.3185	7591.712	33031.10394	13416.10594
PG(16:0/1	PG	PG-H	747.5188	369.072	449992.4107	612696.71	641875.5899
PE(15:0/2	PE	PE-H	748.4896	353.631	29945.12181	41961.40083	44125.38178
PC(26:1/4:	PC	PC+HCOO	748.51	331.273	66125.25802	60839.52201	102744.9946

PE(P-18:0/	PE(P)	PE(P)-H	748.5262	435.87	657293.9805	687234.3813	855285.0838
PE(P-16:0/	PE(P)	PE(P)-H	748.5256	419.94	1345605.876	2970056.902	3493444.846
LPS(34:0/(	LPS	LPS-H	748.5652	373.2015	79415.67142	80293.75545	101189.8639
SM(d14:0/	SM	SM+HCO(	749.5677	372.3	29682.46478	31734.651	52170.40109
PC(26:0/4:	PC	PC+HCOC	750.5253	379.347	386064.772	353084.76	515214.1002
PE(P-18:0/	PE(P)	PE(P)-H	750.5411	464.524	314070.8415	232029.3172	483498.8452
PE(17:0/2(	PE	PE-H	752.5207	419.0835	29048.09071	39499.31739	37488.25867
PE(O-20:2	PE(O)	PE(O)-H	752.5548	474.8055	121952.2667	83234.099	182381.86
PE(17:0/2(	PE	PE-H	754.5351	439.545	29978.07669	43955.496	29487.62348
PE(20:2/1'	PE	PE-H	754.5352	383.704	22840.1768	11965.29972	18251.761
PE(19:0/1{	PE	PE-H	754.5346	403.961	7384.511976	7981.63213	12547.47261
PE(P-16:0/	PE(P)	PE(P)-H	754.5719	509.42	121317.6934	97136.901	141817.9165
PE(O-20:2	PE(O)	PE(O)-H	754.5709	484.167	36363.45912	40678.69352	53838.29733
Cer(t18:0/:	PhytoCer	PhytoCer+	754.6882	618.692	3755.877	4182.50325	5461.02888
PE(18:4/2(	PE	PE-H	756.4581	252.711	9632.403529	8242.498059	13174.33765
PE(20:1/1'	PE	PE-H	756.5506	423.5255	37343.61374	25306.53293	26982.00067
PE(19:0/1{	PE	PE-H	756.5514	470.9395	66544.8105	73245.14424	90438.17484
PE(O-18:2	PE(O)	PE(O)-H	756.5866	515.072	95581.48744	110911.292	138873.9245
PE(P-18:0/	PE(P)	PE(P)-H	756.5879	540.737	38308.12267	52417.80444	65168.4699
SM(d15:2/	SM	SM+HCO(	757.5461	333.3	17768.13	21747.62929	23901.99786
PE(18:3/2(	PE	PE-H	758.4738	285.2675	67260.77342	53888.31965	89965.17214
PE(20:5/1{	PE	PE-H	758.493	345.8275	27761.92491	29097.63965	40134.31067
PE(19:0/1{	PE	PE-H	758.5664	502.0595	35035.518	36608.07224	52209.86433
PE(P-18:0/	PE(P)	PE(P)-H	758.604	547.952	177342.5449	206383.6776	276066.896
PG(15:0/2(	PG	PG-H	759.5072	415.405	19524.30579	18537.96983	39970.49417
PG(17:0/1:	PG	PG-H	759.5161	431.598	25889.72544	24167.89076	61127.53094
PG(17:2/1:	PG	PG-H	759.5285	371.93	19880.10496	16840.86119	29779.33339
PE(18:3/2(	PE	PE-H	760.4818	286.154	14402.11838	9460.546721	16767.709
PE(18:2/2(	PE	PE-H	760.4911	324.128	763993.7664	592136.8037	972429.0812
PE(17:0/2(	PE	PE-H	760.6046	561.773	20578.8594	20394.44867	22860.32467
PG(19:0/1(	PG	PG-H	761.5203	353.629	27020.07413	27621.27007	32487.70564
PE(16:0/2:	PE	PE-H	762.5058	380.212	3095885.272	3243664.405	4131156.091
PE(18:2/2(	PE	PE-H	762.5067	366.922	1710543.138	1554375.517	2480051.808
PC(P-22:0/	PC(P)	PC(P)+HC	762.5608	461.069	54478.25858	45118.94147	75749.88114
PG(19:0/1(	PG	PG-H	763.5662	460.861	28858.2216	26588.64311	42682.97018
PE(18:0/2(	PE	PE-H	764.5207	351.873	232676.2037	161175.7248	215289.4492
PE(16:0/2:	PE	PE-H	764.5199	396.961	423491.9949	437513.2624	552073.8943
PA(20:5/2:	PA	PA-H	765.468	352.756	7214.420824	6972.116053	10681.45437
PE(18:0/2(	PE	PE-H	766.5373	442.15	838332.1516	846589.2898	991475.0671
Cer(t19:1/:	PhytoCer	PhytoCer+	766.6893	629.526	2256	2774.317	5391.078429
PG(16:0/2(	PG	PG-H	767.483	290.384	10190.44521	11500.34874	26368.8086
PG(18:3/1:	PG	PG-H	767.4841	263.2	5652.512077	5863.275	7504.140222
PE(20:1/1{	PE	PE-H	768.5507	402.2	100732.6335	99153.67235	121251.6731
PE(18:0/2(	PE	PE-H	768.5507	462.715	319866.3459	276907.495	551305.6263
PG(18:2/1:	PG	PG-H	769.4993	299.5055	18708.634	40645.42214	40897.6444
PC(18:3/1⁴	PC	PC+HCOC	770.4949	278.664	31403.03375	60060.924	47723.59769
PE(20:0/1{	PE	PE-H	770.5672	488.5245	214618.8124	218803.6808	330881.0176
PE(18:0/2(	PE	PE-H	770.5661	447.338	132660.6363	201715.4865	242786.1758

PG(18:1/1{	PG	PG-H	771.5036	299.402	11434.34194	8018.430444	8502.875118
PG(18:2/1{	PG	PG-H	771.514	338.926	23554.32882	25897.86	32556.63765
SM(d14:2/	SM	SM+HCO(	771.5602	381.0635	13303.03369	12922.22615	13552.88
PC(17:2/1:	PC	PC+HCOC	772.5082	274.914	32732.37	28416.75159	37464.15847
PC(18:3/1‹	PC	PC+HCOC	772.5105	303.2215	91018.84106	76782.79613	131479.7097
PE(20:1/1{	PE	PE-H	772.5812	486.0825	276047.7791	166479.2052	136871.0492
PE(18:0/2(	PE	PE-H	772.5822	520.404	93717.30714	103233.4493	136736.4417
PG(20:2/1(	PG	PG-H	773.5215	370.542	171029.2104	211312.4995	232350.7434
PG(16:0/2(	PG	PG-H	773.5304	410.067	692761.8343	918139.4293	1016903.259
PE(17:2/2:	PE	PE-H	774.5059	368.976	232171.7829	152665.3816	327445.1103
PC(18:2/1‹	PC	PC+HCOC	774.5268	343.0535	639832.2692	571407.406	880818.1904
PE(18:0/2(	PE	PE-H	774.5968	526.663	58093.89882	65410.119	95400.77372
SM(d14:1/	SM	SM+HCO(	775.594	489.659	7309.750809	7665.50104	8299.425522
PE(17:0/2:	PE	PE-H	776.5213	406.551	330175.2325	341141.928	464779.4357
PC(16:1/1(	PC	PC+HCOC	776.5422	386.36	1436032.128	1179607.756	1663552.157
PC(26:1/6:	PC	PC+HCOC	776.551	451.124	494241.3	615642.5689	732193.1156
PE(P-18:0/	PE(P)	PE(P)-H	776.5587	466.3245	631492.4593	690495.519	1110488.584
PG(18:0/1{	PG	PG-H	777.5512	450.925	101967.1592	129314.8762	152065.714
SM(d18:0/	SM	SM+HCO(	777.5986	426.753	11042.29425	6162.067455	8496.170308
SM(d14:0/	SM	SM+HCO(	777.6065	526.626	3385.21669	3167.527443	4198.523
PC(16:0/1(	PC	PC+HCOC	778.5575	431.52	1837580.739	1503807.332	2284038.701
PC(29:0/3:	PC	PC+HCOC	778.559	527.5815	24047.15314	9832.50075	14959.578
PE(P-18:0/	PE(P)	PE(P)-H	778.5725	494.2965	129185.5822	140763.4343	237688.6402
PC(O-16:2	PC(O)	PC(O)+HC	780.5156	321.3745	24224.13823	19251.60125	18079.74
PE(19:0/2(	PE	PE-H	780.553	464.452	34302.47888	36498.14327	48099.23175
PE(O-18:2	PE(O)	PE(O)-H	780.5811	586.747	5206.335414	6846.156	5010.22585
Cer(t20:1/:	PhytoCer	PhytoCer+	780.7041	641.348	1125.499125	1418.696118	1369.545063
PE(18:4/2:	PE	PE-H	782.4745	274.9465	115154.721	109884.8262	164664.3012
PC(O-16:2	PC(O)	PC(O)+HC	782.5291	287.05	12351.1284	12515.16819	24433.0183
PC(P-16:0/	PC(P)	PC(P)+HC	782.5313	331.4705	109792.5461	112079.5687	171919.8709
PE(17:2/2:	PE	PE-H	782.5655	490.597	8536.871111	29890.04229	65037.53414
PE(P-18:0/	PE(P)	PE(P)-H	782.604	544.5315	134569.2293	150510.6637	222877.4658
Cer(t20:0/:	PhytoCer	PhytoCer+	782.7192	642.376	2882.810062	3574.812	3379.088529
PE(20:5/2(	PE	PE-H	784.4905	309.126	283400	273380.1693	409601.9248
PS(14:1/2:	PS	PS-H	784.5057	439.4565	10641.45841	7750.620571	7899.051333
PC(O-16:2	PC(O)	PC(O)+HC	784.5451	369.6335	25717.2201	27093.54411	35120.4
PE(17:0/2:	PE	PE-H	784.5815	510.272	14440.11878	14984.15286	20420.29143
PE(P-18:0/	PE(P)	PE(P)-H	784.6182	573.0095	18936.57726	22587.82311	29867.08089
PG(19:0/1{	PG	PG-H	785.5204	320.43	15117.90135	21022.80113	20813.65
SM(d15:2/	SM	SM+HCO(	785.5788	389.923	475444.9979	540592.1191	684285.9136
PE(22:6/1{	PE	PE-H	786.5068	346.5335	4271752.363	3614628.136	4980800.796
PC(O-20:2	PC(O)	PC(O)+HC	786.5613	423.6995	103641.9788	115231.9818	135546.3503
PE(17:0/2:	PE	PE-H	786.5972	538.156	10617.80133	12181.0939	16511.27345
PE(21:0/1{	PE	PE-H	786.5956	509.399	9410.314125	9827.463114	12345.06337
PE(18:1/2:	PE	PE-H	788.5221	386.373	3634337.427	3395551.683	4327377.72
PE(18:2/2:	PE	PE-H	788.5219	366.032	570266.0755	478880.1725	875510.086
PS(18:0/1{	PS	PS-H	788.5417	438.9525	258.4744318	2982.044728	33.12563636
PC(O-22:2	PC(O)	PC(O)+HC	788.5765	465.054	648112.325	730097.112	733551.552

PG(18:3/2(	PG	PG-H	789.4672	316.217	4722.211706	5069.223	5865.271062
PE(18:0/2:	PE	PE-H	790.5379	429.785	6800778.013	7498923.52	8484498.007
PE(22:6/1{	PE	PE-H	790.5353	400.4215	248579.7103	231691.1464	291179.1429
PC(P-22:0/	PC(P)	PC(P)+HC	790.5941	474.7185	1787058.955	1720628.393	1674347.816
PC(P-18:0/	PC(P)	PC(P)+HC	790.5918	561.775	7164.214542	7294.334839	7191.53925
PA(22:6/2:	PA	PA-H	791.483	373.418	11116.94539	16216.574	15356.81271
PG(18:2/2(	PG	PG-H	791.483	255.6115	3214.5744	5196.114125	5610.5708
PG(16:1/2:	PG	PG-H	791.5024	313.6445	12197.68733	5340.79	19340.594
PG(19:0/1{	PG	PG-H	791.5988	474.788	720117.1776	701452.0325	683804.6745
PE(20:1/2(	PE	PE-H	792.545	429.256	801811.7082	884804.9527	993943.0735
PE(18:0/2:	PE	PE-H	792.5553	459.554	468009.351	583747.6098	707853.6778
PG(20:4/1{	PG	PG-H	793.5003	313.304	8623.044891	8404.725	16408.25394
PG(20:5/1{	PG	PG-H	793.4996	296.697	8826.133163	7282.902207	11187.43275
PG(22:6/1(	PG	PG-H	793.5181	343.976	9597.546167	5354.482235	11727.1062
PC(20:5/1	PC	PC+HCOC	794.4938	243.2225	53044.39742	50490.71008	61517.6942
PC(22:6/1:	PC	PC+HCOC	794.4988	281.4845	8065.018812	6154.683258	9371.4521
PE(18:0/2:	PE	PE-H	794.5595	443.469	111618.9354	102726.3737	126329.3615
PE(20:2/2(	PE	PE-H	794.5641	461.388	176627.4474	441799.7054	330363.5482
Cer(t21:1/:	PhytoCer	PhytoCer+	794.7202	627.722	2785.836118	3225.587375	3602.395941
PG(18:0/2(	PG	PG-H	795.516	343.964	9637.999404	7810.131559	12774.68596
PC(20:4/1	PC	PC+HCOC	796.5041	241.4535	13245.61739	23471.56377	12410.56137
PC(14:0/2(	PC	PC+HCOC	796.5101	294.9985	299189.9238	389077.2617	511949.2985
PC(18:4/1(	PC	PC+HCOC	796.51	278.688	108369.4545	94779.70251	141237.2311
PC(20:5/1	PC	PC+HCOC	796.5248	381.965	14758.78452	29104.35829	45153.92281
PE(22:2/1{	PE	PE-H	796.5808	503.005	32275.71655	257268.9586	305771.9142
PE(20:1/2(	PE	PE-H	796.581	489.614	50875.35863	47533.15713	68333.7915
Cer(t21:0/:	PhytoCer	PhytoCer+	796.7351	653.31	3689.896235	3859.60575	3874.716412
PG(18:2/2(	PG	PG-H	797.5273	346.578	4897.173745	4618.357588	9966.781067
PG(18:0/2(	PG	PG-H	797.5338	375.1555	19286.03433	8575.457917	22719.26935
PG(20:3/1{	PG	PG-H	797.5488	358.923	17861.5785	18996.02109	23397.2905
SM(d14:0/	SM	SM+HCO(	797.5741	388.9795	2871.86639	4371.992	4893.6072
PC(18:3/1(	PC	PC+HCOC	798.5266	310.734	201513.4642	174972.9688	260216.703
PC(18:4/1(	PC	PC+HCOC	798.5275	330.5155	493327.7549	449835.5027	573403.1326
PC(O-18:2	PC(O)	PC(O)+HC	798.5599	397.755	21800.06333	17987.89583	28127.42148
PE(20:1/2(	PE	PE-H	798.5979	523.033	51324.46642	52111.45614	67745.0585
PG(20:1/1{	PG	PG-H	799.5452	417.2	45948.95358	55758.66975	61565.41747
PC(18:3/1(	PC	PC+HCOC	800.5427	358.9145	1961139.491	1058810.756	2089138.384
PC(17:1/1'	PC	PC+HCOC	800.5404	291.534	50676.22462	52648.06148	41422.05992
PE(O-20:2	PE(O)	PE(O)-H	800.5578	454.4215	144771.6005	197327.6048	209670.3528
PC(O-18:2	PC(O)	PC(O)+HC	800.5744	421.8305	15851.02637	18394.31238	21769.66157
PE(18:0/2:	PE	PE-H	800.6128	553.2985	19637.13263	20813.08121	24622.42447
SM(d14:1/	SM	SM+HCO(	801.6069	429.003	10659.14538	10060.5558	12760.776
PC(17:1/1'	PC	PC+HCOC	802.5466	315.34	28651.72478	24107.35638	27589.968
PC(18:2/1(	PC	PC+HCOC	802.5591	396.091	11572705.83	11343561.27	13053536.68
PC(26:2/8:	PC	PC+HCOC	802.5775	466.33	34380.96933	35251.52327	45628.15725
PG(22:0/1(	PG	PG-H	803.5629	396.0915	4876756.021	4766211.002	5328894.207
SM(d14:0/	SM	SM+HCO(	803.6235	477.4	99645.53647	22405.1658	38046.528
PC(16:0/1{	PC	PC+HCOC	804.5741	435.11	11868963.93	12574002.2	14584876.65

PI(18:1/14	PI	PI-H	805.4773	278.6995	4143.806513	2925.03722	4848.826583
PI(18:2/14	PI	PI-H	805.483	258.444	753.35	1391.543636	1968.307714
PS(16:0/22	PS	PS-H	806.4917	368.743	13092.56341	14447.39269	13645.05512
PC(O-16:2	PC(O)	PC(O)+HC	806.5298	343.972	78464.82948	57697.32683	99804.22559
PC(18:0/1(	PC	PC+HCOC	806.5881	481.3975	441636.9237	393310.0644	514124.7634
PC(32:0/2:	PC	PC+HCOC	806.5917	560.901	67687.60317	72867.71294	60414.92917
PG(17:0/2:	PG	PG-H	807.5286	370.6985	18588.84546	17186.76719	18611.6416
PE(20:5/22	PE	PE-H	808.4907	298.585	812871.7371	931672.2412	1128241.469
PC(O-18:2	PC(O)	PC(O)+HC	808.545	376.986	148717.5221	149007.1508	195967.222
PC(P-16:0/	PC(P)	PC(P)+HC	808.5446	408.31	8334.978737	11250.99124	10973.27475
PE(22:6/2(	PE	PE-H	810.5075	335.25	166532.1859	217677.3902	205712.7693
PC(O-16:2	PC(O)	PC(O)+HC	810.5615	387.266	499589.8825	539691.8625	605284.7178
PC(O-18:2	PC(O)	PC(O)+HC	810.5613	412.763	88755.1742	107974.581	117943.4265
PC(P-18:0/	PC(P)	PC(P)+HC	810.5592	443.044	5014.262415	12518.06352	15423.09424
Cer(t22:0/	PhytoCer	PhytoCer+	810.7506	664.269	2180.570813	1643.589882	1616.150813
PI(22:6/11	PI	PI-H	811.4519	266.802	1449.792609	1542.807294	1935.381316
PG(21:0/1	PG	PG-H	811.5651	387.263	236659.5932	261790.6949	300436.8552
PG(24:4/1:	PG	PG-H	811.5617	370.7425	16388.60852	14096.70229	23518.72833
PE(22:5/2(	PE	PE-H	812.5152	336.872	29322.63554	36932.74423	36453.29455
PC(O-18:2	PC(O)	PC(O)+HC	812.5771	423.628	133740.4244	164580.0922	171228.3548
PE(P-18:0/	PE(P)	PE(P)-H	812.6494	601.8715	2844.395835	2987.778189	6563.705582
PG(19:0/2(	PG	PG-H	813.5768	394.351	37557.28314	20523.517	20714.75
SM(d15:2/	SM	SM+HCO(	813.6076	442.889	26030.14465	25897.92828	36663.48365
PE(22:6/2(	PE	PE-H	814.5369	394.354	405633.9634	436982.8537	720984.9396
PE(20:2/22	PE	PE-H	814.5357	506.652	15500.269	15165.35506	17142.69257
PC(18:2/1	PC	PC+HCOC	814.5552	381.97	223095.8348	211986.3349	238688.9444
PC(O-22:2	PC(O)	PC(O)+HC	814.5894	434.288	93662.74928	67263.19676	75798.53963
PC(O-18:2	PC(O)	PC(O)+HC	814.5918	469.5565	68037.66892	88212.14241	94973.06335
PE(23:0/1	PE	PE-H	814.6258	571.498	6926.4822	8351.374111	9292.613
PG(18:3/2:	PG	PG-H	815.4827	339.532	6641.091111	8119.0374	7848.4834
SM(d14:2/	SM	SM+HCO(	815.6228	455.878	9947.975623	10909.95241	12358.88923
PE(20:1/22	PE	PE-H	816.5529	433.329	509195.1157	443729.6492	570682.0143
PC(17:1/1	PC	PC+HCOC	816.5725	422.436	628820.433	606417.0537	705028.374
PC(P-22:0/	PC(P)	PC(P)+HC	816.6077	512.189	66000.20667	81315.98779	91786.199
PC(P-18:0/	PC(P)	PC(P)+HC	816.6076	530.288	13982.92843	17527.93353	18069.36514
PI(18:2/15	PI	PI-H	817.4771	312.895	6568.855713	1556.64	7558.721111
PG(18:2/2:	PG	PG-H	817.4985	278.654	5330.608636	10658.547	14848.11573
SM(d14:1/	SM	SM+HCO(	817.6387	501.891	42110.74933	20648.55	39192.12075
PC(18:4/1	PC	PC+HCOC	818.4935	227.161	28894.1087	25111.9427	38571.4875
PC(15:1/2(	PC	PC+HCOC	818.5741	472.9545	295332.6775	295423.7891	401182.8804
PC(17:0/1	PC	PC+HCOC	818.5868	461.7465	432772.5731	415930.0998	482555.7939
PC(P-22:0/	PC(P)	PC(P)+HC	818.6234	522.21	302524.2691	356889.9998	396161.8662
PG(18:1/2:	PG	PG-H	819.5147	426.7015	1894.358625	1920.4875	1863.111111
PG(18:2/2:	PG	PG-H	819.5155	318.6405	10736.96474	5726.61375	15469.24161
PC(18:4/1	PC	PC+HCOC	820.5099	261.33	158310.185	122037.0784	166799.2536
PC(33:0/2:	PC	PC+HCOC	820.6102	576.918	4438.472647	3777.2376	3689.852429
PG(22:6/1	PG	PG-H	821.5358	365.102	83359.50883	28578.87726	47515.91378
PG(18:0/2:	PG	PG-H	821.5477	343.425	34152.2802	37029.42622	47895.67008

PC(18:4/1{PC		PC+HCOC	822.5179	263.2325	73694.10187	55068.34543	80715.99119
PC(16:1/2(PC		PC+HCOC	822.5271	303.18	1752423.941	775775.6653	1115972.851
PG(22:2/1{PG		PG-H	823.5638	381.077	64792.15082	65151.93558	72545.73829
PC(20:4/1(PC		PC+HCOC	824.5327	301.7735	122443.1227	102426.027	154405.4181
PC(16:0/2(PC		PC+HCOC	824.5429	351.8665	6752225.936	6791296.718	8577959.532
PC(18:3/1{PC		PC+HCOC	824.5432	325.008	1647193.548	1421523.894	1816576.578
PE(20:1/2:PE		PE-H	824.637	605.1795	8205.787778	9057.303231	14271.01829
PG(24:4/1(PG		PG-H	825.5787	410.0845	23684.90951	9885.26	32330.61982
SM(d16:0/SM		SM+HCO(	825.6072	426.346	15541.32957	15136.84344	19056.51107
PC(18:2/1{PC		PC+HCOC	826.555	309.787	55912.41067	49563.81203	48103.3784
PE(20:1/2:PE		PE-H	826.6336	561.778	7666.659	13381.60371	19508.46373
SM(d18:2/SM		SM+HCO(	827.6227	435.89	42115.92488	43826.77316	59073.39945
SM(d14:2/SM		SM+HCO(	827.6317	477.2885	63056.92265	62134.12044	84607.0082
PE(O-22:2	PE(O)	PE(O)-H	828.5709	561.788	5006.396484	5539.272567	6017.8452
PC(18:2/1{PC		PC+HCOC	828.5731	402.205	4370670.546	3931617.131	4415494.519
PC(22:2/1‹PC		PC+HCOC	828.583	493.3985	10455.04829	12867.30967	16338.332
SM(d16:1/SM		SM+HCO(	829.6412	477.5855	593538.4309	554142.6796	753126.2776
SM(d14:2/SM		SM+HCO(	829.647	584.654	10463.00102	8741.310586	12578.40829
PC(18:0/1{PC		PC+HCOC	830.5899	447.3515	5255680.814	4593245.21	5886840.949
PI(16:0/18	PI	PI-H	831.4939	380.283	11723.11283	11297.70494	12713.10013
PI(18:2/16	PI	PI-H	831.4984	292.332	6078.469299	7366.472722	7130.788765
SM(d18:0/SM		SM+HCO(	831.6468	477.472	68050.8525	66132.27716	129850.0432
SM(d14:0/SM		SM+HCO(	831.6558	521.3105	416809.8278	309577.7957	451674.245
PC(18:0/1{PC		PC+HCOC	832.6052	485.0575	3344169.676	3813235.377	4060172.276
PI(18:2/16	PI	PI-H	833.5144	314.2715	35564.20636	53325.78522	31428.5198
SM(d18:0/SM		SM+HCO(	833.6614	521.3045	70533.63545	47140.85567	68921.97545
SM(d17:0/SM		SM+HCO(	833.6687	539.66	46577.446	40625.22	50201.45964
PE(22:6/2:PE		PE-H	834.5263	368.7655	49194.96778	171151.0629	58367.70027
PC(O-16:2	PC(O)	PC(O)+HC	834.5607	396.9515	466751.7865	518764.7707	520731.5291
PC(20:0/1(PC		PC+HCOC	834.6175	528.501	66277.16755	64783.9927	77011.68052
PC(34:0/2:PC		PC+HCOC	834.6218	591.276	7455.793529	6969.474261	5898.793067
PI(18:1/16	PI	PI-H	835.53	368.887	39508.17825	92368.12867	39014.3475
PC(17:1/2(PC		PC+HCOC	836.5244	339.5335	472947.335	448464.3521	579608.481
PE(22:6/2:PE		PE-H	836.5371	385.481	11472.29007	23545.65565	4276.8
PC(O-16:2	PC(O)	PC(O)+HC	836.578	408.006	3498923.282	3901721.609	3348913.39
PG(19:0/2:PG		PG-H	837.5818	407.445	1567298.99	1738955.748	1536375.051
PC(15:1/2:PC		PC+HCOC	838.5438	370.488	116435.436	126975.7195	175969.2826
PE(22:5/2:PE		PE-H	838.5527	487.849	8441.491474	20968.93994	8885.10155
PC(20:5/1'PC		PC+HCOC	838.5546	380.213	105644.5351	99482.34323	126241.3955
PC(O-18:2	PC(O)	PC(O)+HC	838.5914	425.455	313854.0741	300617.7821	328011.3695
PG(21:0/2(PG		PG-H	839.5848	407.4235	99016.09895	108927.4898	97500.51713
SM(d17:0/SM		SM+HCO(	839.623	444.79	78091.515	63288.20358	94838.58057
PE(22:5/2:PE		PE-H	840.5679	519.586	9474.828882	6034.9206	5021.575364
PC(O-16:2	PC(O)	PC(O)+HC	840.6063	473.8565	73826.80997	81541.23717	48684.33765
PG(22:5/2(PG		PG-H	841.5191	272.114	16358.0655	5891.542938	9125.542812
SM(d19:2/SM		SM+HCO(	841.6275	443.884	15429.16791	15340.78686	17647.02424
SM(d15:2/SM		SM+HCO(	841.6397	492.3795	159005.6184	175916.2482	243967.8745
PC(19:0/1{PC		PC+HCOC	842.5817	525.789	7601.074916	4969.861143	11508.08582

PC(20:2/1' PC	PC+HCOC	842.5859	426.3625	58842.65382	57497.7	63752.40769
SM(d17:1/ SM	SM+HCO(	843.6546	509.423	100714.9416	107941.4174	135984.28
SM(d14:2/ SM	SM+HCO(	843.6523	561.668	10788.86004	7998.450429	14656.34244
PC(18:4/2(PC	PC+HCOC	844.5114	244.236	256511.7827	234848.6007	390622.0869
PE(22:6/2: PE	PE-H	844.5821	547.965	4404.90731	5294.067383	4620.632867
PC(19:0/1{ PC	PC+HCOC	844.6008	472.9825	174374.8933	182792.1883	188579.8724
PC(P-16:0, PC(P)	PC(P)+HC	844.6589	535.073	23556.30213	23996.22604	29324.74336
SM(d19:0/ SM	SM+HCO(	845.658	501.064	39079.63006	38501.24833	50417.70282
SM(d15:0/ SM	SM+HCO(	845.6718	545.5515	317007.1245	170443.7218	263811.8648
PC(20:4/1{ PC	PC+HCOC	846.5273	276.836	1050845.465	899323.6107	1422616.22
PC(19:0/1{ PC	PC+HCOC	846.6192	509.4705	136149.9588	128739.8375	135294.9593
PC(P-22:0, PC(P)	PC(P)+HC	846.6612	551.281	259323.4199	185237.4843	260092.6696
PC(20:3/1{ PC	PC+HCOC	848.5436	316.0275	6559829.759	5202459.237	6496110.777
PC(18:2/2(PC	PC+HCOC	848.5414	367.833	46745.72639	19126.2039	47321.24543
PC(21:0/1(PC	PC+HCOC	848.6538	551.3385	66731.23744	65640.70376	113309.9045
PI(20:0/15 PI	PI-H	849.5584	345.737	158585.68	158653.4994	240585.1143
PC(22:6/1(PC	PC+HCOC	850.5575	373.1895	7193877.466	11161977.32	8282925.807
PC(20:5/1{ PC	PC+HCOC	850.5582	357.136	2974148.539	2948520.13	3638762.582
PC(22:5/1(PC	PC+HCOC	850.5557	339.533	362734.9478	275088.5372	407249.6712
PI(20:0/15 PI	PI-H	851.5618	373.2015	3171212.454	5052715.715	3724440.491
PC(22:4/1(PC	PC+HCOC	852.5623	357.129	373712.2022	388847.8047	454757.933
PC(24:4/1‹ PC	PC+HCOC	852.5633	373.1895	1014301.634	1039489.158	1191847.12
PC(18:0/2(PC	PC+HCOC	852.5717	405.685	1222671.326	1409244.635	1607564.501
PC(16:0/2: PC	PC+HCOC	852.5726	390.8035	4066478.092	1208895.866	1515199.163
SM(d18:0/ SM	SM+HCO(	853.6398	474.352	197876.6331	225586.8223	244548.3266
PC(18:0/2(PC	PC+HCOC	854.5861	438.651	197501.1156	228391.6195	236400.3371
PC(18:2/2(PC	PC+HCOC	854.5849	411.597	613713.3882	682708.9456	666511.8718
PI(16:0/20 PI	PI-H	855.4986	272.173	18630.92012	22635.81271	13401.60564
SM(d20:2/ SM	SM+HCO(	855.6558	483.157	533229.6015	444708.6794	688516.2737
PC(20:1/1{ PC	PC+HCOC	856.6032	450.778	643299.9606	709475.466	605912.1896
PI(16:0/20 PI	PI-H	857.5145	308.6505	64152.86104	49470.65206	29274.72567
PI(18:2/18 PI	PI-H	857.5141	281.461	22976.37778	34252.00776	16396.50057
SM(d20:1/ SM	SM+HCO(	857.6723	518.642	6127629.769	6697506.255	7306016.8
PC(20:1/1{ PC	PC+HCOC	858.6193	490.5295	668575.3642	634545.7943	722082.3549
PI(18:1/18 PI	PI-H	859.529	322.263	31685.248	50047.74013	28148.26024
PC(22:6/1' PC	PC+HCOC	860.5375	322.31	67368.332	85158.564	71340.75865
PC(O-18:2 PC(O)	PC(O)+HC	860.5858	474.872	12519.76544	6632.128851	6375.924818
PC(22:1/1(PC	PC+HCOC	860.6363	529.384	342314.2518	355254.393	331445.621
PI(18:0/18 PI	PI-H	861.5482	367.814	140839.7078	247633.5122	141415.66
SM(d18:0/ SM	SM+HCO(	861.6922	566.139	128083.6673	65722.30184	105190.3459
PC(22:6/1' PC	PC+HCOC	862.5537	366.8635	117748.215	178148.5712	137101.5948
PC(O-18:2 PC(O)	PC(O)+HC	862.5911	448.26	63161.97521	86303.88295	80613.59711
PC(33:0/5: PC	PC+HCOC	862.6503	572.9125	62536.79529	51916.47273	56838.34205
PI(18:0/18 PI	PI-H	863.5586	358.0965	157577.6188	149657.8107	160398.2906
PC(22:6/1' PC	PC+HCOC	864.5702	399.5515	202048.185	150122.6899	160883.7235
PC(22:4/1' PC	PC+HCOC	864.5697	344.815	75029.55386	91285.376	96278.421
SM(d19:1/ SM	SM+HCO(	865.6489	541.633	5566.938492	5434.845245	7316.198312
PC(22:4/1' PC	PC+HCOC	866.5853	374.063	78212.61205	75606.22588	84699.02371

PC(24:4/1:	PC	PC+HCOC	866.5869	418.1355	50217.65632	54720.84432	51027.60093
PI(22:5/15	PI	PI-H	867.514	265.9095	10836.59775	8754.302118	15092.24248
PG(22:5/2:	PG	PG-H	867.5368	322.403	13121.26473	14190.39447	14082.256
SM(d19:0/	SM	SM+HCO(	867.6562	490.661	971300.3005	1114791.522	1286659.78
PC(22:2/1'	PC	PC+HCOC	868.592	420.923	14261.38809	14696.50668	16898.80706
PC(19:0/2(	PC	PC+HCOC	868.6028	463.595	11926.49686	13703.47857	13103.60867
PI(17:0/20	PI	PI-H	869.5146	300.4305	7028.532214	9750.342619	8633.635706
SM(d21:2/	SM	SM+HCO(	869.671	536.841	94568.4555	120513.2384	154416.5667
PC(22:6/1{	PC	PC+HCOC	870.5271	266.797	2510093.4	2190688.775	3113882.349
PC(20:5/2(	PC	PC+HCOC	870.5435	396.094	59145.66237	53351.07743	55848.6371
PC(22:1/1'	PC	PC+HCOC	870.6169	459.87	8749.220833	6829.113445	15824.68626
PC(O-18:2	PC(O)	PC(O)+HC	870.6712	556.127	12092.4067	13475.21622	22442.79071
PI(17:0/20	PI	PI-H	871.5296	336.014	14825.65483	20357.56935	13470.2645
PI(20:4/17	PI	PI-H	871.5518	396.09	30866.79379	30473.46317	28700.36122
SM(d16:2/	SM	SM+HCO(	871.6857	542.609	117262.8379	125498.1539	142958.619
PC(22:6/1{	PC	PC+HCOC	872.5346	336.0185	81588.35533	88708.15549	98297.04114
PC(18:3/2:	PC	PC+HCOC	872.5425	302.228	1493778.261	1421852.632	1749418.556
PC(15:1/2·	PC	PC+HCOC	872.6268	510.312	15403.005	13122.98571	16266.52
SM(d16:1/	SM	SM+HCO(	873.7013	587.59	50385.91	30044.41863	62038.26526
PC(22:6/1{	PC	PC+HCOC	874.5581	338.647	6175560.452	6343674.294	6419845.919
PC(20:4/2(	PC	PC+HCOC	874.5544	318.579	165897.9335	157980.1929	190611.2438
PI(18:2/19	PI	PI-H	875.5573	384.584	12011.792	16483.0848	14891.80405
LacCer(d1	LacCer	LacCer+H	876.5557	228.349	31636.02802	25337.83919	18237.96563
PC(22:4/1{	PC	PC+HCOC	876.5716	356.261	861086.5521	784516.9503	908793.7716
PC(22:6/1{	PC	PC+HCOC	876.5726	378.496	3383949.192	3846383.628	3559165.974
PC(23:0/1(	PC	PC+HCOC	876.6657	593.469	32120.84852	26104.95947	34849.35719
PC(35:0/4:	PC	PC+HCOC	876.6788	564.407	38227.57725	26322.76595	37954.20321
PS(22:6/2:	PS	PS-H	878.4925	256.567	3613.858383	3256.860765	3664.223
PC(18:0/2:	PC	PC+HCOC	878.5884	424.5335	1501072.064	1806522.924	1671089.395
PC(22:2/1{	PC	PC+HCOC	878.5867	409.1735	401388.239	421101.655	449183.8346
PC(22:4/1{	PC	PC+HCOC	878.5856	394.3735	482862.1629	454854.6996	543653.1771
PI(18:2/20	PI	PI-H	879.4982	237.5835	14283.70647	15767.86824	14005.30376
PC(18:0/2:	PC	PC+HCOC	880.6024	457.3915	133400.4762	144591.5121	142111.287
PC(24:4/1(	PC	PC+HCOC	880.601	441.241	212100.5324	249886.84	228109.9737
PI(16:0/22	PI	PI-H	881.5142	295.822	61152.31418	83655.05175	59184.00675
PI(18:1/20	PI	PI-H	881.5151	279.5985	70514.58315	92378.41867	70870.7592
PI(18:1/20	PI	PI-H	883.5213	295.638	13480.34366	16890.15314	13476.53332
PI(18:0/20	PI	PI-H	883.5308	326.8	315463.9903	398312.9601	225225.7106
SM(d18:2/	SM	SM+HCO(	883.6861	562.5475	23932.97822	29792.72154	19721.70128
PC(26:2/1·	PC	PC+HCOC	884.6341	497.175	131095.2228	128919.7165	118491.04
PE(28:0/1{	PE	PE-H	884.7042	650.621	1719.195175	2493.28722	3927.749913
PI(18:0/20	PI	PI-H	885.5465	360.6905	363991.0649	632176.431	312908.6573
SM(d17:2/	SM	SM+HCO(	885.7007	563.525	132900.316	119813.0967	175910.9471
PC(24:1/1(	PC	PC+HCOC	886.6487	532.8775	118147.0607	118746.1025	142853.1545
PI(18:0/20	PI	PI-H	887.5587	381.078	9969.846176	16850.06738	10363.12507
SM(d18:0/	SM	SM+HCO(	887.7058	563.526	26057.4803	23573.02007	44310.03121
SM(d17:1/	SM	SM+HCO(	887.7162	606.5635	35638.2	31939.54292	50912.89762
PC(24:1/1(	PC	PC+HCOC	888.6647	572.18	143320.2545	156917.9573	197556.9175

PC(36:0/4:	PC	PC+HCO	890.6715	572.117	24292.04648	34830.73486	41003.27188
PC(24:0/1	PC	PC+HCO	890.6797	612.326	81863.09657	81033.48497	126289.074
PC(24:4/1	PC	PC+HCO	892.6021	449.3695	14393.6298	44487.53745	46359.93661
PC(O-18:2	PC(O)	PC(O)+H	892.6395	560.902	13566.6358	11747.45775	9777.368733
PC(19:0/2	PC	PC+HCO	894.6313	561.809	2716.6884	3451.229353	6056.682542
PI(17:0/22	PI	PI-H	895.5293	322.272	18587.595	30636.91112	18305.09363
PC(22:6/2	PC	PC+HCO	896.537	322.363	435951.9207	629845.5735	584507.0385
SM(d19:2/	SM	SM+HCO	897.7007	578.755	15538.4627	21885.84391	28532.73805
PC(22:6/2	PC	PC+HCO	898.5572	327.72	628632.801	800600.6317	722555.9869
PC(O-18:2	PC(O)	PC(O)+H	898.7036	578.822	9183.65495	12095.3877	17313.56918
PI(19:0/20	PI	PI-H	899.56	327.7005	333353.5973	388537.7765	373034.5729
PC(22:5/2	PC	PC+HCO	900.5704	348.294	336556.0708	330151.3416	307191.0449
LacCer(d1	LacCer	LacCer+H	900.5914	242.349	14585.54926	11270.416	6891.908684
PC(24:1/1	PC	PC+HCO	900.6639	560.031	21284.75438	23138.23725	32908.51123
LacCer(d1	LacCer	LacCer+H	902.5703	250.6095	31622.66829	27174.52512	16375.72071
PC(22:4/2	PC	PC+HCO	902.586	388.0925	218429.6139	205969.9897	237501.4049
PC(20:2/2	PC	PC+HCO	902.597	487.39	24076.29919	26151.39975	22611.27062
PC(24:0/1	PC	PC+HCO	902.6794	594.515	22249.97528	31727.23728	37688.75522
LacCer(d1	LacCer	LacCer+H	904.5872	283.172	44615.219	33473.15593	25789.88271
PC(37:0/4:	PC	PC+HCO	904.6845	595.918	6526.204609	8318.159793	7782.0246
PC(25:0/1	PC	PC+HCO	904.6943	631.5345	8843.263	12289.23417	13931.46175
PI(18:2/22	PI	PI-H	905.514	261.3145	43418.82124	61234.89	38300.49341
PI(18:2/22	PI	PI-H	907.5291	277.7485	12861.82767	16274.87822	11983.18459
PI(18:1/22	PI	PI-H	907.5306	302.3065	171497.1053	266499.522	156057.8263
PI(18:1/22	PI	PI-H	909.5435	317.9615	17395.71762	31903.79523	16301.36081
PI(18:0/22	PI	PI-H	911.5602	363.365	100010.6967	178644.6655	99965.46626
PC(26:2/1	PC	PC+HCO	912.6636	538.9455	92166.775	103636.8	151681.6964
PI(18:0/22	PI	PI-H	913.5735	394.3505	6304.37473	8034.627786	5346.502071
LacCer(d1	LacCer	LacCer+H	914.5708	221.8	29320.2954	18013.15442	25170.70069
PC(24:1/1	PC	PC+HCO	914.6807	574.913	248204.4853	295416.7035	435822.3919
PI(20:2/20	PI	PI-H	915.591	386.3355	8469.106708	6371.802	7575.045333
SM(d19:1/	SM	SM+HCO	915.7422	642.457	2967.286705	3768.515669	5681.834483
LacCer(d1	LacCer	LacCer+H	916.5869	263.198	19331.74483	14306.63228	12607.77621
PC(24:0/1	PC	PC+HCO	916.6966	612.1965	229523.3494	295677.4993	557058.3229
PC(38:0/4:	PC	PC+HCO	918.7024	612.074	33538.34914	54059.06829	92542.56044
PC(26:0/1	PC	PC+HCO	918.7085	650.341	16117.0603	15561.36709	27853.056
PI(19:0/22	PI	PI-H	923.5586	371.423	10750.47106	20059.92031	10198.93041
PI(22:6/19	PI	PI-H	923.559	312.481	1053432.275	1121978.379	965621.7842
PC(22:6/2	PC	PC+HCO	924.5637	371.434	19125.71916	25895.18342	28449.35044
LacCer(d1	LacCer	LacCer+H	928.5863	250.877	85872.68789	40620.8392	57665.4764
PC(26:1/1	PC	PC+HCO	928.694	597.8985	25491.9484	28855.22194	46888.992
LacCer(d1	LacCer	LacCer+H	930.6015	281.492	31719.17	23026.7678	21890.64
LacCer(d1	LacCer	LacCer+H	930.6298	372.314	6211.361654	10836.06505	10561.88981
PC(25:0/1	PC	PC+HCO	930.7104	631.6845	12491.70133	22224.46358	30919.35086
PI(20:2/22	PI	PI-H	933.5437	311.521	4034.24375	6216.054706	4824.7066
PI(20:1/22	PI	PI-H	935.5592	351.02	7513.8624	12359.484	7271.411882
PI(20:1/22	PI	PI-H	937.5752	366.9165	5829.933262	8409.491647	7256.530867
PC(26:2/1	PC	PC+HCO	940.7013	662.893	1667.346443	2277.754481	2441.390579

PC(26:1/1	PC	PC+HCOC	942.7101	611.1095	37444.98529	55046.04126	80119.112
PC(26:0/1	PC	PC+HCOC	944.7259	649.335	21985.41881	31373.4252	51877.2765
LacCer(d1	LacCer	LacCer+H	990.704	496.4485	3072.137598	3409.586684	5026.457579
LacCer(d1	LacCer	LacCer+H	1014.699	462.731	1451.321757	1525.852119	2961.342125
LacCer(d1	LacCer	LacCer+H	1016.717	495.589	8671.51075	12747.97	23549.37663
LacCer(d1	LacCer	LacCer+H	1018.731	531.249	3352.939333	3608.683261	5873.2135

· in three groups

CAP-04	CAP-05	CAP-06	CAP-301	CAP-302	CAP-303	CAP-304
3356.619545	5668.519667	3949.492	6235.908305	5391.5455	8391.215294	6384.100364
19363.23	18501.27625	25711.32271	41482.93788	23784.15212	17438.30638	35472.55024
1488404.479	1043005.795	1727014.289	1524493.537	980781.4338	983179.0599	789545.5675
21554872.71	10836117.09	17519495.61	18783218.09	20096902.63	15059244.76	11234697.22
8035.731	8601.113298	851.6684444	11953.76614	7985.219043	6321.742304	6528.396875
367343.5864	265039.8113	409535.7954	391734.4694	221397.5753	257468.4552	185817.7564
12413542.76	10681184.88	9825986.131	17267479.65	12373549.13	8908701.372	10591449.94
17343.54491	7825.657	14487.02357	10864.68893	13390.41289	13245.54425	3401.708545
29452.465	72864.176	51033.23143	65632.08523	70395.53154	84274.01542	84307.64123
805333.9571	209151.4011	295668.3656	353449.3227	871.824	76875.23049	155042.3351
221341.7784	169199.8187	195894.8638	217219.6596	167880.1149	159272.7457	169769.5736
20239.69148	12728.51956	16131.36923	45328.16792	33387.97286	58899.02964	55901.74571
298383.8744	169916.1627	334716.9723	300077.3469	164197.4839	141243.5369	124051.6453
1202548.369	719782.9565	956585.5492	761312.5421	407951.6935	537330.9385	355328.0875
26331.435	80539.12714	42505.929	60907.558	50414.47	71019.19386	80188.25143
12568.40243	10315.01889	16405.39583	14303.9183	17115.68574	8319.972737	3836.83125
76455.98673	150847.085	117059.4375	128501.464	129576.3228	163496.0614	235269.6009
199933.4846	765959.1895	387615.1428	493808.1373	541921.1666	831304.1486	1239617.191
98985.86793	80043.62573	87494.48583	118297.5722	83215.71012	133724.6968	127865.1398
13139.5302	32780.57309	24115.62633	59584.60679	36542.5767	50922.11025	47089.03091
513355.8812	330592.4906	452909.5366	402171.2024	354772.4991	404022.6225	337445.7971
23899.0623	28076.71061	14359.38	30339.85975	32613.04715	28898.49589	51985.78127
200908.5804	541805.0564	376056.606	417855.8038	390068.107	742466.2126	1037361.931
188203.3523	469187.8579	337433.9755	368820.4	531677.9366	615185.6734	930106.1423
84882.42271	82958.02059	83782.61111	90225.26489	92408.36984	85647.48065	76919.47228
30891.15482	84616.91071	33851.13218	42785.16529	16567.64775	21015.4986	17749.69085
29471.079	43059.19745	40956.11518	45011.15964	45549.87375	66277.9745	115442.9389
22722.525	25429.59317	42251.3616	29796.13	27115.616	43034.67053	58879.97445
197729.0505	108465.1293	235144.5586	207419.7189	154816.1813	95829.4784	161813.129
78430.11881	188802.8966	152268.62	206629.7357	286865.9699	340466.3043	654397.8556
842273.7504	812440.1219	679196.1737	1215752.904	826648.3749	602155.6963	730481.901
4353.94436	3043.601132	8365.128667	9113.591667	8090.883562	7607.042667	9954.2352
21483.79626	16551.98494	25044.626	26823.52812	22602.29206	18899.11704	22885.471
35869.47692	34262.02667	29720.0475	41914.4	39894.1686	41506.245	38196.4
383117.3307	353789.0233	492961.9672	493983.512	479992.0461	317233.0907	452790.862
8920.63848	10152.164	9141.195913	7129.472944	7285.525	2536.454015	2494.328617
1034309.383	767255.7609	1196199.807	1403158.431	1071810.578	735919.7942	1032123.703
10276.6158	9704.1126	7640.946692	16133.42912	9273.633923	8543.206385	10696.0872
12111.5215	14729.4504	17290.985	19914.7325	13506.49222	10984.96676	16968.74793
11861.60456	9534.079571	6189.1458	17872.52743	16487.4476	7202.755875	8004.082952
42735.39646	34049.36529	40751.91389	30368.54455	26947.77408	33824.2025	49397.66357
22164.33833	20490.13637	22145.54247	22616.48138	20512.26576	13241.33433	16213.25659
415666.7928	410385.0558	482231.9851	519664.8542	476960.5869	346569.2544	485738.2485
259570.1739	233411.436	268610.6985	306953.6911	263292.4748	197139.5269	258968.4059
251143.0924	188511.9683	309362.3584	296168.0732	271538.3521	180037.5679	256540.1569
13495.805	18059.38615	10289.3925	15345.72108	10746.03091	10999.8	9130.200175

11223.07779	10877.58	8646.882	15069.5402	10994.39053	15798.99087	11699.48953
17127.858	15149.13754	20324.388	22450.36362	12340.782	9179.221071	11566.16792
8583.43	5925.948	6398.5735	18875.6505	21927.60074	11347.11286	14624.1
19549.24354	19503.28286	43498.59073	21435.97292	20549.316	21827.30786	40230.80291
151690.7424	230595.0405	161288.8368	179295.5734	216807.3594	175846.2315	226961.65
7758.378529	16291.56633	6009.2624	9426.258253	6433.724991	4825.743083	4480.025786
26818.14721	22297.00567	26005.75977	40180.92071	27690.78	34148.1825	33976.50936
68673.11721	76960.69231	68409.45254	75592.22875	65382.30887	72233.78887	52107.43609
4468.486846	4553.309668	5558.275286	5487.505933	4738.238211	3981.112235	4161.116923
16168.57077	15131.84863	43837.01728	20301.3252	14770.505	15872.79533	23497.444
9962.026364	13539.75319	5256.030182	5052.38	3509.183	10619.36737	2403.6012
2404.843462	3129.243133	2424.756923	2994.475	1691.382727	2649.833231	2780.895333
8865.674235	11378.5418	10112.61071	11550.963	12293.51261	8668.446667	14942.9252
20893.69421	20889.88573	39725.64307	34563.16289	36089.00489	21118.46713	28272.90787
8701.204909	7029.101579	9085.57625	9945.870544	8088.75375	6019.5368	8147.660231
1288429.254	1722055.956	1281269.827	1266029.481	969801.5141	982366.7169	1030522.611
1439012.994	1056476.418	1466676.903	1435665.005	1034762.685	1042351.175	820086.8204
355759.5944	454131.8406	488018.3664	398931.2507	511222.2452	483542.9328	659913.054
46008.37041	35660.33659	87546.47344	38240.14333	42034.707	40915.9898	68752.05664
11090.83333	6265.9671	8269.54625	13774.166	8743.899154	9861.542857	9797.89225
8925.50525	9497.129765	7327.166923	9490.554071	5746.700769	7366.858778	6197.851
1861.896	2315.782726	2497.602857	3414.118235	2189.327842	1564.568735	2429.850889
78278.05837	67792.51178	35741.08491	16691.86286	46972.85809	52078.16018	25877.25
30047.34308	26242.84769	26700.82938	35521.93363	29675.40577	29989.74769	29672.06746
287229.7992	364983.966	307716.682	276863.2731	209519.3232	234081.5847	250345.2202
142526.56	163637.5506	146689.9986	140537.5829	115321.1215	122194.4793	134591.3296
346893.8847	378084.861	323234.6171	332922.5967	328619.04	257091.4947	290157.6981
35547.6	28204.05872	26654.18631	31448.93744	36283.48	18283.77517	19273.31146
203534.0571	361534.7215	194802.3231	192819.6278	146397.8536	172658.3143	166978.6899
306622.402	832601.7952	291564.4445	298198.3684	254525.6	285678.5325	255748.617
409903.8346	603890.32	494527.6342	356229.4702	380172.5346	403753.2008	312511.6109
223600.8688	318994.34	217288.1778	237542.3419	213576.2849	236143.1154	222201.6028
6810.228	6947.205185	7275.6966	11629.09389	10072.92875	7980.729583	8847.045
39388.22775	45839.15115	50529.65886	56942.595	47791.30883	37183.3629	58671.0795
1544457.614	1995342.522	2874945.244	1599355.32	2125340.514	1278756.706	1253201.968
17306.8932	11079.23046	16336.88862	28911.129	16464.69862	13840.122	12964.35938
13813.16928	12729.87241	7376.877176	11864.80614	7851.156538	8087.990438	5975.538643
12056952.36	14868956.47	12072496.7	12495214.98	8742271.029	10580103.32	10672646.48
5457622.72	6121229.233	5320414.893	5506467.755	4046259.495	4976514.041	4913325.343
120373.7309	75210.323	99426.525	125769.9347	84616.89647	86445.71409	85034.48857
78065.61583	109286.32	110999.0894	91232.48875	111574.9421	116728.6931	63438.88182
261569.7475	338301.2489	271407.6571	266195.361	182123.6857	215170.3154	234171.4315
132368.0337	119222.6995	130702.0138	73631.15915	121714.9945	60362.64758	82566.58083
283881.1569	281465.8079	298780.9528	472822.5478	364676.4836	314415.6133	310154.4017
307314.5543	317043.7091	253468.7827	410899.768	331574.1088	371012.8419	365244.064
48554.88	61258.56953	48398.24	71504.75557	33158.66988	39006.86667	33679.88433
92507.98928	45012.3625	53328.83733	48503.312	28444.6125	77500.22694	68895.424
146604.7328	124138.0828	143297.9657	313574.3908	198547.6791	145021.4107	134427.1767

412369.4772	487931.3636	413173.2546	502021.2304	376020.566	377208.9164	457871.4725
67614.79695	28717.52567	62584.14388	39049.9245	25686.423	50561.82643	66167.82777
54331.84454	162189.0382	76950.96629	94605.28696	70473.67608	82512.53875	57441.538
236467.0042	243542.1161	229039.629	266715.3918	180927.659	206931.2663	222801.9078
111735.267	131520.8491	77610.58865	77207.0898	81273.71059	91967.43	73788.729
485119.1094	882222.1042	259345.24	286857.818	244834.8728	264474.8182	251569.1373
872123.605	1130160.506	746708.5269	857007.2186	686828.2464	650286.5727	626578.4843
104127.4159	109636.3836	114367.2969	124853.4114	108355.0221	93013.144	98291.6186
36650.5452	40834.71786	26463.42481	29593.08	24665.87673	26173.95344	22764.04976
738231.3885	1238762.256	706679.7475	723327.9672	626648.665	657165.52	651914.2235
2709635.691	3609862.064	2446077.257	2899719.568	2374104.639	2701559.512	2654259.055
11638.09324	12012.27928	11347.70194	15664.556	8610.08975	10112.18242	12420.92929
7962177.32	12106067.24	7723958.192	8146827.315	5615440.907	7077203.783	6731652.785
105651.84	96259.525	103969.7049	163029.9348	116744.1612	110175.6698	121969.68
7866315.393	11342690.47	7826938.295	8285148.591	5622965.507	6721639.237	7028523.794
134902.1695	92816.50554	122963.4303	238706.455	151845.6788	101584.1604	102680.1475
11229.192	25700.12222	8745.810417	14583.64913	9357.947	8680.975416	7661.086302
4926360.622	5298151.462	4730429.617	4709093.098	3426966.991	3823247.128	4100379.625
13002.83182	11423.39392	9809.578182	20405.37231	11614.55333	9196.051292	8286.690546
430725.2518	459390.5541	437427.3314	451425.4162	321626.8	335523.3091	373418.5107
1818932.311	1626582.767	1891650.708	2247075.422	1397203.297	1572724.084	1759647.03
932006.6768	211752.3973	667545.367	696335.6348	445033.1083	329606.4417	175743.1845
192174.5851	241182.2321	120008.67	205901.2947	109938.42	191185.1936	120300.5981
35472.073	53949.65054	33605.32843	43187.34533	34325.96977	41312.87	37839.03151
225125.7266	320969.6833	217993.0967	388857.0227	185718.56	217812.69	226365.5912
10401.25404	13161.4121	12577.35333	23089.05025	13367.83915	18613.40562	17015.88858
5488666.677	5918166.822	7391053.217	8653955.26	6545449.75	7163586.808	8010959.11
4246.984375	4621.822071	3012.683333	4042.317505	2988.325455	2008.89	2187.651123
50656.08322	100081.7391	31881.87333	59004.2931	56188.53917	52527.7677	57411.72
637793.8983	406568.2954	541872.5758	1082962.246	662517.7894	376781.7658	367147.9668
200056.6135	169982.7692	164816.1253	286176.7689	120887.3313	151860.247	149682.9464
91112.66818	194241.4773	211174.368	114033.5785	93960.14091	110860.88	100463.184
10825.8448	9210.609231	9826.096	15816.95117	10550.36082	7703.569802	7655.162476
252685.6462	526659.5745	258008.0698	339839.859	236920.1608	316404.0906	310155.1292
60808.49775	58751.66	55838.02575	82153.03444	48251.7945	50573.06306	54536.43927
4452.892455	3696.037909	19895.9165	29534.70703	5003.68725	22188.40858	20572.99834
2743492.213	4787978.234	2520909.073	2756461.256	2280581.991	2812319.935	2648742.691
8333.954083	10071.68706	9592.747636	15950.09546	10190.91755	8160.130832	7896.55725
9220026.326	11360790.3	9555068.124	9610229.919	7396004.475	8908005.871	9087742.682
2110676.382	3046389.383	2085142.02	2215039.166	1762708.094	2087990.533	2028898.65
2715974.207	2641505.765	2702778.034	4221592.577	2243411.125	2457409.419	2565895.018
76293.33724	54181.20544	54569.65876	86806.72778	57434.75432	56346.98874	52168.06238
17494.23315	15548.56631	11303.30669	66056.43412	52660.25263	64293.97174	47931.3532
24736.49682	24631.64382	23080.94278	38329.6121	10671.45429	22411.47703	15961.7029
1677718.533	1534415.243	1780922.167	1684961.288	1536331.42	1519453.024	1638454.337
132756.3838	89059.73669	150317.3663	151023.0815	100795.989	90768.51231	101474.1723
48454.17981	25962.5925	36694.35143	35115.75711	35834.56041	25780.50439	18095.44
47182.21469	68824.67207	43897.79121	42223.17793	43324.22954	54919.44436	49160.2276

163118.2467	144357.0839	125782.852	154806.9437	156190.8055	144935.1468	130760.0452
53064.65478	13601.45373	46321.09733	23165.58762	16828.61	16940.76	42344.53555
7281.236182	5832.2228	7672.9985	10072.33333	6267.547929	6517.015245	8023.640625
70897.08223	48357.07646	64515.98369	119741.1077	82412.21423	47949.31557	48480.08571
534395.4757	523187.1173	530972.622	634617.7739	420168.5124	439056.2916	477428.1223
56021.006	32094.12986	46291.81336	53410.98929	40360.96286	54522.20613	62505.32385
70972.9898	80860.25823	62263.35409	70019.27164	44800.16688	60315.5857	55092.0601
384165.8192	325714.7492	369944.9638	600156.9736	351969.1948	322550	364042.9026
352717.2846	324167.2174	297321.102	490799.7191	414603.03	441594.399	473935.7199
95295.82096	140046.4352	92978.7414	111088.1739	93531.60655	108347.9513	96718.63019
165972.7564	213299.6131	142372.3778	202211.9538	142842.2966	191245.4024	171223.5409
10501.955	14211.48916	14950.80982	13586.77963	11337.24277	15442.95483	13127.33494
51416.38971	22154.52	56247.84769	76556.45708	57433.81173	56090.38462	72519.53031
132161.3274	168106.5179	177387.4417	160730.0133	99296.00614	100033.3034	129147.0824
191556.2027	137686.5751	157474.7571	180030.94	144564.844	170846.2449	161066.5232
16256.238	11292.51927	17018.7255	17889.39131	3961.584	23222.67777	21285.0889
221523.9614	156776.8922	204406.0945	342206.099	209424.4978	131699.3167	136454.2108
83526.06396	168187.8158	151782.7032	277522.0587	217038.1154	197533.4486	256645.337
18249.42624	20267.69445	21938.22564	17202.73957	16689.792	19724.16379	19383.79638
644946.825	465724.3473	509769	559830.2292	497375.1512	318725.8139	287511.6733
44115.27448	30983.61661	29263.643	36049.57082	28224.46855	33496.15275	22916.73929
69514.929	56836.25383	61639.945	67340.13835	55563.96957	54989.11364	57956.09082
18065.625	28987.27989	29023.4302	38904.09471	25194.17009	21988.833	26399.57483
4321.127502	6681.565735	6931.82975	3947.849101	3275.105313	4910.076736	3060.338823
79801.722	56217.82638	75582.90638	123044.3977	93179.68031	44904.26623	40750.52308
490141.4412	791377.366	471337.682	529935.1866	448368.492	515194.4804	466359.1116
9656.494364	11411.8191	61963.041	77478.78105	5818.485	8114.22	8076.502778
37457.13211	37926.13862	35694.939	48131.60018	32517.72614	31104.9	34658.77958
3633179.645	5443997.573	3796365.676	3886240.686	3073063.887	3752044.002	3870847.117
8066.947354	9042.200824	6855.8994	6982.8512	5731.662417	6762	6900.63975
7614.003	7046.169466	6604.238786	8265.83875	7804.568	8516.074737	7543.722333
5127736.876	6792153.759	5479454.461	4808721.042	4101213.573	6609038.167	4625796.26
1529892.812	2083819.274	1448317.752	1503958.727	1104303.251	1328998.955	1317006.828
9041360.431	9044516.839	9974674.331	9903133.076	8361937.872	10081328.38	9661965.467
969393.1724	1239659.657	873609.7174	964189.1546	769763.5421	837118.5815	839207.2614
145887.6147	114604.2516	132890.3546	115485.4395	97865.43121	66250.68908	63218.23686
471359.1654	480123.1661	442796.07	425935.8264	358070.948	373601.7321	488877.5047
1166604.02	1164101.888	1139175.09	1197663.443	1006019.44	1026897.095	1047712.278
17689.60375	6788.373743	19096.37217	28438.88667	23346.90891	13249.1216	17388.588
15521.85785	14347.31363	21421.33067	25674.11926	21565.90617	26585.85512	21856.32692
15972.26567	22989.7752	16690.61689	11028.07027	8637.968004	10210.1688	8102.379015
216986.7452	156799.7308	157799.7543	149694.16	150028.483	101334.2288	100126.4
132264.9067	161087.9016	132585.7848	81222.42554	119473.824	134641.4902	123188.3981
110591.8972	122599.2308	85777.3179	241361.5492	205261.0064	206382.3391	192546.3717
166244.6657	166399.5784	157775.064	214210.8	156814.3228	196926.9709	219046.1186
118075.3877	121179.4948	104804.3923	139735.4338	101117.5499	98656.1985	109580.1611
233139.4563	170106.1796	199931.7069	242087.1768	178989.9692	138599.3519	155207.5037
39195.04154	44458.167	38654.39913	34609.17115	35520.16621	39800.52133	41038.63264

1155038.52	1022122.69	802270.48	1213234.808	1101905.034	1060501.044	907680.3028
193870.7982	223290.225	189262.9477	265795.1955	174537.243	186004.8823	196078.2518
194053.0558	167737.371	201525.1957	265471.8266	163697.3634	153888.4112	184046.7062
11202.00323	10970.444	9757.236364	12467.50267	9005.061	12491.69645	9071.636364
60590.27087	50368.64444	49821.192	69145.12	68607.877	57927.85081	164229.7145
45909.24292	26034.735	80092.78985	57991.84462	38430.26685	36146.00562	41793.49985
26276.4492	28331.25656	28274.8578	24544.81615	18189.47871	18270.46735	21585.94172
25248.18542	21128.75013	26349.49121	12879.92	28208.42684	10324.3425	10133.81202
70091.70353	97030.80765	35340.25	32688.96321	24195.8938	26094.57788	25257.5734
48288.98147	37621.7625	42475.0384	65429.72871	48745.66687	48857.79868	57148.72093
176898.9263	180067.641	154871.4692	181554.27	154064.1173	149297.0026	169202.1538
207519.3473	153218.7647	163804.6486	186904.1452	160497.9623	96667.6032	87263.1051
39307.64915	31215.24847	35517.50187	28578.09814	27653.385	28088.68526	28484.03055
4482442.065	3749713.004	4259993.276	4773575.355	3659412.714	3689246.818	5735683.966
12877.592	4970.933182	10797.21727	16766.17286	12336.29358	10000.83213	14223.36121
1576877.845	1184541.945	1347619.294	1381801.55	1273811.994	681589.761	689174.6601
248985.6313	246967.5787	202834.7226	251426.332	233166.3259	173472.6862	205837.8672
412474.4049	329011.9858	363799.1379	495069.0277	309607.2349	317094.0889	454028.3631
618192.1525	861301.3841	551634.2436	619508.3535	497920.4063	570483.8745	531887.157
247556.8935	246837.5849	184144.3869	218063.3597	188948.7949	250408.0945	210646.53
66910.3984	47725.59461	58720.95668	64298.77444	41764.93143	24675.168	32357.4009
15669.28943	508.3066406	5854.64	11133.03038	6159.071	1782.88	3555.490909
76718.65223	64937.21036	69685.38444	66911.83519	54870.95865	61166.80885	55373.47522
11425.49563	9650.293625	9477.089923	16604.65508	12547.06667	12083.63927	13238.61311
106717.745	92451.68318	110332.2851	79913.73375	162561.6795	53769.73286	67643.1293
340137.5743	334885.7793	297225.9695	297721.26	289159.4954	312032.5667	257671.7446
299519.1436	282508.1733	291156.741	404219.85	256600.3096	270368.9246	295440.8619
1187029.34	432155.7408	814424.2768	1201912.44	906428.8292	1083003.003	1265923.835
1602419.08	1466169.382	1507288.12	1419990.065	1199320.852	1205474.146	1239299.063
1110681.478	1028609.453	1031459.647	1035523.406	836891.669	1421120.564	960099.854
4968.688515	2756.744792	4688.719	5585.416	5135.578636	4093.056	3833.977844
224947.5263	172348.0843	174308.4476	266570.6357	181931.7692	287759.8623	234823.3335
1966473.576	1769438.224	1858626.605	1952628.743	1681335.142	1393871.902	1588094.815
59785.32623	71109.14645	52097.95	53476.05927	71691.3	61610.65229	56540.51667
2914707.53	2971801.477	2389868.265	42774.14375	2567829.385	2907105.768	3063867.945
104829.1944	99776.9918	91193.33113	119371.1756	93576.77136	127991.5064	108731.4221
53464.05831	36570.35908	55162.65557	58131.51	56798.14631	58825.34131	55393.48031
102497.2899	109897.1028	192744.5652	154709.6078	114387.1263	177632.4361	132396.3244
212250.8625	244963.9449	197774.2431	256974.378	209812.4658	240846.0766	280624.6917
2831687.74	1897360.724	2008823.673	3229160.008	2581703.839	1986122.544	1966949.771
89083.6	72505.57336	102654.138	96135.952	72382.646	76532.03643	119261.9155
50742.40575	47577.436	48757.61224	54067.91138	50657.668	52494.4002	62886.57426
35386.59333	28575.79152	19893.17667	28158.99445	25186.33366	40217.87333	38490.96203
478248.2014	392434.4827	583922.0645	405949.2316	752542.7	461348.6149	427036.5513
483261.8883	473980.7453	422561.0414	563044.7921	413965.7936	479250.7405	769599.9196
12609.60783	16677.80228	12754.7	20588.04279	21150.30198	24265.99658	16430.54933
1974629.644	1488194.967	1966160.528	1793215.904	1791698.94	810615.9696	823855.5929
213198.5361	219372.447	198940.7877	232294.1154	189184.3102	194569.0334	173868.4797

56416.60817	60028.50883	58297.03615	51396.45938	57694.982	60682.25385	50254.36131
2098866.884	3115170.96	2129925.477	2225650.846	1578968.166	1896084.584	1968374.964
390629.0241	309711.7456	333936.7193	480883.8348	310642.8058	321425.6346	534594.944
856133.39	820407.3519	891340.3651	1362365.286	1079247.957	1101279.883	1068775.471
40767261.41	32567838.04	35866269.82	36415035.54	34811846.73	26081826.82	26782498.45
471688.0761	559280.7942	634149.0386	749601.424	519198.752	337307.5747	971180.2776
1145818.155	970769.0987	898000.4301	941185.9804	766759.7071	1538287.296	992073.5023
41024.85216	36547.41817	37761.57064	38269.08924	45228.6293	33972.12283	41773.24542
221334.1171	128293.9376	120190.8089	183521.4281	149675.8648	183323.1756	226436.8808
2547394.576	1929420.335	2147716.617	2243006.35	1929916.759	1621087.52	1916885.215
40033.1815	61485.0057	53918.62588	77792.02473	72771.68444	37267.26754	26196.44143
20255.73888	11129.53991	16028.90857	25885.22653	17873.88429	13803.95861	17440.21
561294.5492	579451.131	636358.9292	810750.115	748924.4277	444887.3353	504253.53
484663.1156	441229.9761	423726.5767	492726.3759	480559.8931	449749.2166	462223.0151
244245.5503	256527.7721	197464.4949	282625.9282	216745.3484	221904.9986	221252.7204
86926.80375	69441.76892	60628.326	112591.3729	57458.59887	100147.9169	82634.12806
1076778.894	925701.2568	636760.7342	675040.9837	565403.6373	395335.1508	415715.6787
76635.2394	62486.06522	58100.19614	73966.0984	77076.34159	72614.43663	64429.85616
1911102.861	1674103.84	2800377.936	1701394.932	3065467.607	1492513.538	1486383.7
4860874.045	4075436.894	4718971.032	4233046.014	3396692.652	3577162.814	4104634.77
4623242.263	4703655.71	4029640.774	4397575.041	4037889.602	4624536.429	4793062.617
24148468.01	19236808.24	23520581.77	22337908.46	20778087.68	17880258.19	23307412.44
261002.588	186671.9165	242965.5658	251889.6083	268925.9317	173267.8833	236209.304
294250.2247	303921.4822	514095.2351	245437.7318	210011.562	127599.9155	150818.0402
78163.8766	75649.14052	69211.056	71403.83932	63951.89286	46774.29988	79860.38949
62884.06534	49978.58296	9960.2343	43611.28499	31002.83956	29718.28889	22434.10517
7173951.123	5156041.892	8315706.58	6523235.074	6063767.381	3071032.926	2866876.766
1518615.03	1338344.75	1901125.963	1483521.173	1214442.895	701797.2994	763036.4744
292913.2626	267417.5008	245983.6957	281263.8883	247723.6509	209603.3237	251350.4904
4206.746556	5189.950562	6338.525	8930.430672	5719.534687	5940.42881	4799.149333
10389199.34	9422112.048	12467578.44	11255381.5	9734075.775	10740269.54	7825630.239
30526704.92	25404024.15	31553399.75	28858365.33	27958872.46	31234612.04	23984934.07
156197.6338	145129.5166	152761.9256	135971.5844	130382.5511	208443.1619	127582.0904
303896.4731	236973.6801	278865.785	351667.2233	239948.9452	236305.138	359758.4951
313700.1849	223482.4699	253862.6657	341236.0343	229216.8093	225192.9925	437915.3311
23534.30141	19797.96	16513.14625	24116.65846	14554.2565	27891.044	16448.64
99188.39418	6375.46	11937.19455	110834.2133	83458.26096	8793.98	6139.4515
535194.7052	498791.6716	583122.5745	686855.0599	525565.4	757715.2	875132.619
118560.8741	88474.50554	109721.0512	113242.4769	130133.8228	60007.08408	84826.52169
51071.48119	56063.36697	46964.52078	58576.07558	48764.62484	47054.41494	48682.5134
73739.63629	365947.0968	1744314.342	412725.6751	1346430.936	1640927.182	356975.6039
39957.98936	31954.56523	30964.62757	41282.75391	26774.1326	24296.31812	33005.57693
11776085.12	9512599.789	11225653.65	11967259.17	10076241.48	8117248.217	9338869.884
5733960.017	6718769.757	579243.6573	665220.8261	5649044.221	5813253.089	5529415.624
161084.1251	152887.5203	190638.7569	263106.4324	189920.6892	273628.4653	214476.5396
102087.9348	129713.3515	92764.85007	70600.9738	65010.00533	69083.46387	56694.3804
2019534.593	1863821.805	1933729.146	2182484.456	1886296.93	1892756.393	1942831.141
8183845.672	6366833.626	548985.6527	7680314.972	6602104.59	6336938.517	7166866.435

131299.4605	119855.6876	141395.8376	100026.5719	139544.5066	115828.0942	120234.9734
8366990.28	6497511.677	7710228.102	7894773.782	6554771.997	6399616.165	7376641.267
462056.712	1271380.352	1121783.617	1012821.58	1011032.035	1228080.402	2010192.954
2195947.665	2053962.296	2170877.172	2333361.611	2098150.661	2239492.996	2382747.02
20683.48828	8704.576019	18235.97587	17610.80057	17726.9624	4837.021648	8634.731792
76084.08068	69249.74732	69702.04707	99329.13564	65442.50158	151566.0002	74652.9797
4826148.939	4096854.429	5052075.377	6909061.49	6818877.48	5879807.781	6722333.942
1022933.188	1309042.634	882291.8461	1295670.392	1004798.077	1435529.607	1416716.324
23985.83818	28211.13636	21263.075	25928.8188	19738.053	19001.28	20289.4296
19170873.62	17409919.81	20598219.26	18739518.04	17867015.52	16286559.05	18440223.09
1008297.703	1066045.203	1021900.346	1533826.904	1092407.4	1182439.565	1860292.097
15294.87887	14081.83022	14122.22867	13606.11	18337.33824	15182.93443	12629.80364
106695.2965	92251.52779	122414.392	96044.79888	96613.19684	62928.19905	65344.49084
4632827.387	4842679.32	4106463.656	5293283.94	3827645.952	4209942.493	4657162.587
5052913.869	3455997.475	4600358.485	9009262.633	5230728.702	4751241.198	11404075.18
73976.71636	7366.187012	31250.96336	27349.086	20009.51622	4480.3462	6938.68441
973825.9438	802341.0554	1032732.378	1058663.46	1292558.217	492178.8452	771029.2074
14784170.2	12324948	14664066.13	14960872.84	12251068.4	12168162.9	13636835.83
661510.0189	1102618.114	813832.962	815432.1841	677823.5554	906675.2457	934408.3461
342392.037	365242.4773	323252.7123	293111.4776	251806.9606	270599.4072	275721.2537
678042.0125	529153.9644	652575.8484	1106788.558	648405.2244	587267.0252	1146497.985
811301.8032	741176.1234	920321.2214	876771.875	795849.1781	722224.4704	813390.0058
17741056.51	16382461.95	25684802.71	18271820.98	16867803.42	15277222.8	11518224.85
57419.685	23597.402	51738.34358	75868.60769	65811.54865	23214.8315	31826.769
381625.6098	442769.7395	347767.9045	551872.6242	451321.0278	376965.2018	409584.7062
497045.6851	279945.8833	245886.278	720900.8867	290921.9021	458817.2606	602331.2434
14205003.7	16312534.95	14318663.89	16905063.64	14770139.59	15908036.07	15548453.68
179212.8318	153402.5313	139519.7508	208312.0856	146339.2913	108311.5302	204982.4063
308985.399	255920.5416	274140.0346	221095.0246	193714.1461	206819.4193	241453.96
1464.947111	1607.111111	1588.909	1793.912308	1370.720545	2138.794861	731.64
281065.8697	244144.8656	265816.32	333488.4444	240812.118	153806.8833	217961.3987
169041.5255	191299.4274	202678.1242	290293.9471	232825.0526	157877.8688	315457.602
8355747.514	8206680.406	9390248.51	14419511.52	9600544.749	10972463.66	10869887.13
681337.6264	621058.6523	808462.2916	777758.6477	630556.7769	501381.6305	598939.0688
99747.77888	233004.5887	101419.7473	67496.54468	63858.1412	76236.27734	61734.9448
14371142.42	12303660.29	18888537.35	14385833.63	13188679.86	12020092.23	15286781.14
45851.0377	45491.23016	39947.29133	48635.58218	38841.34409	35532.67588	38372.77568
267278.6937	485980.5679	278567.6448	159873.2367	156987.2977	177438.4934	131793.396
308973.4257	559993.957	267998.9114	165877.3609	197995.2438	176775.6691	138094.35
11690975.33	9092563.323	13606740.4	10806292.46	10320047.23	9285509.385	11527942.23
678016.96	620293.7191	229050.437	274566.0614	862325.376	543163.7431	643643.6322
11696800.39	9068622.667	13595695.97	10825078.39	10294408.38	9171404.822	11549248.16
175725.355	170787.6497	153340.374	135188.4702	126910.4342	153482.2416	112169.0487
23581.70013	11920.11372	21618.11082	106384.0352	78841.14	115491.792	112860.2499
2510.498801	1132.044983	684.8171176	12171.64467	10636.78819	10426.13738	12396.92412
363040.6142	395370.3158	475065.4746	490875.8364	380190.4333	426471.5417	482512.61
2507066.62	2621048.222	2899676.217	2746320.345	2325623.983	2244494.171	2342488.534
25154537.41	24439898.34	30069204.82	24955223.88	23441999.89	23603819.19	26358280.94

2593159.6	2611901.552	2170954.86	2898682.546	2811465.701	2314202.369	2628707.84
466804.0471	529240.6371	566451.6701	523491.8023	446547.0941	427867.0989	467398.2368
107971.3212	113209.7666	119898.7145	122117.025	75213.045	62262.49615	85205.227
1830493.413	1803030.061	2111318.177	2050996.455	1635559.2	1622106.914	1891953.324
623347.6802	523687.2842	1406756.569	368427.818	1401973.142	1311761.252	677077.8845
1775037.19	1214227.68	1904110.942	1421074.924	1056118.23	1152888.33	1288732.861
6939863.597	6600722.038	5582762.729	7483450.48	6835422.846	4943115.778	5178104.709
177602.0586	141095.5148	152739.7886	261829.8233	327983.9512	244630.8232	277810.1175
767884.525	554503.3372	741253.9376	883732.6077	502829.145	642628.3907	791168.3862
609930.1389	655984.3241	451640.4325	842717.8609	1304312.54	594175.1359	640917.4359
554397.1785	534954.5626	554917.2015	824056.389	741517.1768	584208.5955	592262.8761
36830.81978	41739.20736	37936.4275	52100.6155	35731.53691	40942.47005	51777.52023
1341495.867	1525997.888	1203854.468	951828.871	846609.2848	882965.5399	754289.1415
44692.6692	39692.75131	55927.09371	52279.55827	38833.62888	29163.90913	44197.79659
41695.512	39124.777	36435.841	48950.97783	46528.69475	38313.0285	40144.02923
1233323.232	1145800.595	1147890.283	1290836.541	971064.8012	908426.4307	935183.6878
75534.36377	67215.67669	77196.04908	124139.5827	63937.54667	62493.96258	126048.7926
3670129.691	3112610.37	3857580.465	4045662.946	3294822.283	2993652.075	3275805.658
73029.11383	127890.083	66830.37327	45839.20195	43990.86674	40751.64836	39967.26118
12543.6725	9630.605	14069.28925	14813.69127	13102.33846	14677.725	11153.75525
339047.8058	375333.5011	295092.273	321754.6438	345367.1411	417964.6823	327244.0378
12395254.08	10047474.69	11387123.14	14896240.78	12716354.89	7306810.074	8062936.823
87761.58442	110639.1162	174178.057	1063593.655	1080827.567	1162781.845	1092907.179
1678831.701	1810577.124	1926775.751	2269233.715	1883930.465	1560054.567	1896464.061
15326.01167	15760.47	16338.26923	17404.47836	18445.46331	14570.14154	16706.09431
133704.3447	156639.5339	123986.1907	701094.24	226460.96	867315.5841	228060.4876
361012.478	305445.972	390925.7423	345439.915	271792.8274	278508.96	330829.1666
91285.32794	82835.98256	84127.7745	84572.43862	67840.479	93347.57529	94297.51692
1479974.007	1795020.336	1526918.347	1695358.37	1616273.166	1498088.421	1337118.581
365187.8362	467077.1762	345814.3056	277374.8565	248885.1498	272203.6018	240518.9448
7313831.571	7589752.705	7258243.173	8437514.495	7729574.831	7043549.639	7186486.688
5871.629824	6617.67598	5916.016909	6140.098462	5735.655762	3843.027364	3388.581818
2082252.009	2495058.208	2654432.669	2849888.173	2182312.462	1728987.128	2292004.602
15344385.74	12355131.45	17091568.05	17418083.59	16154694.1	12538032.95	15893787.84
2865524.43	3496165.801	3095801.364	3584596.45	4009369.616	3395987.74	3215361.656
700072.9567	617979.3671	675049.69	951499.2707	636641.5686	654377.0383	1057912.807
4232.904053	3246.656372	3519.307917	4958.314148	2489.718	2758.060776	2161.405838
1304603.599	1186781.4	1253992.532	1516652.916	1036210.785	1153557.383	1318418.276
18692256.28	17440655.23	15565383.63	14539374.18	13743668.24	15473521.23	13495208.28
604282.5735	537668.4001	570998.7712	633461.7471	506014.3659	500483.9268	616157.8658
395442.7255	588082.0454	438019.5848	596735.36	467323.9609	580150.1813	638318.6232
672945.9402	1508229.666	872793.3528	476809.3662	428808.6756	581393.0751	395027.369
1231360.744	1033051.327	1348952.895	1748246.707	1118042.612	406202.0737	1387584.028
540098.1991	796617.3626	590071.6249	669304.3066	659555.6828	842878.188	748921.0006
1473835.57	1285275.369	1370369.212	1493413.174	1250306.346	1031005.844	1170106.972
5789159.359	8391546.554	5772507.432	8089299.055	5819689.946	7219456.421	7187310.335
413751.139	381679.4802	578196.2476	484783.3517	361324.1792	332237.2146	423325.2756
22250.94679	19739.30997	23822.7647	22489.5	25373.6631	23221.53644	28237.86

3220298.117	4517782.152	3190499.08	2072423.323	1848676.856	2151893.993	1793352.392
129476.3154	123868.6449	129911.0878	130791.2766	122585.0041	88544.86818	116662.3176
6785586.616	7813343.86	7774473.176	9265035.01	7218265.697	6733115.011	7093691.92
34309.50234	46941.53544	64874.664	80849.74738	57881.89425	105511.2006	89662.82266
62905.31101	57574.1412	87529.76128	74750.35596	49196.2709	48628.14225	61188.82052
9609403	11728995.33	8335340.117	5857442.173	5446000.246	5866079.852	5130283.711
104379.3773	79013.23125	132875.7733	135948.5317	118535.13	53882.01	55142.02725
1685360.277	1189471.58	984710.8762	1358808.255	1058255.769	928682.2271	1010987.188
244187.5261	275454.4979	230192.7202	356135.3221	297358.1467	278078.2098	353702.1741
71112.55194	75419.97246	74757.384	106104.8508	81473.75905	74213.77186	100135.6917
84682.85596	81972.9188	83838.64154	88097.80015	60313.16528	53339.49257	81238.65385
90703.92795	67791.525	90182.37517	188419.2721	74485.33333	76614.64115	152025.366
61488.28136	128735.5067	51810.45844	57959.51205	38113.66957	41794.88458	32798.71918
43018.19407	36528.25378	45182.32738	38381.66253	38913.17906	33526.82571	38238.14938
3127544.418	3794358.023	2724141.309	2336264.057	2034417.749	2323827.653	1891036.855
197803.1924	395835.5483	182464.2538	119867.6697	111772.5307	147112.7306	110108.9089
78907.90708	68541.83886	78588.54769	73197.91121	69688.02462	66246.19231	66974.08414
497473.7727	475003.8687	438636.4079	471173.8034	295630.3536	288639.5526	299951.4328
446990.8745	347502.9619	439801.4029	590003.8042	312255.1456	356136.4458	500358.9655
399627.5699	646331.169	373228.121	262073.0618	248931.4924	281020.4015	237903.7476
71463.4395	116564.3367	76599.90337	58560.53595	48561.67057	59117.79932	46087.59736
76283.96371	101663.3733	73980.06	168312.6789	108186.0785	94733.58352	95142.78953
471061.3481	707373.4602	447350.2333	324299.136	298250.1897	451148.1712	312179.082
12220.76769	60407.91875	50296.29462	49735.28108	35198.13688	41720.4362	34895.81611
27739.33264	29755.83043	75407.036	49980.87684	42889.40602	63796.56586	17408.17714
105927.6874	72679.07631	113369.7748	63703.02449	80374.29246	80710.79529	77684.77385
40529.30718	57591.19383	65315.90588	49292.40491	44588.26103	64365.06119	68688.36325
413406.1672	296320.9829	434782.2867	851970.1194	540317.0802	434036.226	1118357.548
20776.37243	19664.15313	15792.8465	16963.69495	14812.69769	14905.4505	13508.04289
316425.7793	236274.6301	334896.2996	521328.4173	386596.5379	346886.4867	633192.6929
3004800.295	2792735.84	2904527.187	3049195.241	3047719.988	3149872.206	3004284.703
2877857.12	3343086.397	6843265.063	3757476.875	3412801.481	3126785.121	3213965.665
4661.055199	1908.088664	3382.426667	1718.371582	5719.625862	3180.425578	3031.734154
15419.44466	13685.90481	12919.47035	12888.34913	10368.00494	9414.57943	8296.07666
245334.0085	148201.5245	288394.8242	306508.4922	251406.6184	214132.8148	284707.2928
40302.84836	88376.65924	36221.10738	28346.10974	26350.78358	31542.30969	23791.98804
817781.0492	784997.0886	687594.3106	706500.6461	734440.1024	614391.2566	681161.2955
309508.0024	327341.755	444580.5723	260388.807	255671.3292	462689.1454	530674.32
547545.1366	851518.9421	697376.4492	697593.1963	785780.094	647188.8317	784919.1276
234849.5211	241767.624	320165.1635	323567.9955	335475.2468	243232.2649	278594.1274
4233.468429	3939.390813	2874.054	5049.854437	2169.421941	2334.748571	956.0375
1744400.755	1941008.284	2702602.881	2739521.72	2408960.973	1819949.236	2278672.621
12314.26282	13647.40901	10941.17527	9692.946555	7967.801813	9931.942017	8411.763361
111249.6	116512.8195	112097.6246	133667.6518	136685.379	113746.1062	118753.1762
53654.29504	59851.9274	75973.91279	60125.33947	54300.43344	77331.18113	84207.47568
43052.4052	43620.30265	40212.51671	41449.79165	39918.04235	39467.93667	39319.56933
5429256.068	9901215.15	6936178.455	4870035.18	3334326.379	3966424.624	3539304.249
27509.11947	68940.57922	42098.24675	187067.8561	129458.1573	146307.3777	175136.8532

77572.42566	113509.8492	95684.45	92435.77319	83836.3726	95853.17083	100454.2632
64772.68057	92352.76005	75836.46756	62562.19508	50498.91451	57565.84951	65079.35937
201315.4776	188702.5248	240319.9874	265040.6995	186019.8231	192801.2243	241486.7098
57648.92902	150798.186	48290.66583	38288.66602	32341.8665	43897.99745	33557.48683
66850.61236	64957.20817	68572.05709	74629.395	65743.56218	75529.57217	60824.4525
34903909.84	42836444.93	30246756.39	22458643.78	19616298.86	21947144.7	18830619.8
622831.865	609158.1871	624151.1354	601665.489	666581.7565	715331.4285	713197.16
48316.52421	37222.62913	51641.52973	79892.565	58664.03923	48953.29957	105749.4465
99617.20015	181224.1454	90965.10192	63938.9285	60260.84743	78281.88607	61551.67847
78134.598	189986.4431	88841.36791	50732.36143	48298.44725	82950.7965	47729.1072
360950.1915	757817.2747	346195.9152	244102.3423	220379.9109	303748.0848	223094.5775
63239.48623	74527.24756	82998.73232	113669.8164	115870.048	101745.9531	123925.7712
346992.1423	293123.0608	313565.4523	303613.954	275157.811	285780.558	267140.2114
53850.88542	58639.944	35669.98455	29610.84364	52685.43513	48270.64925	45314.94452
105748.276	126213.4999	184939.9248	103147.6175	97330.67279	160129.3372	190908.5083
277866.5142	252855.8306	270957.8332	246193.4286	244915.6486	248223.799	233581.332
22695.90021	21426.42979	23155.00708	22029.06718	19918.56901	17468.09387	13321.38378
4612184.111	4413538.273	4600667.873	4357377.248	4444310.964	4709374.376	4969246.633
51047.03827	165010.4429	52118.235	22128.40674	22104.59534	45734.25251	14946.02869
57544.5594	77273.70288	56482.2888	40398.96326	39377.26406	47944.51493	39952.2609
321691.8112	456662.1407	294490.1212	203625.8773	202816.5418	226625.7672	213794.3394
115558.1841	308691.5086	95633.69889	75643.37731	58399.90276	73354.56822	49027.00478
18018.24503	15609.25911	16507.49518	14959.83447	12692.44263	13467.4528	12139.59694
18804.26571	13154.8323	16495.101	15122.72264	14001.30713	14946.183	11455.0751
13934.47507	8520.460693	13771.55312	17361.80876	13556.04218	12100.00989	22532.03125
53140.13945	114956.5857	13155.13671	20686.64083	34052.30439	36129.86624	51628.269
20793.01265	21046.88831	18026.14073	17786.36379	12172.54093	12772.31188	9728.885986
16973.16169	13352.29608	14339.91193	19651.89916	9529.157185	25497.8677	13701.11963
252803.5266	262472.9525	264442.4129	266024.5135	274305.9056	236949.3537	255709.3466
267723.5749	505678.5493	234310.2918	164360.28	156808.9886	194462.0744	139408.8548
10384.80226	6988.676786	8241.7412	7253.335815	4605.592765	4399.328357	3759.865417
183770.2856	262195.1493	149241.2086	114500.6097	112688.8956	129289.2061	103291.213
24069.0505	21969.17754	19600.521	21332.13693	10244.37033	10032.3137	16959.88183
53497.52155	103715.6042	49087.1934	35900.46816	31820.095	42675.09111	33471.86142
137502.3366	143330.3182	140525.8347	96028.9203	129638.6675	150038.7379	138651.0418
16600.69995	22139.13107	22177.8918	31470.93867	17324.77693	18169.75018	39709.30847
27142.78852	29399.17495	17430.93978	36313.42367	20907.8023	15327.74571	46450.818
22264501.48	33466204.64	20621634.62	14648904.84	12749339.64	16482508.87	13328894.91
185020.8815	217418.3789	186461.9562	225173.2526	181397.3723	185717.2848	234493.1554
87817.97996	162761.9565	76068.38889	104661.4908	55545.00841	78127.92874	107208.3463
13567.70183	32006.52043	12070.245	9365.550528	7400.970616	11445.54339	9709.749268
184000.8556	281949.3163	155452.45	137951.462	145731.8455	173836.0856	146655.1475
50012.688	31253.8974	72132.01029	39389.22	32598.45782	24209.58964	33049.91979
177786.8865	166428.24	177837.6075	177643.644	186947.2689	173723.0878	180553.8469
329021.7125	637287.5919	274177.044	210964.019	195725.2642	279233.1969	191865.4318
63885.1185	90985.32466	78352.89894	120426.1195	68180.53875	58904.21333	59203.0526
463543.4568	360287.5157	398723.9165	393339.5105	372742.4509	402155.9333	369685.7821
63655.51069	74602.64435	92340.25306	58996.00951	56574.13079	89263.14447	97037.38844

164.3847656	6675.377734	12205.8756	22123.87439	21888.66	17590.08185	17679.88257
99982.38423	290189.1451	139007.7548	99765.77953	72482.33262	47937.07133	102208.6826
12701.0907	26221.95239	11077.472	8115.310669	7207.641403	8098.53717	8022.52417
10220.86121	23724.67671	8942.424	9954.257813	7621.11969	8556.037402	6954.139307
3945.417446	23870.02377	8591.97925	4829.412346	5293.961792	9369.777908	7048.25013
47501.79029	58061.58404	33608.145	12331.7586	14852.435	14493.96825	15543.9828
61569.46171	191689.4183	35684.97818	38812.35022	34991.67737	46956.9281	34334.80235
221619.9838	306745.6291	210398.5598	169583.2485	154973.4531	419694.2342	175774.8983
195082.3193	259047.0486	177339.7544	135103.1498	127460.2863	159405.0725	125772.3949
34104.392	42892.24122	58994.925	23790.15333	22478.901	26568.87086	23138.91273
274936.3476	804247.0241	231586.6077	181637.7761	142536.8501	182024.8541	136006.2522
249333.2916	267810.3498	219002.1833	152719.8566	165590.4183	191499.8913	179425.2077
370226.0695	83648.107	57813.62787	184957.5024	247234.881	284981.8509	197574.7131
16017.23532	15066.82905	13255.34244	16572.92663	15117.70488	13950.12776	11832.51508
409299.6399	1067537.733	353596.2694	241124.5037	210817.7238	278228.1248	190531.5744
6097.393117	6503.51825	6137.704143	5276.541452	3284.352458	4200.767125	2946.856038
30970.55	42612.30093	118536.8503	36362.72443	102195.5212	47142.07706	41830.48242
5880.302124	6567.86673	5533.92125	4755.939067	3365.4985	3294.315706	2663.884969
34066.9826	44605.75232	41048.75533	42329.45646	54955.87328	52265.2085	74620.59653
267422.7351	400378.2697	219396.6304	163247.0416	168034.9513	179925.312	149708.2749
2475.69025	1512.814273	1761.36	2104.84575	857.12	1164.93	1409.129054
58967.64145	130858.305	33646.9441	33151.26091	38824.87821	53266.97964	28362.98036
13294.86011	29613.59415	13950.97118	8473.839422	7761.953888	9544.249828	6011.505615
27921408.39	40050628.28	24601175.35	18152737.26	18402119.03	21910388.39	18847136.56
472289.9569	653414.5978	683325.0012	472340.0647	648910.2766	521099.2671	462230.038
216478.1908	250106.3489	215995.6038	187594.5921	219790.8795	283054.7864	177242.2359
326141.97	352448.3812	355172.7135	319014.8479	299099.0375	317969.6591	321579.7335
19670.44439	22824.16442	32239.23161	22033.70625	20660.13254	31970.30467	33500.84106
209039.5525	285336.1977	205956.1275	250374.0974	153137.5494	173021.9679	249857.3337
11133.33245	8855.879013	10715.12264	13484.46655	9696.084872	10958.15422	14593.26738
122211.0708	227074.8562	105401.1828	79867.51538	74486.21969	102216.1225	77030.75169
86253.93341	75693.64972	92319.822	67774.22344	71719.55884	63180.05078	61050.6178
27193.24636	39542.43462	22550.415	16328.8589	22066.8	28512	28821.27733
114442.2181	398554.4325	134191.305	86757.39536	70247.1528	102418.4529	66869.603
336982.2677	423560.1151	300340.1965	221398.6867	218485.3537	279652.626	223091.3843
58251.29178	49016.86425	135967.5667	46787.09412	39384.0683	48593.4021	40881.3032
47337.81218	136381.1739	29369.01767	35012.12382	17961.78078	44000.4646	29371.8693
46770.7047	143224.7874	48605.98154	38779.26341	29378.26556	41631.97006	33019.35523
186949.556	449985.1146	147165.9647	123061.2493	102429.534	130810.9482	95405.24934
2724.617416	1186.117889	2217.829333	4350.555	2302.965	2296.137455	4631.559
94764.04898	125637.8715	82470.28444	66616.23883	61530.44568	66640.14146	53782.42739
49239.7036	58778.067	47506.04433	44935.28667	41948.9525	43093.0394	41216.82273
209961.4526	319708.4741	176647.136	146394.9862	146690.3011	164912.1748	127951.2884
38256120.36	53310767	27130834.09	19501544.74	19042718.64	25300396.42	20049767.61
296496.2736	433424.0179	264373.541	183576.0557	160175.5913	233017.9388	213039.4724
14612.11466	26527.00461	11436.22246	7466.431554	7435.822083	9904.064378	4272.56042
343535.6884	808256.5359	461897.6656	261873.013	468846.4795	652724.8533	186854.9226
15404362.49	22213951.3	14198557.78	10248675.98	8690702.715	22592668.46	9225504.247

217357.6463	240087.6792	303571.8543	481574.5514	391524.9066	319726.979	287161.8543
694611.5055	431007.4412	422099.5447	636697.1444	421104.2344	389382.4128	411713.7167
514860.0863	678097.5557	681917.9815	1074213.515	997135.4166	1015678.29	885887.9154
156514.0688	232065.322	165964.8381	131387.5423	101151.915	134682.0433	133595.0874
130618.5296	487142.2887	131049.724	80335.51077	109263.3401	178421.0574	93320.715
132088.4869	539602.3529	104651.4073	318568.7799	81408.60325	354644.8463	103584.615
83472.88403	111510.463	56536.849	70772.77979	78346.09293	89573.94053	68516.85821
34386.14627	69548.41715	34045.89375	24040.61426	20438.59859	26626.68938	23218.05194
211469.111	393916.6014	178574.1097	149946.6101	153892.5084	194645.7217	149532.228
43065.82967	117040.6013	40507.54926	41370.00342	34386.62019	32988.0202	31349.68051
110297.1655	127425.0878	93530.99533	59240.45502	67856.88336	72418.2455	75580.55054
214329.37	202619.9629	203465.0866	181958.1798	161875.6163	158275.7742	143536.2992
178872.4783	581274.1799	145253.2921	127909.0291	105031.5521	137703.7093	101636.7657
41608.67175	46601.19249	49731.75245	42744.40368	42416.05074	44642.65229	35314.1088
306833.3444	703979.8462	274435.6769	239744.7255	219235.8716	241360.5501	316419.4299
64047.45996	86345.84163	55205.1369	53105.69432	44633.29518	65958.18202	56066.48573
66286.8276	94361.40101	58263.96727	55585.39449	56717.27056	53850.75183	45416.57813
11680.55546	11191.38031	9519.174429	5785.219273	6358.726051	6069.750279	4562.126923
39703.78726	49513.24666	35989.76291	33443.17463	31317.2639	36099.57305	35225.72338
173675.8057	231390.9468	151069.4874	138363.9469	121414.8039	161880.9628	124221.3508
16997.76617	20804.31843	16473	12032.33636	18222.56374	13892.11278	10505.77854
42699.38669	48966.17527	36658.66392	32620.7381	33703.81219	36135.55504	34856.8433
4182.904053	5162.474013	3689.07825	3125.829798	2847.59421	2441.11829	2710.73933
26280.26417	40355.02649	21652.70195	20037.79711	18479.13476	19549.42005	17711.34906
6762.672327	2520.65719	4766.180476	4039.053955	3632.311578	6585.280334	5139.462312
41799.35196	40782.41414	40222.45714	39975.39063	42397.54353	40999.46244	38562.11369
8019.693277	11268.68223	6179.283929	5015.539876	3451.609087	4252.245928	3755.208252
674908.7615	357523.542	830126.9441	571819.0355	490776.5224	288911.7287	619838.9663
17050.89611	12794.256	16199.19012	14618.64476	12490.08739	7050.219222	11252.33825
241658.7847	138029.067	283432.776	247606.4628	199358.3509	106590.4102	186767.0432
157864.0935	112018.1828	165050.8461	172449.2799	114943.1268	84682.24	141699.6372
25292.58114	22927.21221	20743.9644	25308.9225	18225.72267	14031.24932	15691.833
36318.2292	18190.10905	42017.34439	32735.016	25844.45637	15287.06617	28242.34011
13922.33213	12352.47294	22800.85891	13895.76218	12656.6954	7718.711214	13374.537
288019.0184	222860.694	388964.3198	287794.2793	249211.5694	160244.2831	297849.7245
339555.6183	203121.8339	410951.9439	312714.2956	255345.3286	155128.2128	296350.2989
652706.3956	350531.6842	790168.8527	546964.8763	431224.1567	303192.8436	491711.2396
9699.930583	4959.6152	9667.296	9014.432917	6909.783	3018.170556	7292.283727
17393.56371	8893.579167	20549.27581	18925.83768	16242.37488	10055.17014	15180.843
122370.6198	103558.2644	115807.185	110650.046	102521.7714	84888.06938	123736.7224
48301.19192	45229.257	51310.81112	47938.78147	46031.86333	37576.37161	48364.21888
11081.00506	6927.3072	11151.42989	9732.268263	8278.765833	10345.82136	7733.267588
44002.43484	26713.34461	44549.57235	34514.21104	31473.4487	22401.38721	33092.34067
82876.767	42393.38507	99019.80248	65169.16114	50772.58917	29665.47226	60925.28836
18398.3505	19779.85253	18222.69607	30088.80891	21897.25176	14229.33622	20499.276
52707.78444	36027.83661	54366.06511	51251.54378	50664.21176	30828.4655	31676.94267
6309.893333	4049.067	7414.485526	6277.948471	5090.444	3366.183706	5141.991556
11179.68006	3214.462411	8053.752205	3083.844604	3000.567559	2595.484635	2019.840308

845890.6214	815190.8938	985053.5125	902096.2184	782409.7412	569084.0125	989049.9274
34645.77688	34032.70417	37226.83412	37006.80342	37448.56353	29905.66821	40065.51294
132060.1911	123283.6425	175962.8324	169901.6284	145381.3964	105607.9519	163581.625
1869.672711	1890.080463	1919.897778	1911.871294	1827.962523	1643.84325	1366.040146
106089.9998	84432.72558	108533.9011	109395.1966	105204.5556	68255.40505	85076.244
1373304.351	1094341.566	1565242.076	1811758.342	1545121.792	1095443.183	1435661.516
8306.737412	7683.4112	8067.503053	13529.76418	10285.96906	7137.296056	7770.122813
45358.89906	37418.74618	43792.6725	63497.7525	58285.74128	40091.96844	53540.63047
33992.68401	5440.357	4917.151474	8147.08175	6957.193235	39899.29355	4629.703526
30537.48982	24207.18709	34650.88564	43358.94556	32776.27704	31790.77894	29573.03974
17111.2514	16280.28444	13431.49358	31728.092	19961.92089	19506.01824	26455.66315
26483.52588	28586.99488	36414.79271	33779.07935	31897.79133	22312.62735	29659.35371
1070961.66	1013935.613	1291176.124	1230620.322	1159916.072	915398.7648	1292817.728
1011393.939	964486.7733	1059955.14	1172635.017	1082603.202	851799.2156	1058995.426
13131.32319	8148.269706	4701.159867	11407.18494	10729.2256	5240.39425	4370.6884
110314.2348	98597.46	128912.1083	135208.6146	96548.74994	83903.10088	128961.0676
27702.28055	26994.467	30288.93579	30005.1669	26410.7337	20813.06763	26479.3648
59212.56273	51084.59517	59642.33136	60704.1833	54878.06739	41009.38435	54376.20741
60397.72358	42053.56386	81695.04892	73561.89413	54679.33444	38502.17633	55720.5639
2975.526622	2601.9598	2893.191347	6045.144712	4532.44129	3301.868647	2768.012857
44145.453	19008.36275	15903.766	39421.28582	48104.06269	24763.19618	19777.77529
5345.979813	5219.246118	11660.62433	4852.241333	5149.47025	5321.535333	8746.239412
190345.6786	200753.4136	262171.6046	233114.9949	231583.162	189501.1367	268966.3346
15462.33467	13341.15347	16753.0904	17467.367	17840.33767	13717.44	20866.20395
7174.218857	8546.439043	7501.734778	6455.113737	5965.01	5832.282556	7300.926783
5288.205505	4780.332765	9390.265564	7921.993059	7938.973412	9652.716736	7068.938818
44837.22353	30639.00741	26756.21988	44430.36533	55007.06624	37009.5635	34650.80944
4662.795789	3802.950444	2905.383938	9529.928	7592.9175	9903.673	9875.72129
869.3100952	943.81875	738.9447632	2397.816056	540.0935906	793.3466667	875.2585714
7002.97	5978.178	5399.3374	7363.269	6831.847375	7426.543125	6711.593125
5691.016733	9375.864453	10914.43394	12363.95914	7182.690909	4105.7415	8803.311415
9093.698571	7734.23	8828.537647	7028.060813	6676.869	9499.412222	8413.57825
6983.272421	4255.368	5453.26	5887.085	4612.136176	2999.9536	3608.560348
12659.97383	8708.9533	10270.21417	11529.83869	9496.030895	6815.431	7160.5405
27547.00722	25759.02983	25251.13867	36535.97017	29733.05533	35368.3585	40994.36583
1062.516333	1672.379389	1064.577881	1039.816167	757.123	1284.366667	1532.658063
56558.96661	53196.01467	48299.28094	64914.30537	51962.472	56475.32	66335.00089
9174.308	7002.351111	7558.943824	9202.625	7674.968	5423.455391	5529.529167
72235.86082	56454.67165	59913.88571	69695.20133	54242.34776	56696.80306	59827.70853
6084.367059	4565.143188	5606.566133	6110.59455	5005.276444	4280.908387	6458.996702
48011.84422	55677.18462	39782.2	43810.32913	31990.086	40495.84944	39128.74875
16891.842	13892.89007	12080.36436	16592.47088	14097.75373	15711.90646	12402.48068
4719.2622	7913.359804	13827.83412	4490.855471	12455.859	14400.45575	10205.49606
9983.115833	6912.689318	10186.97278	8034.770042	7446.474956	6457.911984	6078.976579
4906.452	5005.79254	11384.927	5760.96451	13060.29409	15552.19841	12443.79447
26564.112	20845.65321	27474.53911	28447.59363	19422.43341	20113.04773	45175.613
50223.90482	38763.4475	46300.70705	50073.72229	43697.94672	34126.49588	33757.0555
3683.686611	4412.213727	3893.271	5600.616	4081.406455	4104.436636	4434.165038

6340.538737	2542.571686	5110.502882	3693.602788	2152.295375	1477.546132	1783.495239
53549.27172	51040.72316	46959.7315	66865.6279	53234.9805	58612.3365	77193.00948
62713.54516	84940.23131	60221.72411	66270.19315	75938.0994	73912.394	102888.8932
41971.32237	33828.40089	50469.19129	41975.103	48656.608	41335.08359	48300.14588
5756.942111	12866.905	6851.410091	9106.956474	7430.001494	11807.4848	7193.589801
379184.406	350308.9979	342575.6254	374030.7197	283773.0471	346677.4676	433755.0792
6354.147571	9520.239659	9983.007048	10871.84838	7561.426831	10734.76437	5271.476167
14644.65	23340.94625	52890.97162	19772.99494	14950.27847	37937.14194	28664.26387
36075.14733	49624.98303	52975.73318	42278.16505	35661.252	33300.88905	36920.11664
3971.131236	7736.37168	19820.62447	7104.168188	5362.09901	12567.49613	9818.200778
6772.486667	6786.282632	7271.426842	10084.69064	4493.164943	5357.599455	4967.554257
172002.1992	162876.6362	158921.2751	174805.0004	138864.5608	147705.577	195515.1205
79768.19012	62003.62879	72721.00911	97339.79824	63038.62833	67644.399	88200.83547
11565.66062	6666.603611	6600.885	7658.296389	6255.6693	5672.865419	5570.812615
9544.09625	8703.24	10469.1015	9128.365292	9873.96345	6880.645271	6293.583333
10154.67868	12540.75505	9556.678889	15663.22253	10554.576	10740.07428	13212.3063
27941.056	23119.228	27272.91053	46086.49003	22250.22505	17591.64295	20764.10753
16221.56645	14806.33829	19578.44223	13331.20455	13336.8525	11986.74532	12652.29861
76832.58929	56907.04469	99652.87341	105876.3535	79925.50218	52732.45725	63275.27771
52818.60611	67609.86328	63716.33744	83352.31474	58276.74889	72038.424	64745.36017
15334.44412	16154.712	16574.08628	16751.89159	14612.73231	15521.33305	14956.05029
169917.0356	203181.0867	171349.3915	158658.577	134755.8011	162568.0728	179389.5888
70336.70679	53343.68504	73070.82193	56628.43371	54949.52171	37562.44113	41784.71505
195303.0494	202548.1041	189193.3168	263346.2401	198066.3897	217730.7796	244670.2482
23750.91537	21627.5649	29632.33411	26173.58033	22990.2365	16193.30389	16974.10006
30198.10921	23111.00321	27359.57733	23815.81872	23751.96884	16382.41978	15327.56267
723373.2683	631001.8634	752960.4565	662887.8665	552640.0018	505803.8562	559343.3564
1008810.293	954140.3983	884668.1588	1069779.834	650887.389	705031.4755	1052110.431
6647.4585	5913.271154	6625.682	5850.582267	5462.688706	4326.368	5392.270588
14133.68711	11280.83824	12893.29979	10797.53596	9841.443112	9608.634966	8190.626018
978757.997	897212.6418	916716.0294	1004741.276	825758.2653	701203.6313	733912.8016
46586.95509	26317.475	31032.72919	34162.66144	22859.739	28318.82675	31866.143
5802.706667	6727.535257	6720.419778	6038.52378	5994.819299	5754.9235	6679.89287
4541.362	6313.607493	5117.5599	5592.997366	5076.005431	4594.934	3061.7496
90497.28882	66247.02483	85138.25382	88000.79082	77018.53506	63927.43481	68449.17347
83640.70539	73589.84267	66898.55212	118303.1168	109844.5315	93544.34929	112971.9235
526960.4163	477856.5167	424128.3108	666886.668	518694.2589	572945.7357	580000.1165
643353.035	582393.2438	509762.8854	647038.6565	568013.8842	672093.8209	649361.5603
1578.865092	1167.99543	1366.885084	1029.4038	2171.487061	2146.120972	891.625
1908.489917	1575.056364	149769.4977	166981.4328	755.3372222	1613.648714	179417.9202
51239.11795	38319.93018	40783.55874	49309.49616	40018.58784	30250.73723	24064.04911
261664.3918	254357.8042	239558.1859	284986.9408	240404.1639	255531.0335	338228.0731
188100.9273	258006.396	199959.417	211687.4499	218889.8634	197485.6569	232316.4946
80698.27006	97373.49262	90757.78188	91028.18862	70906.24369	86949.203	104411.5528
19994.89776	18445.39514	18346.51688	20185.65338	17047.08059	16512.90813	22999.4131
7189.034387	8814.252158	9428.745316	10394.2992	7605.590928	8571.380046	8203.531368
126481.734	127454.0988	132164.998	146939.7739	105732.8532	122814.7669	168768.384
8205.154286	7071.626625	5696.241353	7181.39925	6315.890722	9391.341235	35216.01356

45760.60881	30237.41865	32826.13647	44823.21655	25871.89571	33692.35558	54585.34612
7873.989812	5013.127437	6350.927471	5071.621211	3778.202219	4458.749219	3270.949869
5136.868748	6364.76832	5558.999069	4683.5295	4076.097784	4272.067319	4087.13546
41941.09955	43440.05909	52322.98953	63781.00684	35661.92419	44247.88109	52438.8813
1329294.214	1291777.385	1376132.363	1781517.253	1112079.482	1262748.637	1995533.88
12505.49033	6305.835789	7873.19085	8455.875556	7303.321421	9349.731	31140.86081
5610.566063	6511.78675	6825.14675	7690.338875	5892.567882	6668.46	5893.653294
9863.726685	11075.02995	11289.46473	18162.42995	9754.946135	11504.95778	14813.84557
5041.005762	3189.0768	2900.399217	2807.296607	2488.196996	2876.881764	2772.414923
94330.69124	81774.03	86238.61433	85028.61441	69610.2645	68024.0575	73051.16971
6749.9685	12664.08704	7609.455	15226.18038	7051.99875	7276.878118	5432.167143
26895.2595	26967.22167	26717.53811	26281.0175	23462.5734	20477.17733	21463.76956
10492.13657	6303.959929	7426.846062	6762.520706	6494.408691	6779.396	5823.172122
715866.9194	608425.6917	746022.1533	1226670.784	682522.443	582242.7484	1373571.903
179800.7946	152450.1312	148002.0432	159395.8877	132591.4103	116155.0261	120115.0065
279496.4264	147202.821	287358.4586	380785.906	222073.4531	223137.4958	408665.0241
59937.59007	49802.40581	53388.1271	70966.46016	37561.93917	47204.85053	62729.40063
3024.569881	6408.652714	8596.79625	5207.672694	3740.569846	6544.621765	6172.337575
30589.60133	16923.97053	18466.2019	30996.45388	15580.61457	17765.89508	27820.89406
8528.948222	4636.073786	5684.831429	5394.332437	5937.593929	5938.956562	4686.017143
25026.88783	22510.26487	22138.92059	23851.254	20208.06296	19414.7508	19017.45783
6520.192	7788.651	14052.28956	6630.26825	27773.71832	20628.43013	22633.21865
15489.29383	22370.93722	17526.53846	17380.68245	12788.22559	15730.67309	15879.14139
29580.38371	31345.539	29924.21025	39544.57533	30314.97286	58735.23256	37702.67627
80306.41259	52123.137	64761.34976	67165.48806	63504.58494	49161.14959	39090.2575
55978.44244	36997.704	43500.19875	43561.84813	42708.70941	31008.22919	49043.34372
1489300.636	1103075.479	1306694.478	1269472.614	1085734.369	882941.235	1083812.441
77442.72981	35085.65325	51229.5955	63771.707	48956.44341	33918.12375	71878.89234
214020.731	206407.6443	279056.1345	174704.4351	176689.2011	170781.5233	188483.3845
438998.5243	426725.8232	484365.6811	428454.9872	363653.0966	345097.6983	385452.9669
56359.5435	55401.432	55543.794	72107.12337	48380.01035	58386.21533	108073.6901
9040.169437	9110.6262	9388.374067	10826.492	7745.315933	9437.716375	9853.97287
1772333.654	1600971.335	1772519.627	1592006.424	1297218.458	1392402.622	1570427.868
61235.12325	53549.28626	58862.313	83710.31238	51827.826	72841.50924	85489.31996
17592.17392	17598.36059	21927.01497	19697.50496	15226.15588	14255.295	16225.22767
6695.918077	7983.871133	9400.002733	8219.821333	6101.928643	7018.265533	7285.916308
9489.190937	8650.671864	8108.452542	10599.99043	7515.365379	8464.070435	8486.918
187128.9106	157739.9829	239594.94	200279.5675	183001.7022	164148.7931	206132.2878
1204848.802	1103517.958	1182188.268	1300467.897	1080807.208	976168.4142	1037486.737
16876.54942	15853.48532	16757.169	23633.93025	17513.9975	16969.578	17851.33579
49989.79658	38172.19795	66673.52237	43791.72499	48006.54595	56093.18837	84940.78278
18736.09227	14161.67795	20914.45018	17357.72775	16537.01361	11151.9686	18280.32633
405723.288	411717.4369	330020.5801	475958.4225	391586.5758	344256.0421	375036.5077
19445.76433	12359.06481	36780.65108	21443.55669	22006.32583	14609.309	16596.617
8188.432111	13109.19553	14755.54704	9110.624203	8113.555832	12006.59929	14393.75047
575071.0216	541829.8775	633453.7365	723648.7959	713313.9006	748372.6998	967018.8915
44745.0417	35306.01729	44112.34138	42928.18386	34513.68305	31936.94471	33052.48518
104445.8638	81808.46528	96058.33116	86230.88021	84980.984	50693.88361	49215.89929

817549.8457	689421.4253	655886.8524	938302.894	795079.5146	654842.0612	775044.4615
1624421.755	3049654.006	1395325.774	1581953.104	1358033.982	1096227.263	1334320.629
85680.15973	94852.65038	57188.56	112826.6017	74116.16727	95091.978	93185.81475
37420.92626	31340.96669	31031.5847	39197.8455	25119.45973	29417.664	28655.94533
539636.5023	393455.4722	472136.9793	535064.1799	474708.0646	358273.4986	343290.354
270665.5692	391773.2173	327960.7004	333397.2521	251595.8977	277203.2464	275624.83
40149.26296	33610.60093	39296.34235	42033.11082	35460.6975	35389.30094	36636.20714
95273.26826	64508.72923	137170.5904	177681.5503	126859.1765	70398.081	91383.8985
32414.31	14476.44411	23012.21156	47223.74492	31991.94068	45124.14083	23007.21762
18876.1755	15397.26789	31372.83927	18972.7631	20474.96424	16886.00788	16984.74411
11620.71674	8946.213609	6359.838	7637.051737	6198.894316	7597.201579	6339.042733
120025.2919	106336.56	114649.5016	205908.5965	107748.1203	95392.57559	156904.182
34021.20448	35670.7585	37728.4512	39830.2164	27115.51808	31200.852	34625.7615
5190.539846	4477.222588	4076.094	4967.471625	3508.0818	3979.842941	3916.867826
12457.83	11530.32313	13490.31741	13932.97412	10013.03647	7764.742562	11038.20048
31392.653	26487.00278	28133.71672	27335.14824	24987.21926	23819.32989	25439.53347
88469.72989	79076.64658	81799.15695	79696.89982	67032.16236	69014.361	74814.2252
108700.1908	102434.0144	106140.6045	103201.616	69556.96683	85020.15822	99274.90232
53249.5139	47211.17867	53497.38976	73989.1787	45546.9997	43833.04933	80730.26585
19006.3095	20886.96267	16894.098	21441.17889	16145.35	14350.44894	13133.67493
73164.61119	57163.43675	69844.35331	69413.27518	73298.91013	57490.31074	68286.25248
31305.82792	31147.53901	8269.44729	34251.31162	26463.91169	28656.50076	32396.81427
46585.75086	43570.37248	44980.917	51718.0335	40245.172	37175.75515	41294.42975
220707.1244	207713.301	214960.1267	228516.4344	147098.3395	167188.3266	200762.0986
30225.81771	33257.48782	25955.71742	32958.0855	24731.00263	27917.47684	29460.23168
76355.27498	96251.17346	32602.46912	33277.84633	25479.71661	28655.88865	27450.1
21789.01226	9479.5431	8075.035	9233.04	21825.01697	22288.42813	10825.76
16784.13768	13882.19322	16766.50071	16098.0335	12684.80096	10606.05388	13219.46509
976345.7935	779894.4806	974608.5937	869481.8151	794977.4806	692791.0425	988092.3992
18711.2822	21344.02481	22432.592	23638.73815	20369.9582	22427.30269	24117.38044
25063.72892	31553.70406	27020.964	28050.16035	18791.35353	21832.95	22680.00388
3899218.953	3609687.301	4284388.527	4098974.022	3733398.655	3191872.214	5714060.183
2302385.227	1790217	2137918.179	1777055.367	1663067.801	1618573.299	1810541.927
59327.38224	54808.50188	33515.46982	74224.6215	65748.41574	56145.47729	56732.71129
34280.97067	38085.86314	17557.77687	25467.751	19802.78957	32337.87726	18578.98727
202204.1938	204044.005	187143.6292	216400.6182	188338.0695	175380.5074	176294.5905
552806.1825	479461.3281	537260.3427	527254.2096	452464.745	414611.9726	442576.224
10216.206	9464.973143	8474.65755	10568.3165	10484.2845	9491.768323	7670.43165
888144.6517	921775.0854	926166.374	919311.2442	820241.2736	879784.7356	909116.8225
2575.956923	2930.9968	3500.797273	3005.137692	2551.394897	2313.139571	2193.634214
22032.11942	12464.304	15670.34025	16804.953	24298.65148	14707.692	17556.6545
5672.416878	7679.236259	7535.277706	8167.531529	5740.722588	6987.102107	8000.552143
124366.1082	115060.2295	138265.4138	124844.5532	159288.2451	159736.282	103977.4735
206968.8306	288195.206	591192.5774	247160.2694	180188.3829	247892.1049	560842.4203
24824.34325	30959.91681	34044.54229	33223.66937	23202.14847	29478.50953	38010.8916
50789.43133	88911.28724	110192.6144	40524.21431	76331.89229	32126.2602	92543.28049
313135.8718	259729.5587	281836.3122	294231.973	222443.1093	215886.3995	238950.9117
165921.779	229676.4419	168765.9559	206816.2954	83877.36467	89286.89154	138714.6549

8331.236895	9083.494	9018.554932	8703.076414	7153.598561	8245.058684	7689.738824
30932.17381	26852.45973	42125.08261	39730.78347	35487.49378	32695.50622	45674.56639
8873.81955	12114.19517	10614.89384	13383.93731	11989.81168	20723.87478	10349.2463
22941.78777	34404.12444	26409.8025	28214.00306	29051.16067	20427.37844	23479.94371
152121.3472	137642.3825	151001.82	129998.4793	122719.1095	67535.216	69542.46476
114909.7463	99662.6475	97907.68429	196814.561	155822.3427	156195.52	169761.6419
131372.2985	113007.7792	133827.0739	145967.2104	110882.8081	97122.37342	117097.6247
215006.1323	218190.2647	203458.2982	196912.5357	195599.2174	219143.7993	255102.0323
887498.6088	960550.9533	819596.7904	1057741.921	794365.1751	844272.7707	912760.3145
329825.1185	257191.731	352442.5337	253536.9166	221962.7235	183873.3632	230041.6351
979193.8337	751363.4042	906383.4018	781030.1994	774777.1389	502276.7916	501084.6251
76958.31584	67101.13361	69765.46328	73953.79235	55427.44887	55619.64133	58920.885
7290.52625	7941.818275	8109.073624	8875.746	9140.68845	6979.671704	6849.75504
478417.1061	396557.0352	485423.4251	473809.9401	431787.9948	350124.1023	391031.5193
1758008.965	1424803.466	1595060.09	1699778.667	1484273.646	1092998.86	1179730.048
656775.204	615917.4062	654198.5094	691006.5212	569991.4688	547731.6916	632648.9459
963751.3038	769333.6104	711554.1364	958423.1783	681980.0466	646411.8963	767123.6071
138091.025	131472.2705	134676.8518	153672.2581	120591.3376	124639.387	133537.9581
6766.137	5913.192	6821.620615	13763.02776	7803.27625	9758.6426	8110.208077
3636.115459	1538.346727	3045.211529	3773.155216	2708.23193	2772.006569	3212.917194
1961825.333	1527463.884	1622785.891	2327946.59	2045030.467	1774844.592	1901723.634
28487.84033	24075.29143	13766.35278	17094.59136	29883.77045	14668.75977	29210.63741
179672.5958	150097.3648	143683.0065	228413.9787	137540.6118	136245.6254	216945.7729
25628.8022	16354.03318	14444.22961	56527.49106	58267.49084	53859.93895	48054.17283
43707.4255	40644.40847	40823.8638	44307.5574	36439.5042	36878.98846	42408.55286
5271.265448	4727.152112	5065.309082	6119.465018	6132.594413	5169.207397	5257.574977
1183.750038	1401.856235	1306.042751	1448.543438	1212.856331	1509.598789	994.883
160325.8461	163798.6133	172343.9734	201217.9241	154073.3885	113243.3513	149212.2945
14279.24923	12555.43933	13449.43227	9025.5384	4375.941818	16527.74048	6498.0052
119498.8068	134860.016	100969.8002	131151.4375	115947.5494	113613.4354	104227.8865
51612.01688	54669.24306	50661.74022	32935.24612	30926.62555	43620.768	58784.05461
198714.9129	139518.6931	192207.3064	237586.289	144035.266	135153.9378	296555.7229
3296.430353	3291.555938	3687.355063	3765.838438	3236.990235	2979.690938	3162.086889
382001.0704	385804.5103	454242.4782	461110.3078	387221.8932	308631.2611	397532.6376
8624.044687	9902.212412	7117.502778	9773.096667	9070.302435	7713.43725	4628.090429
30766.512	30638.89686	22132.02839	28050.85191	24124.69326	32624.38383	28344.66263
27808.28453	18889.40563	20400.12186	22771.68121	15258.98132	14579.23571	18109.68939
26521.85111	19602.21116	27511.3445	37148.68353	22391.92189	20869.24567	48090.836
19271.5177	17949.56143	18721.82623	18014.84964	11176.79622	12361.40591	12886.005
548273.4727	585760.9389	485132.0821	652958.8124	463288.284	450863.8116	432943.2651
4858096.02	4453264.92	5986444.801	4804736.165	4467005.166	4125615.204	4928709.55
104574.6438	108699.3939	96384.205	114221.9791	93601.80683	121207.043	126690.5247
25948.23162	14903.54065	12543.22367	29037.37145	9814.9815	9575.524174	11533.92774
9750.889096	8918.71309	12215.0115	11072.7238	10882.44045	10135.62294	8818.694
4440852.583	3772470.211	5072876.144	4356473.161	4017676.953	3675083.881	4270852.125
772962.6671	623690.505	786873.0631	649360.1309	618639.0368	524828.3995	592308.1375
2784.533991	945.3568448	32.38947366	78197.38538	71972.93461	30867.69904	44542.88242
598373.0784	792105.5191	720792.3811	659346.5912	648700.9879	795994.1366	871391.42

5644.295067	5854.0125	6193.2324	6552.7605	6831.141828	4911.004714	5299.566875
8671428.345	8052726.758	9938497.856	9416040.699	8125675.205	7867085.261	8752643.774
276340.2826	237432.4481	379892.2879	290189.8765	276418.1118	210343.77	258441.2175
1542488.925	1792158.17	1516825.332	1846167.86	1530239.262	1738190.334	2011306.346
8057.12885	7985.273743	9031.716	8888.428297	6039.325385	9244.252625	10042.43622
14154.38446	16883.88652	14561.2884	16621.514	17291.07735	17725.2821	15422.87053
4620.144	5185.263273	6843.977233	5963.862333	4321.238313	4753.952385	5694.230929
27497.033	9511.67875	22795.27444	9535.930904	8486.39	11211.03083	4960.302857
633426.1281	730858.8962	621764.6489	751966.7057	630770.4098	696307.6699	817261.4357
1024119.898	982081.3129	1189039.697	1140301.043	921936.0142	897366.6971	1008227.15
648120.2126	674154.9233	719977.5275	707490.2651	593389.9678	580086.2868	658434.2748
14626.90331	8337.829875	13744.75129	12173.71835	11757.77	16173.67675	17349.06888
10917.45809	8735.370831	9708.21	11740.38328	9772.381606	12826.85904	13501.32685
12963.52556	14546.89695	10283.45756	8213.482916	6948.12529	8454.0275	5901.984261
64593.2794	64196.01853	95648.5475	62723.60448	47921.082	33406.14095	36422.74242
7798.381	9246.856667	8326.032	11191.43937	10107.05623	9005.875238	8105.440645
125235.8753	126184.0372	139798.6512	132025.8135	117937.5206	110884.372	112569.5343
479134.338	484719.0397	326230.7119	491982.975	105989.6074	232119.189	261519.711
2940.487688	3215.258471	3285.319235	3590.440765	2730.242333	2570.669563	2802.469235
9933.006357	9956.141185	10962.9675	14151.76522	11753.37733	12369.68837	31619.61255
13064.259	27145.426	16362.91116	12420.96239	25692.26986	17446.84708	9474.6352
526339.4491	396687.8564	422576.9136	475840.0849	435076.4892	246995.4054	223709.936
157756.86	145917.0815	204330.3009	148815.945	139211.1626	90663.02618	94905.333
15816.25848	15002.47325	13613.73164	19354.41	14168.72559	15146.35864	18255.04207
164054.6284	176394.1966	294096.7455	123515.0429	165698.1648	290962.6881	266579.244
64936.998	57191.6052	65212.08893	69844.284	53331.78651	54679.73716	60289.19279
3739.991294	4522.589882	4541.04	4894.078	4401.82544	3912.058062	3791.3315
9691.719674	6911.332245	6652.069875	8458.574643	7390.18621	5596.048187	7976.472506
23857.16982	22447.77157	18806.36303	24067.72973	17288.53498	44436.57097	15494.4674
18795.14944	53600.34076	21368.81533	17792.4195	24177.18933	13931.75032	14175.83394
3656.0518	3857.3042	4634.082167	3553.271429	3089.564535	3115.645714	3004.978923
287710.115	239488.1298	302890.6874	232762.525	238153.9146	158530.1186	168661.3667
615013.8186	550757.5442	651946.6915	652722.4025	554363.1078	429731.2733	455035.9609
24385.543	22583.82758	14368.07639	39818.67598	42234.82051	34217.91245	33718.81102
71948.28432	56760.45821	68287.141	79114.59468	112019.7645	55737.66186	57453.64344
59880.33788	51847.49533	50120.87181	74391.73358	54664.49982	75472.44129	72670.75965
1557376.164	1930456.464	2474740.735	1503410.179	1377841.675	1074317.639	1232767.421
38614.28385	30443.672	39473.632	33633.64114	26009.424	21032.29684	21740.17198
189644.8683	194171.6354	193582.7756	198119.3502	171963.2954	165295.7709	184452.8435
9673.561091	16916.64245	17614.17646	33424.44366	20306.98657	26401.33518	26512.74435
24577.784	22547.26	29864.60905	31944.17325	23979.52753	23443.02071	26988.55828
11546.36786	11344.949	15928.5475	12938.3305	10400.01021	11502.46785	15074.359
32120.14176	23576.88277	21137.87714	21164.66496	13820.9721	17609.00259	20665.9611
13006097.28	11885449.26	13877043.88	12832221.29	11434700	10959357.3	11899927.02
43027.655	38191.96171	42273.87378	49850.14899	39472.92	40309.16588	45655.50804
5469396.463	4916309.063	6142942.701	5485142.009	4763845.272	4599070.886	4974287.967
33258.70471	21837.22333	28083.66011	44319.30565	31996.64341	38271.3596	22880.08331
13707886.88	13894471.38	13588592.02	14949865.43	12521982.06	12330285.84	12793797.25

5386.659851	5138.81197	4610.549957	476.252	4242.308935	2843.074714	2895.399111
2243.625013	1513.675	2156.146125	2787.445875	3101.087	1610.6769	1822.19126
13675.05288	30530.13751	13856.73053	28597.56226	10971.40931	17508.86836	12574.59686
73236.38606	89362.21882	77174.15107	119646.0776	141228.1254	119612.7887	105522.4085
518005.7632	376216.5518	502804.0358	521096.3809	465851.8572	438477.1382	473907.0784
64357.4745	67042.06128	68853.514	65561.33053	67520.648	69911.06256	67331.29572
22604.87853	18595.10374	18506.87531	29105.05784	24659.19983	22317.93073	20283.55312
1061572.378	1234536.924	1331862.696	1337535.774	1141653.598	911199.8895	1167545.311
163383.8764	175290.1618	146186.5605	141366.1491	128641.7314	158003.606	127275.2513
11806.18658	10326.60722	13865.69665	9079.398279	8263.104876	9599.413889	25218.85847
196425.6	239587.6843	248856.7829	246456.9251	214226.903	217078.7492	496128.1733
547855.8694	578588.6431	496123.2371	605218.4344	519075.6883	488869.9757	495450.2012
99465.17226	108596.0225	93586.36267	111578.7969	99110.76347	103446.074	106743.7974
13459.67709	8948.270692	15258.67444	14856.96662	9859.416188	15175.30621	14010.88472
1544.993125	2120.192312	1920.277375	1894.162	1737.4895	1673.43	1317.07655
1756.68	1771.678286	32899.45545	2561.878063	1695.852308	1800.348	1855.906813
267774.6343	278644.0427	242356.0588	302891.7401	255396.9538	227274.4384	234724.9585
19890.65035	18841.82129	14375.21606	17165.85045	15467.27569	17533.62133	14325.34573
38061.8785	44489.63077	41811.8124	47122.06985	35343.86983	36110.88431	46887.79921
142250.3161	166045.9518	133490.4211	157327.4775	133910.9218	155314.9689	160862.0543
7607.9358	3062.681579	3392.821421	5033.248	3171.56755	2763.49	6188.656167
40839.219	37742.80418	17890.41392	43443.4194	19365.14731	21977.1342	38883.81768
30792.30194	26815.6275	26348.588	33163.42118	27608.017	23383.78206	25760.97856
799074.4918	398300.1029	787352.0754	769997.5048	420862.6671	602976.7794	408045.2899
15733.01314	15195.78667	15841.0301	20288.0941	16542.56233	15091.95111	21474.47443
261030.443	226950.1975	277669.3224	295692.3492	286493.1363	240259.9217	291318.7922
86106.1872	69519.54382	86029.9624	111115.5517	76331.07525	76957.51828	116739.6153
78086.95284	86245.61968	74868.90775	86194.6306	69691.85232	93401.84862	105737.2164
7182.602483	8528.297394	5352.543625	5812.704651	3864.025286	6655.274233	5439.588771
7747.361617	7788.576133	11000.82372	10118.034	8678.650167	81920.18352	9256.7088
5887.177111	5802.001692	10842.32993	12961.00757	9851.834	12539.0918	12524.66216
646925.3544	564153.0737	654753.8832	641127.8177	587128.4663	491935.3029	610677.1265
719638.7664	704340.7358	765800.1	789424.0819	694711.8455	672804.506	691116.804
73486.69183	81482.66	73282.65211	100455.0797	80195.604	84369.87478	119547.5543
15859.26893	17786.56052	15643.7619	23041.21041	17737.38804	17787.6784	26051.31763
786.15375	1399.87275	1846.427273	959.64	1467.350909	2004.493733	2924.981085
9616.257692	9090.850462	11016.11	13435.011	17546.39072	9925.763077	12314.268
30671.929	28139.77467	31006.39133	58417.134	39790.608	43726.51348	25810.95833
43856.22633	43298.45148	52726.88577	49397.597	55112.757	24629.49225	27081.81317
445867.908	389238.885	511860.4527	448064.3409	401713.8058	328628.3644	358411.1672
473965.6483	490152.1967	508027.9769	556593.1239	456358.6812	776083.3094	529271.8985
355393.1977	290393.3591	324514.547	607692.0545	376971.5288	346377.3985	780823.2959
1612.426465	2525.45	3109.152692	2766.439	1886.984762	1903.614423	1753.494531
11582.0124	9470.898407	14184.74436	10948.33161	11644.97214	12823.46541	17648.47692
191750.5299	164118.0154	212897.3568	203153.0165	188829.1311	139689.3351	142136.2055
3421.518417	3270.759957	4699.335161	4745.033116	4296.704407	3957.327148	4775.55801
102548.9036	47865.51843	96865.68648	47594.2665	44500.93758	44975.13811	48337.42317
35568.04697	44533.57363	33943.44803	34708.74831	36672.08636	36073.9874	36273.93396

110460.8794	92947.75487	18409.72557	101114.8124	81279.58091	64266.37432	64368.42833
1224855.01	1022491.55	2278079.182	1245889.604	1142244.864	1083996.462	774508.7213
62318.86359	68627.52847	66117.733	67542.63476	61525.68724	66240.05648	81323.87481
160096.804	146808.343	146603.036	166679.1751	150381.728	108743.3156	111259.7664
7969073.43	7821259.867	7298494.179	8993026.726	7838439.05	7004408.641	7191558.593
2003203.731	1725276.252	2141606.833	1806747.824	1684217.329	1351055.902	1546973.295
20243.49042	17896.99427	9217.581	9818.767273	10101.53482	22089.64652	26980.10195
28459.33374	13590.60814	25921.9265	33389.73885	15670.69617	25418.83371	25833.36619
21836.89152	17971.81176	17111.44688	16247.84661	14647.86619	15496.2954	16555.37871
27507.88708	46360.3396	30085.59172	25633.51833	26694.73566	36378.44318	33603.26375
17391.16326	10751.961	22559.73427	17428.5165	9734.088	4978.462909	8419.947923
52959.70433	40520.82116	47808.56661	67295.40565	47815.67435	48176.46181	59354.9385
71752.24848	62243.55657	62462.63248	101938.8714	60644.59856	60723.78955	61800.54629
6034.846842	11442.28978	9341.8299	6075.375	5413.333984	6261.766176	6151.147888
4327504.553	4286901.039	4901976.744	4512694.986	3897295.882	3950049.182	4160288.675
13330.9678	14669.537	15237.71419	12833.39176	11772.91849	11671.6519	12233.04369
686587.3894	655611.7929	705563.5325	1089948.058	757323.0149	854716.3018	824021.1171
11489.36813	11854.55838	11500.33901	12856.38457	10049.73463	9919.694114	7014.045412
4938081.319	5677533.365	6021984.969	5129574.954	4220068.367	4317133.389	4528824.745
13972.03888	12338.37941	12741.17572	13117.82715	18625.66514	10487.18341	14905.68416
4458.035714	7372.019778	9939.213059	13007.428	10840.923	7815.952941	9209.111294
78240.39447	75525.46222	81987.19022	114635.318	87849.74242	92528.33933	91233.97033
385040.4537	387291.0666	395284.0613	537434.821	450180.5501	431570.9453	444244.595
3554438.004	3640200.35	4177245.027	4398162.496	3616020.039	3403891.99	3566942.23
37675.29088	53588.11135	55489.887	82888.90912	75761.95	68486.627	57440.54547
115106.9482	54439.22133	56725.7061	79503.18371	63602.73	69872.58264	56606.09826
41074.29777	45721.36252	49470.63376	72927.53557	58416.8365	51251.04893	53006.26418
86819.33978	71230.856	89265.27333	1384375.456	914916.2158	703449.7448	1189933.538
432254.2295	543664.3913	510158.688	460006.3652	494019.5187	469837.1948	559523.6994
84053.05146	73017.18086	86807.13427	81994.36079	81651.50586	66411.29367	73031.17687
7775.201438	6004.0055	7945.280863	9550.314	7878.028813	6191.301187	6541.591125
64709.31409	53027.95582	74341.9444	868297.1019	447054.9639	358359.4315	586796.8779
581384.6247	519159.1878	498773.7738	601706.7473	482328.7609	659957.9137	766546.3596
18961.47461	13586.08866	20016.22663	141646.5539	110790.2908	91887.91827	127775.4381
3142750.625	3690326.355	3610189.894	3921276.117	3403593.4	3155843.583	3295981.02
1477100.481	1664829.344	1690528.631	1845113.99	1589739.11	1573929.435	1492981.739
158723.0246	145386.5824	148292.3308	150985.4705	140792.369	112344.5829	261230.2592
11170.86122	8775.9228	7952.776563	29736.24027	21383.53208	18858.27658	24809.97721
124457.9438	130527.8987	119303.3676	166386.4195	134819.2186	122612.9324	118158.6162
302383.6795	309625.1836	270225.7121	363084.8012	314887.9391	242885.175	257137.9733
93087.53232	106138.2482	104419.2433	117052.7014	98752.7331	82405.08367	94226.12355
88237.96329	66762.66531	78813.37692	69121.3785	64806.90242	68195.79831	67787.96562
12028.59794	7009.132929	10537.67076	7534.798056	6151.581857	8055.655796	2932.7337
76888.32245	80878.06474	70366.24897	96438.54968	142974.7973	81589.66789	85882.87726
9425.405813	17180.30953	9674.549125	10620.4636	9079.123125	12460.37836	8003.7736
16357.22653	15070.2325	16927.05529	16605.89285	14576.68395	14019.75483	15720.0715
203267.8477	178462.683	197171.2394	213328.9808	165535.1516	163128.634	151795.0057
5571.5925	5988.842667	8901.93391	9573.514797	4985.498143	4712.87	8310.841938

67853.26212	65435.21065	65573.7765	77990.02747	66680.25269	84263.99521	66252.81482
124331.0966	119472.837	138581.3169	161072.0684	124637.6179	118380.1009	133924.7
12165.36576	13001.16194	11567.2282	13238.89167	7156.575	12448.05844	12055.48761
392631.8049	365600.4837	440424.3076	505179.0723	432564.4946	257039.4213	280825.2046
5940.717	4028.351105	4907.329297	5900.385807	3148.162111	3428.687059	5197.294169
195289.9881	184456.2403	210014.5994	230282.0352	197896.2062	180234.1784	189902.3137
25867.5517	23561.16227	22041.00858	32027.90594	24946.23588	25372.65661	32726.74347
37853.25366	38608.63266	38015.0358	49253.33073	35105.54036	40598.39633	40246.4082
235687.884	208938.2139	237656.8827	321962.4408	235853.2918	267349.7433	244315.68
1414127.75	1239167.639	1350158.027	1632518.12	1459781.343	1007700.446	1067638.624
128868.7443	133224.789	138271.5549	143055.6762	140795.3599	126932.9333	148324.014
243127.5846	197394.7236	234395.6105	310402.2989	234781.8397	228939.7507	299218.2477
6417669.834	6013485.545	6340869.677	7327630.986	7770436.606	5557964.63	5802062.401
14046.0978	607468.159	21138.32158	30029.54763	30361.89555	21217.6544	27567.70083
77352.74033	63991.60376	75040.62081	110990.6249	75739.363	66936.50137	114706.4924
207007.0702	203357.9408	204199.3562	240594.8201	212165.894	168522.8761	190412.8497
7678496.957	7996260.203	8902407.789	13836879.58	7512346.449	7644737.59	8196227.998
3331723.459	3533983.485	3555228.166	3856620.775	3556278.164	3169596.667	3249646.172
449511.6757	389385.9223	367822.7195	386354.5113	350931.0417	301660.9099	317513.2989
3419769.957	3604492.039	3919821.806	3985404.246	3350817.228	3668487.671	3691635.063
419414.6735	450886.7822	451900.3614	482677.5141	441449.1317	395346.2757	412187.9642
1099835.358	1128442.537	1217385.176	1247882.208	1057745.603	1079604.427	1096649.741
1487894.965	1561089.988	1487534.698	1828689.153	3064759.812	1470255.531	2873316.447
1375004.508	1395993.239	1289769.544	1461221.943	1217779.005	1229635.7	1229140.899
214425.1565	214892.0581	270032.3686	172197.2009	159736.0283	228535.4478	160272.1488
191492.1208	227294.536	211238.7989	253637.0901	224754.5224	214401.0471	193507.2969
664159.5252	730664.7858	756805.5095	803859.3638	709795.2455	542388.4892	702212.5063
31218.33397	27631.89784	26538.67713	55569.88313	43542.82971	29513.83129	31807.281
666935.0245	559393.5579	581952.1913	884574.9118	665163.002	712435.8382	764263.9773
722330.4263	622686.9524	739181.184	702991.0777	735942.7693	651378.2108	687927.4671
36978.26583	45329.18153	50218.92394	86329.19965	78363.177	129666.1689	62782.52689
30642.85987	36783.04924	55126.55657	62954.551	55371.735	40700.07947	36982.41806
7306180.177	8164537.248	7821581.421	8647215.999	7017278.202	7252021.942	7955187.246
682194.3997	660176.383	776219.9509	784562.9883	713796.6876	597605.4909	651577.0633
35349.654	47378.60438	59889.06367	79431.4648	102093.0013	62901.9824	52445.23667
89127.0571	79927.39536	149588.3629	82477.82156	73546.90194	132486.355	90507.54625
6665.866499	4029.49125	3091.628571	5839.182571	4916.708357	5657.891417	6296.515586
352361.0103	412676.4451	472304.3825	502302.4431	436576.014	397143.1976	402664.5208
157927.016	211079.3175	246378.6923	400530.5676	352499.4259	348113.7568	300554.1125
91824.78895	102170.1896	96923.6758	117124.3584	91171.83624	86628.59989	87506.18747
141114.8923	164935.0184	200663.7641	332002.241	275198.6963	275234.4388	248049.4449
62937.21253	77745.129	80856.99729	70924.9735	68555.896	78960.9835	76669.17758
58224.10175	55985.44819	82264.5291	66798.63248	59437.07765	51789.07678	70355.83591
162679.4728	165595.0337	192441.1259	183475.5876	181241.6978	172046.4175	205425.4217
213843.2394	164118.0567	250554.7742	262009.1137	221986.5619	246777.5828	241915.9038
71872.88171	88204.92275	91375.2786	82791.47333	149844.2728	79413.4546	76859.631
6046.24	15766.20356	5946.922412	8330.196375	5948.029059	5507.628984	13257.59234
88127.79392	81191.50947	134674.0402	82175.68114	59393.97682	62407.28274	69345.60048

55022.27678	57367.0945	60340.37653	60953.0502	52082.559	50297.72	48141.5936
11533.06617	12750.17206	14827.36617	28249.43394	24825.305	14236.43912	17597.53088
16136.41623	13233.72203	15991.89043	19199.22767	19664.27336	15881.78673	14198.65239
1126942.824	1062723.956	1204373.319	865894.6037	814717.1187	827072.148	909033.7243
15982.4728	16168.25143	17462.8306	18427.90805	8231.7082	31458.15115	14975.0218
12814.64594	14261.77773	13360.807	13204.97093	14172.26147	13212.531	13171.65519
10379.61463	14697.49791	11899.32013	30796.21835	17303.298	15161.12988	17800.68995
114518.0337	117580.0442	109594.3429	101572.1178	81228.6783	83050.32125	77059.76953
3066216.659	2891093.139	3145537.896	3895198.027	3331777.717	2598851.95	2920737.403
62859.37806	53759.92837	61731.95933	53407.5805	64851.75067	63560.0454	54988.15519
9429.273434	8665.706667	6256.381592	26811.77592	27155.39181	23538.68763	17579.9925
13046.22058	15965.33786	14028.65166	13274.07602	10901.8523	11751.8273	11007.40239
16692.73011	19273.78726	17931.67394	36989.61118	30595.81594	28168.69444	22936.48782
31693.67081	27448.71962	30736.28256	39488.68563	41653.10529	30530.81903	28836.51082
139898.2015	135058.6108	148758.5987	161377.6867	119446.8208	128251.557	137460.3226
112522.9612	105564.3783	109096.3181	32606.62533	29862.19268	116283.4039	121949.5544
1657428.441	1621762.542	1901573.154	1942707.95	1718688.335	1557679.855	1740845.582
14972.62667	15565.09091	40119.1662	22010.04527	28304.48547	15278.2341	15721.38172
43566.8613	35570.0484	55772.36934	54052.38995	38274.38078	40155.67743	50574.48107
6216318.074	6575823.719	7850960.363	7029335.908	6358020.586	6724203.211	7336103.742
208789.8994	186160.7985	177732.4437	174399.2061	181203.1596	150063.043	178426.3935
13720.26812	13760.2625	16363.6025	16754.57741	16586.73092	16235.84663	193400.927
16675.01625	25866.73816	26153.83321	16494.47594	17039.27175	17124.19485	18042.2055
887580.253	851923.0262	969426.6811	959922.0775	866400.5741	818674.6288	938474.2551
3390219.389	3786594.515	4537452.069	3820873.351	3538320.705	3914328.399	4265302.594
28560.19243	23742.28086	33064.85661	34499.7209	29669.53988	23468.49459	31363.5876
30438.7791	28046.46344	33948.43771	35676.06016	27837.05065	28281.1485	55277.2885
4008.243877	4443.85479	4835.273204	11370.50109	6451.004368	8969.7435	11575.53058
2040719.676	1807467.694	1907819.708	2640133.755	1539456.043	1811172.168	1781961.041
415501.6843	465101.6441	459525.098	495923.9868	470261.0001	446506.4857	465583.959
476241.169	524618.6795	521815.6943	542065.1034	482360.8745	499603.4556	522135.8847
17991.62071	20123.00871	28049.86306	43715.658	32714.53624	24091.3005	23501.82306
139157.4361	157640.5954	164477.2127	173968.0323	156909.0369	148275.3704	152461.0019
213820.8088	251825.7783	243382.8715	261439.5246	232553.8722	240328.7334	242699.8029
72591.42471	83009.967	87574.43471	157097.5381	140718.4651	109772.6899	102530.2142
89054.24545	101522.084	128554.2177	214921.6836	172688.2269	132993.4032	115078.4267
14726.98865	16260.56678	18661.12155	29766.16129	26683.34392	24090.37041	20993.5218
306712.3121	383962.0011	522263.2608	790212.432	798999.1254	606375.9483	502443.7026
11769.66327	18084.04364	15106.13029	16554.762	26215.39168	10457.68044	10599.9336
134610.4448	124661.3488	157464.8786	162458.2804	161697.4188	136209.3089	141079.355
1990.724	2344.13287	2343.271649	2989.807076	2019.7096	1989.215318	3669.806364
391495.8167	522475.6044	575072.1495	1032095.445	885501.9988	911421.3859	753038.6404
155471.4486	144212.8199	178993.0834	212272.129	136243.5187	147745.5143	148999.5754
121277.2552	133593.0848	170820.9176	178787.0752	147172.4558	127540.8428	156134.769
11314.6108	14659.25344	14257.9208	28858.77181	20362.27782	20026.8495	15784.9276
31855.76575	27672.71852	32069.9246	36864.95222	26856.8721	29898.5691	31409.23326
31496.282	32554.34627	38305.32111	37996.992	33011.248	31800.92025	35304.99972
158679.2469	166332.0492	238047.3311	227321.2343	171821.965	163189.4446	246416.674

28709.816	32634.8728	48231.062	49177.31995	37429.42796	26935.438	42915.14733
94745.54016	81252.74491	100529.413	122978.5181	87926.87007	70532.54187	132687.1548
48607.03479	56506.59846	44773.76842	48102.48489	44149.97633	73133.64279	61683.082
13945.34029	9246.3765	17352.07158	17396.36466	15369.23035	18977.90606	53821.68477
7704.899132	4141.075748	4449.186546	4445.013694	2985.84	3832.272438	3744.209063
25255.95276	28040.25263	29733.26647	52902.28125	46729.4015	40627.8645	44328.30317
556215.9113	670317.5952	748649.994	723763.0735	578532.2888	645415.5887	742853.7695
19191.81691	19836.4806	20973.96308	16947.46575	13792.38068	13272.97782	11774.745
620474.3383	799631.2679	806531.7528	774429.837	668737.0018	794490.3144	886293.9043
10965.4746	11489.07945	11209.4404	10461.1047	8060.9345	8693.82525	7399.537579
320518.3123	398388.8059	408960.1773	402527.7529	338754.8235	396321.6402	444019.9105
305176.4683	220733.9729	213988.975	275711.2072	198300.9473	309560.995	217186.3685
6610.373053	11274.38622	6139.16	12161.38782	13001.53167	10823.85812	7607.747722
22283.70548	25384.52912	31149.34122	24855.43405	28540.2804	26075.28	27971.98632
15596.556	25938.04659	20344.608	23265.29593	16196.89094	33318.25513	17284.24694
204143.9527	207928.8646	240919.7835	222358.8547	202311.6083	209653.0468	227952.6688
22795.75371	30676.3243	27998.97084	22556.34743	23598.24648	22694.8806	22444.99918
28727.88258	32665.04366	35706.98338	38489.72464	28073.602	27692.02304	53453.78567
40466.63665	62817.28624	34938.9018	21198.681	40054.2417	27889.93647	27804.9772
7113.638245	8728.269447	7601.108273	9448.948482	7692.953935	7335.847812	8513.741
11385.59985	11466.59004	16561.63863	12686.89283	10014.45343	9888.055567	15444.53915
47389.80235	61714.61141	85541.31931	129201.552	94721.95688	84938.40082	81017.48806
14804.04263	18061.13778	21894.974	34479.82311	26446.02814	22152.54093	20090.26504
212161.7719	243899.2637	307590.5938	512739.1434	458593.8589	386071.3344	356320.8044
29379.5317	30260.58064	35243.56476	74317.95476	61821.95819	66205.23067	46195.10088
119329.8805	158223.0071	168526.2345	329485.1395	245322.5022	249165.2225	209279.1675
92316.31691	105952.7937	110483.974	117205.264	91716.003	86459.66541	140775.7807
6737.45993	6944.452667	9387.1167	13728.70606	10881.30713	10216.86138	12315.19571
13946.39667	20461.50925	15681.07384	23544.40699	13902.67235	14172.21474	14573.95373
317201.09	324306.7629	326627.4327	486412.6438	137028.1975	161307.2581	606390.0885
5559.696214	6095.267167	6032.509502	6686.093786	4318.223924	5701.675317	6277.895059
2152.327125	3951.543164	1686.001889	4195.892899	3239.052595	3206.306717	1298.031059
10654.61637	15579.867	11169.80742	11285.97671	11072.33574	12019.05	10055.69883
302171.2597	291153.8149	298642.3215	492596.4066	250138.4019	283843.424	516876.5082
47783.57873	46921.16882	49868.177	80299.53	48200.5312	48972.872	80868.95113
24577.0824	19832.048	26442.53405	18739.46678	13402.42843	25014.56313	45102.62549
11911.73113	16981.89363	20716.91148	26882.20959	21201.05856	24599.36294	24582.45806
861362.9145	1147687.877	1418188.9	988132.943	870785.5418	1217478.987	1383248.383
30041.62733	27234.68125	31045.74202	29993.757	29480.90796	33137.72063	33161.01672
28488.69994	44214.3072	33912.09173	42108.36891	23116.7188	30704.22888	29775.11744
29296.52343	29910.24718	31267.96035	33954.59965	23769.32249	28392.518	23592.9935
17378.78117	27390.59786	19704.598	14776.0668	17443.65805	18842.76007	17780.97143
8636.670703	9227.952413	11878.00796	8750.901231	8355.997958	12553.45091	12491.27585
16973.656	17530.75096	18580.437	20523.30083	17411.28517	18805.3255	27250.75543
6059.793438	5241.296842	6538.330333	11197.2326	8440.5416	9468.729136	7735.734765
11492.96006	12201.56141	12197.97394	22102.05971	18813.14235	16289.43071	15448.24729
6259.556625	7378.912537	7793.714833	22683.93642	11892.496	10174.29853	9738.855
2456.048571	2365.655758	2270.912063	1756.65215	1527.317342	2000.898926	1554.492144

53352.024	57237.31634	54519.74729	62435.69379	43898.27669	44824.54617	75452.63156
32147.19529	27815.21028	34242.12062	39530.06288	26116.32195	23885.33136	39145.54957
4356.768308	4247.294526	3379.853882	5095.23	3681.117263	4344.216158	3717.125583
2531.269026	2284.610401	1708.048599	4462.366676	2948.957129	1936.39021	2093.018765
19417.69913	18768.22519	14764.68613	31567.05956	21592.29494	16968.85106	14686.72238
4743.014864	4025.728313	3769.752158	4762.275294	2849.269647	3567.266889	3481.384571

CAP-305	CAP-306	CAP-601	CAP-602	CAP-603	CAP-604	CAP-605
6466.306364	9350.245091	7290.914091	5887.00525	6719.325667	8509.078917	5915.021167
22450.83618	20828.84679	17853.2236	25271.5166	18116.9845	15901.5444	34444.41712
1305650.347	925833.926	1090768.931	994315.0183	1205159.313	1382640.784	1618164.877
17347473.55	11576237.14	10482077.31	18958855.44	16268630.21	13975795.76	18908534.87
6445.90712	6340.311	9560.905467	4913.691429	4682.500588	4340.87265	4205.025213
259214.4967	203087.7497	266890.8363	234777.8899	287180.0733	250063.5178	310680.9827
10890183.16	10755576.43	9656990.875	10710689.89	9665644.647	9076800.011	11962246.46
13730.28494	4256.446063	5338.151579	11361.37982	14181.00889	11330.735	9713.841789
57841.92692	67934.58343	32295.90877	31014.23636	17692.23027	33140.61946	15610.3
113285.6874	172756.6869	113448.465	47267.47088	83577.7386	77435.468	364244.6124
183336.2449	92924.1655	115290.7473	117977.5549	138350.1156	88253.181	118918.1748
36330.47762	43369.26493	66403.75223	56763.83571	82578.307	80417.37677	35316.38357
183242.95	135315.0344	161395.4336	148418.2206	192865.3584	183768.9294	240311.1268
616362.5396	322678.5058	727288.1888	448214.1643	533074.8459	417680.1182	438262.4748
39069.84429	79026.45715	45139.03077	39066.106	26128.81169	37356.982	14165.60529
8634.16275	6974.009364	5542.030833	8462.326857	12118.99995	9724.564652	9361.090692
92004.99293	190098.198	137112.0068	120350.9435	66924.20625	98865.46933	40316.40824
324371.6473	930732.8989	491794.9603	348254.2998	200139.9932	288330.2785	84653.1745
128772.755	49053.71056	105545.1238	122937.9507	76477.87752	61072.56123	89774.04896
47335.53082	30648.708	40823.8246	36106.97055	38452.32	25255.40028	78958.84044
515154.1785	274189.5417	290769.0972	320155.2081	363993.2418	292036.6845	387819.4802
17200.90685	31505.40979	30279.62309	19149.27197	11610.84181	13123.66983	13152.02201
263208.6765	707524.83	478743.6074	311890.525	193232.9222	261870.7579	68580.08606
376159.707	595836.5769	692404.3212	364703.43	158171.0042	210361.3498	151493.7834
80263.02539	841.001	109765.2343	86500.85393	81974.80332	83256.08708	687.08
13300.217	34471.48312	37457.76033	21825.48529	56991.5034	33581.03517	15902.29333
23749.194	87457.5	54415.58367	39639.8385	27432.6614	30323.54182	13856.095
25202.15171	53678.93256	29352.10126	24260.70465	26121.80827	29124.38504	10346.35569
135410.5342	120756.8615	213229.0961	130399.1964	125211.6586	129519.207	174953.08
131378.0883	418411.4663	219069.2127	194202.918	62578.04143	134870.1024	34174.59419
540943.129	517887.8283	1012497.008	763592.9108	731502.9346	663554.5053	577760.2394
6249.5095	6835.72	27431.8666	17347.785	11610.53365	10721.00014	12217.69429
19597.4625	18710.51	25186.53535	21462.99157	18124.26525	16939.53067	21832.22
47164.75685	29919.77908	61495.616	39644.88	35639.61923	35521.35592	50772.07158
402923.7751	404298.614	442545.396	420383.8321	403324.2169	302880.1211	429582.4822
4343.948625	2421.531779	6477.449875	6972.129813	8465.600733	4996.451769	2971.623706
866904.6469	800539.0787	1445967.01	1033158.834	941298.0525	770437.8096	962457.7917
9144.552786	6412.027471	19687.7805	9661.064714	12626.297	9690.255	7490.879308
18736.31222	10535.51953	22990.34612	14602.51528	11948.04368	9007.618667	8050.35825
2933.479427	8522.5582	15279.4978	22571.93893	20229.16779	13824.5758	18313.7754
28083.17529	31815.9855	53426.85336	47273.54771	32114.25153	36683.05372	32975.06194
16467.10827	16731.55406	24841.07294	19472.616	18881.00607	17074.03288	18055.35794
498116.6395	476963.8741	586513.837	411470.2676	394852.3884	341496.984	460456.7383
251437.4777	230742.5514	289998.8129	240955.3318	214461.6088	158838.8894	172418.0984
231442.6562	235984.9331	302174.4066	276535.0971	252911.2054	210567.8329	238126.8466
17108.35329	10160.78175	27367.9832	10306.35991	8784.431458	6094.771429	23589.48724

15865.1595	7025.977765	18261.07007	11364.93346	9125.255176	8991.575071	17963.49407
8136.617154	7159.891143	15612.01454	16277.592	16717.64973	9066.735	12816.31107
11991.2275	4217.606833	12790.4625	21556.25864	11560.70274	7853.021062	14990.42
24576.11056	35110.81276	14588.13231	34110.26469	22200.17187	26666.6898	22692.0825
366534.1365	283037.4661	309348.3791	183917.4671	248354.7935	156452.3406	241320.8364
5415.977944	5242.218404	6345.365714	9545.706176	9472.890176	4595.954882	5990.466305
31009.58615	24555.63538	39786.97283	31586.87429	30168.47979	30821.29886	37567.30225
60215.12538	51945.086	72517.20992	63929.58339	54618.29673	34688.18783	61562.95246
5222.3444	3241.1313	6623.483533	4701.163833	3990.252	3245.948125	3812.2965
17212.97129	22752.11453	16633.0476	21379.9245	19189.12736	21278.722	21463.1828
13684.04643	3532.827769	14833.00319	13348.44608	11587.32802	13005.25555	4991.528182
2914.645333	1877.857545	2699.953455	1935.088182	1467.756364	1478.477333	2427.679538
13037.40635	11615.05588	15465.3845	10968.38747	9862.3694	6413.693538	9440.605882
50495.27317	29135.95256	33425.6418	35469.726	29980.23893	30344.85847	24890.16121
8564.318	6617.553429	7524.519333	5552.4934	7034.136444	6039.889471	5340.781784
1260745.833	807196.7732	1488121.781	1103104.985	825282.1123	684753.2465	1373445.373
1052650.853	803741.4646	1120071.692	1101404.885	908767.0359	687487.6689	1208716.856
638667.1907	663071.6089	562399.075	439339.4666	448230.6703	399330.6943	459536.8868
60068.294	75150.42857	30417.15319	49029.276	37662.27307	53004.1324	52961.7686
13861.42857	11316.15818	13560.66093	13266.46855	12096.29931	8787.68	15780.54046
4436.154	3159.982	7669.654611	9020.648556	9654.546923	4700.463353	5125.692429
1863.595898	1456.78272	1838.637206	1515.935916	1689.434131	1215.709192	1638.613113
69606.301	19293.36889	65830.5119	22196.02333	17000.6625	17257.11135	25502.09063
36330.01545	30567.34145	33360.35767	29386.20986	31107.20925	23312.05583	36667.58538
255671.4449	173070.1393	312781.6022	216175.5077	196757.7416	144389.926	269182.1945
132474.1264	95427.234	154308.8834	132057.0402	110596.0022	90066.08836	154882.64
257911.8782	263007.6806	283970.8444	221379.389	153582.9867	148683.1194	234467.0659
30327.61814	24164.46214	26109.76258	17366.58626	16237.27494	14965.09984	23944.46462
216792.3209	128394.9859	241743.9067	158476.2823	115067.9492	86791.03371	209795.8286
401446.8155	208065.4501	506387.4244	329632.0775	228169.7341	293973.5861	427434.6952
372367.5519	223238.7916	452494.0412	389966.0376	308845.3893	215981.367	400542.3462
255769.5906	202071.7125	339284.8287	235429.2453	186703.3259	160659.1977	296835.8622
7361.991	7407.684273	7670.4	12571.39175	9191.101	8104.664615	13288.9175
35838.26474	36484.50935	53599.49583	37563.69723	31993.1978	35539.65739	28907.04532
2735814.572	1694415.668	1761219.696	1339302.895	1147587.294	851621.4045	1821908.72
17250.44736	12644.74905	20414.914	21319.518	21168.16571	13705.20631	29120.30492
7252.195161	3720.289307	14973.61774	18312.80729	15594.70938	4785.338085	8910.91336
10754893	7656006.467	13384195.64	10438981.4	9277123.372	7275499.108	12431123.04
4754456.96	3566438.917	6159920.657	4962694.88	3774537.344	3238103.431	5483103.12
116417.9783	71374.50385	117780.5855	64534.03624	74874.40096	68149.12983	76858.25428
97747.059	49314.206	123733.0748	80206.19192	71435.09769	95786.96159	108992.6905
248057.0182	154246.3136	314280.6646	213545.2754	188162.002	136590.9372	307652.4406
80629.2905	90316.57843	107415.4075	80232.87677	67278.72	49532.64791	192366.4546
388252.308	310236.6623	395215.8937	440122.7704	439670.2247	367624.5572	485251.4947
333124.5948	276914.6124	542798.9938	418601.2531	354069.1938	294229.74	620751.2769
43343.45681	24336.97636	50486.82191	42357.05174	34995.46475	36959.125	54279.776
61780.19985	42993.34	50898.13618	32488.27091	39705.76709	35718.52836	31996.73425
198272.766	132077.6456	200363.9715	227102.1923	236993.2915	173052.4998	256317.7929

440824.6707	289503.829	687464.33	458968.0711	384160.0865	299631.3847	522048.267
33436.14487	32170.34769	24679.49923	19804.80192	32528.40215	27503.681	9183.72
115816.58	64981.56672	127801.2255	53778.39075	40447.44	31353.75415	121188.9078
192919.1495	145411.3561	258757.123	315001.6074	171018.7765	149482.0293	209322.75
99902.18571	48094.80462	142504.5529	74992.86185	62782.1535	57543.486	108411.3486
341350.116	211593.1208	439676.8091	559878.5931	239881.4315	173125.1884	394679.1273
850223.2018	494446.3328	1059171.998	674498.8633	509189.6229	574134.1943	954313.0259
114294.308	101831.7266	96744.57631	80602.58	71743.30854	64323.88014	82709.77736
15381.98252	11945.5698	27270.434	34137.47031	48398.736	16111.90831	19511.77753
1164748.929	686312.4604	907379.0804	710604.7588	548779.2598	466025.6515	1000842.738
3348689.303	2012759.938	4441073.982	3105701.158	2311054.18	2009472.513	3966353.065
14109.71667	8326.09633	12320.92489	7098.169189	6868.8078	8599.257	7107.196686
7753499.981	4194578.056	9948950.252	6968576.215	5654769.086	4150619.415	9884934.91
112052.5673	95666.01275	119389.2777	112736.5873	164643.3377	100090.2521	112343.1545
7616674.755	4319239.874	10371953.41	7022560.097	5985021.083	4309393.417	9534715.94
145108.4993	91714.47	144809.104	156358.95	158834.48	116096.1039	158586.5229
6056.564794	5071.511481	14288.89707	11404.70029	20280.36731	4365.63	10642.20885
3702731.924	2583834.501	5146023.576	3651294.294	3047494.377	2568971.164	4467330.978
25204.98931	7238.102833	12274.86333	14379.74331	10976.9923	7271.04928	15304.263
344864.9486	235855.5521	442643.3189	363665.6477	283026.7789	256801.755	411516.4787
1470687.235	1057048.515	1675867.156	1639702.51	1399763.687	1121457.235	1726008.87
297797.005	307210.1369	172761.2593	553945.2572	341959.4434	171262.3134	564395.741
238523.9759	134913.1343	297010.5198	234115.4692	169260.423	148628.311	274901.6312
49989.0332	33105.296	76794.5685	12216.24946	33540.05939	27547.41263	71874.40471
356954.0822	158691.5072	406574.0165	275991.5772	215693.8585	173230.6659	363486.0911
19714.1724	8397.692353	30937.59491	22206.68633	12944.1158	9863.078357	17310.6455
5433113.625	5854015.142	5473092.911	5727442.159	7567651.441	6640888.512	4301459.789
2795.546695	1800.957642	2386.376343	1787.679159	2288.529221	1424.294369	3716.8
64287.29427	37729.66778	76647.2901	53706.77256	59675.30182	35925.00422	68891.1918
567113.2585	354994.3583	417815.6649	566399.9825	579754.1385	420217.7913	643741.5911
113380.662	80384.93633	121847.3915	148623.0867	149065.9207	101647.7805	150763.9235
144648.301	82926.54373	223405.6	138393.2235	156005.6129	78839.86091	192035.5103
10809.42534	6414.2765	9689.739115	10858.17083	10060.065	7849.575	12437.95475
381247.5321	184496.3224	533586.8869	373496.7441	223803.6726	185230.2281	457050.5245
51189.7782	32440.07813	92992.00772	52057.7958	47445.83436	36591.86213	57515.44867
24825.93521	18691.18218	3123.954	3831.12	5533.110588	5146.010625	5750.3744
3681814.713	1877909.895	5073148.183	3248113.685	2108283.307	1822729.352	4785654.996
10644.74246	6367.058182	10107.66654	8668.385	10673.76646	7925.288583	9310.25475
9443374.184	6379153.963	11502008.73	9119092.333	7054445.274	6273129.44	10749190.87
2670316.773	1437998.169	3903657.744	2541958.735	1723647.24	1537441.518	3441893.975
3552698.967	1620073.456	2841318.919	2817333.382	2307462.19	2132254.466	4418247.022
63923.0496	43223.62956	90255.113	56965.08316	56199.91411	47205.58804	66948.80322
71267.32867	97308.69206	125079.5105	77936.37067	75574.78931	72517.2931	88971.14667
26600.28137	17068.11253	45573.99916	25362.41648	23868.67136	20069.64667	30167.90551
1260646.64	1133606.589	1489145.009	1599747.67	1468155.99	1719732.765	1382842.56
94561.96438	77275.48192	120991.6226	91928.16431	99291.34154	77993.163	78276.933
35402.79307	27570.19571	45218.25538	32502.31769	29866.25406	28652.05744	42189.88714
72925.34857	42227.81857	64380.70769	60216.37021	41017.17086	39399.43569	77516.60915

154216.4253	116300.8292	227288.7701	134064.4696	104899.5646	94570.83318	147360.9413
15343.68987	30867.15716	24181.37687	17077.08053	17193.034	35401.72794	12533.03391
2653.533333	5090.323333	0	3394.621222	4200.3	5479.647643	2227.787384
73175.22369	44151.79808	60122.12107	76382.05654	75037.38	55524.59685	91914.19431
456774.3799	291940.2036	569180.5994	475514.6096	437011.5509	310101.0118	549811.7534
28409.86942	27907.81457	12692.70297	20381.51175	39025.14944	36026.85269	17983.60779
72790.5762	35822.13407	89106.2941	58673.85643	51687.97237	40921.8724	88471.13713
336783.3793	230992.1618	381826.9901	387379.3366	354159.5811	246212.4452	429541.616
460302.5992	323245.3637	764580.7616	612541.062	503326.4965	430294.6058	664266.9508
125926.9212	68087.78706	156555.6195	112331.5484	93417.76611	77654.62234	149796.2722
186286.0879	112414.37	330260.8706	202235.5825	194145.3079	128361.2188	274388.9215
12889.79047	7596.201786	17666.2885	9053.422556	7609.718462	13843.68	11449.82035
27480.88875	45963.03677	11122.23246	37499.84238	72127.25975	65098.77715	33148.31938
129307.8249	125785.7359	170318.5057	133719.093	114365.8964	99659.30233	149336.5588
172516.9564	140770.495	242236.4	152918.6712	144515.5729	120841.056	176774.4857
15174.56018	12366.80288	17294.10727	8744.492692	13329.93601	12451.26938	7649.115444
208588.1058	125461.0904	131940.0014	180999.1295	184970.3245	140191.2658	201842.4539
287370.1325	173228.4141	303526.9423	195621.3178	217631.4603	208406.4667	263878.076
22352.34462	16422.599	21119.05841	23147.407	19956.88835	16821.49158	21198.26154
492419.1131	276406.8069	476385.0626	384189.4546	394907.2582	312579.8529	468495.8883
28783.74252	17102.3975	26163.45833	16790.89782	18234.2265	14541.12	30490.551
61857.21871	50014.6885	79423.15875	52026.40544	36287.68	36685.982	43035.54657
24009.73318	16792.28618	35878.69745	27991.08078	26787.64383	16320.33	34299.68417
7148.098633	4372.005717	7112.47898	7544.628276	17200.69875	13057.74654	19509.52019
77233.49331	44255.8501	48291.14829	72502.35815	70068.18	51515.99723	104353.2269
615517.6485	384596.2091	849155.5756	566468.2048	423245.2455	367708.3993	745627.6285
59731.5954	53103.97412	53081.62663	13375.16675	15134.8007	53284.5787	9254.348
34412.65989	22812.73282	50752.04404	38933.44339	34501.38059	25353.36109	43995.89167
4157298.935	2737944.567	5608610.846	3919073.469	3053674.434	2743328.004	4961520.445
7687.843071	4374.239214	10938.15221	6170.862	5238.584214	3964.543	6693.433077
7817.446846	6849.242625	9125.727844	6603.736328	5185.544	5897.355443	9224.412353
5427834.092	3580818.928	9044272.376	5652909.21	5592310.397	3601074.323	7295078.216
1642617.648	890467.3407	2504598.646	1727521.403	1217504.39	920849.3702	2134895.699
7285701.75	6269078.064	9347045.298	10789491.91	8358657.838	9132572.663	8789185.251
952509.3862	618842.165	1674108.972	1110381.528	848156.04	638059.1593	1373192.756
117621.5663	101370.0522	103058.4506	93498.90094	89362.99744	83752.63029	113291.9931
427371.0524	364653.54	418953.933	344652.2292	318001.0069	346527.4313	357975.0143
960474.24	759437.9064	1251746.783	1188024.122	1020193.84	1031164.518	1203084.96
7136.012545	12998.976	4165.062506	10041.98462	29246.10229	22520.22792	8116.3988
17320.33415	17884.60486	15602.99783	20932.28404	19449.09576	20557.42436	17042.19598
17383.96234	11773.1376	49979.90737	16787.79843	9724.954791	7362.489732	18187.60162
147099.4191	119291.8903	161632.3194	127123.92	136463.3843	109551.3511	189752.5417
150317.8979	124793.2292	157202.8062	143335.3886	108305.8725	108133.277	183583.2226
253800.101	180270.2639	520749.1644	236216.8868	206765.152	196547.0019	380262.8735
215672.587	159833.1285	272787.6328	280386.4184	165746.4729	165808.4065	208227.7855
106480.352	61204.9215	239770.5738	108858.1622	99975.9195	77234.05213	118777.8244
161812.3121	137568.8924	201157.176	125473.6968	180669.9382	141759.5429	142885.6943
47170.29167	34884.36	63061.908	54697.435	36142.65775	36695.358	56346.41815

972672.3863	761760.5298	1836462.639	893217.7187	776591.5417	746398.089	1077941.018
204104.0694	126879.6536	271044.1483	213259.8528	206989.374	140111.4805	263788.3608
127302.12	109596.7077	108992.8667	130039.5881	134541.2636	110525.9307	119077.5597
11616.02736	6298.8709	9658.035364	10398.95593	7344.311	6361.5926	9215.069091
67475.90116	54866	52968.9825	132498.7096	161031.7955	56283.385	59811.15179
24399.98469	23775.81157	10470.24	21228.26592	30890.07208	25912.89054	19459.71469
23315.48978	14875.15418	19942.3168	13494.5955	14068.83303	9373.968665	15870.93858
30136.43733	10124.64615	16184.01664	11351.41336	14893.03625	18502.45563	20422.51033
25470.01069	22876.38302	29534.68088	20524.36715	19461.3093	16881.53492	22574.1035
47417.59086	34446.40385	83354.87515	56387.85	47985.83218	68388.205	42815.49645
141062.6557	114246.5725	216310.0934	153226.99	144429.0396	131166.6134	175389.3031
143480.4257	93564.6565	143748.8255	112771.56	129842.3763	92343.44065	153323.4857
30449.43091	26902.41538	29258.125	21781.08	26246.82355	22146.0424	30755.52386
3864913.545	2970181.124	4065258.422	3562814.565	3610760.28	3345809.199	3758423.848
5559.013836	7766.38688	3333.374023	5306.702154	13579.18071	10232.18	6782.150101
1134534.941	647197.646	976457.5458	855460.2932	921687.3646	714403.9842	1214110.198
298987.4234	237240.9221	384630.6192	266546.0543	148370.2514	212422.2024	332872.0453
330043.4325	278596.0586	773529.1055	335985.9914	345859.659	298764.0798	363508.5944
771028.4567	421667.8516	1242278.129	744266.292	506711.901	441131.1468	1048167.19
182198.4742	182535.0403	204126.4734	139533.649	125837.757	165844.1306	185544.3321
43397.36312	31392.85815	48991.70894	38222.93238	37802.40508	26352.99563	53899.554
1468.237296	2220.3724	0	785.4043579	2104.6	2928.135	7201.010667
56775.44075	50373.32175	84929.25156	47999.32816	47940.81553	42866.21306	51260.57039
19809.55206	9792.821538	29688.96636	17453.2176	9915.732316	9255.02	13731.4908
73860.612	74843.288	70722.763	105731.8629	52753.7	61392.14286	85113.08875
245233.275	258425.3612	250593.2	288635.8634	233434.1158	200497.8186	213018.8492
326483.1958	195919.8683	344685.12	286843.125	280495.4858	289827.2766	435903.3212
562065.2923	581968.8127	159335.0562	399560.4124	793273.2425	665538.025	270658.2577
1310390.39	1235429.995	1583155.232	1209782.596	1227386.08	1120541.561	1333974.242
1000016.268	1174061.582	1455199.106	744024.9793	986897.0576	941909.5552	822281.8653
2860.017	4062.415704	886.2731429	1435.215	3739.355556	4188.0926	1982.418111
311867.304	188721.8634	410734.1383	271308.5205	229027.207	228305.5355	392625.8779
1757750.318	1435938.04	2291890.001	1597108.611	1610634.826	1423737.215	1933569.099
80535.552	68199.61091	60976.615	77986.48833	75155.90773	66033.47883	96772.0662
2684242.541	2118633.779	2856765.577	2320321.532	2068720.495	1977309.203	2325683.283
162790.7216	107259.4415	193255.9017	140942.0396	129976.3648	125460.2211	189349.3491
46192.55954	51141.678	22164.07917	47754.375	61506.71479	50098.4445	70819.092
167899.8185	99913.233	300508.56	173762.8405	119987.9426	135461.7567	208266.4562
250877.4269	198011.1917	219990.6427	197975.2492	162971.4659	159309.1793	163557.6404
1843849.576	1499797.835	3002283.635	1670229.413	1783122.56	1864012.366	1922500.865
62350.79636	63988.3778	55441.64582	66078.83081	77109.64925	70983.73466	53680.17893
52414.8688	47269.80416	44320.7339	48591.11096	45310.73234	48727.05872	51136.92194
44987.133	32874.2955	51508.35646	45106.542	36819.22292	45040.61815	41242.31011
350436.5735	497394.3524	682125.7836	228212.3697	326940.225	228380.5371	276412.997
608253.4174	536161.1684	509110.7906	401922.2784	462680.0913	492055.2559	473705.216
22440.29923	18811.98704	28696.96347	23140.71864	20415.4751	25453.55472	22164.91933
1606844.269	901526.9373	1237713.088	1104052.087	1274975.253	957824.2482	1572907.914
204902.3668	174680.3975	161429.09	113426.0226	208315.2642	103862.11	153281.7003

56670.2305	54771.89108	47858.80915	62503.99675	55792.81283	67993.34123	58639.014
2368532.31	1331225.664	3162132.014	2003370.868	1356403.387	1232945.393	2822692.117
333608.0642	287903.9133	656670.1969	331898.4139	428334.2685	372222.6823	345375.9561
1293932.218	1017025.035	1519379.039	1329881.38	1211845.615	1066971.963	1721793.227
31999161.25	24648075.25	29747557.25	28097540.2	28790762.18	25233156.86	33645002.51
359799.7783	446341.8903	980744.2741	583266.0343	493242.8793	521652.9561	414340.4338
975960.712	788189.8746	1107198.154	537740.7273	633315.3582	1371045.477	582618.5133
49716.23116	29441.39648	62407.92718	37555.45975	42929.34808	47423.12079	51834.26829
135250.8	112416.0606	222728.1857	110790.6908	203573.625	219105.0895	148716.2857
1830361.182	1567488.504	2448037.889	1849879.193	1915056.231	1666169.08	2430197.057
60713.72258	40454.34956	67333.99258	49196.66492	66543.38576	48580.976	71917.67442
12458.946	20831.1479	22352.25906	17515.93244	17994.8745	21771.27386	27152.10897
447037.1984	742255.1837	799939.1359	380645.3088	410400.5858	631830.3294	579889.2492
528549.2634	393304.4071	630321.9469	498932.8566	444616.0875	403686.6002	568243.6915
212737.7773	172449.0653	402200.722	220655.1667	203883.9121	156627.7794	214992.6734
79474.656	86456.76254	129248.1144	65824.91563	104176.0601	99734.21333	75129.90632
463450.0871	402257.5136	437693.6187	422766.1433	455500.0073	396490.288	467437.7245
257596.9485	63326.51725	95013.26386	59552.14152	73456.25385	69010.22311	64344.25482
2202507.611	1420219.357	2597725.718	1726049.622	1487843.79	1292897.945	1534756.152
3262794.342	3046945.101	5452086.012	3233372.484	3639866.517	3154591.759	3603459.6
4182204.312	4249603.315	3515791.73	3280173.169	3916273.442	3952757.449	2960842.814
19705229.44	17491724.36	20244421.08	19071704.96	18969393.81	17976347.86	21274456.11
197471.3245	153089.6178	250071.8374	199435.4755	147671.8385	229342.5259	138450.7239
226348.4403	175675.5437	178330.612	149193.2055	249207.3369	156150.8537	173978.3496
91739.64206	46725.33993	71120.50062	51908.46406	54062.25673	57772.98667	88373.17029
16192.1333	24050.76667	44892.24981	12388.83957	14265.35757	20631.1917	19377.52769
5384472.705	3397269.28	5843692.471	4633448.945	4990977.606	3806099.051	6587782.091
1156498.632	702665.7816	1087715.58	887373.7669	1058832.595	779663.5341	1073501.76
220497.3703	197128.6049	296691.4224	239895.3407	229067.33	211289.4849	272180.4763
3974.96	6618.725199	9603.610077	4314.7323	6723.114357	5363.96625	5217.217882
12916852.11	7749504.488	8851954.02	11920695.08	10244837.83	8259702.865	12803194.96
24950107.13	22667464.06	23801989.97	25111982.32	25163039.41	23056548.63	34740463.66
151618.1398	139149.669	129555.1334	141610.6551	99539.79663	134753.31	162590.8538
246121.7293	193274.0616	396358.9627	245016.0327	251023.5015	250127.965	243005.6546
227406.27	198224.0002	412003.8484	299298.5502	231440.4804	325012.1076	235416.1129
13815.3085	9908.186667	18640.39246	13388.17583	25403.85081	9774.1745	17087.57115
7696.857143	8299.87975	124543.7535	11179.3563	79862.87531	10023.3	11261.46236
627418.3055	533174.0653	1450001.113	952829.1407	974937.612	874937.2349	921014.1754
75514.11854	65951.31167	125653.4724	98673.21952	116732.3456	58950.44925	104464.4696
60040.97646	56385.11731	64993.36619	46416.58191	50402.79796	46827.59006	51018.4672
1125083.626	961134.8199	1553953.814	1169565.762	1553970.56	1253964.867	1498696.43
15664.625	16652.384	35701.12	25428.50937	28003.64478	19999.05657	25773.0835
9764718.815	8669467.889	10871953.19	8855917.922	9581089.046	8494483.066	11655030.11
5093987.785	5494372.346	6935109.213	5041138.332	4629706.581	4408400.899	4742901.038
190249.2339	252762.3894	268044.2924	202986.8367	203088.3072	367278.6541	193098.5733
127227.9103	87066.64971	277084.2158	113144.0484	42988.42546	48959.32135	106340.7498
2496224.142	1974463.874	1958731.835	2140035.872	1679531.946	1990255.68	1991283.335
6155921.434	5646451.618	9867583.152	6773991.942	7064860.397	6225123.589	8105789.369

108659.6957	84547.81725	114358.6822	134308.0603	123964.6928	98601.54051	113087.2496
6207057.064	5660624.404	9736431.751	6662500.604	6970559.296	6159389.144	8251542.473
2053037.727	824194.8333	1024324.596	994215.0671	1083394.164	1330045.804	1005412.894
2192117.797	1770696.981	3461842.654	2244486.168	2153879.382	1850968.431	2335308.372
4764.436295	8415.564896	6429.855545	4007.346359	5688.081299	6619.873383	22031.99078
63361.91453	203771.8243	198875.6366	215505.8736	204462.6208	253281.6863	304921.3111
5755351.767	5802124.824	7449170.096	6851509.219	7777664.841	6608661.267	6578607.309
1003183.606	996931.6308	1422541.124	783185.68	981006.1695	931543.5465	834973.2418
18802.32545	12834.24734	24613.50322	16881.6278	16853.19136	11388.6461	17874.06879
15183262.31	14490574.76	18022753.42	17489445.95	17790999.34	15458463.77	17673418.42
1025580.387	966324.4257	1443509.74	931250.631	952649.4971	1124137.363	839693.1375
13478.17968	7839.131143	24540.63622	11086.87179	9210.163867	8457.111	18375.53768
89098.19084	71574.965	66728.50461	73269.86892	72961.94556	66413.90001	82877.49867
5024458.072	3823440.128	5933018.212	3619680.947	4041102.921	4215563.254	4467372.024
3850270.785	4297500.603	7074365.155	5324750.643	4867031.318	5771045.431	4565058.665
5640.204142	18574.2854	604.2304688	1655.744819	7472.06	6946.1955	31842.15708
765071.4973	766032.7111	762575.6343	729405.0348	1179294.223	908567.8729	1385836.197
12567303.87	10853251.6	17746141.43	11149168.93	12603080.42	11718713.47	12755676.39
895613.3942	846005.55	1215715.773	702957.273	497338.16	664608.1282	709141.8031
350631.7322	292562.9984	284838.3842	242062.7325	215027.3881	201651.1915	266421.9007
535077.7476	552362.0206	929690.0417	716893.463	640323.2042	843351.4566	568825.488
754879.7365	770751.5538	969892.0895	744044.2082	779982.6493	692479.7078	900366.4457
14165825.41	10325918.33	15400337.11	14141285.09	14516913	11376122.92	17777455.45
27315.76994	43493.90625	47607.73985	26299.82533	30963.17261	31444.55981	28205.57373
362288.5858	316900.6535	799128.9622	466177.2423	526138.9332	418012.4337	358069.235
536022.6683	213832.165	366243.1809	505275.0334	237145.2276	462679.2346	551576.4005
15771107.61	14893230.46	18952807.49	16034875.88	16789847.56	15187666.57	16865344.55
140570.9742	110790.3975	234570.602	149818.9486	123089.4743	84959.355	76896.40544
154108.1985	174621.5175	145117.0833	102578.7618	109783.7125	117322.7573	102995.2165
758.9994	969.2966667	3219.962396	2451.573333	1913.622437	1877.217102	2317.575521
231079.6494	181194.8325	316561.144	177552.7002	266575.1887	197340.1566	321900.3231
184030.743	181501.6571	463422.6344	263068.3065	258611.7932	274992.5888	245091.7043
10349067.29	8772614.174	13692237.98	14657421.08	13396424.54	11820523.88	14623584.78
616168.2273	535524.5826	766466.6058	511849.2379	580087.2184	578116.7564	752855.0554
197161.6428	98061.55784	648472.0518	165968.0543	42759.57769	46858.0419	167049.017
12598456.7	12355361.16	13243150.67	11996865.37	11433002.69	11378199.09	12868838.55
41862.41975	31006.76147	122867.5518	41177.18606	35575.40119	33014.70535	43907.47364
465007.6616	271898.1778	1239153.555	380122.67	88762.41038	101067.7637	296707.3716
344821.7552	253915.5476	865905.7284	308497.5716	90727.65201	90407.23823	241567.8873
9865150.232	9656755.756	12051451.12	10739086.53	10362950.46	9957027.637	12240178
567924.1722	508283.3001	634857.9018	541206.7172	586775.0718	472015.3491	610568.1781
10123933.65	9893445.076	12157064.17	10685295.59	10296969.6	9928356.247	11835034.19
84533.77966	59157.39109	160696.4251	180364.1561	290595.2602	69822.61988	116022.9194
180336.6928	54655.39262	195685.3535	142131.8684	70062.15289	42799.29491	177256.2453
58641.13007	749.3940989	58264.12987	101923.4824	56730.87717	28188.93394	211818.393
437841.4742	365867.4931	662450.5195	429888.4386	417432.225	396585.9645	301495.5814
2378455.233	2070601.521	2244234.472	2104214.939	2161843.328	1944432.018	1934258.491
21977188.82	25059982.96	28413239.12	23813796.16	24720138.05	23713352.93	25054908.67

2569333.685	2589379.133	3511423.675	2067714.371	2049848.164	2619302.528	1895364.763
475295.2853	404506.4531	420849.5168	431681.1834	387587.1676	359306.0746	113572.9641
99362.35096	80567.88417	111106.3914	75585.12935	108552.4134	99249.4876	96829.74731
1654489.652	1590422.991	3049467.561	1381144.336	1955194.268	1659821.935	312026.1437
660814.5163	527208.1869	348885.3071	665057.8153	726803.4441	603592.7785	780000.9038
1285004.011	1253936.121	2497752.704	986226.8155	1305000.836	1314595.491	1035307.597
5588372.761	5501779.36	5748447.142	6232017.486	5954726.797	5812202.247	5963823.149
297969.9812	140737.4122	390148.2544	369066.8378	251911.6465	136251.1773	225745.9841
755618.7771	760429.3077	919379.4616	627487.4445	879985.8678	727536.9926	773580.6781
497149.1446	992392.6504	219733.0261	556308.825	593825.8368	613273.4672	577922.2456
390880.266	728225.6472	473392.3644	425838.2338	776366.5672	743010.3182	387026.7016
30972.55938	12389.8556	45471.7665	25497.12	29658.21375	29286.27616	24126.88
1411339.849	1154344.21	3076637.667	1504318.071	706308.4568	588734.392	1188456.006
42718.78933	29476.02347	54747.19242	60318.03671	42802.50733	31440.2775	55520.93604
36768.46462	81743.49927	51149.45987	36577.23583	45829.5775	44209.76275	33286.55283
928890.9543	838499.7361	1006592.704	672640.4593	701695.3861	577569.1211	667884.4039
56096.63599	61622.61734	165407.3122	74742.32254	78412.71448	74750.35886	63586.09837
2984074.819	2775143.91	3828247.505	3649801.223	3842353.608	3194678.716	3355297.68
125398.2593	69257.60056	303112.4228	106864.6875	34844.68578	39886.70606	102373.388
13301.9225	15840.92618	13760.56	13845.76336	10421.74682	12556.62	10604.34375
357927.3867	375630.944	335052.96	365401.9942	341028.6647	365866.0956	386612.952
9450666.416	7107996.236	11817341.81	10660316.43	10963473.72	8517284.825	12843668.77
1088865.61	1109518.013	2835595.59	1688136.349	1823460.743	1774763.022	1927599.856
1581082.194	1419858.318	2054902.49	1755215.32	1979970.318	1607820.322	1591965.341
15701.52332	14680.80352	12557.09517	15441.6018	13348.54156	13889.81169	11421.22337
1267503.945	785764.2092	2187481.73	1335912.902	1224234.504	1157162.811	1227160.742
274082.5165	253630.3215	467110.9018	303954.2531	350949.5911	252378.19	305690.0535
84235.26343	87862.83	64837.40909	61157.65192	88629.2295	55841.28	90568.36139
1374014.447	1572186.512	1480291.781	1323821.515	1346315.916	1492965.135	1176587.514
385977.2487	333030.7658	697494.7097	394440.7182	207226.3454	191163.5378	329451.8037
7580226.149	6974130.562	8281089.593	7371419.759	8053332.628	7125536.765	7904103.003
4155.725353	5289.509943	12234.09306	3259.295	5079.228765	4321.033929	6241.974917
2356272.387	2251347.804	3392393.26	1754533.118	2529430.637	2071406.819	2603417.137
13049801.67	9614974.324	17360416.46	16840557.62	7071862.425	14631833.08	16388023.61
3325563.943	3452301.779	4487802.687	3563848.005	3929702.446	3386110.532	3307675.22
583062.975	616475.55	1318971.427	784085.152	758327.5864	780483.1679	637032.2794
2974.920667	2508.954	13936.99038	4513.951929	1623.688182	1667.9808	4746.4362
977729.1792	892868.4668	1352228.782	943426.2763	934040.505	867129.2004	868396.7593
12464826.64	14032249.63	13322068.63	13716785.39	19651751.84	12588215.86	12416317.63
518301.1761	469021.0067	562279.4202	425098.0151	427963.2047	430690.3181	400345.0185
443356.0092	522729.5123	495572.88	293501.0014	508711.8907	587189.2467	240058.416
1058703.196	656868	1975588.115	816549.6098	269240.3817	307993.914	830873.106
961361.1442	921707.0471	1269171.521	1195961.7	1230482.641	1114066.68	1045468.882
771780.0965	775600.7692	1033724.542	548490.9756	658603.137	727041.2667	532384.05
1161154.637	571584.1322	1112647.904	329415.1829	362635.5117	484316.4262	543645.4757
7143390.543	6596007.527	8434305.857	5207477.936	6782936.703	7078892.535	5133888.068
321177.2897	270215.0103	454987.7818	290860.7878	356315.2129	294773.5907	261040.5917
22531.80944	17966.35556	24645.24084	18080.84106	25374.23636	20159.12567	14400.368

3631686.96	2634792.951	6930707.076	3497782.731	1457042.763	1337348.137	2929223.024
94420.18482	113309.3199	63345.82154	95104.27222	98546.67502	95989.87463	93234.94956
5865524.72	6124383.785	8311386.643	5470670.737	6496646.239	6989247.53	5126228.777
64255.35077	65788.55043	93871.615	69865.45983	74094.96479	74236.91771	62680.91377
55160.12446	40725.75013	89346.19452	49383.15457	55611.91504	39610.319	46630.61459
9469307.346	7072540.315	18187206.9	9274348.793	4450254.718	3911296.498	8077827.712
120048.1416	75104.23885	90437.2975	69889.06629	114656.6154	82386.08	128725.6382
914009.7968	799982.3055	1140048.349	864949.2421	853812.5662	1119453.844	684247.9057
245683.7513	176336.7831	370077.3013	255221.9663	257201.2009	231894.2077	233295.538
77247.86127	72154.95308	98766.64283	77571.82225	81224.8773	80672.99334	79283.14364
51139.30654	51932.18008	99534.91541	53407.761	37018.2275	46004.189	43173.43777
59694.97	49939.83825	141973.1209	86241.78923	83483.12215	97077.826	67599.48
100443.4479	45181.11768	252951.6484	68700.66118	20785.80652	23831.2381	76797.13724
38506.58164	39288.76481	31614.96195	34174.03829	36233.47357	29803.3425	32315.37824
3677403.263	2685648.367	8333385.553	3393255.581	1713298.612	1446754.203	3298132.932
286231.3912	161163.5765	653273.4646	243284.2589	76520.45785	83517.27429	254796.2283
65961.74792	70478.82862	56223.90729	61399.22562	59408.58943	58258.558	60142.55579
283438.2627	238564.9377	284889.9968	181479.883	166953.092	156084.6337	175262.0393
326288.504	265512.8302	498245.317	355189.1535	327420.0834	301880.738	318822.3797
521726.9705	363984.5151	1106609.617	489628.7986	181837.6919	187348.8885	453139.3189
97906.7568	68296.8402	138414.8411	81519.50234	47163.77953	51553.24827	90848.72
53493.70147	64362.50838	141892.7967	84871.78292	167554.8289	82878.79138	71454.51243
590554.5219	440911.5855	1060047.835	560185.2282	264591.575	251816.376	529423.9552
88731.51138	38872.25679	73222.92392	57292.13673	33203.43953	31857.94941	48840.71972
31083.85625	23686.68547	105273.7736	33094.78132	40651.35669	55553.94218	56014.43161
99760.47971	89327.83708	104410.5384	76107.90862	65443.595	89834.92264	93245.241
53937.06783	58284.29855	72812.00232	65969.58736	71134.01393	75529.34987	97930.66904
336866.465	571077.9723	450833.0807	531774.2093	610284.4116	771931.0923	377994.8628
13765.79012	14653.89067	58829.63	23734.13124	15629.23735	14028.28408	22878.5659
260370.3375	622912.4797	301625.5385	355918.4726	438908.0459	526076.6723	294411.4519
2785022.493	3082320.944	2141201.156	2809396.526	3004326.558	3134784.516	2637993.378
3258697.729	3110150.073	3419779.383	3527470.133	3980282.846	3427730.804	3162701.497
57083.2964	11592.02728	159384.005	36399.61024	11233.79639	30118.45816	54376.751
9765.460972	9215.722023	37855.74589	12666.84437	7784.747375	7283.157515	13577.72091
182569.4656	188491.3876	162457.3794	201593.043	247654.9757	218196.9374	199061.7272
27057.98475	10254.18055	88132.8438	59985.57936	15465.1905	21906.81404	21247.03473
682424.1677	668043.4834	1422460.291	639264.0301	697219.5666	658136.9046	805865.994
323759.9103	446307.4442	530.6903076	275707.8088	368149.1478	439707.3466	281997.665
555166.8092	526887.6046	713683.1443	676963.3593	691194.7052	622294.1554	592036.3915
301236.6565	297347.2583	217007.6278	290538.3279	349807.1172	317111.5474	256594.8574
3031.925143	1855.888364	17910.44475	2375.53	881.7335714	1715.127841	3765.357056
2051279.121	1932881.833	2168555.117	2490504.55	2761668.271	2404939.574	1933746.269
13439.32753	9925.874756	15778.90106	10508.58054	8220.737965	4178.29475	11890.73633
116564.024	164823.3917	94449.24357	85935.95246	102995.5575	111987.54	88460.52
71333.07513	75099.7376	55165.40615	62858.99998	62657.95371	111892.8345	83004.82989
50238.93061	138459.3984	63135.13394	45663.13206	37956.54842	36401.6496	26308.80509
8345317.902	5507457.327	18867655.61	8290871.621	3593540.642	2995754.402	7766136.553
87977.67016	152762.0207	253971.3192	188879.9019	214157.9401	209207.122	165101.0414

88665.04284	81451.76158	44835.1325	56013.99845	73354.45135	77057.61895	69084.93439
73840.23715	57439.77332	136433.0727	73377.22352	47537.72776	46837.04484	66722.43875
157321.8293	151588.8143	222512.4357	161127.1558	204096.0069	178030.5565	178348.7839
127504.0897	45031.37563	331420.8401	84445.95626	23853.35467	30097.31714	108124.3411
94026.46361	101241.2989	66481.36	137403.3512	68970.69525	88178.982	75238.72958
35723967.53	25600507.75	71764962.43	36528459.6	19186298.75	16319405.33	32836782.82
638819.6874	690195.2955	570892.0485	616033.6921	604022.1983	661197.0733	575888.7113
37174.71758	61949.96418	56429.01386	59786.80609	70270.21773	87401.4	48550.72727
124756.9006	78319.59979	230203.833	115817.6722	47528.16374	51332.99093	125636.5285
118123.5126	87561.10183	230293.8513	122669.144	51379.86621	39699.36964	129237.9946
616247.4301	318660.9391	1304050.625	519541.8004	172363.1615	188639.3101	571847.2494
113088.0441	67333.3422	88888.55164	143870.6728	70776.34406	59600.05649	120470.5984
298102.0548	305835.0137	216238.4564	268880.738	259473.606	252070.0401	266020.0264
44168.4243	22966.36364	226135.052	93099.23435	23486.35764	50931.75121	104287.9848
160949.5827	184162.8074	74278.24398	119375.9715	110216.6173	163380.9256	135334.6763
248413.3367	240999.2057	258204.1761	223389.1535	230102.3026	197089.0535	223281.0285
19960.30115	19331.53641	42631.94658	18335.19909	16158.6408	18831.61041	20662.855
4193725.309	4277609.289	4578699.284	4663179.704	4755679.774	4739672.027	3883458.176
119712.2023	50264.16021	312547.8457	90805.51385	6409.225357	15410.59951	121746.1216
62241.03434	52708.02083	94011.75129	64474.39982	32199.91772	33209.18127	62453.27229
367933.8195	271224.3953	655536.4626	348740.6251	176862.2602	172506.63	338872.7261
205933.492	58631.55075	769543.0831	146211.6179	45215.19179	34332.59758	238652.3838
13116.75332	10984.47144	29147.69854	13950.21187	12125.63231	11288.03966	12861.62155
12023.61462	11013.336	28371.91	12922.52475	11714.54682	11075.49545	12329.59667
7914.297455	15384.92517	13447.10194	17428.724	18557.11845	16555.55896	8219.713211
90271.19181	37419.26719	165952.4869	104557.7451	33671.06848	55540.28663	84778.37695
11542.46775	13172.34507	67856.62894	20281.52134	11187.65889	9642.413769	21884.77387
17514.65447	18321.47711	16907.93151	28226.08583	94397.74233	30112.92731	31067.6632
281690.2818	263784.2589	248384.3058	268638.6383	112001.3083	284498.4473	291961.1127
435406.5364	231010.8109	951207.7695	395901.0462	140317.6543	126630.8416	406619.0163
5087.414769	4315.667053	34646.493	6860.426154	3440.955933	3193.570211	8798.934158
255813.2388	150341.2042	413699.5693	223986.3777	98625.16559	92697.82214	238782.8266
14263.3492	15249.65808	19142.82011	12903.65149	10089.25666	15668.48222	9670.439331
10674.91822	41255.78641	156640.2081	63389.09824	23232.7516	2138.5133	15502.076
135280.5281	146980.2708	268709.9846	275032.7193	229261.83	110769.2558	262185.8213
19912.25586	18521.02478	33610.66495	18246.8219	20447.44168	26677.06062	14430.80116
7383.3474	20954.89084	55948.47376	17371.18857	26371.89309	30931.23545	11896.76
24654839.7	16735768.84	41065538.82	22076673.74	11062225.73	9859250.402	21837566.82
175361.5683	186424.1086	148899.15	172790.4141	172787.3312	181440.0924	164528.7315
157294.5096	72782.16295	502330.2087	139678.7428	44198.59076	53728.26671	199053.9218
22041.54527	6210.139	68029.29192	16072.78702	2730.758	6012.604719	21835.97122
270361.548	195208.2199	377944.1131	229384.0656	145088.5994	135926.3465	239637.1909
23688.15043	23084.90545	39825.80018	23774.17536	24111.26491	23129.54057	21262.4607
186987.4612	162618.2394	105850.4553	160481.6222	160115.3699	160051.2171	147365.9972
551446.673	262365.0887	1289839.712	477009.5262	144948.0859	165267.9184	529619.3592
90913.5395	102167.6295	155794.3898	66713.60222	66239.51937	67391.38637	65420.62577
376969.2795	372325.1705	220328.2234	316471.8925	361428.7338	358085.6073	305963.1102
75124.83355	91213.39044	46761.75234	64697.75209	62097.18272	81765.61162	69561.18205

20673.59268	21119.37805	22756.8	19925.7314	15459.54557	21079.8102	18497.65364
191200.2972	73011.40558	368420.5511	134567.6771	63503.4593	60923.83758	307980.917
19260.38098	7481.996338	69109.02334	15301.10851	6842.082901	4715.680496	6517.44
17637.45245	7315.950427	51116.93018	13153.01953	10311.33942	5618.150647	4611.6
24812.14256	9032.408203	58802.62763	14023.3973	4948.564697	6146.060776	23417.91518
78683.50745	22339.01576	24381.83689	18586.52169	15409.99929	13919.05231	17370.62223
107438.4924	37995.53547	313314.2078	69604.78995	28655.97582	22657.14182	80947.06803
280141.84	228811.6	369834.96	256180.375	173283.7013	157185.8096	244289.7267
243770.1295	155422.0952	414594.8327	224797.1011	123842.76	112708.2888	244271.8522
52054.81543	43477.54391	112116.8671	31233.06464	31069.3484	30493.08036	62646.14789
518726.9836	156780.0376	1680629.784	381445.7178	132989.5665	87425.57163	450883.7263
252192.4131	203858.7295	249098.9676	218673.7252	141085.4935	142029.1137	213784.3923
440816.1985	337696.021	828356.4922	485673.9201	273739.4309	206670.4216	523659.9191
12185.25064	10702.06145	17061.53613	18894.37688	18179.20673	13239.91338	13801.17387
863900.2625	293632.5756	1409844.275	468516.3723	133685.6206	107162.1416	549290.4362
5430.748901	4098.016767	21472.59045	5211.488861	2832.032776	3201.149111	6955.867571
45242.94902	51866.304	46895.43075	52722.50149	74862.359	41878.4625	60697.51406
4452.740987	3741.967686	21086.9625	3848.437286	1934.085533	2691.985082	6569.375244
54106.6004	60192.15363	152318.2677	82867.0664	71248.91748	81302.13135	61252.05199
373163.741	223489.5327	603747.7384	333077.4252	148243.2052	142880.6518	333374.4645
734.8707	1657.178356	12214.92235	1587.4875	1419.43139	471.8405	2591.998657
83570.84831	42982.064	153529.1801	63627.91868	46945.9277	23445.76985	81667.34385
30613.93386	11806.70881	85777.23996	19437.05906	4993.827194	5393.175681	22863.16496
34985499.92	23195235.69	65303488.2	37038768.8	18088587.87	17064757.2	37723291.11
518474.3187	711059.8908	366279.2895	707886.9979	672205.5566	616929.2521	476955.9843
326278.03	269271.1965	292098.5832	348670.0149	225194.0132	228087.6196	435138.6724
364194.5363	356664.735	240468.9408	340083.0908	300682.2031	333656.7717	347167.1225
33599.7804	41522.68172	20750.20942	111144.9733	31736.35127	39555.4376	38695.545
244839.6705	96726	332218.2119	225036.5196	148630.7573	142019.4056	231517.5502
9698.151794	9886.314431	8076.407694	10323.16386	10847.02443	11475.94995	8245.000721
197835.6274	104109.825	355972.2153	176266.8191	66392.63431	92287.0395	182979.5721
66732.27667	75742.7542	83134.44943	83680.8854	67459.87888	48628.845	24513.55833
41029.24	28604.331	25561.8	25826.87045	14916.5028	25469.30831	31601.45055
267765.1651	86485.80462	807872.3698	165783.6912	60394.61598	49562.02393	232296.4175
427647.2078	289752.257	561310.1037	377853.6775	186053.6269	187824.3679	370212.5635
53550.20081	53238.31238	106259.2817	49197.288	48976.34119	40172.82682	57995.19588
107887.9183	26737.16515	287293.3693	47375.853	28983.84595	24005.78652	64105.1581
110487.0954	39235.01143	310487.7333	66568.1768	28937.71927	23588.33407	95174.33802
365604.3485	141277.2377	1084332.812	321561.1598	99129.44336	78822.41854	382448.0313
1923.855714	1646.502686	3148.544857	2347.178077	2671.445385	3176.898462	1950.317182
117934.8624	73904.82766	311686.7597	113784.383	48774.14464	51175.96785	119438.8004
58390.84842	48356.39681	73623.64502	55948.02339	32390.12223	29694.40091	55568.6627
352595.592	205914.7653	641105.9722	331515.36	156382.1161	149122.3732	381966.6704
47140451.67	25362112.98	79155846.28	43280033.87	17902948.27	18586231.55	49236403.43
388744.2522	216890.4442	806644.7718	368336.1438	167206.2306	145267.6457	395747.3336
30130.75531	14292.70219	57117.15906	14027.80385	5811.678864	4570.432078	27512.22095
565163.343	433792.5232	1757165.47	939048.8807	452780.2636	165480.9536	1013553.774
18513516.96	12276517.05	37537612.88	19181956.03	9997978.73	8240713.771	21325019.71

500758.2589	221223.01	569860.7571	410922.7675	376997.3733	273906.8055	455415.4176
462676.4707	410792.6527	486981.8048	698537.623	398016.75	366307.3429	372480.9959
1387038.413	754148.8492	1369509.68	1008618.788	1014079.489	850291.9928	1063207.076
192802.0918	138664.0724	264356.2286	198604.56	122532.8186	125141.5165	218633.1539
370362.0726	97008.4455	1054785.878	297734.4619	131398.8317	64133.16857	411391.4935
518566.9767	370141.5402	306623.9744	143406.2365	357418.8154	91582.928	138022.0057
371222.1586	161701.1435	657476.2725	655492.0715	167790.8137	143906.7239	804990.1017
41432.625	26451.1128	64351.72142	45556.6167	21061.61	13615.73926	48537.92667
390272.4478	165051.1935	831307.3161	340910.1124	140679.9773	149976.0765	372037.0705
71901.32905	30533.89809	304275.0745	65177.30679	28434.60244	24743.1239	78231.4887
101448.147	71220.11493	264780.2158	112640.804	36112.71567	30841.01274	94055.38398
193325.6705	160814.3995	182374.4624	188999.4572	151280.844	150943.3126	219023.4154
440028.3752	126272.3026	1369745.907	318707.4141	88895.97375	77239.27141	363221.7333
62675.45562	58814.17842	57316.55362	73017.60225	50310.1911	48564.5715	62436.97206
345886.043	448990.4965	414556.0385	344009.0707	166552.2618	128026.9916	313484.9273
101966.1683	59728.37133	23778.48	96904.86385	19157.38073	20945.04069	99447.30011
98236.02193	62594.24718	230299.8323	97070.06951	54271.34533	52109.45668	17450.70909
8789.546204	7813.385324	6725.771057	5317.363525	4131.042857	3281.540955	7681.585854
38877.29221	33572.1867	21958.29396	27515.14107	23123.30867	27168.45967	37186.34521
230816.2105	156581.0514	473611.6	283593.1	150387.1629	203570.2677	269683.3904
18198.5193	12741.74134	15841.39917	18414.89264	13570.11537	9380.171769	16930.9035
42874.24228	42138.75325	99839.93758	63422.84991	40599.97089	42889.32781	55768.31669
4686.929206	4970.683167	31884.1785	15307.91857	3537.121571	8126.884575	15443.95174
31432.08995	21524.92232	94562.49619	30964.14723	16842.05025	15164.29395	30353.05824
6283.310669	5871.555481	17794.44141	6141.06901	11152.3061	13735.68029	47135.48434
19510.9026	40822.53973	48312.47042	48805.32562	45690.50157	43946.60326	46314.89293
7909.803813	4981.094666	26790.65944	9074.783936	4826.437749	3702.879155	8476.342773
468987.4965	415340.2221	565346.2825	521967.3058	393123.7924	325735.7685	569037.056
9768.291611	8704.380611	14598.54435	12182.13506	13148.50442	7420.814647	16675.65261
159127.6864	162921.3316	253562.6616	206015.0705	176766.3466	134909.6898	251835.6796
141691.0054	105420.178	179919.6709	111733.1108	102468.9911	102834.2995	153537.2408
13784.54153	11737.82376	26197.13416	22127.34342	19784.10832	10928.57939	19226.6712
21417.93317	21586.4919	36690.57317	25897.08315	23403.36935	17912.94	36389.14182
11443.39827	11955.05788	14386.04	12492.31263	12515.06278	9429.399091	14816.89912
221241.4238	251253.0424	283222.6629	262096.461	235992.7994	168981.3167	268380.4642
223355.3593	224202.3402	360474.0234	305781.9485	239342.6455	170760.7911	326948.1566
364096.0393	440537.6995	652740.599	464926.7738	385569.6276	345696.1757	550065.1838
7305.007538	6242.645357	10655.58343	6131.849545	4699.887833	5363.645455	8614.623846
14035.82476	13246.5615	22679.956	18209.94462	16504.72853	12950.68841	19832.90976
106748.6129	109386.2107	170562.1534	107245.32	109076.632	69309.28818	141132.9694
42661.43094	40440.99335	69167.68031	42713.45365	43487.90929	33552.34911	52900.38741
8080.278833	8552.073833	20414.06773	9698.971706	8433.142421	6433.921565	10579.42111
27650.871	26069.39629	39285.53475	37127.21965	29065.90238	22468.72556	36061.93474
50318.90979	49233.12318	69486.20955	63580.90875	45620.52379	35397.345	70967.6695
16017.23406	23922.1126	27602.73635	18494.98371	18242.68379	14566.30365	24606.96194
49312.92529	39048.37961	48376.74176	40093.62478	39540.67044	36807.516	47524.29374
4932.946941	5336.635793	6067.279059	5325.962944	5233.031556	4342.577167	6525.955824
2395.33979	3667.619867	4554.179	3874.492224	3551.725892	2271.275692	3157.096489

776444.3241	815836.9023	1215206.848	852979.3493	869336.9956	576521.2114	860245.8559
28675.16135	32870.86729	45618.14747	30859.56122	35017.95194	28829.74033	32407.23006
145570.3095	114524.3309	230849.4628	137103.1835	155744.7898	104389.2872	171478.6286
1516.798077	2170.9754	1467.318514	1533.095101	1839.704655	1582.192766	1375.147613
95445.927	78033.94558	92174.41417	86446.35261	81830.31163	65072.83368	97268.25416
1297169.691	1346791.911	1641913.707	1392510.865	1361059.61	1231316.299	1303982.398
6992.571	6366.895688	10109.83056	7550.815667	9831.871333	10488.972	7312.901979
45329.102	44620.498	66243.41724	55763.76081	94621.11047	47196.73328	58742.49222
39475.84192	39187.85766	36005.68161	7261.926962	6530.362091	6415.668188	4767.359471
25225.57596	26212.45454	32896.78189	29414.92467	30323.78329	23379.33496	28940.75738
15212.47863	12884.11723	24532.9425	15722.58348	17484.18874	22632.88777	15090.98095
34127.79829	29765.91676	32117.199	27082.725	29642.66547	20842.65053	27496.44906
1062689.232	1166022.585	1132935	1040777.997	1059694.047	877216.3456	969494.8752
973074.3136	990662.8792	1196127.16	972628.3491	904609.7038	744190.2669	893943.0115
2397.565895	7151.781	10090.92424	13245.58527	15412.705	10911.77293	12437.11313
140179.2699	133196.8958	130952.3417	97844.22635	125910.1058	73763.59681	131612.2421
27050.3048	28695.4556	30033.39835	27067.54653	26260.038	18740.54972	29569.80032
49483.233	48618.22154	58649.40703	57669.67021	48099.18964	41086.93952	50068.30467
52730.4036	65191.33606	55934.3052	60958.52318	63010.35519	51515.97521	57195.32
4193.19875	2790.450808	5164.992667	6530.268539	3540.373132	2239.655	3510.389
6886.88725	37860.90438	43267.367	55816.04063	49972.86056	40094.39012	74482.29069
6480.509	8983.222588	6374.743286	5290.216313	5580.585	6667.963824	6056.980941
259476.8884	249485.3054	259805.9468	221194.6225	211236.6346	173385.2161	215320.4187
16536.22533	15948.9365	16880.12184	15921.43371	15136.20033	12475.2015	14800.39519
5399.9045	6108.815	8392.81666	7652.7972	8456.53075	5636.662263	6979.8372
8643.892909	7661.093167	8272.256937	7987.7375	8195.033	7861.2765	6410.95
12720.25959	56016.59929	49796.89535	65492.50293	55243.36779	42393.71541	72408.37412
10267.45753	9617.365448	19972.64153	11597.75757	11192.166	10217.01827	13828.14071
1231.428444	434.826	374.107251	884.4223846	764.1472727	367.0159302	259.9939575
8194.408444	6656.7325	12276.73853	9049.629952	8011.833353	7039.967875	10052.8
4924.164	7179.836478	9917.543741	6859.239814	7855.124518	4127.842529	2986.509231
7572.578824	7166.875118	9310.427438	7350.943306	6099.744737	5924.241	7901.64325
4016.645176	3429.538714	3803.308111	3884.404526	3874.71581	3578.703273	6100.74024
9641.99775	7558.208667	11404.88267	8711.903471	8666.237222	6856.429263	11306.74417
38493.57153	28755.986	53792.32724	36781.16459	38637.21983	38909.90639	43264.88978
1032.537767	1061.605	855.594905	1017.670219	563.3915863	553.6647949	659.4934387
59065.01863	49988.2955	80814.294	57044.689	52796.28694	51695.65133	63599.3
10375.77475	4967.693471	8012.726235	6307.4115	6224.486625	6426.689722	13998.90958
64397.55421	55490.68306	92613.95965	57395.93224	58985.838	48769.39429	65751.55753
7604.566526	6349.810049	8188.728128	4223.035412	4578.451125	4427.8416	5012.454824
47889.10663	30894.38118	64368.15956	40961.62962	31938.7122	28008.0205	55020.65794
15207.9025	15484.00031	15580.23433	13926.36309	15390.68385	13930.61856	12867.07829
11336.05882	7652.622235	7558.014937	5359.343889	5086.560368	8736.83	3585.955952
7447.866493	6614.993857	8172.087	6103.389158	6901.542211	6724.628711	7219.294333
11433.34694	8494.549412	8608.379	7557.914696	5882.019636	8833.342824	7183.60475
28287.23657	20835.81887	41404.34193	18961.302	25364.46557	21556.9845	22364.66087
49077.07306	41537.89382	66688.21176	43781.54733	43284.49012	35286.67765	53305.41833
5113.202708	3665.374737	7294.684333	4610.15795	3560.022667	3056.469667	5326.592437

1961.436354	1507.425328	2479.085919	1709.784802	1918.299527	1495.190031	7090.483722
57290.31953	50299.34541	98606.53065	60817.87476	67070.88119	64754.677	68334.09688
59955.56226	49148.06325	58264.80276	48258.27663	51624.75221	65884.45269	50620.26255
39371.84067	44750.64133	31145.37882	38090.43124	46362.50129	44350.34818	31631.98165
6834.068473	5539.7244	6508.892133	6233.315453	9701.707944	6273.435267	10510.43369
354259.2271	306878.8822	352503.3786	303325.9989	314933.1791	313750.4321	297993.098
9977.105794	5722.817773	8082.865875	7732.704315	7989.647235	5493.676769	8255.898905
54730.6616	16663.25568	31093.75706	18211.97206	15898.44273	18516.03963	91686.38453
43187.85785	34798.71111	32787.42827	33506.89935	28084.7732	30925.00447	55877.83178
22895.53887	4774.950158	11671.85594	7993.446	8015.608726	6846.478233	34039.82965
6477.926895	4576.489449	5750.509566	4838.835867	6129.913218	4745.198971	6147.596
152310.7885	120589.5457	164166.4876	138130.3533	140611.3152	126725.6311	149882.8022
69598.24556	66029.47312	136456.9837	68291.34311	69773.33989	61323.55888	70807.53
11245.80449	5253.12	8428.941684	6515.183684	6119.66	5153.704	7308.162
7782.533467	8773.630111	15028.57644	9444.842	7277.043429	7587.715875	9706.888176
11183.26474	7701.524889	10184.6725	11396.60995	11288.9845	11464.5888	9508.740111
43112.42508	36938.66216	29851.17462	23004.15878	23296.48235	21118.01632	25847.712
13719.06958	11648.58308	12722.81948	11321.9679	12075.00091	11944.30552	14364.64191
68479.85365	61868.46176	47995.94365	60763.42547	78391.32863	68238.45	66881.83988
64697.91359	51720.16578	62554.195	56406.672	68919.69005	56783.804	47497.7725
13779.22724	12024.59844	15710.60163	13763.08154	12984.52829	12777.06767	14874.70705
133397.6384	182583.8244	211839.8387	178835.5781	176548.1676	129008.0098	138776.5414
45801.48664	49711.10196	45711.86483	45041.29512	44772.8557	40666.47	46878.56814
240774.4483	188100.5566	444470.3984	218886.2801	210089.648	208621.62	344107.7477
21421.1305	22034.38539	24892.54331	20959.30571	18117.89806	14817.70673	18712.0765
20657.21739	17400.7375	22485.60239	24142.52874	23403.90667	42305.76375	20109.75679
544964.4143	556125.6544	686684.7005	546942.7693	574915.2329	512771.204	573221.1878
842049.3026	616874.975	1119144.629	663829.2654	743520.6295	751876.0692	678433.4095
5527.022789	5101.094118	3759.817286	3013.563462	2124.917778	2683.5894	2279.231111
10995.64429	9237.220626	11343.6777	9502.826553	12287.5005	9467.149579	10137.44249
838629.3447	726214.3069	1016883.042	786090.5781	794251.3648	710508.2079	915376.981
22613.269	23272.386	47134.80981	30368.8815	29609.39688	30334.32188	29178.67356
5792.913388	5028.4408	14104.34616	8211.636	7477.104492	5054.496812	7610.520393
5003.751211	3705.014648	5654.999824	3115.726667	5587.533206	4910.538261	5581.047468
78063.00065	62362.13941	133577.7263	77895.621	76007.14253	59365.03417	96253.74847
98865.84235	96355.34059	199576.3938	135740.6531	121779.058	109554.5889	149499.6214
560980.6149	434461.1672	768836.8911	530958.6431	488239.124	474613.928	605338.299
495305.6448	436343.5498	892502.4834	469782.7687	509050.1901	528262.8238	501627.2029
1241.436857	1391.090706	2639.8	1265.616	1785.981407	1656.9055	2064.453
127057.2219	122741.4937	5288.787579	1863.1548	123827.0815	1625.286	1455.661444
41829.45995	29226.76509	37266.62211	28981.81089	32528.7508	25395.19967	52883.04453
242398.0726	217142.608	328799.8364	231215.6706	236834.9365	257413.6135	261223.256
179790.3889	222399.2039	178055.0209	140131.8068	176591.0541	227632.1215	135936.4738
77742.65606	81197.69188	76030.56452	74305.71223	84251.69571	102226.4157	63385.11625
15882.28881	14377.86565	20750.85441	15213.39406	16107.87806	17133.895	18269.21088
8106.480421	7451.583632	9904.675105	8365.7475	10006.69937	8960.871789	7242.9835
126989.7427	118543.7649	143868.9035	114396.924	114228.3672	124420.3431	113949.4571
8166.994333	5957.7785	5584.090111	7024.746167	6114.0376	7333.127294	7494.842

41200.70048	41659.1009	45070.20169	38346.11785	41765.4705	36128.6495	39776.92208
3364.751167	2659.721649	5425.794474	4160.269833	5524.735125	3128.057775	4936.636286
4663.479913	3304.584725	5065.382102	4204.61018	4985.986	3263.578438	5013.405706
41714.49143	40943.12743	61084.86088	70909.90395	34557.2381	39401.9078	33124.48425
1192704.936	1147370.248	2007566.248	1215026.728	1264035.985	1350649.529	1194375.385
6085.507714	7457.275125	12668.71536	6532.422	7179.145882	7243.242222	5974.837895
6302.880835	4500.018	5514.837625	5480.22975	5742.72	4242.362305	5458.293647
20431.84091	12631.17241	13175.96373	10211.42711	10008.29537	11181.42319	9506.166412
2911.092496	2746.3265	3803.093357	3054.742022	2817.192143	2530.624131	3951.71325
70242.20871	62674.60583	94071.48917	68790.69428	67887.32456	72004.024	75128.43929
10452.38428	6245.474563	11108.36203	8603.512688	7732.216471	7713.860438	6436.107118
24477.87505	20453.1635	19536.51084	11861.20628	11471.98072	12489.53785	13774.04368
5206.255655	4782.733107	17590.16325	6004.532155	6916.836479	5216.25487	7043.23213
610327.9387	607845.1874	1388406.32	772932.5841	753479.6569	800948.3302	691312.9073
148538.9443	106296.464	195865.632	128124.0437	117058.488	93139.88961	145778.0209
236039.168	222638.7777	414692.3277	248819.9433	228781.7883	240614.2602	234029.7933
50308.70897	47765.464	90471.29472	58077.60523	56672.70272	43097.21221	56408.77781
9156.40875	2117.451225	7693.967235	3551.729248	4244.9125	4115.053741	11043.11631
18673.03247	18131.07376	40075.23617	19888.46675	21821.27681	20152.55435	20426.94035
5673.754667	4017.9285	5521.6	3711.281857	3849.2685	3762.483846	9823.991355
20710.30994	16407.8288	29346.99272	19934.90168	19605.62938	16005.55465	23786.32923
23049.05633	13197.17084	10757.721	33311.16784	37238.87935	32595.66342	6061.079063
18066.99513	12568.53777	50496.792	17795.32943	16537.90287	13749.65604	35426.94273
38806.236	33020.21319	46903.1292	50201.92727	32141.19073	33573.426	40314.70387
111877.2164	37793.76169	59331.16882	52775.19524	50189.97911	44449.30859	77817.48565
34737.01772	29095.07906	45236.29394	40465.67294	38129.39	31542.88	56553.8337
883937.9099	1036418.207	1107426.571	971574.255	1080996.891	1013967.509	1287734.168
45473.24188	67018.81865	25767.67256	40635.04419	84035.18275	43133.50644	71084.90513
132359.191	151233.248	178215.9803	127535.7635	173776.9863	152665.7443	134499.8733
353497.8456	346518.5308	435553.5099	342505.2446	351137.2995	313804.6104	362493.2665
58021.847	47544.00129	97744.41833	84943.685	55200.95757	55210.28696	61596.97261
9381.670375	6208.818	7738.152	7095.219733	7035.385882	6251.6718	7819.2774
1204197.335	1300799.143	1928354.976	1251030.216	1386812.107	1276422.133	1344064.634
60356.14017	48424.5085	117563.1069	63685.88044	63577.43806	63640.932	63575.51144
14778.71413	14023.2635	16511.59831	16896.46917	16160.48294	16040.72167	13777.73184
7695.475857	7044.881154	12299.43194	8713.884692	6399.4385	5485.512385	8129.585357
8138.018821	6929.365385	11029.81638	6946.021773	7710.221649	7623.801375	8177.176281
157373.6044	146163.7898	205833.4902	163151.1655	158380.946	171752.8906	199711.3048
1013206.485	860865.966	1823279.982	1003413.258	994465.7169	888336.0683	1223119.547
12889.68141	14996.0491	22420.62207	16527.8023	18120.28559	13006.9224	18885.47789
45925.50909	57494.7225	88573.61233	76829.78561	57936.54263	52439.45604	65594.36784
17938.17113	8834.4795	21868.91934	11833.5506	12209.46619	10907.79171	40606.96594
361092.2672	266071.4419	474100.5	290432.1675	301550.2861	364083.5848	319081.2113
17696.06358	11997.71783	31668.98985	18290.68588	17568.6993	13232.4597	21302.35011
15573.27953	9602.264239	16180.23179	13175.08617	10842.00312	9880.535833	43829.7575
634934.0506	702297.468	1073544.207	798416.5321	555404.9638	525444.9082	530889.1088
37742.57033	8336.551429	36039.62189	32980.20456	31841.04	30829.71423	51217.47849
82073.91037	47533.67789	64558.29909	57165.16794	66048.91465	50090.04939	93366.45158

658152.6097	535360.0472	963818.892	615291.5887	650388.638	704108.6798	699064.4226
2820050.113	1033800.001	1249563.184	1261841.163	1230539.695	2903972.188	2960447.112
103885.4833	79876.9268	138563.615	105388.4832	111375.48	93377.11075	139908.2674
44357.10861	22901.86975	47848.02875	28643.33559	36420.54364	40445.66771	39384.09338
414968.3922	310848.081	502673.4969	378164.2439	434547.6703	370856.7616	457365.6456
259928.6762	191770.04	425708.1491	335819.3898	339336.1275	249064.2381	407318.3615
32999.73312	32523.45375	50746.20365	37185.5946	36819.99456	36098.88525	38228.82259
92919.03625	127368.0932	199460.9844	138523.0592	87496.814	72958.71187	91734.8115
19123.05358	45884.0064	23017.776	14659.77333	36092.48019	26246.40629	49942.6378
14800.84095	13053.997	18704.36026	19649.1693	21520.12722	16195.14071	29555.95007
4079.221533	4398.81445	8528.529524	4641.730857	9388.2135	6094.5852	6336.009894
106074.7763	76104.54338	160649.6088	103540.173	138517.3068	137573.3252	99949.71865
33438.30672	26786.40638	36921.0015	31848.72819	29226.04267	29310.318	30678.55426
3917.305	3449.9115	4636.998375	3753.109938	3823.93575	3338.859937	3854.3875
10899.65176	8423.172875	11145.65982	9093.222571	12407.94482	9336.072118	12971.15482
22170.258	29547.20844	27853.17016	24852.8995	28097.64932	24107.156	26353.97311
65016.12291	59779.57324	102880.7507	64849.78879	76784.21557	61183.0645	73367.27337
94513.66487	70060.77841	112318.2329	71333.43625	67673.97071	61644.0998	75434.0895
51714.67767	42787.9958	88534.02589	52487.96589	51367.77063	50846.0701	47712.48905
18752.04667	16312.40338	13969.78688	10886.53491	9544.817786	10905.76384	13147.26065
67288.10539	59580.72125	79614.99821	49397.87047	71295.73116	65467.91159	83200.29908
36974.94851	22556.65028	51240.22775	24148.75683	21548.38407	18710.91856	33687.64725
51852.12662	33220.4809	70155.14686	42534.80832	43420.86723	34452.9744	48209.421
165650.0922	142640.877	236440.3753	139421.7626	127535.4278	127523.9625	140419.3459
36360.92904	21494.08976	53908.27848	30706.96128	25879.41563	24929.43024	41246.42044
36445.31265	19936.841	57166.44039	37552.263	23235.71689	19223.42424	48977.88006
21802.84594	17051.49911	33692.50489	24318.47432	25712.31392	20512.75312	30084.15254
11703.0925	14415.5266	15046.23509	12576.081	13237.77525	8358.96375	15605.41384
682343.8028	674974.1564	774136.5036	716626.5212	772710.5439	804499.458	814185.4995
18878.05233	21139.56375	22006.22813	21860.10956	42625.4535	41731.56922	17791.488
21982.69501	16097.86667	18938.09067	16166.6625	15613.73422	14236.87765	19960.94831
5055297.979	5000076.924	3903030.87	3392579.74	3678779.964	5348407.778	3989887.845
1439599.51	1559815.568	1918048.091	1708078.378	1671519.063	1649691.576	1853257.89
35572.00338	50723.573	58520.59223	34160.76125	39040.73333	34934.79306	24570.10385
21544.16806	21882.06602	27246.65714	18449.50322	19189.70392	26599.30617	22212.74365
175400.883	171468.4993	225315.4817	214441.9896	207495.9892	201818.9908	215569.293
420823.9817	447378.085	500407.0713	457853.1101	498169.0331	427882.1785	492481.3647
8489.707818	7245.4877	10663.7821	9587.67875	9498.796048	8683.532	9446.260667
798256.4388	766678.1101	1304936.589	875285.5512	849721.3669	838865.532	945443.2669
3049.414571	2369.1285	2959.741067	2314.440154	2407.517923	3899.980966	2558.1165
14191.04921	12760.704	20564.6305	14899.49572	15288.85795	13703.8374	14378.76753
7001.909895	5891.468471	8936.073312	6249.464941	7184.43093	5829.38249	8827.746
118354.7621	148565.6786	150858.8902	107879.8362	170766.6363	107319.5244	101745.348
278488.6965	300816.9158	227402.6269	191437.8465	188726.834	228288.5079	482872.6897
30456.5765	31280.02853	47597.58413	28082.63282	33660.58778	28139.675	31771.27047
39733.55653	30800.22971	116305.6738	54938.42188	33491.46879	92113.6635	34862.336
229708.1026	194703.2087	370274.9807	236811.5548	246405.1073	194739.1368	270900.5319
194560.1292	136783.5221	225173.4321	133676.5527	143929.4857	129903.5955	200655.6174

8594.560222	7518.773389	10978.57516	8326.023	7700.420778	7789.454041	8461.657396
36762.84694	35790.05188	65930.98267	40791.08559	33760.49739	8239.1769	35460.009
10792.28128	9192.37186	8404.688873	5801.419941	3429.551111	6606.580221	9094.543457
26115.48742	15443.3385	31640.11619	21815.12966	25385.24165	14873.99406	41677.16906
112899.5961	80416.63247	94942.3875	92234.88572	103864.7697	86571.59683	159179.7738
165383.712	159713.7262	221238.4293	178003.2276	164682.7468	145880.0432	184379.6965
115171.8765	96974.29511	181102.0691	115480.2393	123947.3696	97961.6055	132407.836
215155.5055	167439.2807	250505.5845	175488.2039	182495.0992	199845.1579	174631.3806
944491.7443	780974.6945	1085699.401	733973.6711	857114.7116	880505.907	836501.56
199049.8746	189207.0107	297777.4016	258412.4176	233298.4444	249223.3747	306835.4709
687066.6412	552638.966	627873.5919	616602.7236	696089.1347	573417.2383	670038.2235
66213.59457	46347.65982	97752.58611	62095.582	58177.20475	48802.94988	74799.09224
7574.988403	6664.404434	6727.695447	6731.375304	6648.601489	5815.463359	7494.728048
367642.7888	346942.3007	439782.3729	383711.6778	391960.8705	368143.3856	456999.6189
1324360.387	1025200.707	1288913.882	1268423.438	1288406.435	1096137.074	1422195.298
561871.0562	534240.6388	755151.1135	529027.4407	593595.1086	548209.6464	584138.4663
890551.2465	569281.0965	1381562.373	701355.62	706445.4204	636213.0231	812213.8226
123401.142	118326.3356	171162.8762	116433.5377	133011.9544	122943.9346	128210.0628
9721.609071	8273.094	12314.54269	10881.4595	11107.20707	9259.272154	12904.8205
3089.868168	2353.913559	2567.43	3200.016811	1810.068571	1963.132194	1934.1965
1846387.342	1548208.442	2910797.203	1968779.131	2014386.184	1962285.643	2347638.018
11468.90665	12022.76921	40938.81632	12252.954	13338.83172	15009.21706	16889.33705
161000.6268	112617.5516	265315.5415	161391.5438	135185.4076	127496.1796	191793.1834
50521.53179	88452.80946	151045.0291	89576.12505	80306.23905	65517.309	95313.04789
36963.80554	33587.83154	64463.70071	38742.2185	42970.17143	35483.63077	44868.13036
6327.896087	5814.882019	9070.666559	4961.481059	5434.26261	5095.561533	5119.84163
1533.151529	1275.340235	1492.677189	1400.218125	1099.753412	1091.666333	1572.312
165609.1776	136573.1967	206085.5157	124367.6288	182197.5172	145416.6308	212569.4957
20808.3622	6727.648714	7313.742818	15851.75592	7352.5796	22777.19883	4944.2844
103802.6831	85360.05124	199438.7898	118970.1698	100666.0567	89262.21053	114272.6179
45494.55606	66460.39321	42456.79943	28001.32563	25778.06356	61973.5112	47283.93809
131935.314	130698.3653	245603.115	143992.135	155496.152	163792.1205	116942.7803
3221.684812	2995.344	3305.840438	3456.270812	3327.195375	3150.504	3642.363375
389014.2185	337238.3912	418801.8469	327177.1664	366100.5013	327116.3669	429123.0797
7701.261	6842.768588	5233.2245	8390.351444	8505.893667	3749.811429	5578.654667
27899.24522	22620.51	40068.64	23760.5445	25236.66505	29304.05033	24942.47294
15911.84127	19987.62857	23540.86936	22729.87779	18234.20209	18852.75151	17194.92759
22283.30867	22991.29768	41974.28905	24777.13333	22183.32526	24975.72663	18053.36844
8922.8054	10085.53	15102.58763	13821.67168	11743.12919	9933.14034	12779.205
473388.2671	386332.5682	370984.3165	281546.2244	348612.3986	373022.9644	312982.7818
3953448.773	4298589.532	4381572.448	4279389.934	4249526.61	4371540.698	4373269.274
106142.883	109094.4395	132140.5587	88770.3895	104731.648	94189.5985	89669.23976
13941.84913	11252.8843	18516.45709	10817.87127	12083.44386	8999.118755	10767.08067
6855.133625	7743.069429	8466.656176	7810.832	9120.157889	10237.97265	10997.05729
3741035.52	3819493.15	4385942.793	4007130.048	4201445.728	3791318.559	4060106.711
523732.2004	571132.9967	724299.6566	633215.4396	596311.7641	565201.2824	598332.6043
79018.00208	20.15773426	149703.4938	62206.9804	18344.7952	2676.198203	107727.1776
642357.7654	728993.3886	856417.8996	560354.4757	685294.871	676984.3002	559627.5847

4083.4606	5316.011034	5393.873706	5416.203778	6500.69025	4239.0032	6260.884929
7413454.702	7892960.864	10096224.55	7885601.919	8451110.172	7967149.283	8092753.265
721056.9743	260225.8213	348127.1407	317577.2483	302278.6833	1154397.136	281242.123
1454401.424	1515206.305	1221142.49	1301452.305	1576906.666	1580601.787	1193495.785
7630.099228	6875.308037	4247.217333	5049.938154	6120.406308	7632.300938	6110.968719
12491.52012	11576.50474	10566.2895	17434.76278	14209.49625	14540.53462	14409.3379
7563.603809	3775.0408	10697.74284	5194.434438	4404.2656	4473.070176	4849.237143
6862.111835	6041.684377	11490.67237	8548.412222	14177.48311	5663.068	8413
589983.9412	637918.6479	507303.6565	540193.7041	653905.0737	651551.1308	493885.4448
859977.1931	921936.3584	1118258.753	904792.4692	964833.8121	902495.89	918098.3261
585654.9321	592439.914	977106.4317	649785.3928	678621.935	606065.4365	743554.7478
13561.41825	8929.291	16460.36025	12739.05667	11645.20968	8876.999059	12093.35759
8197.916972	9061.824491	19031.30936	12509.01882	10592.45819	10563.63474	11620.48532
6994.896879	4609.027271	10930.17852	8576.410062	10894.07582	5779.37591	7610.883362
49404.77495	44953.06688	42329.75068	45407.00964	65119.60107	42401.69928	52899.85451
7706.011855	5624.206561	11793.87806	10370.96997	11244.45946	7602.194338	10212.66369
113764.4683	124697.122	154554.3712	112923.2799	134097.3406	112719.6438	125497.6068
247664.7244	227466.558	606570.3115	450956.5771	118527.6483	429804.9447	136537.6228
3326.86575	2376.409588	3013.65925	2820.099882	2914.844625	2262.239	3022.983
11589.9763	9848.036665	17207.38218	12607.10065	8758.843571	15513.02731	14798.27285
8958.170105	8093.702095	13702.41478	9634.404939	24155.7241	7228.472842	21174.2559
408441.4627	273554.3396	416013.8636	349125.3365	382560.651	304850.0663	495438.6266
124256.0072	98297.08369	116073.7044	108394.5979	137351.8008	102467.9589	120611.6666
17418.245	13836.02786	19147.09162	15290.08453	14413.37777	14353.09796	15222.82223
223086.9079	254835.0449	149269.6307	135198.8227	116504.3188	236587.5829	185940.8039
53736.34479	50257.31068	84063.70436	63177.1236	59404.4768	54286.85756	69195.05453
4411.796438	3460.175529	4074.885	4114.71225	3474.9055	3527.126	3932.742941
7785.921361	4445.489364	9964.498077	7376.0538	7138.405167	16383.914	5829.7
18107.15051	14144.34736	29447.55138	44024.21067	19397.36517	18181.04488	22987.58525
22779.33047	12262.41767	21123.35028	12922.40973	13121.66481	11277.89765	13450.26746
4108.258016	1648.989154	3051.868891	2236.96284	3437.67804	2915.6416	3059.030233
211896.2275	173903.2378	186334.2405	189812.133	210028.7525	166353.4781	201640.7109
487336.9989	450074.2565	469311.4082	457708.461	574464.1578	493880.4098	497244.7506
38411.01116	15095.2686	43500.54395	48053.78382	25357.58291	19301.91809	29723.14643
56989.6749	46025.16995	89212.68585	57723.24832	58114.62404	49323.70971	63810.63333
49314.04256	43680.996	119579.8891	56165.73583	56052.51717	66839.40809	51573.658
1641549.348	1129396.512	1657692.559	1270099.419	1783397.659	1191629.81	1733374.839
18007.077	17500.59959	37603.70179	19807.76886	26803.08259	30820.5375	21408.1512
186995.2218	163785.6415	246102.2524	163777.5323	201665.7711	188468.0539	179113.2253
15385.1098	17608.4118	57930.3463	40759.206	51177.45357	26575.05654	27289.40657
24055.71644	20390.002	79575.15908	26740.78324	29091.35115	21137.259	43627.19161
13877.228	11051.265	15654.97513	15790.935	12404.4275	9665.679667	17933.10485
20346.05183	11655.55218	23658.0985	10772.7708	15503.61153	9061.523077	11136.24177
10211318.36	10842827.41	11205992.47	11175722.16	12438655.37	10854399.23	10423759.63
43176.98	35392.86392	57681.45801	61229.72	57847.14907	46078.21651	56204.07043
4242313.935	4501525.157	4737329.53	4763381.122	5139761.392	4479838.926	4321357.721
45517.77028	30361.47859	55630.78361	52695.86259	47397.74827	33300.5904	65519.8962
11832877.17	12490950.65	13879308	12249112.64	12923251.42	11216016.18	12635577.63

2784.278487	1751.563688	6222.416611	5158.303345	721.44875	884.43	4491.701028
4523.7276	1556.1	3163.2225	1737.245167	2107.803444	961.553875	2691.985111
22588.07737	12934.09669	9887.082818	30667.45709	33138.18331	13745.83906	13288.91473
101066.0312	90587.944	248330.1558	166533.8216	162635.043	135013.085	161854.3108
400283.5283	402673.1919	678981.8923	443475.4324	496936.6664	440714.2279	487807.181
57192.606	62483.92412	54392.94	57827.32129	67760.404	63095.51211	51508.70688
19302.0642	14317.56653	32548.64171	31360.05982	29042.66777	23011.52777	24114.56657
1198802.763	1154031.964	1488359.787	898524.1754	1260579.06	1163180.437	1361132.471
103852.3102	104059.7461	184092.3227	107485.0104	125571.5399	119269.881	114532.5227
11752.65708	10728.198	10966.00048	9762.680721	8395.438488	10705.83141	11246.80475
290528.296	242300.5825	362291.3781	235365.6484	252697.1032	237585.2999	352405.3
416098.1165	417305.7072	565138.4433	414437.8528	486228.8311	480533.5562	395605.224
96795.38853	93264.96033	137130.0769	85195.15034	99365.2056	104516.1134	87705.615
13462.06096	14677.2486	10591.0037	10573.13039	9473.662	12700.30008	13191.41183
1769.237294	1692.161111	1800.618529	1684.212353	1628.718	1480.808842	1532.080588
1994.431875	1936.148889	2369.523588	2286.847556	2519.404333	2020.721222	2984.811789
209000.763	200352.2963	277748.3722	202131.3506	237988.1111	232586.2367	196886.8592
13693.30285	11826.69044	20592.17975	15756.99067	17558.165	10749.71388	14974.15857
49575.636	44511.69	93868.48252	36723.15475	41585.826	37990.882	46310.94292
135763.7589	123906.3143	173292.5125	126747.1848	136739.6034	147346.6985	123220.6842
2507.071667	2776.490889	6618.187875	3440.7195	5465.209043	3543.290211	2402.297167
37191.81189	35307.73453	35267.41238	31717.50353	38794.11813	24201.77863	13257.23286
29314.13867	26780.47246	23629.22668	23114.09081	21245.72718	18319.11789	26905.04259
609289.2309	402170.3278	404086.609	402471.0934	399962.3251	418604.2959	384540.1213
15177.68394	16096.25258	21607.0241	18967.941	23490.20641	18593.5944	17108.29485
241470.7823	227648.5568	285357.3469	339277.2931	353926.6265	279680.5514	293141.8549
64986.21176	59693.70317	66122.597	88580.33948	67867.49605	73846.2056	78768.77561
79978.0747	76368.135	92592.19117	62839.23718	68849.33677	74409.14022	67902.55693
5434.626121	3864.957468	8213.242222	6015.40269	2170.052182	4140.534539	4095.982986
5939.7705	7064.65	7343.912	7989.7072	6472.161538	6643.931867	6767.355313
13372.97443	9709.599	14174.4304	12822.31817	7551.705	11312.50704	15455.01163
611604.804	569348.6773	615803.1241	645607.4424	621493.1536	622678.6875	641599.3188
566781.8593	564111.3893	766585.5216	672170.68	722366.3142	606242.0964	623363.6788
77155.52011	73265.26933	91556.4085	64273.91078	70906.3905	85820.77147	61381.37917
17032.06329	16491.08214	19704.4702	14833.62459	16610.99137	19233.57466	14795.06058
1390.592	2265.372846	609.65	1422.042222	1408.435875	1494.689167	1395.136364
12900.25265	9694.593063	26110.5348	9021.071429	10054.1	9544.603588	10271.93031
46474.65429	35586.526	55647.49079	63664.72313	64228.21463	44512.43192	72684.03288
41203.52348	30156.09129	54035.83193	33415.76838	45558.69617	31658.58038	43978.361
368927.8867	382140.907	483486.6784	430283.5759	471611.0406	400693.995	459690.9943
406737.8664	396071.5589	483252.8194	435822.1666	468894.1063	393042.9478	413522.6595
277256.9615	294068.0132	489339.012	370895.6432	380436.1539	432865.1952	322512.6958
1438.4	2617.614167	1451.550631	1848.819857	1850.169143	1991.758308	1123.506409
11646.88422	10255.80021	21614.15174	17202.988	15740.1765	10968.51893	17476.35768
179435.0123	142780.8004	158127.1976	163758.3679	209299.5956	160294.1104	193049.7449
3848.317753	3761.659058	3208.118766	3845.54585	4374.494357	4309.3652	3248.56748
55895.6943	78604.00117	84913.70042	95648.33773	57831.95794	45662.82452	95870.23523
25212.03568	33169.63452	43102.11706	25207.09015	24270.7702	23793.96348	26571.27816

75082.16802	64236.9235	77123.54927	86781.59253	98125.67003	67856.922	103447.7552
951414.0815	725536.8278	1333187.802	1381089.806	1468723.653	796459.5436	1204614.873
50875.84376	54292.8945	54010.7204	59229.21018	55808.40483	63604.37694	45962.64935
135188.307	104313.264	137533.7417	139453.1337	74711.26615	17956.44	163211.2566
7349892.744	6783019.019	8726531.93	8036183.064	8505940.841	7399412.889	8419656.219
1515834.468	1384965.976	1360364.762	1514142.59	1565899.019	1369811.134	1501577.561
7800.47213	23488.74809	11861.41886	18557.99667	23533.01268	40542.88618	7335.0615
26801.69628	20105.9942	33499.13538	27450.52613	21987.08946	23453.23725	24176.60386
14832.943	16507.51017	13585.93185	12705.60653	11543.94942	10011.423	12196.43979
23007.70894	20424.13535	47573.13453	23906.82133	26149.53293	17290.85306	25737.639
14397.19848	10770.21086	19885.53173	8410.1	12856.52045	7619.660769	11085.70683
53191.62353	38929.15917	66966.07832	57720.88	72624.92589	58096.1836	83136.91442
56706.73613	60452.45996	75803.091	57192.00828	52207.55889	50711.731	56942.11688
4685.168231	4561.275333	5955.689006	5611.273572	6260.081373	10020.19431	5388.960046
3478620.201	3634392.875	3839706.323	3864723.635	4166373.089	3601929.208	3597554.577
21175.97263	9847.6875	14682.61656	11474.82546	11920.69883	12211.98753	11183.3301
795280.6331	736414.9123	943946.7154	1144737.358	1084032.004	937771.3456	1073734.299
10421.15524	4259.578818	10896.78927	7467.433778	11840.45149	10683.25272	5101.209
3815567.018	4249314.982	4393160.307	5026128.555	4627222.856	3979196.418	4046189.002
13352.24984	13039.466	11042.17917	13638.61865	11609.87431	12676.11893	12912.648
15231.44215	6641.037056	11535.41753	9187.752706	10977.72706	7354.393941	8909.537765
89806.31922	82507.00939	107073.0351	125375.1151	120017.5682	106915.5838	114187.3346
457949.2356	403699.1662	636702.5173	611545.0544	548193.9861	519125.8756	612678.2756
3554260.562	3476806.492	4035061.043	3651844.733	3861342.389	3281730.437	3454798.308
111911.8942	55854.83133	94684.56088	72032.85476	75425.76055	49668.99256	71364.09124
65545.5184	54275.23871	77462.90457	80965.188	86975.32324	70736.521	89868.10292
49653.09805	44269.6408	55885.30432	60028.097	56888.23524	49890.46628	56378.30755
1840366.753	129332.87	3118335.078	1469574.528	439573.6356	234808.1955	1653281.802
377803.6051	521144.0143	517502.8176	397593.0291	443880.0923	465269.6013	326707.2876
79384.3068	68087.80057	99961.253	71926.47096	85963.91833	72605.60626	71564.67287
6671.736533	6036.802273	4335.2424	6556.489313	7887.777313	6355.6896	6302.51625
897242.5269	75480.85375	1283063.228	690152.5218	236999.0588	133040.3166	800152.4774
470344.1837	422738.1052	721494.462	498356.745	540864.0751	422948.312	571515.1436
190719.9745	23484.60467	322830.27	213625.2527	70505.885	35065.15357	493521.5931
2742197.691	3792871.002	2826742.241	3039715.574	3605422.982	3433360.761	2397169.809
1265343.728	1588505.551	1299871.584	1354189.105	1500929.019	1563090.761	1104549.402
273430.6921	115382.904	223994.6561	134855.5622	143922.0957	132389.7723	154133.8856
20142.0987	9664.0635	23954.02884	17005.42713	17671.19214	9486.73732	18432.65748
116780.389	105132.0096	143947.2586	145628.8286	330748.9087	119290.8021	158390.3347
248486.9692	251755.3837	278024.0986	275176.5295	298799.6301	269202.4985	222286.812
78069.388	92560.1805	83658.0249	79225.30068	92160.5551	94521.5329	68443.4945
85842.92024	71867.85493	50871.57	55285.52769	38767.92818	53863.16267	53024.20667
9333.867151	9326.098022	9438.380722	9048.020601	7866.9061	11459.16373	12613.70456
78690.66518	63758.24761	49409.18212	45419.33929	76124.67653	72416.52041	68528.81259
7784.808158	4856.904813	10349.45106	15542.34348	13297.28947	7098.324563	8943.5164
15553.36847	14184.34429	12804.13497	12459.94606	12290.80888	12601.50615	11307.87794
160258.1243	165980.3578	158742.3997	116704.3143	109590.404	107035.2963	122381.831
9372.622176	3520.067	9814.915572	12770.84089	6759.085951	6346.941334	11605.90971

58047.92876	75483.37491	77453.80629	124881.964	78851.7074	89221.64044	78695.45465
115280.9	112661.4583	121817.2133	129537.925	140120.8439	129587.255	108890.4333
11971.40452	10468.39862	13430.15044	7944.86	8613.664409	7883.676864	11009.17826
403441.7459	310599.3742	382638.1817	349647.5857	444050.0699	359371.5738	487262.6034
3276.559929	3531.503162	5052.613364	4326.052197	3715.506592	4592.387126	4169.235279
177223.6087	174298.3337	217159.6884	202545.0808	235558.6477	196137.9015	193247.698
24173.60632	22179.2595	34200.4895	23890.038	25666.33488	26643.87041	23976.68267
42935.29412	36111.75403	46046.79316	41797.52457	40010.67	45550.68289	36854.5518
251359.2459	208441.3026	283990.3702	302004.376	310982.1347	258116.8978	279639.6387
1225960.543	1038711.952	1402454.688	1362449.172	1382645.388	1163399.325	1519884.125
122370.9159	126813.3394	187060.9449	144730.4603	157848.3029	147313.9705	125692.9522
212704.1879	204570.0565	344571.6258	284336.871	278247.1778	267009.0318	244148.9271
5712015.58	5594935.127	6531895.925	6421973.715	6730928.624	5893971.941	6359890.138
24742.44697	18108.547	32929.61287	31401.40232	30647.27335	21362.17491	27532.22381
60823.14075	67376.619	151603.1293	81631.98918	79745.61347	85281.19253	68953.52272
200183.4093	169640.7279	243997.4295	187847.8843	202278.7144	173462.7143	190745.9783
7139336.286	7337602.118	7503129.713	7495290.626	7820962.688	7170066.154	7220592.574
3298306.301	3346070.859	3926997.4	3724870.24	3972295.344	3458092.563	3634435.804
358436.5371	282277.8246	353889.6918	375372.4535	388634.4589	339801.3824	408229.143
3163418.423	3235290.873	3529891.836	3364494.595	3439275.802	3260894.506	3273625.606
411110.721	411953.6893	476415.5916	459405.2892	490791.4636	429672.2165	453955.0272
1000980.591	1000319.869	1041133.973	1053717.908	1074185.756	1030648.574	1044319.57
1434877.395	1379609.785	3659699.753	1703511.712	3658853.679	1469170.239	1616519.24
1216926.735	1142560.295	1407882.732	1433877.929	1410348.509	1212453.941	1297481.345
143078.689	161764.1982	132617.0737	102066.506	100061.3769	90744.24343	153700.8395
203279.6777	210834.3826	287051.568	223159.5935	241410.1128	215884.8578	198439.8365
642112.6249	593121.9274	762650.7039	742715.3658	770654.0533	661612.1135	697467.288
48024.04606	24654.932	60274.641	46253.42765	39952.25259	40295.43042	46456.67529
555576.7922	489183.0241	716538.0388	700863.5241	751359.5153	852728.2131	654687.1385
646980.2411	631900.5592	584168.7866	669857.9273	687935.862	707201.5403	555369.2127
101479.9485	49088.99594	103843.5591	76071.73925	76188.907	60661.3255	77134.70494
65268.76421	39796.07782	56521.48594	47949.45653	49847.41144	35315.04268	47408.44283
7390907.7	6598628.675	7707192.199	6998093.805	7519293.227	6752455.688	6452086.736
650606.4666	612882.9737	703428.0234	664155.7097	802730.1091	648780.832	665217.8532
95195.4952	50729.21831	92238.6062	74918.1028	76247.14973	54459.06713	61971.14033
78878.05573	65830.91529	77715.75625	69620.0934	70055.71513	61904.41331	61834.2684
4114.393818	5972.705538	5600.293746	3049.245	6101.154019	4966.357	2801.131429
433058.045	387823.6238	428543.2581	469429.468	516500.7883	462431.7523	411412.5124
457199.5386	218855.677	541038.696	350885.8421	355167.2759	263828.0387	296565.9056
93077.30558	70891.029	90782.04379	86831.11384	85896.52305	87357.23304	91529.04055
322029.5927	208233.9754	488907.9587	352656.6881	599343.1138	550072.3959	577936.8332
62327.9535	82226.2056	93293.88011	55452.43526	66283.81989	68390.43332	51620.15195
58518.97448	49660.48774	70126.6032	50090.41374	60435.30533	54884.56527	40432.264
152112.3486	151828.3447	348714.6474	282376.3231	282735.3254	255396.3516	262513.224
162630.0831	221861.2779	181335.311	225995.9055	170564.1092	214133.4156	208663.5508
88692.98	66360.07056	77560.07919	54686.80943	63646.42947	70211.20129	55653.10479
5631.164375	13806.43745	8813.087938	6252.893648	5233.863428	5272.1296	5289.5548
58934.53971	53902.6705	57513.08	50932.17478	54653.49133	46051.35525	55958.30057

47583.42363	53516.83956	57839.7769	55566.02788	65018.85411	50595.90963	53862.69354
17511.33533	11517.42663	38207.78994	20409.60842	22038.51853	21933.53589	32829.70028
17817.73	13755.36441	17919.08139	18663.07114	17372.31922	15897.76486	20291.22648
762898.9146	792872.2797	624347.8863	465978.9079	423861.2678	450548.5568	443570.5626
11345.00769	12700.60723	10895.96182	10888.08692	14479.9322	13265.06203	13315.83254
11885.86044	12821.05645	16512.1504	23132.74876	16420.40336	15106.84336	11251.18893
26344.87768	11015.85794	20379.64669	18311.58225	19537.8595	13151.07169	17906.86275
85865.73474	78219.91485	75762.2701	52196.99014	50670.62438	48797.7767	50538.1275
3224101.414	2751726.586	3415884.441	3340584.341	3646955.051	3134837.241	4015441.194
53861.2833	64433.786	60261.07094	63231.20745	57005.63037	60482.94254	52126.34707
21492.69406	5678.513143	37484.21588	42456.11706	11702.88306	5367.871338	23252.48613
11149.46056	9697.621719	10584.58149	7448.560979	7090.555021	8096.131714	7363.441071
38633.57476	19680.55475	41996.57841	27894.15238	29857.34841	24324.76976	32193.60512
28458.44276	28657.60223	40632.654	44542.43327	50071.1461	40341.61195	34774.05123
123630.1379	111990.9832	114990.903	116596.2432	124924.8854	115168.4673	117034.4269
32648.71606	106089.9184	34931.3991	133486.5559	121198.1861	107603.28	33283.299
1740856.643	1514740.413	1598652.988	1687238.415	1718027.444	1646934.331	1887672.877
12583.8225	18014.7236	9794.48625	12091.72248	16258.79728	13737.68759	12850.26215
43839.251	42785.20402	53256.55029	46857.7443	46761.76286	39265.63362	48773.16436
5934456.331	6600450.833	6036255.185	6341463.555	6524540.5	6473599.764	5496383.296
166532.303	141592.9457	181300.5044	183683.3872	200404.1098	174273.5912	216095.5673
14037.89071	271392.8718	16384.20176	16025.71121	19263.8784	16108.55808	14188.43089
10024.39988	11974.248	20881.82893	12293.79113	23549.51715	10197.634	11844.87479
733972.3533	794443.4177	864253.507	946874.0817	935794.6926	860891.4847	780748.0013
3294846.912	3923750.037	4375942.567	3684675.715	3878455.188	3829958.139	3258526.486
27063.7492	22238.67673	26319.09091	21168.84612	29603.15194	20367.83775	18534.57508
31805.5864	28747.46187	64837.71851	34396.64454	30939.2187	27974.95247	30731.80185
11003.54513	3995.637489	12270.699	11603.0955	4842.495059	5232.669897	6638.267647
1417521.645	1632811.504	1641428.347	1605944.564	1715779.18	2308379.456	1244288.295
436192.0406	465191.4382	541774.5757	485190.9708	564755.0396	471261.8145	459160.5594
433179.1427	469272.0306	558125.9955	538846.1727	552382.5159	464583.5529	500891.0583
34604.05529	19397.17347	40903.51712	35938.17987	29111.86429	20767.406	29977.62525
147003.7917	149449.2756	164562.9379	163215.3195	188827.3561	162321.4666	169386.7391
213092.7194	223405.6896	255231.0353	251333.4678	275922.6329	236486.4538	211129.812
185473.4331	78492.31571	158604.6833	126374.7065	122937.0358	95189.01459	149800.7317
210879.7826	99718.6899	234963.6177	190985.4711	172028.2104	109650.9902	165833.8759
35938.25681	16707.1897	33792.35318	26599.262	23844.0426	18699.14915	30396.33181
793799.188	512721.4268	768017.2986	756890.6296	716065.8527	442843.864	807263.8313
26518.632	9310.592318	11382.09429	7857.93314	7766.677929	7667.352	8048.98195
146713.4908	122858.7095	128296.9421	137953.9347	157622.52	149783.9095	116304.174
1860.256947	2175.301783	9785.53526	2617.767256	1915.971467	1886.596959	2370.322159
1137129.397	598248.1466	1571683.285	899934.729	876714.487	672199.861	955979.26
156542.3919	134080.3652	138954.828	145777.584	147791.669	130696.376	152302.1587
135948.4646	131074.4441	167308.4406	143408.8303	155959.1734	149563.1434	122207.9317
27645.26613	13432.5878	32602.37175	23571.58781	21397.046	15322.89963	22011.52643
30419.79005	31267.71717	48439.37062	29573.79228	30375.97145	25214.47488	27837.49911
38159.4647	23973.06522	52508.5137	39616.304	35765.27844	29710.73324	43811.98523
153261.0058	146577.5961	234219.8525	148230.0832	187294.9147	173818.6721	122342.6781

33508.31314	29040.28303	46031.48951	28332.12369	27212.864	34430.143	22954.29493
73381.72203	71360.08548	136778.0414	77696.7136	82191.28773	76843.34887	67408.85387
40146.799	47379.57939	45564.55311	53852.32679	52197.00585	85143.44107	82686.78225
21406.60976	8422.9618	17026.7322	14488.7569	16434.3021	15165.74499	15692.6849
3115.565714	3219.812558	6882.267	3632.964844	5539.835829	3423.417706	7482.793175
63504.95219	28374.66319	57870.96187	44820.80224	43063.53844	35857.75447	63907.30445
715989.4587	666890.0138	598533.826	671183.5531	777664.6976	733620.533	728393.1167
14680.6415	12188.2716	12569.13826	9033.73888	8165.040478	7320.697857	8882.930609
783327.2659	847634.9727	831497.7883	842274.6967	851615.5292	839984.7736	805858.5927
9749.114	7730.514091	9093.48	4996.568961	5934.042303	5957.716875	6172.322667
395760	464376.0885	424759.5844	423098.1106	445644.9626	416643.8112	412727.5034
576044.9151	205784.7143	244400.4876	239533.6532	231098.8171	213054.3791	219819.435
5613.664263	5498.303684	14655.63324	10685.04777	11275.6682	6122.980833	7423.797571
23201.63286	24022.68166	27774.82059	23932.16407	22734.8897	23442.59745	22293.47389
9353.91375	9600.448059	20877.85935	23421.177	19186.35883	10982.55324	26493.46596
191445.354	190157.5895	221082.1942	218960.2663	235484.4478	223167.9815	186128.3356
23359.17027	24051.03774	21360.95721	23346.6326	23127.33352	23879.62975	28638.90491
24690.77291	27625.1712	49345.8545	34020.04281	30139.38996	30771.182	23676.42769
16551.402	16953.20957	34464.69647	35469.83453	31906.2375	30886.26565	33197.92759
4908.231	6999.952539	13855.42236	7559.089355	5818.275852	5456.841	9049.057263
10489.2453	9207.458507	14719.77391	10860.54513	9829.746852	9369.772907	9238.793991
146511.0942	62065.52125	115008.4066	95142.18331	94633.05206	72157.22135	101219.9348
35129.84067	17123.03586	38090.20286	31002.39978	28660.61096	21091.87067	26868.68467
638595.4131	268761.5408	605293.7012	476423.3057	425094.4999	338141.0359	525363.0282
88295.26659	39060.06975	91517.27637	78802.22494	66814.092	52059.804	72875.37088
343066.4585	175074.0051	446045.9409	289028.5115	290875.807	203260.4136	299150.7326
95037.30456	94694.679	160919.395	96994.60339	98336.83029	99284.04822	80728.87011
14937.76075	8232.123625	12677.1176	12185.2742	12014.39475	8533.36438	12508.99525
8890.3573	7831.694111	18462.88788	14649.18446	17694.15343	8124.500322	12015.444
140218.6921	298225.3428	523873.1868	293568.6654	167816.5278	316337.1634	226213.4249
5112.134072	4369.889361	6334.136375	4435.054349	5665.513031	5442.36	4597.490711
3795.592719	3209.900264	6143.422	3581.396864	3608.303909	3727.213177	3497.8245
7198.890524	6382.849917	15857.98933	11209.17244	13136.51916	6817.340211	10458.81415
233275.8144	303399.9975	743690.5365	313161.4851	309217.545	291071.4516	269175.9726
43587.21159	51293.50033	111870.603	51883.04158	54891.77419	47670.82078	43425.58176
23852.125	15495.0163	30569.84354	17896.626	12580.93295	12951.34335	15928.12717
35874.20263	15518.625	34865.1875	24313.48419	26576.97825	27159.48893	27322.91756
936514.5244	1188490.843	907598.5676	921315.5819	1007811.544	1075475.608	846472.9986
39183.0855	23817.98758	39009.05268	31790.39988	34735.96662	29973.62571	31740.68867
29967.60918	18249.04586	30637.24987	32027.53337	52020.31897	25066.80106	33562.51615
26377.37006	27782.37597	50661.53	29157.34909	25437.381	27853.72588	22956.68414
11274.46067	9819.554786	18335.52557	14095.43056	19487.691	9216.1034	12749.2494
10047.02764	9432.171204	8889.271273	4685.134	9283.437581	16100.26146	10161.3898
14935.167	17932.34743	40486.761	16845.46986	17246.11226	20321.38062	17365.38208
12491.69263	4596.9832	13418.29	14729.17926	11364.63944	6016.596238	10980.108
25780.51659	12444.90565	26520.65328	22863.51159	19212.98241	16314.04125	21891.49147
14340.05876	6097.83075	28183.41254	13654.911	13410.21871	9718.529059	11446.34894
2114.33292	1926.875434	2572.347895	2228.903288	1610.154699	1579.725342	2299.945804

46577.24292	46723.68485	123840.6483	50409.75025	44243.49975	51498.08008	34482.22536
28476.112	21344.21219	58007.796	30278.523	31366.26043	29470.76319	28498.90944
4710.564784	3414.4055	7097.571124	4470.229737	4080.627869	4647.350842	4402.393211
2449.023125	1244.758827	8811.390915	2045.880046	2873.934002	3423.622563	2646.617188
23744.259	11795.634	37539.73438	17117.94663	20042.73131	27333.42469	17697.11825
8275.93171	2773.118863	7636.818146	5719.700432	3908.856824	3650.978471	3994.656738

CAP-606	QC1	QC2	QC3	QC4	QC5	QC6
5953.482167	5994.986667	5522.987182	5290.366909	5316.715	5247.142727	5541.905667
19331.65147	29098.51334	25878.7909	21505.06593	21374.03036	23789.68265	28706.33214
1001829.646	1186434.686	1162239.579	1220589.945	1143212.894	1177212.757	1164866.947
10247941.88	12331973.79	11181380.32	11513022.15	11107273.78	11424444.87	11257332.21
5083.851167	7931.948407	7494.860706	8756.137105	7251.43295	7643.941813	7146.130412
205434.1562	284189.0256	278277.4914	287921.4722	286007.6475	280725.3493	274131.6233
10747876.81	10892252.11	11035485.27	11183519.87	10636301.58	10275674.81	10748267.21
13786.1412	6735.662063	7838.247682	6061.907538	8101.265565	6033.039	7282.8085
31594.8	57986.35143	53046.91221	53610.35308	55323.55929	51655.867	49891.456
31682.295	205099.3035	207304.4177	208049.5761	207181.4741	199844.5826	206183.1516
83837.299	139415.8514	139045.2933	141740.8955	148499.4672	151900.5526	140536.3187
58108.88723	40685.23879	39994.68514	39443.40571	39472.266	39556.42223	40256.6775
135325.5917	196953.3011	191010.473	197722.9654	189529.6893	194419.9557	192129.1126
392667.3867	648219.49	624198.5641	646834.7914	625467.226	663005.1787	660613.752
36711.50479	53566.36257	50734.73507	54202.70064	49000.38769	52916.62843	53427.00638
8555.2105	8587.658091	12730.09504	7234.836	9613.811533	7531.128	12711.57873
86132.37773	134781.288	141331.0328	122020.4	120929.448	142863.6686	116524.9164
364620.1346	613086.7045	582304.8384	584476.3716	567881.208	609662.4518	586211.9453
49576.53846	76271.54563	87588.702	79214.27264	90964.02935	66241.456	70638.65292
27355.07364	35657.684	31082.4192	40285.16026	34552.88782	39888.6497	35960.28545
336600.2087	353953.9809	346282.2155	360230.7909	357815.9728	339651.7911	352988.9646
14627.57806	24060.74792	24205.99176	20706.59213	24110.07662	24322.76294	21416.85807
267995.8822	506581.3065	459552.9924	474104.4858	476725.1733	463277.5052	472878.6854
286088.7485	431074.7419	421393.7769	417837.5771	406570.0307	399112.4712	419574.9893
79605.57465	1535.778455	1691.4868	1469.055	1664.52	2016.477273	1177.868571
8893.646667	46036.24693	41558.37247	44843.01067	39880.42744	43109.55582	42659.25406
22990.76	51744.59879	54619.66627	44091.8574	48306.89815	48339.74764	51805.08514
16452.976	32548.02084	35149.965	33942.3755	36989.09333	31966.224	32945.9475
83863.48933	159176.7936	159389.2615	157053.1222	156724.0271	158817.5351	154889.7366
139067.9179	225786.2266	220513.6807	223004.3799	214918.7531	218466.304	221799.136
308301.9248	765737.5705	753020.9165	769051.0069	738056.5911	753193.7493	755742.797
6282.456245	7726.369769	8104.183313	7854.194538	9103.233444	9587.750636	7699.838429
9536.063105	19209.90094	18808.37019	19491.78289	19104.79329	21798.6703	20347.6075
49904.071	40416.33233	37660.31331	47087.32362	40465.18257	35905.76	43498.4
184435.3515	385151.7502	375300.303	380438.4105	369857.5589	374985.2334	376723.217
2480.095247	5207.960313	5300.885556	6080.080826	6379.63687	5361.540529	5383.885863
456229.314	986395.2	999315.7745	1022011.135	1120444.244	1010861.43	1137640.376
4418.313	9180.9725	9420.3228	9545.234786	9106.5618	9839.957143	8963.232301
33050.12904	11664.87583	10804.18443	10948.99143	11013.62087	13052.566	20108.38352
9983.682878	12608.85763	11704.66894	11946.42219	11488.73843	12167.67256	11536.4788
20963.58166	31417.59693	34775.89376	30477.0424	46070.70404	32971.06482	29318.553
9072.601533	19134.87469	17620.81333	18148.75388	17908.09669	17275.81227	17697.81131
144942.3725	421796.8944	443308.8468	414157.5158	402618.6094	397273.1941	398600.3975
99882.35988	232751.767	231238.6234	241104.9686	232640.6773	217359.799	237717.9376
104264.0043	235980.7291	232582.8015	233828.3559	231544.6982	232867.4478	236517.6494
12028.22545	13646.2	10988.0652	15504.34133	10477.38	14650.86023	13975.06508

11772.28213	11444.07105	11032.7312	12189.82454	12869.64606	13244.30744	13487.61494
6704.337	11010.94743	12323.58038	11326.902	12050.53693	11349.51954	12197.0464
5055.242422	3606.142	8075.62025	10040.886	10650.80274	9455.195722	10276.99194
17649.22669	24919.20395	21067.15886	25547.9778	34125.54818	22188.17836	23288.8275
84440.2428	191435.176	181087.0025	193821.268	177261.2127	175705.2267	185785.7707
2399.1704	6795.774442	5741.382933	5144.863615	5491.293357	6346.839706	7429.857235
29075.61643	32871.20265	27235.33643	28924.48508	47477.04809	29469.024	31630.11877
42371.42657	58957.81692	60577.83642	73677.28708	55751.25842	59472.24154	57704.59077
3072.872773	4386.549231	4902.507824	4938.3815	5674.596286	4633.6444	4629.723
8093.202	17654.76786	17645.22527	16604.40571	19452.2736	19162.27868	15640.24007
12805.31877	10926.98531	3797.849455	3060.777778	3146.654333	12544.97489	4051.83625
1598.31625	2205.011769	1671.159875	1959.225	2334.048182	2804.482167	2080.3006
4418.720131	10561.04478	10114.991	11444.01038	11261.34593	9378.634669	8380.260938
18813.52183	27509.42338	28669.056	26646.70914	26444.34169	29478.18006	30304.3833
5820.9624	6958.278722	6967.814733	7744.816798	8269.368435	6278.95	7127.594583
735705.0888	1125392.625	1137573.909	1134635.82	1136519.263	1048767.28	1111280.538
746386.1068	1050373.182	1102253.323	1070357.661	1019309.4	1029483.334	1126045.728
173155.8336	452360.9638	434655.3864	440763.7341	429355.8501	419414.5471	432664.0529
17216.30764	48173.8112	43695.1996	45955.93257	42953.92307	44932.98469	47991.52036
12930.40313	10165.37194	9939.306667	12275.36505	16372.3507	11446.6428	10192.03054
2453.210769	7910.976291	7537.431789	5548.492714	5912.255143	6816.082706	7227.588197
1395.764694	1863.164687	2089.229538	1630.392154	1565.248864	1831.927133	1717.939612
18461.716	47920.18176	77821.49419	28099.1633	28113.95909	28163.4	50880.0264
22746.84525	28662.40369	35384.31438	27262.32315	25512.45667	26925.79715	29868.33865
144663.6118	224203.4162	233224.02	255548.841	299322.0515	235934.8979	241230.245
98341.51833	138297.3311	136071.76	138600.9537	122417.5382	116701.4628	133655.9158
152181.7141	376573.9424	286692.8492	350232.3381	348579.8385	276513.0795	364762.7057
18692.16417	24892.17522	26505.46267	21439.68603	24380.90862	21717.83962	24960.77792
116863.8638	182013.9203	171303.2952	185290.623	180869.5054	182105.6081	184479.4324
288170.036	300876.6864	539849.5371	309589.9699	298978.75	305900.8619	476249.6498
220898.9143	341487.6123	327401.2971	436168.4705	333081.6923	421304.501	433992.5568
265935.461	215384.2757	218609.937	230527.39	211976.7542	233977.2173	220470.0409
5127.44625	7845.771375	9807.748861	10453.54286	9784.985933	7134.363	7165.59375
33858.3315	49016.59717	43643.07332	41783.06295	44759.77426	42700.326	41873.64961
854484.1773	2374274.538	1306617.22	1398060.295	2360457.104	1333503.82	1318576.992
16264.74525	16398.63623	15863.4225	16471.05107	15693.94338	15787.08623	16792.41244
2668.344778	8407.058688	9557.767227	16633.18098	9940.899647	9537.180947	8534.083
6419701.652	10465454.46	10302081.27	10373026.4	10290718.57	10255191.76	10258602.98
3289971.18	4601608.766	4850956.88	4801087.39	4445742.712	4438549.341	4785958.81
53556.3862	84826.63895	106515.7496	99772.47082	94871.57464	85564.50503	69585.06893
57009.49318	143604.085	75259.50775	121270.5526	76254.305	76974.48	77931.94583
132753.8265	222135.9169	261940.2747	228706.2793	227612.6077	224915.394	226159.0719
60923.991	98596.37575	139637.1331	91265.3696	67498.28154	135076.1483	72151.09417
321405.4941	340107.7814	341941.1638	351818.8607	350360.6786	329649.2331	339349.0081
263755.1836	379214.2315	372227.6239	381054.6929	358258.622	354573.2007	372536.4344
27388.85422	43516.0184	66587.384	36635.56969	54067.2	38590.76506	41579.22418
36266.92131	55999.00223	47759.46	50626.0139	47982.39716	49977.06	53962.59346
146014.0478	167940.8465	178166.2372	173129.8247	169350.552	177511.89	164707.4867

267883.1143	398427.1628	436190.183	430457.9144	409298.1352	425765.2277	421735.8807
19932.99963	33704.30093	29550.26308	38716.383	35607.78943	36720.081	37262.51314
81646.53242	86380.61533	59190.581	27767.7818	56898.87	87870.63738	87468.704
130797.5129	339027.9027	222436.9805	197114.7024	206188.31	201395.877	206528.0554
69147.25714	81370.395	99844.2384	90817.366	85891.21582	78357.72793	73143.15364
254398.432	559941.1295	275034.0393	288829.2982	270203.3455	288451.4564	267291.8975
1045703.073	719059.8618	719342.0071	738446.97	747329.679	685609.7622	731443.5386
60311.89559	93064.0145	92963.39077	92217.98957	89226.9615	92955.15339	91626.60479
9904.027188	25987.72606	26183.615	26182.47638	26678.51333	25151.9254	24802.60253
554230.1692	700166.0355	665846.5915	694558.3708	709345.5888	674277.2763	726251.5509
2449323.258	2740122.924	2749908.437	2709046.585	2724619.772	3727236.188	2655986.985
8612.778625	7787.054	11802.28185	11482.19868	11326.21793	2365.584	12037.07209
3932340.252	6781625.596	7018410.227	6920959.08	6919967.738	6732406.108	6859269.008
69600.78655	110391.0588	117171.0954	114200.4693	109619.3854	113301.6168	113271.1
3928660.291	6816328.486	6926765.331	7117719.01	6753347.446	6856089.822	6956300.176
96607.43185	124332.2747	131535.4816	130282.9707	130192.3432	122897.1709	131774.5263
5166.365914	10796.25289	10364.87355	11970.83153	15807.73082	10423.01098	11574.79356
2266021.96	3866239.895	3810373.949	3840282.199	3839184.962	3839295.694	3926011.774
12668.187	11081.4485	11603.67279	10520.62194	10863.79273	11313.09	11298.47061
226502.4355	350322.128	360663.778	359404.6488	343016.6964	359811.0571	357927.3131
821625.6059	1513607.582	1587694.22	1540763.538	1534982.596	1516087.966	1540928.315
181274.73	363083.1449	364219.1038	358955.7362	353778.0668	352218.5537	365801.0761
239413.7798	200644.437	199316.1586	199545.1618	111220.4609	130126.4846	185223.5011
32989.11938	44113.36947	41302.61179	42213.94531	43594.98919	41179.4261	43767.3197
181713.8901	239050.2627	230501.3798	227186.4257	320586.6905	229737.1067	233627.1915
6552.127347	13686.86342	12460.23774	10926.38182	12169.87816	13088.5372	13189.84566
3091240.874	6548768.445	6882714.616	6560531.384	6587236.457	6447280.231	6383593.565
1945.559778	2774.44125	2677.200097	2937.914429	2011.06367	4359.093786	3758.637
81736.03124	51574.548	47396.89453	59974.8105	32325.09073	64052.642	56741.22
313582.8322	503965.384	513033.5135	510110.398	510178.0478	494436.3148	495260.6981
99339.96262	138719.6635	143190.3181	148022.9117	136995.634	140097.1092	143588.4749
204178.244	123276.9955	109317.8616	109069.956	106066.05	220425.5036	133022.2909
6480.891	9558.0815	10260.76025	10198.13117	11436.59888	9280.553333	10861.16375
271426.8501	302425.2	351706.7857	290378.88	295813.8893	270915.9965	287957.411
31409.83319	81963.42573	87521.81896	55964.23167	53836.75935	49832.415	51349.21763
11610.77313	4352.357333	20780.41042	4260.776308	4520.747167	4142.125429	3974.133333
2523357.103	2887100.95	2798533.915	2874143.455	2807983.595	2683827.681	2887573.7
6474.027091	7725.7115	8960.468462	7532.180727	8662.438077	8519.657	9477.066667
5756872.628	8761732.342	8919699.374	8930458.91	8515154.541	8594335.276	10857424.16
1773636.73	2171025.99	2222156.944	2129605.587	2184394.528	2102353.33	2165107.895
1458662.185	2413873.576	2349128.479	2397487.045	2360626.43	2391151.848	2427374.219
51434.96012	65370.29142	56999.31041	56528.86022	59335.731	64662.757	56537.98398
93191.60833	55975.41605	52850.42479	64376.85755	46213.46847	62007.33426	62740.71887
11342.572	26036.95646	27986.5695	25332.06586	18459.88083	28117.99838	24589.3795
910880.3446	1466156.56	1552799.391	1558211.781	1451013.392	1405993.888	1535043.406
54252.51338	93287.99654	96824.63769	96974.36462	94446.85154	93054.01769	129775.6527
30691.15857	34101.86188	30856.86894	39780.48373	32337.844	37866.10269	38037.01365
59129.25763	49781.42185	48666.08557	50205.77336	49063.91215	48452.39721	51619.19323

118181.525	162914.8994	149535.516	148374.5203	145155.8229	156358.7529	148438.0075
31643.4334	25106.0545	17151.95947	45739.48132	42391.78656	15112.15386	14673.22909
1492.927002	3869.457194	5846.0541	4394.205429	4510.23925	5215.4	4220.48
48286.07008	62408.88046	65937.73131	64750.22308	63774.05031	64334.9	63934.39615
264555.2904	434241.0923	457749.2658	448250.1869	437507.7059	445685.4576	448490.515
19058.96138	31449.28969	33121.00571	31238.54194	32365.43958	30277.05857	27489.39006
39489.53715	61807.056	65582.32275	62864.90135	66414.81141	56003.73289	56158.32137
191782.6671	325302.7869	329882.067	323899.4648	322046.9303	326800.9807	341758.0288
347407.0321	426043.3331	412921.8172	453219.0368	439146.8801	410093.6221	435980.3113
95547.31863	95284.20181	102426.177	99908.88914	97124.06005	95412.11395	109278.2518
137780.3037	166703.1259	173893.8521	175186.3554	158825.4789	191525.0872	165247.9306
12613.86686	8847.094118	13064.63724	15702.496	10655.33157	13070.57245	12672.97018
12473.71516	28309.5	29038.91362	31060.16957	30532.021	29614.64377	31400.95779
86860.77217	111275.7958	122244.6435	173747.3457	93846.88321	126309.8554	114045.7979
167988.199	171352.8501	177323.7014	173504.3321	169816.653	165858.389	187994.9822
10839.92964	11035.22973	10471.7525	10140.56492	9072.484083	15235.14431	12133.4468
113803.7695	181798.9035	180661.6747	177105.7385	175819.2924	172165.7769	177753.9877
246176.3571	203448.6212	271000.4592	228156.1042	208316.1961	221884.9173	215604.8466
17687.61	18221.56421	18379.899	18057.30923	16487.74525	18182.40685	21809.80253
338107.8688	472802.1188	458108.0192	440434.5737	407437.9325	426753.8998	440290.2351
16038.39833	19603.76858	17950.24	19242.626	34381.24483	23159.78138	23806.596
41629.4795	61400.24	65092.62964	62513.98373	66030.99844	59667.52	60167.61225
17385.82566	24840.80029	28259.51744	22472.97412	25527.1621	25812.36688	24524.16679
13474.68758	15807.35931	21784.59715	17002.80083	22658.861	22377.79385	22401.08308
47306.27785	65342.82898	63436.7815	51793.07722	54580.66296	59513.55538	66791.835
438407.5378	505420.377	535172.4427	509163.4889	500332.8046	506122.8982	518832.1382
48505.71014	62231.42758	61684.90426	8852.776222	53747.45572	10106.19369	6539.966375
23985.76494	33048.55872	34109.4667	33615.70722	33901.47039	32235.91158	33155.53822
2852596.834	3677424.773	3666481.961	3668401.283	3502634.107	3566590.566	3662674.063
6182.033157	6519.407571	7250.694158	6896.374625	6346.208333	6643.070769	8484.038086
8529.297	8271.753512	7699.397538	9239.45	7834.226466	7912.073266	7078.341566
4475245.524	4925280.507	5099413.631	6659252.375	4865106.225	4841554.232	4955079.455
954412.5319	1403186.86	1386559.8	1465106.05	1371981.195	1331155.847	1361724.34
5847960.41	8899463.854	8901997.776	8754703.245	8609954.404	10236161.47	8851777.035
633848.9077	878286.3644	682742.9439	1270117.063	903516.0805	548480.5873	636081.4355
75146.094	96661.01786	110380.8736	91534.388	114329.1419	111047.8331	115663.2517
373654.0048	420709.1164	405835.8807	417287.2093	426648.6974	466825.6466	391342.6459
660371.8457	1044452.64	1049196.519	1076929.62	1026244.854	1036806.637	1020686.345
3842.243375	7881.322091	7514.251111	8570.841923	7989.219	6989.796	7099.478516
8633.025337	17550.36296	14490.79089	12496.4016	16747.58818	13236.69	17860.66483
31914.97454	20886.76446	20256.60099	19304.61888	19462.06502	19292.62905	20308.79028
151122.7144	152911.6576	155204.9408	150834.654	143521.2418	141331.3351	145993.76
129759.7312	126895.3519	117968.9421	125225.714	124872.6876	115345.8345	121979.0188
365449.8743	231785.9548	227192.8065	249747.4818	227993.1387	224143.5386	211474.816
197784.2445	199382.8267	210946.142	115721.7278	190138.5	200199.1886	205409.61
64607.5416	92131.53975	102873.4179	107067.7685	105723.7171	105232.1529	110471.1609
140299.7432	174462.9503	171194.5187	210855.4671	213793.2356	172058.3682	193614.8966
41278.95138	40048.87981	39328.32	37621.451	43175.08931	40605.10559	37070.7835

908310.6424	1092717.212	1104536.133	1056124.977	1098083.551	1068796.432	1087553.023
117914.0619	181684.7518	188986.7969	183603.1583	177136.3562	183657.1864	186354.6375
61532.588	142844.0269	146030.2675	143672.0388	148291.1355	142661.8109	139862.9447
7707.090455	9370.549091	10586.558	8812.477333	9666.06	9482.3625	9374.090909
174539.7507	62035.35073	56496.73556	148054.0425	59378.484	58707.44	59150.96
7621.6938	26736.94408	26485.60364	22830.13667	25282.60681	26363.68457	25506.81641
16287.6758	31937.34963	24075.11547	20267.02519	33362.72	20517.40044	22191.75749
10730.99815	24213.6756	17744.9055	16452.29	15478.31701	15255.5975	13737.75531
18085.87554	25524.83867	30812.59597	26126.93781	26168.16229	24179.76712	28959.01623
64730.50264	56660.6672	56918.17964	61002.14861	55350.05467	53369.71653	54761.60727
139108.5924	147683.488	144291.03	178979.4203	207645.0589	165577.8534	145619.8408
99091.1438	113135.5669	140944.4899	113398.3832	115542.0917	125369.1091	130751.7629
28299.55811	28573.92922	26697.6275	35213.35143	31720.76959	31743.23093	29551.02309
3435907.59	4108182.811	4182049.423	4263227.02	4128596.879	4172841.625	4168101.652
3387.737779	4418.994583	4890.5355	4326.698417	6009.444987	4603.106538	4997.072
770300.5027	1083589.285	978148.48	1001095.398	1054403.705	1014886.383	1016792.985
264377.8082	233091.7346	255203.6805	264388.6166	292399.1396	262067.2036	265008.6446
351080.0067	399519.0436	410444.2971	382035.6679	389997.9327	385679.6198	376889.2501
606425.2191	628620.72	641831.1474	626468.5969	609598.3415	619649.5098	642493.1165
152395.3753	198757.2729	256309.2498	186531.9546	183852.0143	208452.829	251485.4795
25482.02727	44687.481	45535.51642	38762.13936	42529.72447	38881.07036	41292.668
0	3483.04	4665.09725	3941.05011	4953.52	2405.121826	2769.36
45422.7795	56574.38693	61495.62517	68673.5711	66721.98745	55906.57659	47519.58971
11197.19718	15097.02989	13385.87492	13846.75435	16083.71338	13590.18223	14371.79178
54982.78571	75663.315	82920.39333	98666.1573	73528.9419	87282.806	78441.2235
202864.1552	145691.382	118974.9609	126129.5644	127130.6211	296093.6387	295302.9191
150447.1272	353585.5864	275474.2522	275879.6448	260960.0885	259992.1077	271681.2
253011.2383	466229.298	507634.5293	502970.4407	504062.6169	526361.1259	500998.3566
1099606.289	1410833.541	1417988.105	1439909.946	1416762.325	1439313.889	1402905.968
850518.03	851963.6028	1025162.88	838157.8324	950204.4313	1000809.411	848477.4032
2486.751377	3286.995789	3126.494202	3347.150533	3014.33256	2592.765091	3603.606812
341160.2348	221208.792	275096.9558	260687.373	229855.0076	252855.763	243598.5249
1733412.624	1858235.962	1866748.159	1826024.273	1818073.875	1811202.402	1814906.399
68706.6798	56016.342	56516.14667	72268.22455	58440.84444	65184.692	61716.1151
2387125.61	2706526.602	2596949.896	2629835.72	2635049.627	2539998.353	2567470.714
159439.875	125201.3809	120368.5758	131554.9246	128371.7832	129410.1923	127622.0212
32472.04119	40497.78525	41049.84969	39895.59346	39276.23475	38792.9325	40507.56962
186874.7981	153535.095	149846.4584	151554.825	141824.455	150946.5201	286537.9212
214074.6236	211384.913	236547.6873	196319.759	215646.8175	208594.8682	180003.106
1639744.941	2508298.241	2269850.87	2324575.316	2440074.573	2173707.234	2245106.952
33749.75411	61466.45268	61878.39722	63177.87727	67911.89941	65181.70504	69313.69928
55533.29371	48985.41862	51393.29463	50610.72884	48013.33868	49598.24766	55398.76942
83056.554	39272.05333	40762.83277	40950.8	43333.82718	38124.99227	42102.7365
347441.6946	675441.0604	663617.0143	647544.3909	266865.2538	648689.7718	335177.6253
690524.048	481746.4929	571977.5705	577335.7765	475616.3516	462621.6693	503189.1562
71300.3135	25502.93833	23289.27785	21609.81939	25780.34443	19632.44186	13892.16245
725214.049	1429737.45	1237222.555	1265223.814	1445048.605	1254041.326	1280249.196
137935.3724	306158.4839	309569.2049	309454.7401	181950.2526	176652.5143	311226.4453

44222.26154	52100.14846	70634.00872	51334.31208	55047.77353	55474.4225	49258.907
1596402.714	1982300.985	1964341.817	2015926.687	1967077.995	1970724.182	1937856.165
433073.3162	374208.7904	380805.9441	382397.8255	371725.2748	378768.1753	471961.3024
1409020.26	1167436.591	1142417.14	1159524.496	1151593.269	1133984.865	1148997.761
26113699.09	32030114.81	31680124.87	31193594.43	30589093.78	31043097.67	31633791.88
681691.2109	651156.9054	676007.2562	657413.9086	636680.3419	660000.0855	661606.0664
699429.9072	1272870.989	1891388.533	1339639.008	681484.6508	1235707.968	1272764.263
37962.79663	69195.58948	43737.40336	42073.59423	45877.97029	52323.76394	58148.73651
224254.1321	224202.4718	151001.8396	223160.9234	207318.7367	148603.7097	139117.9406
1518332.582	2107249.165	2098692.942	2182642.098	2061310.125	2042925.17	2056445.981
39971.88962	54359.95938	57233.5764	52836.51913	54962.12444	56514.70521	52660.1528
18543.24383	18186.75768	15501.62954	17751.11667	18506.60959	17884.977	12631.7565
378529.308	828161.1785	505781.513	463609.9208	472869.4684	502263.4692	500620.8805
477974.1081	495413.8167	519514.4405	495061.3445	484873.7869	485191.1708	479907.3406
165845.5392	239765.8737	213957.735	277353.4449	265722.5685	232478.4794	231231.2389
123600.0037	85635.95432	112514.8892	71247.20839	75469.80184	117742.9204	107245.0278
300241.0585	480624.8522	456262.5157	464525.6629	462096.5066	477738.4544	461788.7688
67971.98712	68792.57023	71902.70382	64541.1786	64884.716	68139.56415	78103.5003
1597139.674	1576137.963	1409336.455	2805553.793	1573384.129	1513966.393	2731795.153
2868960.577	4169263.104	4256938.88	4272436.208	4070017.267	4020981.21	4105085.356
4885609.115	4243690.147	4325959.467	4246505.206	4705244.541	4187710.229	4216427.728
17118342.46	20703757.48	20978553.52	21723094.21	20812744.86	21205561.52	20722393.61
157936.9061	200902.163	251316.363	217703.506	224979.2565	183200.093	208124.917
109149.864	213953.6403	203286.5663	207530.987	261680.9431	227149.857	205262.1417
57327.254	64359.501	64051.85783	56685.5148	61423.24377	61836.82094	64465.57999
31184.66418	16701.01	16349.1475	31778.95802	31024.76676	30415.79591	16513.40271
4024698	4779265.889	4658984.249	4779242.336	6333575.605	4777139.457	4822750.975
601749.6705	959239.8866	1037844.392	1052350.013	968303.9643	1138229.824	1004229.588
198799.6674	257850.4929	241573.4456	250045.414	231198.7122	239522.274	232402.1484
5948.703457	7208.858032	4393.125	2852.534	4732.3125	2519.857143	4102.55526
6358690.051	9040605.841	8616820.651	9727862.892	8704919.524	8877382.192	8858829.156
16797123.38	25453097.5	36157775.49	36725493.36	35834999.65	36794816.09	25791135.22
145794.2564	127718.4105	157814.2984	145235.9597	128820.8235	141987.0593	138311.1205
247535.0587	279318.1394	292131.7196	287650.0076	285288.4204	311588.3667	316220.2735
219556.5475	287775.9526	378813.58	270859.7587	276773.4736	283012.794	273586.8581
8461.884692	16216.96774	15180.69762	15354.10779	14908.21486	14554.98942	14656.5669
74048.60077	6407.23	7337.92	7965.14	8945.08	6683.578	9078.9298
833856.7801	591613.65	599312.5058	573453.754	563498.3733	506273.09	623065.8907
48897.891	88517.85683	80234.90585	82183.66325	83290.18223	82871.27883	112249.2508
59477.2784	50275.22569	78162.64624	60282.64	73683.3627	47855.2261	56249.59612
347803.7382	1298459.169	1296488.063	1302039.364	1289294.125	1331095.5	1339035.172
24607.75991	27423.62975	29324.12891	33811.54936	24826.42567	24088.3357	15806.004
7642703.671	10450920.62	10357700.19	10218303.82	10073119.89	10584245.08	10255421.29
4371851.063	5743851.087	5388197.198	5768852.802	5680373.531	5674177.275	5626425.811
213380.7756	340920.8279	379106.9966	217909.9	208629.0903	213452.0025	179526.0105
146922.8916	35853.37772	20040.91737	34890.26348	76439.7193	1303.180612	41790.93337
1266431.343	1666750.47	1726555.612	1779946.138	1741803.947	1744378.984	1736433.464
5494412.213	7375742.805	7495120.785	7600358.294	7548982.573	7802914.951	7456806.728

66283.26347	111082.5018	116708.7042	118113.6941	102848.9849	121649.9537	113137.842
5422747.805	7397357.439	7414390.644	7507184.958	7514323.886	7444508.688	7379206.433
740915.4429	2134093.558	1350560.509	996321.5037	1006997.023	974334.2904	1329728.279
2015794.71	2210509.976	2254521.213	2317143.083	2235808.88	2348729.625	2236812.746
4491.283401	9738.5402	19641.44332	12270.71777	12742.71266	14869.49063	14739.78298
292995.2325	331489.1245	346473.4768	316526.2426	326027.3281	305718.5489	317556.5049
5261995.566	6211830.652	6480883.072	6209318.938	1286.533947	994.7949219	1688.1961
1056787.926	1190924.451	1120355.88	1090461.178	1006399.34	1094478.327	1122115.549
12022.79129	19311.02941	19369.49561	19015.8371	18048.82231	18311.68956	19480.64063
12416720.58	16633663.97	16920538.73	17008657.92	16832427.14	16612036.52	17077316.63
1222391.762	1188892.586	1201166.676	1187778.856	1132494.664	1146620.662	1176049.842
15470.7344	19240.35771	13444.65997	13104.87343	10102.94545	15649.38686	9445.669
61205.06762	87755.33195	79368.21738	76020.4445	80566.68737	78442.36706	79027.9409
4903807.345	4447679.796	4499253.356	4505959.171	4470674	4437456.739	4396048.443
6747304.877	5718973.545	5172220.353	6081413.243	6006963.692	6006277.383	5863072.331
500.6798503	806.3025513	736.805013	527.2042169	305.1386176	2570.487929	2188.749457
672661.8641	692040.8165	686605.5219	675876.6107	708944.6577	694721.9248	699802.325
11756270.31	14031526.18	14054379.24	14211524.08	13769374.26	13720585.65	13840305.94
799624.2374	821633.4419	893465.5555	862380.1629	810118.4572	829851.638	805111.1209
279130.0945	296883.0347	312524.541	303629.5083	292396.6777	298609.7893	307270.7394
841096.5803	724536.1357	643955.8552	737881.5426	696675.8831	739932.8552	699800.7376
666268.0314	883925.2096	813013.7945	827515.827	833619.6939	805092.8954	771272.5091
9331902.301	13573808.06	13408523.2	13295489.79	13620850.51	14179718	13950435.99
31904.92587	29315.53345	31777.30438	33696.30682	30399.67969	1950.96	30882.51929
375704.581	413567.4382	422996.9071	431120.8933	416878.341	339599.1648	405374.5477
470949.7543	521210.9747	549246.5939	532345.4666	534105.5975	535311.5404	525599.7803
15224781	16218137.5	15626187.78	15685458.01	15645157.49	935.1970379	2009.448007
129758.4476	168364.6674	92496.3039	95516.415	109612.1346	169413.9401	165520.2274
101088.0157	104112.0321	120490.192	114137.4677	124924.356	120215.3807	126624.1139
2279.037069	2387.601868	2301.136115	1632.2976	2467.940158	2258.180291	2217.463949
143744.3963	217220.8991	227151.2703	240823.0363	222061.5788	223532.4143	217541.6685
350516.2391	251785.3589	268443.7282	271341.7837	249940.1106	262058.7924	253247.8136
11126207.51	11233301.18	11257028.28	12064139.88	10684404.55	11086406.15	10751897.28
425618.8566	602305.7794	617552.7473	589120.5346	627453.042	592597.0001	615298.2372
270394.0479	199725.3673	197080.1903	184103.5272	190375.495	182633.3526	188342.8777
9000818.126	13558447.47	12793539.22	13284289.89	13452237.31	13029970.77	13127193.24
51842.42456	39920.92073	46521.40226	44856.92175	40262.76733	41725.9705	73933.00064
455148.1547	446483.9128	473588.0322	373089.4735	394868.3924	459000.9094	403372.0845
334008.9528	103169.8936	43208.18665	50541.15988	18038.86703	24707.07901	20748.63965
9275311.517	10764798	10485520.99	10549823.68	10393029.36	10400005.14	10578189.31
525645.1484	576929.0394	582273.4311	939975.8088	793196.2761	578936.9028	584055.8193
8959923.981	10865527.84	10583132.96	10728039.69	10389381.01	10639129.62	10746655.83
46046.13695	156914.7772	139678.7087	144778.8195	115553.4344	141646.3537	139788.3703
73954.14	87538.16537	102288.0661	92109.6638	63821.63	98849.01085	1352499.866
77178.58269	440507.3906	456561.5313	421012.6355	424735.8305	446461.8737	440320.0598
371863.6639	460076.4292	195887.7437	480144.3316	155022.7264	162055.3233	163006.0604
1567607.519	2340082.923	2338047.827	2367355.658	2378837.464	2650793.579	2287192.765
21470511.39	26492352.46	26833212	26773338.83	25920204.5	26516128.61	26919424.33

2619104.304	2473933.709	2439543.208	2638431.704	3054322.845	2722624.99	2567350.652
292862.9763	438304.5383	442766.9226	469260.4862	494086.3421	446248.2563	473339.7236
51839.874	73851.54682	104259.9009	66764.22308	82919.881	74855.51662	14402.7794
479259.5851	227.3971837	1973127.505	1987785.759	0	150168.1682	246.6598511
540189.4356	682487.1919	695342.8017	681207.7518	697682.5883	678919.7534	690483.2001
929522.6721	725951.0334	892691.5147	429305.9812	210177.9402	2343528.091	84993.85602
4003504.34	5842045.578	6439928.029	6179147.466	5915961.688	5559576.134	1343681.482
160480.5567	200288.3167	219580.9905	198186.8942	157499.0928	212130.8787	217172.1525
644165.1443	821492.7555	792227.3565	836106.4265	769077.7997	786806.2603	794627.6389
523118.7656	671392.3778	638749.4342	681059.7298	667398.7181	628823.0441	642928.0398
338255.021	481868.1382	535001.7843	484980.3932	497498.5278	506040.6346	501575.2711
32481.03334	25693.05933	21832.3775	26235.9	21542.66667	24391.96429	26373.65667
1561816.249	1650534.295	1616794.308	1573169.135	1614626.755	1608034.114	1595410.646
38243.42257	51949.63474	75129.30859	68676.35872	74985.31546	69046.76433	71954.32269
39006.63062	38902.41925	37983.6	54837.78804	35620.58147	37526.28218	36699.56718
570522.911	1026689.221	1039853.085	1058041.803	969664.3785	979216.6579	996573.935
87295.59185	77193.37555	68318.33366	65409.40741	54816.23808	73324.39615	66477.99516
1940718.632	3339445.956	3556566.5	3449836.478	3147927.047	3236798.601	3373751.042
157984.3157	92465.15453	119644.7067	114300.7269	76955.97331	72906.70154	109679.4297
9246.501143	11829.44444	2409.000633	14064.4025	1873.658985	11517.89855	5093.5354
457694.3725	448374.5016	354101.9079	349976.0554	353535.8381	337489.7113	336340.8791
6041954.626	9580921.855	10029661.7	9485447.955	9132746.589	9787573.219	9736293.024
2219678.902	998221.84	1042364.882	1032011.731	1002088.883	178201.7198	991402.1296
1161339.922	1760320.347	1792253.721	1800078.387	1770668.398	1727094.753	1772738.13
11652.36869	16026.55806	15345.54158	14881.22932	15981.53374	15359.00706	14374.57021
1175441.186	767501.3077	728634.3646	728123.1473	667942.4985	686115.6998	784763.1715
261334.8066	440362.2195	348991.6024	343481.2714	326508.4421	378419.0081	353434.8532
60578.94518	92747.4338	76593.84923	94464.19427	99460.99146	97870.13749	78388.92415
1176220.468	1434585.902	1530023.244	1543131.771	1463638.874	1519027.976	1530693.972
403938.1418	405335.3224	408168.853	409968.9525	412528.8886	410295.665	418421.8027
6319445.412	7522961.97	7143053.664	6365999.597	4791699.396	5764472.083	5500867.949
2871.862857	6227.587278	5245.7385	5529.088357	6501.324713	6729.035062	6419.454013
1617019.358	2221782.694	2154605.586	2214830.635	2156702.763	2266206.44	2289532.775
10573034.15	16004066.52	13830164.48	16259400.18	13878242.34	15998706.63	16151931.39
3601995.872	3358255.348	3618863.411	3436672.585	3541329.613	3285394.907	3414248.81
818409.496	821913.6928	831081.9843	802797.4658	796118.8431	800801.1	826536.5596
4424.285882	4260.730769	4557.808769	4077.648375	4735.182857	4745.710286	4092.918375
853264.6322	1138055.023	1161505.875	1209429.428	1144874.12	1090204.168	1153728.629
11562072.16	13150162.81	17024712.88	13223958.76	12750419.54	13076361.05	13015902.93
358018.6562	525419.1961	558787.0029	523754.1105	552838.2581	554486.3437	530008.6629
540554.993	472722.0052	492944.4939	51367.96846	2419.574707	2830.491561	10567.94107
981696.5265	1066665.17	1071440.252	985662.6097	1100037.594	1030197.847	963810.32
818896.3488	1489169.99	1349752.892	1260070.346	1109694.307	1208075.468	1398814.235
902582.7357	701660.87	721060.8633	759205.269	664933.3727	700293.125	701936.1172
607.3748498	564.1325543	247.260275	937.5092773	653.8076172	640.0532532	1181.579485
6598793.477	7321557.284	6967613.889	6917562.661	6890241.258	6760515.098	7111097.65
210640.6917	368043.5504	385251.0222	374534.7481	362794.2821	373712.337	378885.0431
21232.3527	15786.93143	27117.95667	22090.625	16902.90186	21984.74868	23932.13889

3577790.829	3759515.835	3950743.955	3807339.447	3775604.031	3821306.896	3755031.263
92846.09851	113577.4124	119317.8837	115777.0276	118860.029	113707.3929	114684.3004
4725348.056	7993953.424	7292340.731	7255693.761	7117907.75	6959373.236	7115966.481
104530.2042	69044.45123	73172.62154	76192.47523	104937.17	78190.53986	75929.51721
41930.12948	56294.97543	58930.67314	57664.59141	59405.88867	56906.75475	58424.42027
9703701.381	10057348.54	10363159.43	10098485.72	10257820.84	9970514.081	10155730.32
70389.13636	65059.66857	78462.86486	94965.98279	81143.35776	93156.16097	92786.55634
671764.047	1458369.912	948757.9605	2350158.477	1062133.236	1012351.246	2268065.583
272955.24	272161.057	282400.1964	273692.4533	319969.9378	275317.2364	284719.5175
127984.2806	84841.16182	88611.51509	87540.58333	88526.73354	79076.51462	92697.99758
47256.06914	71488.95464	76490.26089	72530.55707	73329.47814	83074.86341	72235.86231
83990.09427	102728.5478	161671.884	106711.1171	168559.9154	100109.1552	95450.08829
131318.5453	83090.26588	80912.41763	88068.33	88982.04124	90061.71009	86577.29226
29849.83014	37446.8716	41589.96165	44872.13683	36294.117	39382.96253	39392.83736
4680668.795	4110891.428	4094343.712	3944946.218	3985451.748	4001083.399	4030387.122
396793.6576	304573.1462	286301.5268	204875.5961	284700.7844	286705.0296	284908.4132
50332.79421	62319.2285	70858.60097	74916.79992	73215.66472	59476.52201	66308.80417
148665.0451	337639.3796	332718.5547	348184.5665	329336.3483	309944.5761	319096.623
238334.545	378778.8817	410486.5145	410463.4443	391208.8657	380524.3432	373167.4752
646550.9865	551404.7211	534325.2794	530388.4382	526611.8803	542532.6027	504051.5454
95917.27634	100850.0102	86365.91732	86106.26358	80485.6597	80859.0787	77690.61642
33279.80814	105859.3394	101152.5386	103437.9816	103557.8189	99949.93482	97923.67609
700859.9813	607617.7699	661003.6867	603733.9145	595081.9776	594523.2587	577331.713
61424.98875	53728.18487	65355.59489	60766.975	57221.34125	80665.1835	74298.86256
122526.0324	62370.59869	60092.39844	61983.21668	114371.2317	56529.54493	56198.24758
134350.1346	78454.7492	93908.84895	79765.93886	83304.49614	88560.46324	78362.8385
44135.79927	68953.8803	59339.49731	50103.216	54889.64333	51089.8574	54408.72
464899.1981	538378.4125	537975.842	516279.7962	21126.00911	489254.6881	511615.8695
28325.0136	23039.08413	19643.52295	18269.90282	22417.02654	23128.53263	20888.30821
298434.4592	378987.8289	398260.962	362560.8285	377792.802	362465.6905	380056.7744
2546609.692	2849073.099	2909034.498	2829439.375	2822871.583	2805143.587	2872785.363
2586468.864	3355294.256	3415028.355	3372490.355	3360193.38	3176645.524	3295471.667
327231.0886	885559.7172	261939.4347	239099.0911	231187.3822	853676.1735	263056.11
16910.43205	11719.12116	14369.66972	13182.35535	12917.95013	14036.46091	14442.55215
112040.9603	149215.1904	76402.11244	155506.444	98680.88862	111591.7606	60195.1813
32289.16864	22336.22221	22453.07955	17447.30867	27070.93494	16790.15625	18020.25073
591170.0797	761660.1	735539.1705	743974.0926	729247.4976	740528.2839	747251.4273
259812.2175	356323.44	352363.5885	340567.04	367618.0806	352234.96	338410.16
534850.8286	667369.6506	295107.6938	650167.7937	628620.8439	825307.6138	627024.3005
212372.8699	288213.3665	301066.0619	290110.265	266060.2966	267760.3616	296236.8799
4676.330294	2939.795882	2931.276	2619.605215	3122.042297	3420.759568	2956.148099
1726289.644	2032148.572	2110910.461	2104279.296	2082567.291	2054377.016	2125923.193
15360.90601	7641.843667	13597.76	7681.338667	14361.99983	13965.43501	13054.22521
106243.6072	136105.8009	115838.0054	127001.5383	104119.9128	101275.0602	111568.4953
53929.1048	68351.38078	66217.33472	59615.8785	61145.03314	65564.81096	58778.58397
48924.79564	42230.52742	45605.88199	45004.30743	43834.90854	43243.96563	44443.63919
10557161.31	8758145.129	8978925.098	8825894.514	8444119.504	8746105.117	8634154.503
162451.7288	205855.1748	213438.582	214550.9046	210343.5141	292536.9088	194791.04

81414.452	97961.25225	91577.99508	94218.3375	99562.15667	90611.353	95277.40365
81294.01467	89830.20316	76599.23149	81886.74253	86585.93265	90917.67009	88204.025
132112.4262	222378.4721	232394.1493	218765.8487	343849.3098	199395.7791	213413.4376
189186.1989	101473.5209	99891.44648	102127.6432	102815.5206	104955.9978	99567.72548
71357.98755	61865.65623	49618.7128	40121.09105	71126.29391	63021.66407	62262.68988
42404485.3	39306243.07	39168432.25	38357015.35	38209534.28	38231451.98	38411932.35
687816.6452	645373.981	635698.0172	618480.9118	636766.4249	625306.4988	641595.7375
47413.37802	58735.16306	57687.56842	61674.249	61594.39292	58411.68027	65951.87625
168376.3032	136757.0694	136323.149	130724.0421	132004.0902	138514.9362	95162.709
129621.2785	153207.564	126973.9688	106458.3648	134229.6971	124163.3368	122811.3938
870239.161	587398.5163	570030.4944	560071.0686	576204.9005	544760.8321	564235.8069
42746.68675	73787.84692	72883.08625	65235.32	71205.37901	65941.6938	76681.08
257288.4471	278220.5273	282204.3087	273635.9916	297857.4415	282443.0362	283972.7426
318588.619	77468.37493	38395.4253	75560.52138	71303.07163	76247.97744	73636.94171
100765.214	121559.5206	119440.6101	128181.0764	124640.1499	119103.1863	118220.9695
228174.6095	246947.3856	270210.3734	237468.4706	261866.3442	249005.7734	250283.985
26221.64833	23767.917	20561.085	23431.88701	19406.73514	17275.959	22365.10588
3853934.851	4491193.447	4475415.728	4514354.509	4298725.117	4400897.282	4490861.782
196506.5142	57069.3175	66156.216	56941.92	95139.49233	95786.70478	98252.253
65744.51824	71821.70156	70906.48813	88363.99217	64407.60027	66270.12238	69354.03394
483391.7958	440119.7242	367128.8198	394733.3798	373090.0424	369348.9733	386465.8416
227509.1373	180552.0789	195586.1417	137885.3933	140006.1667	207202.8424	199791.79
13862.32446	16194.9699	18107.0625	14410.243	17613.15476	14256.06468	19750.89156
13196.7128	18538.69536	18247.76966	14539.39409	18613.50909	13732.6475	17797.00781
9759.08	13965.74567	16918.29414	14274.5175	13440.56685	11723.83106	13704.96229
108150.5385	119660.7067	104196.0247	101491.0622	113445.6167	117315.3462	128813.511
24561.23192	21825.2468	22201.7849	23294.22837	22303.102	20020.15695	21626.33062
21351.54654	43494.09364	44721.13676	46972.06713	39803.32038	44088.96693	45414.732
249809.7449	56931.14196	254769.3506	253423.1923	243056.2506	257762.878	39302.87077
633712.6598	67719.05714	228186.7508	41893.77	241569.5531	406236.5739	208091.5736
9928.053	8363.638867	8515.980286	6900.659091	7302.027333	7332.728375	8219.587542
325021.4938	41905.49363	209721.5415	209326.692	36771.84665	211865.5756	189814.6729
13691.64764	10863.93109	12328.28253	9631.582616	11682.412	11774.92721	11382.2743
27442.644	13935.67083	8946.99	8542.873727	9986.755556	8225.925556	12223.25533
130068.6593	137582.3839	127195.8545	145340.2354	137999.433	153002.1667	144106.16
23742.90625	20507.29288	12990.444	12484.528	14145.4425	17852.20645	14462.16233
32295.94378	14170.998	26305.33472	29827.79789	22994.87305	11784.322	20384.56624
27430134.05	25140044.39	25661197.13	24434106.86	25487957.8	24957591.7	25584778.77
181092.9463	186480.8617	190697.7623	215581.9515	229775.92	189596.8532	214555.1635
807697.5807	144015.5509	157819.5632	150631.922	149643.4012	148379.8387	146129.0833
29461.79169	16156.17229	19802.05432	17163.55465	20922.54578	20196.06937	9435.3506
297043.7564	264821.037	262961.8131	286525.0185	263676.5915	256181.1891	256194.7752
11911.6951	32424.91566	34703.26575	34756.18726	28801.70205	34203.36281	32781.45367
136225.2619	176344.488	163913.8306	166533.275	169179.4792	164662.7489	170182.7865
823757.0868	528790.2963	504188.0386	496868.9452	500916.3958	500100.9139	507111.8329
98357.76775	72360.78037	77848.2019	82071.99062	75241.27497	75301.76871	80476.84591
274718.3257	337519.8927	347532.6463	370157.1674	383380.2723	369494.5937	345453.9678
53329.50109	68854.86597	62809.89573	65684.46133	65277.5025	67205.703	64106.62688

31365.48669	22955.24531	22420.89743	26628.68337	21334.36714	26341.15631	16844.3865
277867.1196	86784.419	221086.9086	104663.3846	188655.4793	125911.9178	112455.4205
10747.8	8391.25625	8974.35	16241.89938	19303.35502	20156.67554	6942.422
25784.33075	5106.192857	4890.113167	15760.42991	15579.16743	15798.8127	3765.116
30565.557	16400.79625	18798.03284	19497.34656	18554.17351	19977.25	19728.83189
90695.83381	23588.8345	72930.01692	7914.629125	71986.91038	69759.4294	69201.02816
146648.2915	108047.6043	112213.1931	49184.81587	47547.45625	46330.69375	91048.50769
305636.7846	251140.0221	283298.5255	282105.5866	249907.644	607338.2705	251006.7105
303033.4359	244653.3183	240175.7984	208518.3356	219303.9696	197568.545	238391.7329
79864.15323	74010.84283	75262.54056	66493.21818	71100.85599	69178.67305	75177.81317
806887.6569	454513.9374	491664.7827	441659.1797	455055.2382	452751.7741	459678.7676
214834.5754	227801.988	229241.7542	227019.0274	229554.1993	245652.0847	246279.0844
809310.9693	251262.4654	95501.24355	133373.1234	102172.7118	128192.9818	31614.89676
10684.55517	12971.081	30919.9616	13777.76714	13261.11436	13148.66474	13990.86039
746678.2735	598750.506	522099.1721	565956.5805	538434.3396	515191.18	478383.5913
11508.52944	5634.125	4489.8075	4956.880143	6728.366743	4373.68	5034.385714
50529.6	47547.86349	49983.97115	44947.63701	47294.69839	49118.74821	44549.38516
10014.77924	3936.248875	5378.727857	6426.495697	6048.749756	6337.448026	5070.711375
70969.17325	64217.02356	74221.32458	90127.68907	64044.04139	66381.17988	93861.15
525327.6853	101273.3931	288186.1782	292312.2402	67116.306	103300.8071	94553.25712
3181.600122	2355.545532	2338.742082	2175.570123	2640.710266	2288.765625	2373.917691
98153.5651	79158.16684	78911.93792	78700.43001	82139.97665	75413.82873	77998.3399
48391.29275	31283.385	26648.7884	29076.42565	30939.15865	27284.4	24006.17492
49189865.15	36714952.24	36295037.01	35492894.56	35039977.78	34638487.61	35672726.81
488906.88	512033.2463	542344.6853	563900.8107	542570.3293	538555.0074	500102.5759
504685.7741	449821.1523	495918.3156	474571.3674	494911.4513	387639	353169.414
343095.28	331626.67	358793.3527	319168.8597	339168.2235	306092.1311	331742.7309
27571.61818	36716.79485	29907.80753	24727.40531	27725.79423	27490.68734	31748.03129
279673.3682	239474.763	236145.5857	233881.0012	261970.786	230463.5671	253139.0645
5328.327667	8744.808938	9458.36	10435.04161	12500.595	9554.980167	9747.819197
245748.8045	258245.232	194358.4496	172158.9049	195968.5069	186032.43	200513.6258
39424.095	66599.62097	73959.63263	25942.032	30412.252	44091.97257	85588.23706
37185.82638	32722.61478	31202.05549	32987.76218	29123.95137	31930.10232	31682.68136
381346.7552	267771.907	230010.2963	222181.0141	210366.8035	206584.2398	222923.4422
430282.3815	394686.6981	388627.8955	375085.2734	384677.9521	364027.9853	383935.9669
52866.438	44074.94143	44247.84	58134.17767	56591.99742	52899.54281	51975.02625
90793.91	55003.488	52718.5149	86822.9835	54392.69	52220.9002	50672.91
94829.08	76647.72591	80314.89099	86584.51277	82348.9173	90219.74609	93244.93757
644933.819	319675.412	379901.5193	315654.4626	288176.2542	276346.9331	335381.6035
2079.203	2738.79075	2579.375244	2719.999945	2434.589747	2182.732488	2040.406067
197329.4486	123521.0593	39867.95625	120059.5199	124695.0632	105609.5233	70435.90145
71447.48456	61572.12068	61374.57882	58868.33498	58509.75555	60556.07611	58488.87213
608216.8579	374642.2489	141336.3483	120134.041	246837.7863	135084.7664	153722.4298
67047653.69	44189366.67	42774818.48	41691406.78	42608869.81	42061064.07	42724991.09
520221.3009	376160.6417	379590.3885	398295.3206	373829.5015	356024.7565	369294.6908
43794.69797	22458.24425	29572.075	18583.91719	29219.7885	20224.17007	14040.4275
1409677.28	966118.8291	961054.8983	937159.1402	931321.745	907901.4285	979106.7933
26365120.04	21094493.68	20017925.15	19313914.77	19717813.2	19589165.97	19657525.44

140166.4619	291651.0969	289047.9551	301656.8527	296825.0056	289050.7525	288226.5284
345480.2241	421486.9495	395230.9091	385310.1379	418271.6551	388071.4275	410665.748
577989.9152	842689.3319	812870.5686	829971.8495	735935.2474	829947.0638	760685.384
253558.0696	200841.149	194912.5583	197999.2343	206516.9685	186285.5295	192754.8778
568275.5614	349649.1325	350440.6077	359343.1823	251956.8527	293889.5912	304615.5793
145546.5567	407453.2379	403305.7228	411270.3377	403153.051	387389.0978	392531.7913
1391007.323	806409.7046	759453.021	760609.8704	745712.9886	751406.873	771576.6132
52021.46167	272530.7655	51370.40642	248377.0166	48886.80836	49477.93924	42375.95226
600337.1824	393187.8869	381967.8013	355897.4059	59679.85757	358842.0412	378545.5803
145305.8125	85685.39223	80331.65866	76539.49879	72612.09221	79794.32395	79746.70929
161160.2644	104668.2916	102401.4701	11473.09215	91934.50071	10110.04615	86380.64134
232522.1939	240683.046	199375.5115	205592.0728	217376.5219	213731.2495	200292.3042
694037.1524	338907.14	481922.7794	428416.1621	317220.2414	434773.8742	462300.8832
70886.3152	82235.21135	65058.08323	64066.2232	58147.01254	56378.71119	109251.3934
423361.4793	361997.2748	354702.3086	334628.4478	329895.672	358529.6459	346994.7714
157210.9065	32137.5285	19032.03343	29891.72482	22805.6972	30009.2718	92581.15602
16375.51556	23464.92909	99457.18115	26699.91231	96542.31792	46808.36316	90217.4453
5053.361023	8460.408	5994.854213	6930.756	5656.951416	12990.978	9812.578
27407.49771	59307.96346	39126.0544	34755.43603	36329.97083	36666.80742	35538.60502
352362.56	280102.611	410504.7678	246350.8702	255410.2858	235418.9193	236961.8271
16167.25232	19202.99492	15510.834	18861.1424	16719.07392	15923.13625	12641.05636
58221.48787	21259.7286	19819.904	51268.73855	56786.4388	47204.17485	18116.76138
83927.79125	60842.81832	51562.37679	65023.73173	70759.61272	72504.2995	73270.34
48509.75337	37013.78058	23865.29277	30365.6073	30844.28967	31456.52033	30821.23124
59633.02038	44501.35347	51132.9173	52112.71241	56624.52545	55608.99705	53884.67736
40398.16	48367.3626	44983.56188	36668.15746	44964.9396	45856.74712	80550.77772
13088.45944	5445.4532	10092.8037	4739.2164	9649.267535	6391.841064	5071.3475
183921.4239	480340.6386	486278.8791	485027.5252	478248.4326	488514.1689	486895.3172
5318.697647	10798.88289	12012.85167	10933.14283	10540.60013	10603.90694	11936.57238
68366.33647	197000.7443	191396.971	197502.8511	199700.3191	197982.9196	196920.7214
72143.68	126933.1221	125475.389	118366.8418	128143.7111	128439.0402	123085.42
9799.9187	18453.786	17950.94005	18459.13863	18801.40879	18543.85437	18919.17047
12351.17981	24369.50085	25156.60496	27623.73129	27618.39024	27779.17256	28038.17665
5193.1046	12077.80144	11117.18969	10742.01893	11836.63294	12473.36475	12466.97227
90355.40556	238255.9659	232808.2156	237777.3519	239994.8935	235049.3958	236225.2855
106854.0811	253916.6589	248896.4979	251829.914	255537.8488	253031.1674	257500.8712
176654.7999	472828.6609	487375.4358	482441.4145	478711.9823	496894.8645	497516.8181
2250.583333	7061.673	7177.855091	6433.314727	7009.826923	6047.465077	6687.458917
6588.964625	14448.31878	16796.09346	16434.89304	17176.04513	14549.76842	16974.90004
43778.13471	90444.89976	93793.66694	94933.89753	97894.06329	94154.115	95807.68729
21582.51456	44494.77805	43300.31497	41600.87524	40472.43571	40278.46871	46283.77326
5263.89808	8123.022235	16365.61208	8167.381588	8480.120353	7855.855059	8586.339789
13609.88658	30865.79925	31047.23987	33591.38513	31023.55167	32056.56518	28018.69852
22577.13695	55347.57641	59584.10079	50227.34409	53366.4495	57423.03436	56703.54924
9689.622	23028.4957	18700.73761	24020.74867	21059.48688	25574.114	26385.52535
24804.439	39096.72524	39396.50565	39960.09233	38527.23756	40300.43228	41018.8685
2742.072176	6242.879132	5660.804	5316.552	5718.230833	5263.141444	5655.871294
2345.535998	5935.028535	2741.828571	3460.170209	3122.120693	5851.648	3221.486571

290873.4191	742974.7758	763655.0223	772424.3329	757727.845	764963.1757	771971.0034
21604.49474	32373.50471	36561.83808	34596.3725	33013.04183	35252.528	36461.36789
68967.2925	117329.6344	119813.9428	142706.3125	112404.807	117677.7991	142370.0393
1505.457812	1302.725111	1586.3648	1044.135714	1611.430714	1593.631162	1523.1908
45511.71779	84868.82774	81880.41768	82498.56544	83878.266	83545.91358	85411.38396
619926.9751	1379361.286	1349636.352	1414572.823	1407778.32	1376701.708	1374607.102
5357.257778	8811.684667	8630.249375	8790.528	8480.401412	8947.368222	8358.524706
29257.86806	46163.57118	46906.49329	46198.58312	47308.781	48763.05408	47704.73194
30667.47678	5147.440412	4201.535615	5942.622667	2839.331	5053.027412	4716.505625
13766.76247	28370.38835	27426.398	30359.74085	29315.57	30357.13774	27475.94087
17041.641	19633.1694	19192.6725	19199.09933	20904.798	19369.10109	17565.29484
16583.77959	23899.439	26927.84	25763.135	24693.49906	25901.25233	26751.17167
429918.5294	1061031.588	1075602.31	1038254.858	1048897.665	1048362.84	1046964.17
460353.8669	951031.158	951772.2923	1008613.647	996370.9494	1016805.702	920835.9748
5932.127688	9283.436438	9057.010313	10245.48706	9360.283353	9623.696824	9538.578875
60454.74353	113356.5216	114855.7919	113594.576	114090.296	92502.46494	114502.4406
14880.83504	24349.9584	23992.63058	27992.866	29044.78217	25099.75005	23915.79416
24672.14128	50650.79663	50132.14489	48214.28218	46999.927	50332.34933	49670.09871
26044.6375	53708.655	54304.97755	53803.42926	50765.46865	55490.099	54541.4912
2954.153373	2424.2892	2006.549846	4014.612	2571.584857	2836.077802	2466.747294
26913.442	35299.62338	34666.31979	33409.91631	34174.27281	32626.35688	34309.27444
3186.211824	5640.506667	5850.542375	5180.00825	5407.30475	6085.106706	5644.81125
108345.0234	214626.083	216452.4583	207733.562	209868.474	211263.899	209231.6615
7650.618692	15892.50506	15181.73988	15142.0522	15202.28506	14676.44447	14627.336
2752.616095	5812.233143	6066.610316	6650.717129	6012.0115	5742.143	6003.368
4711.23	6841.626833	14848.10468	7560.779684	6962.311529	6666.079059	14705.32219
31808.88319	43467.69263	43255.90935	42849.73256	43176.6	43174.00482	41628.40894
21811.26544	8859.364412	9621.223111	8707.540833	9647.852294	8848.006	8603.923217
546.5117273	929.2725	821.3687143	936.802	523.4057007	1284.916304	582.3675
9348.476667	7330.146267	7548.8	7923.113563	7322.772667	8006.32875	7228.58
6087.298778	8643.538	5357.4015	8873.822298	6189.3304	6220.980522	8318.482054
6605.533687	7045.636	7253.82	7081.268333	8372.033333	8031.472105	7925.630176
3240.258421	4462.232211	4601.127833	4131.611765	4087.491333	4189.567556	4198.129941
8340.576636	9232.3073	10356.82867	8854.8105	10344.68532	11233.36871	9755.94
53202.47039	38351.56071	35352.56144	33417.69706	35417.85317	40322.52463	38125.9185
778.6187973	956.1807	860.223	905.8150101	818.5572416	1026.230111	2020.451659
73828.74063	61226.57383	57863.7055	60369.703	61451.62833	59715.58926	60160.38617
6865.405556	7827.032647	8686.592	7581.632333	7663.290313	8550.99	7679.815824
54137.95941	65527.22571	64677.74147	67427.05238	67116.07339	68085.49959	64823.05878
3787.744167	5162.626842	3568.416429	4580.7445	4264.009235	7370.857867	5261.753304
37565.47538	42313.51912	42208.40138	40745.27694	42828.882	40358.8504	41240.49131
17179.05708	14569.33296	13710.70546	16219.56556	15127.373	13538.08744	16379.97648
9896.787353	9666.141588	11194.39375	9054.84375	8611.780875	9308.402125	9622.473188
6454.7106	8751.696	8036.9187	7466.962543	6950.582059	7861.263235	7695.39765
9262.7135	10209.23269	8892.239294	9520.64175	10109.36647	10641.92929	10699.96659
32664.42266	22302.69388	29486.34848	29071.29868	28045.54557	24590.07833	22611.02213
39377.88517	44675.40018	48309.67382	48180.48156	45412.26133	47889.77194	47720.59429
5347.857316	4148.272059	4313.1725	3827.1038	5086.337	4099.461222	4523.681619

1419.837608	2509.839266	2660.788737	2664.438833	2502.41895	2768.837433	2832.626
98117.95063	65162.34267	64134.658	68463.86258	63600.72006	65371.119	64585.69994
56882.8161	59667.28479	55248.78032	62947.03333	59721.21211	71840.25053	88788.82788
23226.588	39573.40663	39341.68565	37930.992	73322.22772	38874.93322	36198.52294
6098.054071	8149.454357	7662.072678	5472.106667	7698.675	7087.051178	8185.426894
354733.755	349602.5068	353430.7035	349526.5434	358754.3518	361906.1665	351725.9175
7084.256584	8889.025833	8061.290188	8341.18875	8828.496333	7849.902688	8410.434389
18819.50606	32127.04219	33989.088	32882.22169	30540.95038	31664.17275	30078.51713
24661.49558	37282.33095	36278.6574	35786.7878	34343.59095	38165.32833	35686.7097
6934.5735	10184.41147	10393.12197	12819.75295	11430.09253	11531.89475	11869.45636
4627.791737	6912.192	5635.065571	9591.359333	9898.893163	6416.333842	7042.625548
131850.4926	162347.3957	151879.2059	153821.2885	159710.6711	163412.7099	156615.9371
69429.211	84831.7445	79784.89059	83891.682	82272.14259	77028.06333	80024.341
5902.216	7095.246944	10932.77524	7019.0425	8289.967	6524.592529	7018.8723
6486.805492	7905.5455	8529.078529	7474.7098	9418.847167	10329.32395	8872.057333
7810.938333	11023.61211	10606.86789	10618.71067	10506.33567	9430.353118	11419.385
18006.99971	24222.4692	22291.34667	24590.03361	41157.50414	24466.99889	23883.595
10846.80835	13607.53865	14512.24643	12192.7557	12811.91086	13145.63245	13663.82073
40705.41341	63563.95194	66336.15835	61162.26776	63996.29453	62822.702	63261.41925
47487.3685	64388.80011	65180.14533	64359.95378	64435.56583	62085.14994	61991.51467
11376.26795	15639.05295	13999.36076	13399.76533	14358.66178	13711.78099	14138.06739
128540.2718	156905.9878	151890.624	199435.9198	152616.0124	147630.8618	152760.1357
43003.10393	50483.59407	51303.36857	44072.45861	50198.685	50944.31336	51156.2175
260959.5113	217470.5684	235837.9065	221344.321	219150.6453	251273.6134	317737.8639
18301.25221	25802.32517	21452.47129	20829.78617	21188.80089	19570.49924	20850.54428
22215.84785	22584.96482	23142.76033	23390.37344	22457.61053	22790.93805	21992.38982
455641.2575	624827.1773	622188.5847	657907.3924	634230.2697	625369.665	606631.7104
718707.9605	879490.3086	907261.7593	839234.8735	887942.9378	865858.3903	880637.2614
3526.125586	4122.562067	5418.395063	5028.398222	4810.794312	4729.934786	4928.9228
9081.898583	10410.92795	10583.43433	10930.74928	9200.705	21038.14523	13481.59252
769246.3436	893625.2562	893611.2304	925887.524	930732.913	862717.1635	895235.4341
18931.79012	33860.27071	31703.29	31998.13406	33167.68047	31520.2	39863.541
5521.750162	6208.408086	6232.976677	7036.715762	9560.141235	6884.519053	6279.110294
4195.017008	5333.413858	5439.298455	4861.624632	6235.9812	9759.735488	5245.053563
62027.45259	83072.2807	82695.22594	81500.59794	83268.67441	80574.71629	76806.25538
152165.4866	106561.2647	108422.8682	107029.0052	101662.473	100782.0091	107930.1571
667702.5341	580077.7865	588125.0644	576869.8627	574226.4072	585001.4397	596666.6943
486928.6528	562854.0346	565099.9236	574944.8376	580986.5138	572773.458	559424.4629
1714.82025	1776.363485	1798.583333	1946.544706	1552.62291	1988.946856	2045.278174
129176.5989	1783.3094	131066.4221	152377.0455	1518.15	128350.8209	1447.802444
28474.18433	36954.99432	39435.36726	36663.72374	37993.24784	39017.72064	38059.219
259171.3276	270218.4173	257294.1169	269914.8736	265316.6987	264264.0506	262997.4931
171628.2114	181639.521	175345.7383	197278.5808	191912.3795	183352.1231	187084.691
78353.06112	85650.71532	82940.48524	87844.35706	95660.136	80571.47719	83778.08225
16017.07594	20703.71022	19712.02824	19169.53744	18886.15894	18780.12394	18547.79681
6705.095526	8897.905421	8944.819122	8228.204263	8599.22956	9139.271316	8628.9505
178289.6323	146869.1159	148686.8487	135570.9857	135080.0195	130315.4224	140443.9376
6049.477895	6436.8738	6388.062333	5671.728	6650.721882	6226.2774	6503.245667

37557.09692	29806.06735	40800.2842	42614.15138	40590.6312	53605.59217	37376.90192
4202.360867	5180.367647	5113.234294	4703.900882	5239.978059	4802.715683	4772.25
3957.059448	4881.490087	4515.800667	4715.521426	4615.118667	4314.158955	4490.30213
47279.67589	53237.45658	41717.38615	44595.4028	52586.52533	51406.61714	45027.67481
1518906.597	1436551.62	1404073.88	1431798.752	1513620.316	1439660.733	1497598.92
5696.326167	24583.07291	7697.777105	8412.5176	7194.6285	10411.95237	24914.12636
5217.212	5164.358195	5036.192448	5354.6275	5791.6914	4835.955469	5772.45725
24208.5568	24770.65844	10733.8539	10892.17065	11054.666	12081.97048	11859.20471
2797.243547	3032.539	3265.932571	3413.17	4004.479283	2694.324105	3123.658133
65890.33768	78278.51728	77053.797	75187.43318	80048.19358	77166.1305	80075.527
7394.168294	6354.201929	8364.857313	8288.285294	4581.056727	8190.707059	8826.981529
12298.75933	22496.9139	21598.59189	22624.37758	20412.04941	21848.78274	22917.54228
7675.8776	5719.192375	14285.6095	7464.604834	7281.681258	7760.880922	7331.933531
1015506.76	893044.3444	893909.9646	898388.5606	903594.3397	864270.8635	879109.7904
119338.7893	152195.1341	144110.1728	153654.5439	157297.7954	149351.7856	143603.8139
312193.5384	311127.6553	306745.2243	309410.4001	303144.0383	298699.6849	307108.319
64402.82555	65292.62653	64995.43783	65484.80316	48985.38547	60142.85973	65886.85897
4721.863595	5773.590325	5731.47	6823.406074	7648.437643	5834.350773	6337.139118
19844.887	19567.4178	22566.56843	23213.23266	23696.83321	23260.81112	24318.15533
5343.1361	4275.865385	4374.031365	5292.099375	4492.570692	4829.930734	4408.151109
17117.44385	22770.81344	22112.13785	21192.21267	22648.16157	22011.19904	22217.33568
12277.27681	11596.43559	11094.993	11453.77219	11961.26082	43061.15678	17510.29405
39315.00813	16132.68627	15723.149	16786.0609	18747.63009	15701.94417	17207.808
66049.085	39704.39287	57456.396	59492.10273	39737.6595	59005.59467	39047.53931
39633.26262	55396.47894	56318.004	56595.13959	55218.36106	54680.472	55605.50625
25352.729	38942.87365	36578.49919	39799.75881	35929.64663	37731.86225	41994.82818
917902.8568	1207245.383	1354130.444	1163735.478	1145804.502	1184376.815	1201209.422
24698.02331	72051.075	71719.06563	37354.71394	38032.31006	38503.53044	38464.42969
133466.4045	183389.9734	182468.904	176845.2423	208329.3003	174242.7102	176709.7364
321851.3591	379322.1219	536814.0505	155698.3903	371759.256	396146.5144	387467.0509
63626.05461	65769.70878	62835.88267	61553.39365	89136.81323	61561.22243	61252.14027
7768.727938	8321.300059	7551.331879	8178.7858	7505.497332	7923.595	8119.2606
1105603.003	1598902.965	1571765.226	1544222.718	1552443.826	1484154.19	1580202.115
73712.70939	69945.86167	70112.7425	66111.71487	70763.712	95549.17208	68937.62328
9038.454444	16468.8	14072.07156	16460.17965	15733.35995	19490.4955	16315.63371
7393.87296	8129.7182	7438.4674	12572.62154	6850.48	12529.78956	8092.571143
7020.022217	8600.220351	8678.262512	7906.933158	9056.66992	6021.342643	8555.421184
183445.8313	182081.0297	181109.6901	171109.4867	172189.3688	172515.5451	179482.1513
981478.9934	1161507.023	1272823.263	1182226.584	1227772.585	1211506.108	1144958.68
12148.94882	18681.50933	17475.11415	19805.29807	17618.12885	19447.81614	17147.69246
33181.36142	57378.18316	55664.31479	59625.77395	57872.18855	55829.06984	60450.28889
10425.33357	14923.25626	14266.0416	13976.8574	18353.60198	15321.01292	14599.27424
352093.8267	371239.6166	352988.0231	371751.6513	365573.8899	393011.8912	373607.8786
30050.73846	18286.31653	23016.60392	18153.21074	17420.16178	17216.31768	18497.66535
7368.941176	12051.67365	12376.78076	12596.82667	12137.42	12649.185	12124.45225
426131.0233	700593.0509	726966.528	674507.0135	729027.6128	676915.9679	685449.4145
25257.11715	55271.87437	41360.607	37137.54362	40424.51428	52562.33567	38138.65271
58180.491	68827.75512	71232.59367	73024.23837	70700.42106	68604.382	71628.92522

662897.7294	762703.8708	748537.6436	750211.1168	736196.7245	750039.531	732276.693
2462580.321	3191164.148	3136317.508	3182144.41	3148982.904	1303794.106	3122643.681
127967.1608	102628.782	103430.3078	108927.3732	102967.2292	105957.7417	91927.578
44960.74533	31487.31	40021.36252	43214.73142	31001.86344	38356.52718	33997.629
360978.1676	450834.3286	448647.7519	444530.3057	452624.092	450588.077	457648.1702
359006.0948	387123.8341	362984.8625	380433.4417	287312.3373	280866.3819	281876.6869
31954.58288	41197.96252	39671.93676	39344.50567	37115.23094	37214.67819	49812.87053
147544.8585	96756.61142	152442.902	94100.56217	151342.3469	157022.2742	136368.7482
41706.07467	49108.66947	25313.13414	22267.10621	46857.7013	44269.95434	28009.58927
19391.87335	3936.55275	16607.07333	16003.5995	18817.16344	16758.69679	17721.26005
8818.0258	7665.944482	7141.98663	7472.532252	6177.867279	7072.334985	7648.784871
142967.7218	131279.634	125636.49	164036.5511	127454.5334	126815.0535	165718.7689
26205.62333	38381.92481	37330.27417	31081.92895	37788.55314	32454.72384	32354.42746
4532.662588	4466.210435	3954.16325	3736.346643	4330.842188	3992.764125	3948.13125
7701.240625	10208.737	10375.89512	10518.42035	10444.00188	10574.84241	10707.51518
22536.696	26583.28863	25360.43444	42155.07537	41288.35121	26495.56295	26996.2899
57139.19374	78660.64052	76502.59478	78656.865	83342.11385	76339.91895	82338.75744
71446.31511	98097.83348	96603.28889	93423.90321	93916.559	97680.708	96062.98086
54583.25237	57668.65442	58546.1542	59882.30335	58152.86305	59061.9351	60237.82245
11893.97496	17141.41705	18743.8865	17199.5665	16182.20756	14547.69829	16595.7916
43257.86335	54088.36019	74714.125	69836.92017	69742.974	70594.98836	72373.7385
6982.408286	15239.196	25227.93432	25764.69288	10855.12989	25636.52596	26437.49388
35888.20114	46112.80148	46140.582	47001.0515	48177.41745	44621.97857	46018.38781
165685.4469	191733.4831	201000.3963	195674.4918	201499.3959	191677.2812	197906.9585
33191.38848	28108.27264	30519.56432	30793.13508	30905.91992	30093.56513	29298.72642
24690.55924	31994.65417	31414.91418	33058.75822	33493.21717	35478.25959	30256.49722
34989.60931	27631.57504	13013.41112	22846.00136	27805.48881	23389.80907	27114.04
10826.48808	13781.95557	19637.1454	13581.418	13173.04091	12770.38706	13608.98692
474676.2239	792700.4746	794831.3624	787710.2355	803169.5749	808126.8459	803988.2712
15257.16981	21902.64956	22154.2552	22235.213	23564.69147	21258.00167	21281.29667
17981.34656	23458.79692	23109.42085	23112.70971	17753.32517	20878.70164	20917.85736
2848743.394	3862239.426	5848251.27	3862567.476	3732472.192	3833898.331	5788395.017
1524636.631	1869470.86	1837780.511	1812085.398	1815821.208	1828065.4	1838994.539
39065.89333	25180.95762	42975.6225	41527.97447	44132.92458	68453.73533	43964.8054
21199.07789	24489.76619	21241.80608	22744.84693	21724.55647	24765.82696	22154.44488
159841.7341	198157.5697	200190.8504	186136.5969	195144.995	191201.1238	201892.9995
356716.2121	492318.859	479592.9386	508708.2199	467114.82	488512.7083	483854.5763
7731.389388	9958.143724	9418.915158	10002.29135	8997.6539	9277.397714	10244.97642
789636.3907	954042.8448	937110.6574	942842.4217	946875.5816	883564.0578	930802.6345
2725.002429	2752.747143	4722.834071	3112.042286	4964.172968	2481.509308	4916.95125
10338.5695	14695.19283	17434.2584	14793.35258	14228.12167	13959.8547	19156.94486
4017.614725	6287.334	6418.580438	8335.297111	6245.65125	6434.76541	6081.210103
91928.67628	111409.6978	161458.5897	161788.6729	110679.7937	119262.6865	118957.4282
321469.8552	480093.4866	226043.1634	476875.7042	269721.1387	229061.1581	221554.8195
23530.20062	30749.07631	30485.57241	30902.73862	35942.8041	33050.5551	29976.11976
70222.39101	108165.0751	36841.2705	120189.6835	107699.7503	34942.37681	37308.51731
182216.575	275550.9463	263465.519	255651.0927	280345.1855	250589.1319	251123.9187
172781.9796	149244.5409	216064.9021	199446.1078	109319.1332	91162.29808	211750.5695

6028.243713	7718.698867	8303.218991	8889.167368	8453.755325	8195.661179	8042.36334
23299.185	34275.39071	34755.65855	35097.16044	42506.48822	35398.19994	26691.795
6917.221834	27279.72353	11992.56947	10822.78798	27706.64042	10511.38348	24614.93154
17917.9752	24585.85694	37939.55516	25820.01535	26287.05865	50872.25208	25842.89717
76469.78194	104592.7695	98213.59206	105277.7924	118329.2684	105033.8683	102413.7648
129905.9383	101612.8077	338491.7608	96387.73136	184269.6486	173188.3014	171716.3133
92042.37616	125042.9462	125791.4249	127312.6785	129292.1227	120755.0947	141209.8186
169535.1324	252018.1031	215832.1006	161999.8849	208713.2271	213425.5199	207652.2382
890336.1072	866186.2509	886222.988	881445.3741	856420.5414	876047.57	842119.3414
241673.7878	230994.5507	219934.2656	220928.8958	234685.3507	213854.6306	219655.0575
521135.4477	678486.8234	679744.9338	668348.7169	689668.4075	680449.142	683896.9311
56333.79918	67726.632	75261.35644	65276.7725	67220.26084	61751.73794	73601.678
5899.09928	6547.608095	7517.37804	6375.481	7047.037141	7404.008423	7183.10448
329855.1369	393218.9528	411567.4294	410472.0524	416619.7991	423041.6957	385241.1084
1022501.788	1348916.301	1344083.009	1339958.404	1327693.623	1385729.122	1354768.129
506937.0849	632380.9736	625173.6898	606969.1395	636127.129	636948.4295	627812.8619
634969.5409	800106.0722	784592.0749	922198.0845	836976.7581	966784.3995	907232.7373
117716.5175	132391.9816	143878.8054	128463.06	141574.957	134596.1035	134690.24
9233.1	9103.766077	9901.256154	8138.1465	9350.34	9327.629812	8539.570667
1959.30375	4643.690754	3210.256298	4797.557387	4315.819395	2977.001923	1934.497154
1778873.743	2102487.804	2014485.889	2021766.517	2040977.297	2075404.907	2026885.376
13052.8944	14036.7344	12326.76639	15358.91645	13489.74626	12035.73512	13753.28339
136758.0059	170079.3389	178550.111	166083.1226	173595.9558	161821.8935	168919.8584
92977.17016	56533.92464	52750.42589	134275.6086	129748.6835	58309.45138	55396.502
87984.382	44371.554	43361.1736	42097.90667	43913.87967	43874.287	43065.97307
4681.599701	5340.987	3697.845667	5132.727612	4954.919294	5359.815527	7399.58816
1384.24975	1279.90725	1731.668138	1474.138312	1142.609863	1457.541962	1740.875455
116484.5842	151145.1036	151889.4802	155469.5805	155086.7605	154253.1135	153772.6571
10989.155	6495.24575	20238.51263	5787.12275	6934.3782	6520.5625	6046.73475
94868.66982	116263.7728	115221.3683	115650.663	115852.3744	121040.5268	116329.1525
54454.60594	35385.57969	58850.47433	31924.37588	37691.863	37909.33472	35151.10565
121150.1005	186130.5126	188250.7033	185256.9351	189824.8306	185586.5553	186939.9822
3072.927	3127.134563	3291.685733	3772.179529	3123.368867	3542.911611	3427.78575
257797.9391	358570.6822	351010.1531	388529.3271	369363.8403	369689.5606	387056.7882
9783.822533	9030.468607	11589.75256	6372.9	9377.205933	4249.902857	7652.481522
23351.65042	27963.96375	26028.3294	35556.04203	32956.74812	26559.1194	28380.51681
14743.52918	27365.104	18590.19327	18381.24058	22401.89354	18687.26659	24807.44314
20885.38639	28690.49179	29252.35784	27831.22863	28933.93189	28821.45011	28646.514
11007.78312	9766.425	12329.62875	11498.381	13310.58289	13936.31933	15258.85958
348957.7208	462236.0722	476333.6828	468035.8779	472005.2325	470262.3194	465723.3235
3359195.254	4576294.89	4618973.282	4501118.218	4578339.057	4525336.443	4559801.028
112009.4607	112762.1389	112378.4825	106802.8712	171131.2177	101210.7923	111598.0362
12486.95094	14900.128	13629.07038	11983.70152	12542.32135	11449.776	13778.79835
7179.665039	9776.065926	8957.947957	9515.56795	8695.9342	7521.942118	11207.563
3212594.262	3906878.747	3946997.083	3977248.352	4125683.394	4084765.45	3948293.174
510698.8435	685960.7082	671788.7386	617690.8477	652734.6731	649184.183	662877.1922
18.46140081	14699.62	9206.826366	11877.37778	18765.20281	25453.4425	16618.26366
714076.6654	720280.4385	707857.007	720864.2606	733110.8636	711465.5317	707879.0216

3407.993824	5443.4232	5985.007471	5266.058067	5531.078176	6421.1065	5975.474385
6962012.707	9014206.445	8925562.964	8814580.348	9007121.894	8988611.549	8667282.405
227321.7284	254297.8447	242991.3979	272524.7209	272085.2535	1088626.4	259119.4426
1327338.583	1623430.968	1583837.896	1610888.725	1638908.8	1600085.156	1588303.378
6364.480467	9097.353474	25586.47139	7485.389821	7912.516426	8616.391389	7584.590812
9937.408	13240.91728	12600.72506	14033.824	18411.40906	13874.9625	13049.6135
3259.279467	5976.9144	4937.5095	5266.024176	5141.773333	4904.716706	4493.169462
7897.697868	9557.874667	9828.124111	10840.26667	10213.91556	12061.25083	10714.27
540955.7479	667098.9675	650336.9128	663522.0981	684147.6277	660188.2455	649703.9458
804221.5099	1062593.815	1030247.778	1035537.989	1011274.327	1041309.104	1056213.153
511299.5963	681806.5551	684153.8126	676292.2359	682160.5914	703255.553	649293.4133
7196.063	12461.98388	12279.53018	12303.09763	11619.95794	12477.15535	12583.83706
8035.023304	10471.89125	10664.02935	11704.91126	11573.26036	9637.71655	12882.704
6357.555471	8374.209143	8697.7605	8074.856054	7928.301333	7708.7826	9128.616611
28386.29132	47092.69689	49111.94489	49060.2043	48627.01137	47555.26779	49867.34568
6458.073588	8670.472717	8470.964772	7961.399418	9523.978722	8628.744353	8404.94875
106773.4695	126036.0916	108545.4918	124561.4902	124224.1558	120877.5667	121854.7002
96730.87293	485380.4914	468964.6658	477633.4203	490725.1586	473590.5204	469239.477
3059.875882	2960.203625	2908.08675	3208.571438	3010.232222	3207.107647	2965.622437
11308.08166	10597.08028	9991.422733	12768.42343	12863.06	12706.14744	10472.96435
6114.146316	11363.62058	11307.354	11813.8932	11153.97956	11503.296	11073.01842
338319.193	374176.9152	374639.3957	368107.7835	368699.8786	370773.8197	369162.0434
80384.45175	118349.2118	124172.7052	122170.257	118277.2854	122875.7584	123963.4168
32627.39325	16749.8134	16667.96845	17229.8965	14455.1295	65464.422	14197.50853
182374.0644	184061.469	179889.3821	239299.5086	183547.6592	171717.9012	180351.0803
49271.40549	60434.913	62953.98853	58832.58216	59254.5305	58103.7132	56725.779
3556.018588	4159.237471	4259.326	4248.475471	3770.144438	4105.873235	4136.517
5693.426618	7695.5236	7299.282117	9011.147168	6363.631833	7264.021977	7618.062692
13136.51647	20275.8603	21798.73356	52579.48165	22299.13902	20023.66126	14521.51093
11309.04571	17835.30514	17491.06167	15497.33744	17057.13379	21377.21352	15132.43125
1399.485	4346.226449	3328.096727	2575.35	3315.466713	3489.654582	4218.58623
141563.6139	207573.2437	205994.451	206692.1974	204578.818	205211.1573	207672.3066
395003.8856	519215.511	517543.3212	520871.8887	515171.7912	528341.0709	519590.1071
19667.11811	28118.98757	26591.87523	26526.15136	26092.20728	27915.29824	27625.83374
37296.002	53072.08583	62013.10832	64624.7614	65032.90462	61680.8148	64107.88042
60183.47762	56859.86	59201.70986	56521.13469	67374.46636	54685.7326	57127.21433
1224382.455	1298489.779	1297272.019	1819785.853	1818883.329	1270989.259	1828033.634
30205.18433	31822.7865	27522.43829	29348.39977	29485.12182	26796.10085	29013.80615
184746.2182	179270.6903	184987.9813	178005.9412	183329.6323	186444.353	181349.918
28217.03383	41822.80123	20474.26	53104.50294	21496.40186	20748.40963	21697.22186
20911.31106	27784.60905	27757.84733	27385.25533	26871.5265	26595.55344	27146.75116
13973.475	11629.61054	12018.89931	14574.3516	11128.30608	10988.664	10969.04333
14638.88837	19085.67931	18220.00071	18148.1256	17732.41733	18380.71537	17786.75129
7856478.822	11613609.01	11603124.15	11909349.57	11854723.46	11696218.63	11522295.53
153085.3005	40710.782	36580.22373	18564.84	39452.8959	39845.61809	43735.27154
3262349.233	4837283.265	4855781.885	4953264.957	4883297.989	4925474.637	4727033.58
50086.13495	41768.83761	38346.21806	38619.54518	41489.79689	38086.01419	40432.66359
10175333.24	12932406.36	12683397.24	13265990.2	12926478.05	12634666.07	12885071.07

2093.364	1603.4397	4423.499321	1541.3175	1774.5385	4110.375791	4419.079722
814.6183333	2192.6	1973.15	1794.7985	1604.571111	1641.6	2576.214735
10766.5412	10722.74	9649.435923	14543.32575	28484.96573	28540.78542	12910.80923
192991.7606	120883.9909	122159.6898	120053.2246	115155.9406	117494.6877	113105.997
346253.2602	480005.2066	473039.3483	487267.5373	486016.1809	473613.1776	473709.771
44712.90353	56840.0955	59554.73833	57879.899	55557.11433	55295.23595	55520.35353
25409.66615	24286.27789	23894.32356	18978.51284	26016.31827	22880.883	24633.24229
975663.0434	1124598.402	1141065.712	1136636.372	1203733.916	1131811.172	1139431.841
127331.3275	172378.3478	161473.3147	133035.3899	152420.6068	162965.1784	167973.3498
11880.79238	9976.895118	10970.16537	10115.50905	7963.12	9164.674738	10920.9905
190490.3	236369.4531	227188.6625	285916.431	499212.4994	224715.6577	233425.9448
352239.9551	498420.6501	492659.4223	497283.3266	501333.807	509164.4198	494387.4772
92451.856	109581.2285	107651.1774	98775.63963	104676.9978	103810.2	104552.8817
15868.79385	11385.27553	11181.75	11865.97588	11121.72553	10603.35962	10749.09318
1700.286917	1547.437941	1583.079	1814.667235	1603.506	1834.50075	1638.628429
1500.0392	1836.075882	1753.16885	1922.787706	2205.523765	2109.495167	1896.235
176573.9224	238634.708	246460.4581	246867.8617	246001.134	239391.6185	237043.5919
16205.73565	15050.54855	17509.38	16129.10847	17259.67253	18355.54182	17057.46788
32057.34227	38168.88	34357.86	40962.75177	42032.04149	37144.8	37996.23333
126317.5882	145965.9387	141617.9963	147248.388	148375.6524	149913.6158	145715.5472
3618.54552	3584.506167	6213.039938	7699.505308	3609.468333	7265.612721	3759.381
26714.26863	20787.654	34560.79367	20647.3855	21044.492	30328.83533	32954.94207
21303.1329	25905.68133	26112.95489	25514.12763	28216.91553	27623.78378	25614.928
283889.1984	423684.4736	649835.5083	690755.9937	405271.7635	407990.9413	404231.3873
21572.484	19000.05315	17993.88675	19261.93555	17684.60132	17781.6561	17750.15568
205859.1835	272767.1903	255665.1751	258130.7064	259226.7737	263145.4787	260467.8581
75875.01783	60064.95388	77515.83113	77538.2944	78806.41777	62286.02488	79733.02458
82162.53622	78824.04765	80517.83628	82551.31379	83490.05077	79100.69538	79142.96331
4277.027903	7056.50087	8702.007455	5861.051444	6035.359	6210.396111	6760.312333
4551.489	7378.92	8258.381312	7400.991429	7737.037	7692.663214	8963.031
11201.55692	9886.654385	12277.27538	11061.95469	13849.65261	13302.73238	11255.54571
516238.8469	607896.982	573354.5443	592437.8084	582616.6385	635213.9173	587993.088
455085.9045	653485.4394	649367.6959	652365.3814	681561.5968	620974.282	670949.5074
89666.00028	83595.69995	92813.97944	85306.38126	84476.581	83599.38883	84493.30022
5794.028118	18567.15465	17603.53861	18118.0476	19250.56443	19488.62081	4616.377429
1578.276357	897.2166667	1017	1483.04	945.6741429	1247.555556	1697.3765
9479.892353	12019.13942	10468.23525	10339.46667	11099.01333	10984.70809	10798.77623
48913.39807	42776.86808	43620.73336	49869.147	43780.09089	45771.18	43739.129
29180.928	49637.60015	48770.31612	48710.83102	36906.618	37112.97483	47215.00785
282501.7567	428441.2045	426420.4953	454622.8784	436052.7686	428204.3893	422970.688
326815.92	441179.8237	450491.6191	436610.5815	464555.2791	460686.985	442938.3533
497909.4505	442892.647	426220.5431	422283.7596	411131.587	397570.2875	424446.2263
1208.861389	2140.502975	2749.601	1630.949261	1859.21	2266.046	2470.961511
10112.9808	13702.39339	14920.6064	12916.80253	12986.91159	13346.45403	13277.11171
111050.364	151684.198	175228.45	155250.0073	180107.9646	181890.7665	178594.8892
3016.674735	2965.419143	4270.715267	3334.593345	4001.027467	3341.456072	3901.149118
53617.68024	49268.4144	91612.41231	44467.85406	89930.73167	46364.01405	45248.52663
28441.53325	34801.56071	32431.28211	32470.28885	31380.97882	33217.23552	34426.60988

48171.56813	79471.29033	74747.51773	84266.28526	85127.81363	83443.33074	11879.2905
735570.2727	980922.8246	1348034.395	971889.8205	981903.2616	954933.7261	1337566.696
79978.3039	73268.517	61964.08941	59200.11029	59605.73124	55304.29129	58629.14611
110269.4182	133397.4479	127730.8611	127378.5576	131267.1541	136411.2874	132517.962
6692388.821	7840702.512	7709707.863	7628479.447	7687669.633	7660810.35	7817118.721
1072582.578	1576641.073	1563163.697	1562903.005	1570478.239	1553454.939	1578133.11
15015.4002	9440.436552	6449.506	17184.92467	17012.12651	16353.93639	16761.46519
21166.91718	26308.78617	23962.59888	11462.90667	25670.65041	24856.27258	12868.10314
12795.5463	15000.15013	15180.405	16733.50818	14047.80729	16231.1846	17694.604
22461.16747	36390.75	30726.28069	36399.95527	35140.85713	39600.19369	37878.07
7306.1585	16022.14242	8343.125083	7534.299333	16644.33247	14528.94697	8108.698857
74427.892	56572.82537	58284.34568	58369.6976	57033.3779	57566.8301	56476.35325
52021.01344	65015.145	64948.41336	67032.43667	66246.51443	78314.66994	66148.75872
5542.307105	5552.859375	1448.5365	4451.0775	4471.211818	28072.45093	5768.570133
2641216.079	3953035.441	3990655.648	3914466.302	3863265.644	3927632.99	3827485.168
10990.79773	14374.152	11150.32664	12911.96356	12548.45269	12727.25703	13195.48805
888509.854	853454.217	845380.8721	853700.3864	878892.5149	822174.4555	836590.8292
6588.790684	12448.59891	12421.45624	12859.47111	7885.457167	10895.37544	20285.94082
3541926.223	4561546.099	4490266.54	5308064.405	4417036.584	4311796.121	4398320.082
14903.25567	12561.06271	14227.337	12305.47594	11851.00544	13031.20824	18820.76933
5316.599167	9084.677059	9926.977148	7423.847118	8411.8655	6938.6646	9804.764357
100002.5693	96403.30356	98145.71532	94434.82967	97118.79137	92289.12311	93443.50422
503864.7064	507751.6601	458984.444	497547.6495	516526.688	449153.1301	454206.8855
2766299.979	3768028.904	3757640.874	3692762.99	3771804.959	3704702.133	3676286.116
29638.224	56172.74682	57750.05253	58374.38222	55699.51565	55183.42394	60196.12444
65592.2628	68649.0843	64174.1883	72536.85746	69383.5862	68254.55265	68970.98057
50167.53947	52861.02324	58285.15875	50432.04926	57059.52352	57445.75225	48778.88516
100225.9083	421694.9063	426435.3912	408727.2816	431460.6106	414484.0727	412899.3202
403550.1239	471338.9323	458843.8725	470198.5752	479964.5986	472390.3096	493864.904
60829.99179	73228.23009	74391.93973	77269.90113	77914.507	84762.116	85168.69221
3814.264235	5816.369062	6357.356625	6077.37375	5763.581267	5569.364535	5329.010547
65575.79325	217488.8438	218590.9887	211247.7752	273611.3591	208025.8809	222580.1393
426658.9815	506491.4941	767876.1047	496952.1068	527171.0254	461954.8195	478238.8378
17966.8385	62249.14	51019.52143	55562.18387	63534.12537	48378.72518	46234.58985
2214923.905	3288275.678	3306554.778	3348143.01	3297225.314	3325463.18	3597486.717
1037136.601	1528346.266	1513931.928	1500094.881	1518697.754	1480113.091	1507819.347
115786.878	152081.076	141111.1934	141147.11	157223.0051	271281.1789	142004.0971
8764.114429	10102.50516	22955.79275	21153.13748	22768.7354	11220.56	11111.234
103044.1428	123988.8545	125182.7613	126494.2616	121937.9165	88223.09143	129946.96
195192.6773	289852.4669	287433.3348	280195.407	290681.5353	274584.1806	284606.5197
63830.19886	93900.035	99475.423	96739.22886	95156.57905	93271.4772	91439.9343
40590.34255	61241.20167	65902.83818	61001.15269	67292.547	67593.88893	64945.95785
12346.11546	9344.827606	6394.526158	12403.82832	8672.081451	7282.535	15035.28413
60132.9908	78148.88805	82879.26508	39874.34962	82587.04836	76880.07581	79714.25574
4118.999214	8052.036937	8468.426667	13617.04129	14291.99931	9051.562688	9635.807813
10664.46395	14378.09349	49269.57726	44846.02812	14484.41439	14032.22876	13261.15778
106362.9768	167093.3954	165448.9824	170957.6051	170213.6543	168474.2421	161248.3248
7360.863854	4166.019	4933.614913	6915.313083	5219.859625	8219.44524	10995.44354

46088.4905	62516.05082	59298.57247	93261.43269	66578.2325	59657.85025	60925.12324
113611.0768	128510.8092	128914.5631	115258.9042	125842.0442	134350.0512	122965.0782
10840.58059	9265.485783	12083.13257	8627.121087	12776.16261	11139.561	12497.95276
329689.766	352179.468	356153.1089	363228.3458	369510.876	357103.8417	358688.2203
3607.573278	3349.106063	5479.424678	2793.221091	3466.8862	3550.947	3625.19025
147804.1992	220602.3877	198991.0864	195417.6215	198334.9557	190869.1175	193494.3434
26789.78296	26826.39887	28707.04759	27731.50646	27500.35425	29901.62667	26544.53054
35908.91879	47599.82065	38567.433	42445.1516	48791.98633	46138.11917	50818.55447
226801.6158	255539.0593	263415.8983	263099.8895	264707.45	259065.6681	258815.5821
911783.004	1260008.586	1224619.366	1234724.571	1252217.573	1206150.159	1239515.925
116808.2949	136408.7342	138614.0435	128848.1348	135773.1785	136546.6862	149632.174
248838.7102	265763.218	274025.8881	267060.3282	266351.6841	264394.3285	266767.381
5507694.893	6093549.045	6078745.465	6043107.818	6073091.796	7013983.37	5886049.404
13961.34236	25006.40323	657057.0961	25790.33196	21770.93134	24397.42416	23562.30649
82892.24418	82380.09106	85824.86738	86730.95294	81447.45213	80393.26969	82723.59544
147274.7052	191269.5114	195054.2444	195040.1202	197323.5734	202401.6211	186574.2455
5562737.309	8055314.495	8169942.189	7608091.79	7658486.138	7689879.929	7639204.47
3028299.158	3466184.562	3415039.987	3434194.421	3366836.687	3440501.823	3490387.848
259069.5012	342191.9974	386540.4545	375600.5684	361337.5314	367822.7262	352498.1754
2568484.017	3608804.709	3719472.033	3412081.6	3403363.659	3403015.777	3439387.039
371935.4675	419771.3385	440571.4221	435884.7913	427190.2972	423657.1723	433427.9387
808708.0177	1104326.113	1057777.559	1081922.09	1076987.106	1079403.612	1081184.123
1302768.964	1539652.932	3052840.181	3083735.496	1542113.127	1549442.203	1513845.702
1007480.104	1311926.332	1257186.265	1265599.597	1320692.768	1310327.354	1286736.877
92185.7601	238500.8546	233723.5967	158303.3338	241540.9089	158069.53	157943.6666
217331.205	223314.7718	222131.9828	225223.754	230122.1653	225843.8678	219233.2995
515229.3917	709034.1316	706181.6456	724621.9788	689046.255	706807.8509	587958.7134
19648.91309	29010.62737	27246.78113	31307.82459	37026.61329	32550.11719	32282.05059
580914.3708	677730.1778	678522.921	661838.3239	692887.7314	685106.0025	676551.606
490967.6488	711422.3649	698259.0724	700999.754	667902.8453	710986.6186	722776.9594
33803.8965	56026.63778	55381.51218	59472.44188	56078.47262	57648.09924	58756.25247
16397.93631	38040.02088	35859.95076	45745.40145	43389.12339	37151.69689	43268.06614
5609326.83	7433845.442	7436936.112	7600059.412	7503210.924	7347694.339	8321147.241
491331.6539	696621.8091	723103.2597	712317.9377	710927.1086	653085.3986	687784.7794
30125.7046	57349.39907	55498.01081	55141.6392	53991.40047	54773.0832	54412.09267
43731.23194	70421.43812	126825.0628	71853.106	70290.714	82442.95976	82391.0395
5723.513461	5732.591615	4277.56075	6727.563121	4575.5967	4898.088462	6770.809593
334694.7054	417852.3071	483278.0307	434551.246	446268.2948	416393.0761	425322.035
136866.5649	249977.2142	247927.2566	239576.3029	229883.3154	242391.3814	238398.7282
75688.26262	89840.93115	91476.59365	100524.7174	94602.016	90427.48521	102843.7383
430135.5555	237072.7543	215313.2864	212485.5404	213700.8682	217241.8338	224539.7631
61700.74032	73359.09368	72693.48558	73302.48867	74278.16875	73494.30395	72155.7177
40085.58713	62346.74113	63644.90009	60870.248	63362.48961	62031.48295	58756.23786
206716.7404	246800.4109	180519.2295	180420.8323	181407.3551	175213.6578	168281.4294
168171.9272	227046.9985	176662.7924	182695.5034	175456.1883	168346.4822	224042.5146
60808.3476	75063.0435	74869.7722	73716.5172	83457.85522	73332.04571	77951.60862
6017.4762	6201.832125	6148.004069	6435.9132	6244.842636	5994.883057	5727.87323
46508.6448	69806.41545	64764.072	69478.60048	63847.23617	65193.70583	64593.1915

46812.91495	51109.9032	54959.11812	55169.12571	50289.1627	53092.66253	54307.27419
16078.27289	19655.37558	18152.46125	18197.39232	18018.20735	18837.25195	17890.87335
12202.46729	16341.1139	14459.76043	15571.10732	16119.02632	16337.85	14419.59206
403933.8329	824534.3267	819133.7849	801454.3232	834729.9916	790741.0746	802450.7768
8831.173267	31974.46	18733.31331	13486.6814	15831.56988	12426.23164	22456.21413
11289.77506	15226.6181	15112.81514	12212.102	14770.75672	15700.01467	6205.035
8918.778588	13233.7498	13540.34841	13512.89944	13727.35	13533.65363	22653.55417
50555.12133	90464.9322	89817.92989	89365.7479	92253.49258	88170.95916	91641.9153
2712385.817	3078043.435	3085375.996	3071686.22	3083145.286	3005881.921	3082669.982
66889.75297	57575.94461	52499.41989	56421.03567	53629.744	55589.79505	52266.1194
6240.771444	13080.29931	13058.1	12687.88828	13028.27963	12987.67763	12290.51791
8394.332843	11490.66309	12325.86905	13733.81413	11480.79414	12675.57544	11910.13846
15911.45265	22463.43809	23009.48496	23187.22664	23826.44691	23481.50001	23541.61228
29783.05771	38226.04617	29916.46641	29155.25922	31495.63684	31648.46453	28757.64488
91711.5966	129568.7805	135514.9113	145777.928	144331.8479	130995.6542	131286.1951
96769.43383	114188.8287	115623.3716	111610.8402	110733.2951	109394.8501	114087.6329
1270530.973	1631438.222	1630927.766	1622561.351	1814963.567	1621857.284	1635258.188
7367.629883	14624.05212	12570.6	14166.50278	20083.3397	13215.042	16566.72215
37621.41129	42602.7918	43662.4253	44803.80817	45443.63581	55268.88307	45186.33562
4725610.156	6529748.754	6511808.818	6412669.728	6534595.71	6414353.185	6560438.412
139126.5234	155361.8703	194272.3933	180365.6853	176551.0807	173358.206	162928.7683
13965.08971	14706.60718	158552.972	16034.55273	16936.95013	15268.85435	15632.97358
7010.090063	20025.45125	19232.74828	19685.18806	20496.73806	19396.0494	17601.99646
609495.7819	856171.92	888057.584	868002.268	860045.9309	838173.336	855618.7209
2758669.833	3814330.973	3697857.27	3608449.621	3697007.064	3730856.212	3615482.122
15026.7741	23931.47574	24449.52741	25757.2448	32747.75497	27008.35183	25484.17841
26333.71711	30514.79	31377.88544	30675.08694	33590.352	46638.291	31785.90155
4098.709995	5844.144457	6523.8988	6738.732	4119.201125	6877.36575	6906.547939
1259884.254	1698742.188	1692291.029	2364127.611	1698532.917	1715559.096	1675363.405
370347.69	458711.2382	464166.3499	440081.0102	454659.0761	458132.8704	455945.9203
392923.1934	490933.0742	484158.8971	490721.7911	502476.033	496346.7874	495225.1959
11641.12147	24508.42212	24767.05518	24799.68729	25669.77788	24219.27159	24334.86594
124877.9101	157226.1996	154427.4064	158838.6874	160745.2143	166164.1788	159932.2759
202856.8653	253172.2967	241673.4429	246730.0979	236949.0317	233335.6451	239243.7356
60052.49394	99199.82544	98567.742	98302.92418	101288.9394	100221.5912	98112.963
63679.469	128607.0253	127305.7489	128401.1728	129771.2243	126308.9939	127296.765
11710.5189	20759.88998	20214.36517	20081.60427	20723.74306	21311.40765	19270.18824
249190.2826	630736.7586	631028.4467	476885.3573	475673.5854	481696.6675	641650.3005
6938.7435	12884.28406	13880.27025	13771.16141	12450.00325	14657.2148	12255.99338
110220.0852	127712.3032	129071.1789	145472.368	131465.2238	126494.3115	128474.1917
2721.865405	4209.342846	3882.267818	3033.507921	3505.364111	2682.2328	2078.9076
395818.4928	712258.4932	687945.669	675930.9709	670759.2574	713379.325	682009.0303
113285.8506	158013.3347	157584.3159	152291.7918	160040.1493	160780.1417	149833.5894
134579.2417	142545.712	143847.5156	145875.7425	149786.9506	143355.9951	145988.6438
10212.18979	17047.92313	16194.10669	17553.51638	17264.98525	18247.64525	14962.04267
24496.9938	30501.29211	31878.39129	28836.848	34014.76092	31581.4926	29953.86616
32451.78518	38033.05881	43978.71712	39194.87133	41824.08276	41254.81329	36365.89336
144141.3908	184278.0106	186850.0891	175509.4412	196201.4707	195110.8326	179545.6283

25000.57924	40816.72755	31573.53685	32852.67234	33626.35425	33025.47126	31890.48104
75548.69628	100036.8685	107084.768	91995.28217	112208.7967	97764.416	98484.0435
74365.10338	46380.80967	45439.457	66750.65196	76180.14494	75991.0588	48628.5561
15094.98047	10453.98635	16079.75713	16500.36494	24818.79357	15445.2958	20834.6908
3689.808951	4021.280064	7950.590143	5977.841226	3249.770667	4521.956727	2413.324833
19952.67747	35388.51356	34321.56588	33656.64824	40435.63911	32954.80244	34086.31819
542984.5375	515695.0725	652920.7591	588991.9386	593207.9301	567149.6647	634292.7724
9020.60781	15509.26143	15869.78305	14815.0163	16391.60957	15425.48638	16715.30615
700782.8469	791239.6191	793706.4715	767245.629	790575.9881	789860.5979	792113.9644
6297.34376	8960.06925	9984.486696	9035.08425	10217.28215	9907.658308	8729.959778
374422.4984	398356.1001	390093.1703	396176.8865	393248.7701	402038.8947	391841.2711
305062.3325	212388.7523	230394.8323	226461.8816	312799.885	228592.7088	325116.7913
3901.67	8847.036551	8675.758	9246.508	11194.9488	10866.95018	9338.119337
27784.17176	23349.14016	29391.39697	25968.25435	25736.20088	37420.1893	29306.97979
6006.570938	21046.18338	17462.156	16736.1645	16489.13744	16925.06424	35428.71098
163757.6846	208127.44	203510.7256	209318.2813	213195.4083	209200.7531	214996.7056
19927.389	26408.4704	22362.63829	22273.04044	22819.43897	25555.68086	27355.45947
29435.211	40124.37371	34821.98215	35407.63538	36602.82793	36965.40093	32643.76209
9511.058571	26717.346	26679.98036	27522.75353	28704.27933	49202.74982	27403.06486
8196.039778	9887.7828	9695.463892	5968.753	5890.545	8870.202643	8523.5688
9901.29364	11217.75652	14833.46585	12706.54233	12742.8965	13559.68293	15242.86662
35215.94347	78365.20588	75791.69412	77419.19941	75757.13435	77578.84206	75181.61188
12237.61244	21405.578	21138.21956	21229.01993	20955.38867	21389.23207	20962.88186
183924	332724.0152	330701.4506	343725.9012	344306.9949	331833.9239	331535.2241
31081.73559	63001.21471	51237.80606	47425.56082	45916.70699	47641.97312	50003.30325
133740.2653	211525.7904	202190.3072	201484.9982	197618.454	201330.4746	206456.9896
125150.7742	111097.4352	107044.8175	105354.0567	111017.5486	110235.6098	116734.959
5707.9275	10054.55182	8728.946333	10119.68025	9610.01	9484.221063	8957.435143
5454.7388	15267.66305	15213.87605	15386.88391	14112.67453	14599.89456	15653.58704
316379.5712	352284.0644	346677.3081	195316.1325	348001.5289	190285.293	188320.0053
3191.679612	5378.254615	4944.26	5442.714935	7590.432913	5436.518423	6104.787937
4667.381128	3697.730444	2951.9936	3057.993	4140.90387	2232.149667	2972.009
5107.708474	10913.63074	12138.28647	11192.08585	11312.66374	11457.24	12244.27237
369253.5868	369036.0931	373133.4988	393134.427	368386.6477	406502.7277	360911.0981
55816.79635	62288.83584	61262.84129	55731.94486	64914.03848	60901.7353	55637.23291
18613.94282	19791.372	21921.09657	28819.35663	24336.37688	35623.2008	21980.51778
15717.87394	21272.63765	22219.3123	19432.341	19791.861	23264.401	19365.669
815462.5749	1069828.219	1050460.569	1080923.289	1041210.803	1039622.508	1091952.617
26415.09004	28480.40742	28756.03383	29179.63	30434.69744	29337.3024	32310.198
15110.41024	48915.83023	29844.05944	28924.3096	29429.3664	28940.42667	49252.24549
32904.58749	30873.38523	35516.88246	33817.98394	31354.74519	30583.00805	33418.89103
6400.745357	17873.1036	19662.48505	20298.34653	17912.91433	17254.99635	18867.44258
9170.048159	5003.627	4550.83225	7028.580364	11293.69966	8463.129055	10553.04009
21948.402	23595.47845	21921.39569	22940.18533	23531.61381	21401.58183	24008.45088
3798.825333	7299.597118	10246.24075	8802.661875	9766.63502	6982.813786	8914.076097
8801.104889	15692.60129	14474.06276	14866.54406	10748.9068	14761.10869	14525.30294
6031.860667	9266.957471	7363.46	9989.571889	8899.398375	8801.614062	10149.0285
2205.116821	2223.565884	2455.091754	2561.065526	2292.768	2460.299	2572.375105

59030.97309	60018.47371	60912.79063	60457.49379	59594.41684	61639.86706	59205.11082
35555.83768	38945.95857	41256.12948	34700.745	43578.26467	41760.04165	41815.28758
4646.016842	5092.61025	4346.124941	4276.193211	4382.349262	4197.82313	4726.231211
2544.850542	2932.168617	4976.882195	2615.210254	2941.364071	2856.372063	2696.220596
21037.23318	20870.12688	21130.34869	22212.44831	21368.07871	21040.70094	21255.19988
5768.481931	4673.007	3889.9315	4048.936833	4684.659368	4838.656211	4090.518684

QC7	QC8
5567.286154	5658.7195
21447.961	20441.90444
1164988.303	1189879.3
10794012.46	11474246.51
7962.324578	7336.468333
281837.3869	289569.4877
11025828.61	10690333.94
6452.078571	6111.889333
47517.74892	47691.675
208504.3052	205370.1636
128936.8551	140194.1717
42622.86737	39109.89231
198920.3851	197301.4673
642801.4004	632226.2539
51514.3225	51715.93143
11800.30514	12947.56295
125040.3909	144217.4152
577466.6162	577640.65
87193.17984	73352.33813
26812.15167	51592.69918
350402.5287	341148.0378
23541.63017	26339.511
475792.6563	500400.6
408410.7354	409399.7197
1618.7653	1665.019909
41420.34359	43848.59811
51872.03467	47548.51877
36739.22292	29612.50105
157522.2564	154624.5891
220677.3422	230157.8727
748762.8104	749755.9008
8685.676095	10542.03811
19398.13594	22724.70673
40064.65754	37817.334
375447.8406	405273.6808
5215.769813	5989.857391
1024904.655	982164.4869
8804.749286	9175.190571
20193.9122	7735.363636
12514.57481	11682.49813
46627.12575	39554.45314
18809.163	18590.99529
392446.4431	445919.0691
233271.8307	221133.9727
236404.7498	232200.0856
14972.58943	10949.23636

11795.95259	11090.14664
12556.4634	12562.9456
9913.744312	8661.610071
21059.32285	22098.11775
184867.9473	186260.6512
6084.083857	6275.578703
27824.8241	32443.10618
60460.88346	74743.11491
4292.903357	4382.9808
16363.75385	3817.935
13205.12402	4067.453
2624.553727	2388.986286
9313.060706	9378.425634
30479.48547	27318.50013
6969.708941	6956.931052
1105047.348	1082709.611
1055434.21	1109866.269
434546.6754	437968.8075
43403.10567	42466.1468
11793.9745	13077.72859
8082.093762	8050.853254
1624.538462	2082.853333
49821.34733	21902.49367
30792.72193	29934.16208
227547.2818	232996.2639
128773.4871	125594.0605
285993.2139	293989.7578
27731.4849	17299.61301
194602.176	187440.8469
310151.7314	500426.6667
322074.0532	307958.9643
231960.2028	203467.5858
2580.8695	7220.601667
41286.4767	43391.80926
1308486.9	1410097.935
17578.98123	18400.0845
6839.409	9151.212176
10336726.94	10179614.58
4545877.895	4642979.23
100537.8289	101954.4961
76141.72725	128411.6155
236247.8792	224471.8857
61057.29	133187.0578
340172.0809	342643.8715
368199.521	389244.6432
34858.97799	31271.66979
55589.88831	52768.46575
177180.7122	170628.8973

411810.861	415690.6338
41580.8905	38202.11636
122484.3183	70338.45087
344793.2071	204621.3216
78814.17	79719.40136
515182.5547	540981.0855
720015.9376	720131.0668
91261.22636	91417.64757
25185.28031	26937.85088
675889.0962	683272.1568
2670653.426	2595716.92
12884.65685	9648.469
6741634.278	6757208.967
116662.7598	106993.0033
6912443.403	6940208.615
132211.1047	127626.3399
8036.200889	7371.2972
3900242.497	3709188.538
10114.02728	11169.65349
347877.2437	331250.2941
1551887.36	1497617.944
370596.2823	360503.7785
179489.6473	188660.7141
37058.46693	36566.92927
325409.8132	233536.0458
13445.49308	13920.8436
6724990.647	6406400.4
2041.97536	4046.867
54513.46333	33358.52861
509155.532	508081.2217
139746.9753	141712.6233
123215.3819	106368.4409
9834.3045	10115.33358
299895.3792	292496.83
53679.21067	52401.65378
4160.037636	18453.39375
2815336.003	2765062.252
9305.518333	7456.427727
8728773.289	8768121.994
2197856.386	2042260.539
3669027.458	2377743.659
59732.38717	60036.43216
48870.88521	64116.58897
26748.5587	22530.202
1353882.789	1471910.728
95321.18931	127530.6071
32932.29553	32628.33329
46350.42536	41585.45166

144098.8646	148464.163
44659.43579	18350.76559
5004.426	3893.857571
61687.647	64393.20062
462646.0238	434485.2541
30753.80853	34119.51865
59162.82883	58259.51733
341463.8309	317330.1662
417748.1147	427169.5423
95356.43756	101839.0165
160923.3685	175495.6205
13519.76001	12210.645
28683.80021	28092.95
62003.19196	61201.12702
171074.3862	164662.4881
12104.41125	12177.0076
186924.9947	179144.7969
210927.4784	301295.9128
15522.62333	16626.41325
437651.4013	434768.4105
22019.745	19753.40182
71345.45587	66323.93795
26928.01078	23815.44647
21566.22662	24117.7875
62808.101	35595.39394
477826.1467	499136.722
56780.71185	12788.571
35491.89695	32553.64865
3636854.763	3593681.643
5265.736	7535.517527
8767.260554	6286.532571
4882329.432	4872241.478
1319549.025	1352810.252
8608656.444	7069471.015
385666.149	472777.8253
108498.503	102374.7083
417008.8599	429056.5474
1012313.75	1032537.018
6568.205	7178.065173
13010.3373	11551.16556
22503.19077	15092.78571
154507.5378	140038.8778
139034.4008	124800.1714
222589.2336	233579.2493
185113.4551	214076.0661
89806.43755	101221.6847
176120.2844	176981.7027
41986.019	43800.53379

1065157.059	1057413.198
176751.1099	178104.2589
145865.9414	136362.0563
8808.514673	9934.706616
65017.5708	59069.4468
27483.18819	28685.93714
17453.61966	15305.01237
14223.0675	14451.45907
26385.02808	25376.27274
54476.25921	61483.87
155332.062	165404.4497
124629.1282	128791.9366
31427.17964	28483.84048
4241380.081	4154805.823
4624.549231	5755.061769
1053074.572	948742.4948
266102.046	292319.9808
381423.3084	378326.4315
997004.727	577605.7009
194960.0887	186987.2892
45477.504	37536.38211
2766.34	2889.04
55334.78286	55676.57778
12611.67048	12968.51943
79745.6275	86624.9885
279057.0393	108870.0032
268795.5098	262914.3426
507030.0486	514188.12
1445935.373	1417604.906
945851.7169	913601.9395
3194.18708	2991.523438
261733.0513	255523.9849
1831190.752	1831790.465
65356.99975	63999
2531849.549	2717165.887
125006.4772	144941.4191
40576.05085	42002.80754
275403.9138	140923.4518
230148.1097	175725.7181
2225199.114	2215606.422
64111.60751	59717.5034
49988.39514	50814.85357
38578.68575	44838.21011
296766.1175	294005.88
465200.7913	457163.775
26216.55064	26029.51709
1418689.87	1154579.061
180117.3818	306483.7859

51958.12431	51715.48292
1924994.404	1921242.556
388972.6226	388647.8666
1097073.017	1108344.887
31029393.93	31628119.82
640422.3178	640549.751
1274952.4	949012.368
45537.51354	38472.77225
145791.6814	143233.9217
2103530.447	2091113.358
31786.20667	56126.85052
14565.91003	18264.11497
506297.8564	503202.3775
494198.8291	516483.0485
226466.4151	227351.8571
89454.57509	92012.6589
439420.2304	461288.5366
72681.93588	67707.1805
2732033.945	1586649.563
4178745.384	4107220.268
4029354.364	4013146.403
21192677.68	21285843.25
291276.7517	166934.63
198473.8275	202977.9868
64105.2804	64309.22017
19679.22727	29390.41154
4499581.567	4589275.842
1058258.823	965720.4654
246483.5589	229616.3224
4893.976135	4473.736369
8543287.246	8759143.391
25279760.49	25475862.59
133241.2064	139460.379
273776.8051	265428.8624
274153.3884	274777.5271
15200.66966	14454.59111
89478.55099	5989
575027.8821	591066.9793
94319.50408	76892.11725
54289.56273	46842.76333
1277016.317	1195025.067
25238.7425	26415.85344
10482054.18	10092575.77
5860504.903	5442419.968
176718.7675	185495.9345
19200.29162	31162.69447
1748233.567	2236079.488
7465938.853	7133673.008

103829.4128	118030.7308
7313763.873	7060856.248
1012001.855	968011.7768
2250836.547	2312949.577
13619.47315	12597.01457
361398.0049	365036.5263
1034.490909	2100.195269
1059116.107	1225668.551
18931.97141	19863.37949
16718434.58	16763766.89
1135397.415	1176742.944
14309.28827	13534.77222
80410.82161	77184.68116
4368923.379	4410070.307
5770505.409	5829603.59
1339.265686	202.7304348
730531.9507	696797.289
13882079.58	13406799.77
793690.642	798916.9153
297232.1343	292985.8188
822867.6671	775395.2876
782803.1935	785167.8103
13443597.24	13501261.6
38993.22488	34947.27566
439157.5925	556698.4873
421287.2983	449351.4976
0	3612.875786
164074.5622	154781.871
111892.4462	119697.1882
2424.713949	2416.46342
215851.5373	215753.8074
252133.416	252753.4423
10732803.11	10735124.01
567679.2713	572738.1944
191878.32	191177.7658
12831256.03	13207256.3
44054.10706	45077.64935
407682.9482	399277.3983
693.3108346	515.4435948
10551448.19	10766430.55
611441.7029	616716.3602
10558010.18	10752901.66
135579.1574	135519.2419
59142.9388	92845.30498
458557.3525	460041.5744
124682.4136	235932.8544
2334630.248	2391535.596
25882644.49	27045073.47

2263730.121	2525003.017
343977.3592	463608.1687
91867.31782	105417.1551
49596.98776	348.7611643
678129.4224	679740.2097
514217.2482	205403.5217
5549912.102	5540211.875
211399.6173	202243.3562
778439.663	774102.1321
674343.4585	673182.1506
514777.528	493747.5088
33434.66893	26736.525
1585815.196	1557440.671
72016.72154	71371.14843
38121.48147	36463.74356
1065125.097	996188.0216
47548.32373	35737.76137
3185741.414	3295000.767
107601.0239	113951.5955
3432.644836	3445.044034
334275.7282	331256.2914
8894818.445	9295908.239
886.6593876	2507.9284
1776425.13	1771535.332
16712.26495	14490.69506
739009.5556	724796.1575
336779.9212	334769.5709
107879.289	90293.82886
1501548.269	1502119.587
400249.6663	406298.6068
5129099.922	541708.0986
6440.623901	6787.297252
2104854.58	2189557.125
16310174.69	15923793.25
3358031.227	3343305.767
827034.1709	808933.9365
3781.367182	4105.08
1135812.622	1127278.31
12893667.41	12574525.53
536094.6872	512502.9307
698.8638212	4031.169556
999245.2078	977149.8741
1374117.791	1265488.209
694783.44	826346.4011
732.7092983	628.0410243
6789834.479	6888653.194
369236.4944	366500.1749
20405.59863	24111.06346

3768000.425	3740403.363
109520.9959	113833.8267
6918597.429	7087410.706
101875.6374	70338.05847
59436.26877	58681.63893
9793506.696	9811295.82
82838.67933	101813.0363
2322187.23	1063005.912
276143.4192	276476.963
85221.04063	84235.19791
70164.063	68438.18571
104951.298	107913.5355
85892.04943	87209.21708
37593.47046	39010.21362
3809098.691	3761165.705
202527.8723	274161.0804
62235.22314	61386.02658
317613.3827	229129.1539
388838.7515	390608.9171
539337.8729	507808.4241
84771.96975	85516.72478
103312.9506	98734.9985
597264.1026	610658.3563
54891.48141	54535.36108
66057.80508	63331.26751
82240.17233	84318.20433
48601.94529	55980.66667
212.042333	46.78661601
22061.09553	10140.81361
390218.545	396417.397
2854365.869	2981533.085
3440837.843	3236488.78
226748.1022	242878.4711
13087.27641	14488.74641
51014.70536	20065.88981
16489.45375	18356.06393
733126.7481	717118.893
334201.77	325181.8776
663408.4537	636689.0907
281052.3996	298188.1489
3253.018658	3288.084623
2074620.989	2077074.036
16607.0016	13684.82922
110653.9796	109041.5936
160629.7187	61525.72818
46632.58713	46515.01139
8800167.596	8475868.92
190490.443	307350.9252

91761.69156	93515.07422
81732.79232	80064.91437
201146.4639	203304.6985
97736.38809	99524.84806
39319.57564	38054.28223
38507658.23	37985119.19
630552.1201	624674.9882
60928.52191	60566.18273
104769.0858	141429.5454
136087.995	132719.9658
570772.7815	557045.7016
98901.60182	100620.9013
296173.7748	275307.1431
76698.45892	73503.71836
128990.592	124229.1065
236997.8837	16664.96667
26143.95313	24902.80658
4475220.735	4420304.95
92892.3034	87082.15064
75941.79119	77671.81981
380585.5616	414347.6334
145233.5817	175824.4288
14863.82333	16720.13432
15704.42052	16746.57637
12978.09857	12847.46538
107255.0046	110762.854
20495.28756	21446.90791
43436.04415	42019.13578
59676.04575	56672.58458
236690.3289	436930.4623
8506.617541	8512.304086
44265.38836	42633.38357
8207.784	10611.61763
10847.89378	13223.8224
167769.0927	137249.6314
17466.04571	12367.2808
23860.7435	20703.46666
23445457.06	24717547.13
203500.6548	163834.7702
163318.5745	137029.9077
7875.63	11048.895
251888.2716	275410.556
33806.53775	36367.11367
150288.0549	149846.6339
497797.3307	483712.5384
81212.11866	77404.2334
365341.3032	354683.2005
62885.10002	70652.92033

25760.43964	14954.52
381305.7475	178034.5494
7367.052	21642.01494
16212.27858	5911.85
18889.34483	20914.73682
75192.89187	73670.57058
88734.02244	89512.46538
247302.5431	243674.7858
212714.6667	213936.2337
75482.9263	75442.77953
460895.7271	454882.6998
226618.1941	236279.9003
55773.8192	34476.25739
28439.95907	14061.7198
504635.1082	506373.4186
4873.355625	4172.848
47352.0672	50705.05924
2813.537	4548.897727
67147.177	94240.7562
325100.5341	119373.2327
2125.341634	2411.38237
75008.91568	75613.83168
24111.8865	28947.78343
35019973.57	34498202.98
528248.477	569452.983
400147.7706	375742.095
309356.1337	319616.6092
27381.20463	25761.50783
256302.8346	238324.5584
8899.711048	7154.0469
180768.9939	192602.3242
28751.664	70115.68567
29347.81342	29965.72007
212591.7349	223246.5954
369347.3683	379942.511
48483.34864	53243.07953
55202.17227	86464.87451
84780.89714	89849.983
359809.4831	345355.5694
1924.896606	1895.308527
45575.35556	118255.443
58351.71083	58009.64924
140959.8816	385008.4058
41013642.07	40344398.44
378672.2552	349213.3841
19788.72788	20356.6989
883680.8341	931131.3903
19650859.14	18964851.34

280167.9901	290435.986
450870.1392	417921.4315
731536.7735	771441.2216
201185.2233	187126.2392
304379.8157	302879.2772
393807.7019	392845.9774
758567.0712	750760.4901
41833.6842	248219.952
338353.1917	351657.8142
82537.6662	78502.94498
10747.67693	7847.022273
226637.4964	217983.0575
322918.5872	449038.6806
97544.62567	180572.5781
340343.7019	338311.2342
84362.95894	34379.68921
107956.4414	44204.27264
8069.118222	4833.959821
50565.83438	34742.06737
289763.9294	230619.7774
26526.45415	12126.17456
16116.92727	19113.78219
71143.26847	98044.836
30187.60157	32176.88735
48588.49933	48144.0648
41926.4925	41671.07619
4724.479667	4696.324286
475317.6828	495848.4351
10888.728	11026.92088
199495.4494	197342.6563
126776.1729	130030.9868
19348.674	18903.66317
25007.11074	31889.78186
11221.9016	11305.4858
234047.4926	242415.9484
256161.2662	253487.0383
500221.687	507924.3146
6972.200545	6968.65825
14916.25642	17495.17056
97492.48429	92062.13671
41732.12135	47100.51065
8509.2786	8789.864
34427.61629	31506.45977
55305.54276	57314.27895
18992.69594	25625.25491
41724.0985	40739.43933
5549.285053	5724.433056
3219.3866	3135.455714

770305.9503	773435.1652
36992.87317	33931.898
142674.1712	143035.8482
1724.8985	1846.818
82677.18517	83269.817
1385257.199	1385304.678
8680.758235	8575.170947
47153.15641	49560.90939
6528.1675	37344.6738
25828.90565	34681.53282
19540.0219	18919.69871
25472.91929	25619.19813
1042495.311	1070895.081
939650.5481	914809.8352
8879.825133	9722.962563
113194.208	97838.51129
27916.63467	23995.38995
52019.922	48887.96438
54178.55806	72344.25629
3341.831111	2598.591095
34212.36688	34162.18906
5312.397563	5361.882059
207359.1826	212545.4958
15656.32294	13836.41043
6916.771273	6173.426889
6470.406	6851.1795
42988.93056	41594.27738
8656.36	9165.96617
624.9707201	955.8641667
7682.410714	8071.767294
6736.65216	5770.581158
7544.7534	6371.7738
4614.915111	4784.512765
9793.508789	11054.39308
34051.46082	35891.09333
1222.773333	1469.720777
57279.53044	62465.421
8222.342529	7141.775833
63714.56559	64478.68517
4345.776	4287.494444
40159.31638	63994.21249
15255.79719	15719.30678
8749.6606	9760.239667
8756.71335	8527.056018
8624.196824	8842.579059
27910.38214	27766.32171
43801.94478	44174.14447
4492.7974	4212.578063

2401.066059	2402.527072
69175.89459	61476.52206
63411.80232	74793.191
37468.93812	39763.555
7935.793333	7282.346302
349801.1034	360626.8905
7931.86975	7683.04406
30595.72125	29834.65575
34502.1852	45986.78861
10897.78568	11659.549
6264.266773	6513.412782
153761.7068	162999.3138
80468.63406	83379.66389
6434.458211	7105.383333
8840.110278	7652.271929
10242.92145	10090.24022
23805.27658	24360.38778
12846.46818	14609.42038
65074.94862	64189.79271
65328.68059	64579.78333
15409.57371	14364.77769
163426.5835	153530.5479
54018.815	50906.085
212273.9977	217770.718
26997.62089	19855.00353
22242.19367	27203.28275
652808.3314	617476.9431
882184.4437	880182.4483
5366.725342	4578.125154
10632.5621	11294.61474
915844.8347	895800.2185
32671.93511	44878.558
11024.83295	6901.0395
9459.536286	4184.772706
78185.63325	81187.18212
108542.9303	107539.5739
601799.4587	580414.2466
558206.1825	577837.5181
2243.537247	1629.295288
134524.8707	134901.0651
40756.32369	40490.648
262760.1768	283651.3258
187125.3551	252090.8645
80721.50588	82044.91805
17963.14375	18841.74886
7953.062256	8433.688421
130400.2548	147089.5283
7941.09425	7247.618368

40619.81531	42348.91734
4657.957125	4066.812667
4476.21946	4978.345982
52340.97471	53132.97856
1422629.398	1430413.208
8227.776889	23995.46345
6009.8665	5728.992588
11878.98636	23112.58244
3486.682908	3122.043915
73717.05517	75945.89429
8658.00075	7102.799
21950.928	21989.71744
6534.470558	7286.541067
886276.0236	914075.2934
147045.1346	146205.76
306391.3449	311183.5875
68984.65613	61722.46513
6233.0258	6660.747429
22630.46906	22578.98631
4737.333462	4647.74845
22610.4375	22721.99563
11071.0758	16988.482
16335.6005	18227.31863
60071.81345	33830.0985
54003.99462	53206.08356
39685.381	37691.76471
1241762.52	1154034.887
37637.09475	71312.03
213458.2583	179574.4266
151315.0757	394430.5171
64804.03344	61636.32509
8470.3682	7782.049333
1547267.793	1566507.087
101185.2335	69740.68292
16451.84295	16478.986
7738.353571	8191.1932
8098.22245	8587.765478
180176.8593	176722.4336
1161117.876	1159442.805
43507.29773	16289.612
59291.05811	59523.79795
14626.81545	14772.21586
382249.9821	381228.7309
17605.5698	25373.94424
12466.924	12529.90954
780087.3283	687407.6449
38279.03088	28588.95714
70535.46383	67024.54283

761580.2942	774628.2565
1291441.66	1365060.31
90955.6384	102963.861
34837.41056	38097.93886
445642.1856	449504.5609
274952.5078	280042.3087
36891.02435	38930.993
93711.22712	148403.1463
32381.03865	38045.93984
18357.401	16324.88
8039.348959	9535.717714
128809.681	127092.3425
35785.67457	31744.36988
4011.022312	4286.597824
11654.78783	10356.02663
25290.41642	42270.75372
78026.179	81596.00073
90372.15855	172054.4345
60641.31684	58945.65348
16013.56575	16916.6592
72482.86385	72036.28571
15948.7358	13285.50043
46920.08475	48836.52859
192837.4179	198412.6994
30185.44533	27941.4904
32850.66411	31876.43565
26510.80523	28655.7885
13204.22732	20323.79524
802214.361	785368.2941
21192.48153	23401.31694
23562.345	22776.36
3851824.951	5932137.054
1896305.985	1843280.432
65367.21643	66244.86636
38331.61313	36504.24487
206315.186	205959.1791
505350.0541	477306.0658
9512.8	9206.276308
942815.9386	929033.7443
2783.172583	4835.4724
16897.75657	15275.73821
6583.422333	5988.7175
138255.8261	111217.9746
222166.18	223729.4659
32243.72324	30856.79776
37657.74771	109840.0749
260862.6374	255347.5473
142239.405	220750.5185

7710.204353	9065.628947
33981.9355	35906.961
9747.674077	11758.91326
30558.6234	32351.75194
102568.1955	102572.5099
336253.7158	107971.92
122862.1366	126504.3859
228085.968	221071.0455
888599.5264	869634.3619
273543.735	218908.001
684712.5169	686733.4565
68450.367	66331.55928
10898.34405	7178.71797
392741.6762	402735.681
1337420.566	1362585.532
638181.7464	632940.7012
800800.4266	809140.4791
132345.6745	131213.2749
8556.254182	9506.195529
2391.097813	3039.00959
2041526.372	2109508.495
29334.30886	29262.61097
175337.2131	176674.5867
130325.2938	58344.746
42661.4958	44072.97
5202.421155	5409.344076
1296.537813	1438.010688
154138.0474	155999.9297
22260.0125	6160.840833
118557.598	114518.1538
56690.64533	55218.47121
191502.779	184523.78
3813.682167	3568.852389
364380.4472	360105.2098
7317.473706	8338.886579
27137.7049	26805.50968
18692.09419	26185.38273
28736.28044	29371.24611
13999.755	15932.65649
472100.3265	478139.2
4445163.844	4695646.487
106291.9021	107886.4477
11593.4285	12756.00569
12497.08181	10137.52893
3931879.386	4098303.493
659769.2718	646937.6947
15025.10322	15777.73
721458.7239	716961.1645

4531.5688	72844.74596
8633350.065	8817778.087
279190.4375	257748.4588
1606753.206	1636631.071
10193.27295	7256.972667
16077.49316	18246.89709
4759.186875	4712.956462
9732.864556	8740.813125
653355.5327	661988.0419
999783.9212	1021361.286
674062.7356	659783.0663
13441.27982	12384.61824
11891.67695	11274.652
8185.759054	9029.241333
48359.47137	48813.87615
9397.507141	9578.981053
117726.4718	129325.6277
474817.2728	488334.2866
3221.084235	2940.127313
9476.649267	10242.64194
24675.9805	11285.76805
368778.1917	370198.3574
123056.7602	125631.2002
15279.53524	15760.21708
176988.1844	180316.9859
59774.48143	62973.5814
4027.4325	4509.327882
8763.527035	9738.356148
49463.36425	20285.12186
15284.53231	16868.09645
3854.614318	3959.049316
205672.2708	206237.7051
519549.1163	509776.1298
28639.19113	17881.71413
68597.45536	55818.44883
58812.63245	57464.51747
1854109.175	1820779.934
28438.781	30814.41385
183201.9452	177950.9235
50040.17144	14395.27762
26479.28337	27795.28455
12038.52908	11485.13385
18393.48113	17818.06694
11907912.21	11455643.77
41302.5325	39929.69175
4982586.294	4845367.474
38539.56441	37543.47919
13179159.57	12963560.97

1647.18625	1618.972727
2129.65	1468.9595
19718.55627	11437.54435
114720.5752	119325.2782
477333.4292	499812.6884
53746.53394	57141.67222
21828.668	23075.32521
1153363.45	1174584.885
160615.2095	162951.9073
10693.88957	9604.111404
272640.3503	236407.6728
488456.3788	499157.1545
109494.5365	105142.0663
13142.24359	11681.00727
1771.484813	1797.629611
1967.654059	2590.679233
240007.5705	244595.9128
17679.68041	16352.93813
41992.06	44204.65008
143771.7057	148444.008
3458.220111	3619.251195
36868.98918	20167.38043
23824.28794	28599.3183
669184.2839	680160.2432
17342.10152	18377.89405
248857.9067	272006.6324
74248.257	106072.025
75957.67536	79397.75458
6328.213816	9194.604273
6891.093	7711.05975
12889.09213	13389.07999
590098.7742	594574.4304
663121.3693	650517.89
86869.38884	84841.57306
18989.94456	17777.1667
1243.905	5952.908203
11209.077	11081.75708
40570.26469	51555.08012
36390.53817	36565.96643
422799.2071	431124.8407
457294.1708	458211.6619
436907.0706	425445.4588
2100.076007	2852.984077
13318.80392	13538.80297
165622.5411	111762.4344
3293.003967	3446.806955
93678.4384	94955.34337
31232.77165	33218.18633

85356.44452	83776.03181
1354152.041	971447.0586
76562.356	56972.1285
130762.4503	130450.2549
7707880.4	7680641.727
1593533.552	1595874.878
5525.64	16885.01087
24904.73613	25201.48922
14044.23162	14956.7715
37730.55531	35706.9375
10801.83879	15862.51209
53051.28183	59350.718
66686.90756	67263.27376
5450.1555	7275.5795
3989935.553	3988489.791
14701.243	12862.36394
849479.7335	855878.6595
7757.240111	13028.99606
4934145.759	4460986.656
12158.28941	12988.14235
7914.4105	7921.725
92132.30983	96950.87867
450514.4204	459927.031
3688108.001	3757822.434
55135.88753	54860.25176
67712.55943	65778.67805
53071.34625	47446.465
561770.9198	409912.5893
467587.5868	462874.2443
86679.98824	81567.41184
6390.070062	5884.100062
287595.4379	224883.1198
463154.5512	534632.8781
56493.25413	62197.8512
3775635.458	3298410.555
1701406.456	1525077.262
155051.9068	151358.0815
11576.21108	7752.6054
115577.7936	130819.8995
275465.0236	289633.6282
93186.01005	96722.37705
63650.85085	63816.41538
8333.593333	12860.58075
77697.93826	76579.18526
9275.61375	14302.4416
50568.56721	14365.23176
168184.3122	167389.8072
5837.720167	4517.765455

59647.968	62149.53835
134898.0477	131194.448
13450.18606	13214.60907
352267.601	362621.8112
5575.114607	3467.173389
194229.409	192012.0842
30225.74142	25665.61774
41202.28853	48237.49567
262038.6189	257527.0004
1233581.233	1236342.004
118476.3368	142820.272
264553.1855	272880.7783
6153328.115	5965188.653
29337.1165	24131.54928
81215.38941	101750.5172
187968.3722	184018.79
7908007.941	7905246.74
3438084.945	3411255.507
360778.5298	350305.2834
3553291.487	3445114.799
435397.3913	430975.6938
1111616.209	1087160.823
1582118.543	1554404.167
1301403.716	1307299.279
158407.5034	237046.7584
217158.9304	236772.9675
733295.0247	602773.7895
27627.2775	28268.11837
681452.6653	681807.4767
641414.6705	605287.0504
60014.49441	57311.55318
37034.73929	45105.08
7448453.275	7590212.854
719443.296	655223.3042
54465.3382	57109.7478
67922.263	83665.939
5994.174683	6120.801053
432151.0327	437991.7938
247286.881	239816.9039
92343.0647	89244.909
227236.3581	223048.1342
74435.23455	73526.4144
60673.95595	57881.901
254253.9146	168397.2928
173148.1632	157781.4323
74798.04981	72965.77607
6319.675267	6111.607617
63616.74594	60061.04741

50654.16038	54489.23365
17830.57235	18754.53826
13017.64912	14911.50163
812303.156	793344.9076
13887.0603	32426.61182
14981.83726	14686.73979
12642.5304	13012.31981
92797.541	90333.144
3039691.374	3026596.495
56238.54764	64009.10005
12934.49426	12097.4935
11899.42615	11803.64008
23176.01411	23392.90844
36062.97294	32722.7025
127429.9518	135633.2233
114644.7578	116168.6664
1647995.439	1607336.178
19888.55032	15623.66667
43800.8634	42056.8896
6510551.484	6563296.956
174030.624	172032.4048
17596.381	16385.58648
16914.86601	19412.77144
857270.6924	845318.3849
3650788.105	3747857.521
31746.56845	29598.03745
32593.94045	33755.37952
5953.710547	4970.04585
1700715.01	1715556.474
460725.7101	459455.5441
495214.6685	499178.3385
23644.61888	24647.21535
159517.779	152998.3532
242340.7887	249772.2164
96558.12918	100539.8894
128289.5597	132558.0516
21704.308	20468.4177
468916.1374	617837.3708
12590.95153	14416.64775
130126.0653	131369.0201
2504.678	2037.8078
734472.6694	687896.8436
151048.8216	153870.9089
144863.9037	146106.1583
18478.38438	15511.32613
30585.84458	40744.85288
41820.16	36350.63922
187182.5277	191696.5625

33224.04804	40431.78858
108678.3578	87731.15655
74197.60194	71446.06759
16059.51514	27092.84994
7218.233	6366.57425
43284.64327	35157.16031
542611.8062	665370.0424
15726.30927	15184.9914
764101.9561	778821.2518
9707.662286	9503.5134
396168.2821	392519.6471
305169.2268	300658.3271
7966.086	11562.74933
25700.99113	28646.62757
21565.56738	20082.15544
211332.5597	212011.1746
22951.434	22495.17713
36820.3528	38154.64703
27824.4502	48251.83871
9231.676138	9103.808818
11408.01867	12448.07193
76446.83469	78764.00194
21146.27533	22060.31439
330687.9609	339842.4854
49725.68606	49230.56137
217053.7781	195712.7587
109037.4349	115239.5494
11104.07082	8829.578003
14868.1291	14677.31925
172636.0032	342724.2618
5874.112209	6795.785706
4072.847023	2434.447842
12073.78963	11315.76016
423617.6261	364462.7897
65540.9104	58884.53048
24164.95294	22272.80344
21299.15794	21194.7515
1039848.931	1048201.101
27048.61391	31063.02238
48742.06659	48821.5952
36192.56076	37109.06022
18385.541	19447.68737
10938.29806	4748.388
23597.27484	23036.68524
8251.44752	6895.58013
15931.12535	13795.03676
9614.290125	9190.263529
2297.747833	2336.061078

64812.5028	61924.69
36455.63585	36003.59169
4791.523263	4535.881053
3180.090353	2667.662155
22105.99556	21262.182
4270.0595	4099.095
