# Supplementary material for: Replacement of Dietary Fishmeal with Clostridium autoethanogenum Protein on Lipidomics and Lipid Metabolism in Muscle of Pearl Gentian Grouper
Source: Aquac Nutr. 2023 Jun 30;2023:6723677. doi: 10.1155/2023/6723677 (PMC10328730; doi:10.1155/2023/6723677)
Supplement: Supplementary 8 — Dierential lipid molecules in the muscle of pearl gentian grouper in three groups. [file 6723677.f8.pdf]

Table S7 Differential lipid molecules in muscle of pearl gentian grouper in three groups

name	Class	MainIon	mz	rt	VIP	p.value	FDR	CAP-01
PC(18:3/2(	PC	PC+H	802.5536	384.772	4.94748	0.000511	0.060203	21618097.9
PC(22:6/1'	PC	PC+HCOC	862.5537	366.8635	1.99006	0.000511	0.060203	3727351.02
SM(d19:0/	SM	SM+HCO(	867.6562	490.661	2.916323	0.000511	0.060203	4759921.964
PC(16:1/2:	PC	PC+H	804.5664	392.5115	3.770653	0.000627	0.060203	16676988.2
PC(O-20:2	PC(O)	PC(O)+H	782.5953	425.354	1.424994	0.000761	0.060203	1106347.931
PC(O-22:2	PC(O)	PC(O)+H	826.6695	535.048	1.913733	0.000761	0.060203	2171952.821
PC(P-22:0,	PC(P)	PC(P)+H	690.5271	405.184	1.473421	0.000912	0.061556	3970102.95
PC(O-16:2	PC(O)	PC(O)+HC	806.5298	343.972	1.061639	0.000944	0.061556	1893226.819
SM(d18:0/	SM	SM+HCO(	853.6398	474.352	1.181693	0.001139	0.064902	1011049.986
PC(O-20:2	PC(O)	PC(O)+H	844.6367	529.703	1.420669	0.001464	0.064902	1936234.089
SM(d14:1/	SM	SM+H	785.6359	476.2555	7.489962	0.001675	0.06898	104387290.7
PC(8:0/26:	PC	PC+H	758.5828	460.412	2.266245	0.001996	0.06898	11054561.56
SM(d15:2/	SM	SM+HCO(	785.5788	389.923	1.518456	0.002117	0.06898	2828321.254
DG(16:0/1	DG	DG+NH4	598.5303	502.149	1.219316	0.002552	0.074381	2164945.36
DG(17:2/2	DG	DG+NH4	670.5268	456.893	1.52559	0.002706	0.074381	5829033.525
PC(O-20:2	PC(O)	PC(O)+H	792.6073	488.9755	1.276729	0.002754	0.074381	3208485.049
PI(18:0/18	PI	PI-H	863.5586	358.0965	1.034639	0.002754	0.074381	2658541.089
PE(22:6/1(	PE	PE+H	764.5375	444.595	1.120296	0.002937	0.074381	2043524.04
PE(18:2/1{	PE	PE-H	740.5207	408.273	1.126383	0.003006	0.074381	3320585.789
SM(d14:0/	SM	SM+H	677.545	334.686	1.294841	0.003225	0.074381	2314037.72
PE(24:4/1<	PE	PE+H	768.5346	441.993	2.544033	0.003321	0.074381	26392498.87
PC(22:5/1'	PC	PC+H	818.5544	333.3655	3.084124	0.00334	0.074381	8482638.146
PC(O-22:2	PC(O)	PC(O)+H	784.6356	516.467	1.324121	0.003379	0.074381	3533055.248
SM(d21:2/	SM	SM+Na	869.6356	619.69	1.105806	0.003379	0.074381	2048599.164
SM(d16:1/	SM	SM+HCO(	829.6412	477.5855	1.901897	0.0035	0.074381	7196489.012
PE(22:6/2:	PE	PE-H	836.5371	385.481	1.093254	0.00458	0.075512	2461203.003
PE(O-16:2	PE(O)	PE(O)+Na	770.4961	405.161	1.375913	0.004828	0.077527	1516193.987
PC(18:0/1'	PC	PC+H	776.5976	436.277	1.487836	0.005089	0.078606	2171559.336
PE(P-16:0,	PE(P)	PE(P)+H	688.512	445.476	1.153463	0.006136	0.082854	3194033.441
PC(P-22:0,	PC(P)	PC(P)+H	812.6513	513.82	1.184651	0.006136	0.082854	4286722.548
PI(18:1/16	PI	PI-H	835.53	368.887	1.685078	0.006136	0.082854	9781855.556
PI(18:0/22	PI	PI-H	911.5602	363.365	1.052319	0.006245	0.082854	3400578.296
PE(22:6/2:	PE	PE-H	834.5263	368.7655	2.581769	0.006281	0.082854	23773655.61
PE(22:2/1:	PE	PE+H	716.5066	400.8035	1.562191	0.007708	0.095512	12069705.89
SM(d16:2/	SM	SM+H	811.654	513.834	1.578164	0.007844	0.095512	10309161.96
Sph(d22:0	Sph	Sph+H	358.3726	149.259	1.704309	0.008125	0.097119	5544721.301
PC(O-18:2	PC(O)	PC(O)+H	714.5321	345.929	1.084982	0.008317	0.097119	1910479.883
SM(d14:0/	SM	SM+HCO(	831.6558	521.3105	1.324	0.008317	0.097119	4854111.567
PI(18:1/22	PI	PI-H	907.5306	302.3065	1.170414	0.009683	0.105179	4614656.102
SM(d17:2/	SM	SM+H	791.6045	488.9755	2.522567	0.010632	0.113516	17109661.44
DG(18:0/1	DG	DG+NH4	638.5594	527.93	3.495159	0.010757	0.113516	39232407.42
TG(18:3/2	TG	TG+NH4	938.7384	619.957	1.994006	0.010948	0.114304	5012487.139
PC(13:0/2(	PC	PC+H	742.5278	331.635	1.606718	0.011539	0.115186	3336901.614
PE(16:1/1{	PE	PE-H	714.5051	400.447	1.11101	0.012022	0.115253	5235167.22
DG(16:0/1	DG	DG+NH4	614.5738	537.578	2.274948	0.012378	0.115253	897939.1269
Cer(d18:1/	Cer	Cer+H	620.5844	526.979	1.03845	0.012378	0.115253	3013058.672

PI(18:0/20	PI	PI-H	883.5308	326.8	1.266002	0.012378	0.115253	5855233.098
PI(18:0/20	PI	PI-H	885.5465	360.6905	1.71478	0.013124	0.118646	11982245.72
PI(18:0/22	PI	PI+NH4	928.5684	347.389	1.575717	0.013752	0.121642	10440908.58
TG(17:1/2(	TG	TG+NH4	906.7509	696.872	1.237824	0.014752	0.123828	633802.8854
PC(2:0/29:	PC	PC+H	720.5594	421.826	1.573952	0.018859	0.146125	1677171.197
DG(14:0/1	DG	DG+NH4	584.515	481.563	1.709319	0.020953	0.156861	11345187.04
PE(22:6/2(	PE	PE-H	814.5369	394.354	1.2621	0.022214	0.161931	3080687.495
PC(18:0/1{	PC	PC+HCOC	830.5899	447.3515	3.094795	0.02411	0.168009	33492706.06
SM(d14:0/	SM	SM+H	731.5893	425.3515	1.354819	0.029759	0.179202	11583487.06
DG(16:0/1	DG	DG+NH4	608.5144	457.3075	1.808351	0.031367	0.18434	13427239.04
PE(18:1/1{	PE	PE-H	742.5375	449.0925	1.64912	0.031922	0.18434	14701449.96
TG(16:1/1	TG	TG+NH4	908.7634	716.7895	1.44581	0.039403	0.202122	810102.6687
PC(15:0/2(	PC	PC+H	770.5527	383.245	3.663116	0.04081	0.204604	56791204.28
PC(18:2/1	PC	PC+HCOC	774.5268	343.0535	1.136083	0.046959	0.22427	4786801.343
DG(16:0/1	DG	DG+NH4	610.5307	490.0585	4.483379	0.049787	0.227626	102038828.3

CAP-02	CAP-03	CAP-04	CAP-05	CAP-06	CAP-301	CAP-302
16972233.95	20560732.91	21877251.17	18215465.08	24667819.06	10211591.18	11891351.24
3545549.99	6757992.312	6780664.133	5461397.069	4780198.634	3187562.412	3027758.043
4684872.195	4779317.761	5553847.933	4191660.457	4489012.665	8313477.292	8963582.829
13431039.7	13804058.44	14264181.27	11596443.93	13062957.2	6731224.584	2491541.574
1031309.316	1237884.39	1446220.931	973285.9045	1123415.138	2122739.256	2131258.512
1824567.686	1882507.175	1924033.064	1656194.125	1652149.971	4523752.312	3252548.769
2374884.155	2331414.636	2422806.918	3593414.407	4061331.289	2317318.701	2258297.991
1674302.543	1833818.3	1664287.082	1529493.55	2144763.293	1148725.197	1553803.119
1026159.184	1128258.589	1118591.373	1452444.741	1024482.257	1653269.94	1757428.545
1898966.21	2414773.24	2578866.416	1560174.558	1805364.663	1796043.499	1424308.989
147363203.1	151053598.4	145709915.5	138190193.9	123648187.8	138442116.5	105625960.3
9579581.118	10993070.14	10785226.77	8703415.025	9266848.533	6594513.835	5782312.519
2830617.558	3930836.703	4598200.99	2957629.881	3878050.068	6269075.054	5097134.713
2225710.491	1731750.344	1832795.651	2215670.767	1691231.549	3196398.772	3615492.932
6158383.01	5675341.078	5304180.362	6277200.845	3860816.896	4712181.254	4561495.659
4340048.742	4370303.151	4429114.842	1073243.679	3254655.844	5026058.821	4912946.805
2838963.228	3188028.879	3148234.476	2480701.817	2297292.255	1761554.033	1994035.639
2040794.918	2289955.765	4527391.694	1824746.1	2371351.261	2526091.908	2089522.596
3443489.115	3959306.64	3868224.769	3425494.88	3576810.63	4113607.813	4000940.422
1966743.251	2453939.491	2568996.853	2493599.418	2735816.351	2664496.722	2387870.685
22565679.82	24286422.87	22816624.4	22068235.77	22402005.59	22402667.35	23084021.2
3311883.874	4088956.632	5970099.653	5137350.03	6749.901026	14338299.9	13755970.31
2644843.733	2916020.006	3389794.503	2316071.651	3895370.252	2787119.965	2561559.829
2765131.892	2585079.646	1365436.85	2477607.921	1445483.917	921976.2369	1426294.977
11508993.49	12223181.98	11559773.9	10146592.18	9874221.129	10464620.51	8332128.314
2147751.732	795000.7563	432243.1572	4663688.533	199669.7454	1359952.359	1218923.631
2166659.173	2305452.065	3122167.297	2881450.461	3256125.638	953661.6929	720003.6307
2433657.287	2424576.844	2485725.537	2517638.095	3102039.065	2814171.151	2770400.298
3465074.53	3585672.898	4271594.324	3382798.225	4152505.74	4089421.282	3939525.177
4273862.695	4825569.829	5309058.251	3783186.994	3978746.396	6081883.208	5567210.48
6938675.345	2672322.048	1639969.047	7561294.147	728759.3721	8336543.742	4918534.429
2905843.192	3279818.224	2505562.188	2826919.553	1486287.347	3163395.653	2699057.768
14774850.81	4956485.161	2894447.206	15623209.78	1113834.334	13291425.85	10065981.3
12162947.18	13839594.55	13812756.34	12605811.91	12220185.97	13633362.66	13195023.83
9485046.328	10531928.05	10688978.24	8206190.094	9482532.605	14561425.29	11400473.84
4506268.503	6010773.507	5148683.369	4141500.001	4363805.971	7309382.116	4488317.124
2901895.575	2632124.944	2471508.073	2012989.053	2254477.56	2858429.795	3181355.87
6148369.32	6181251.871	6399190.781	5789697.327	5599568.207	5159926.117	4952922.378
4789878.383	4793223.274	4168216.422	4964584.254	2043991.115	4922822.255	5045486.273
21155416.89	24376221.66	23968736.74	18278387.96	17421194.84	26367495.22	25586700.86
36709487.97	34362526.4	31667341.9	42215458.34	25182840.48	45212129.02	37703764.62
6590205.109	1891953.045	1773917.703	7607008.81	15458594.9	679491.3552	861969.9736
4250418.455	5136065.596	4887479.348	4417200.759	3336650.224	6481086.573	6220615.989
5498869.001	6482551.704	6320857.649	5416835.084	5063649.561	6364404.169	6080189.451
648814.1563	844258.6798	840064.6238	726296.4056	595184.8455	1207520.513	930961.8551
4424919.013	4957574.267	4531655.593	4585537.62	3571855.626	4539581.951	4012199.807

7609648.859	8074118.841	5458873.282	7628495.137	2769310.821	7586850.734	8790652.211
9047789.755	9885539.061	8286112.013	9033828.661	4398824.962	9909176.023	9742363.735
10140480.68	11434420.84	10481428.37	10047111.85	6423339.266	10313552.74	10970563.52
841313.3011	760655.011	599440.851	231648.6299	438129.3354	650702.1353	789064.3161
1990409.275	1837611.692	1963781.579	1545589.702	2379062.159	2467217.889	2308373.511
11090428.74	9305604.822	8440855.809	12978806.86	8176065.465	12155182.97	10669833.74
4046375.496	4509841.284	5160075.576	3633833.614	3154928.118	7392766.676	4630364.687
50531836.34	52175015.89	49050987.89	38235743.92	39362267.72	49249186.58	46429529.32
13370399.81	13664365.47	13152411.53	16270630.18	15658776.95	13080105.62	11873972.25
13465122.1	12939810.15	10497815.84	17216703.23	9496085.699	15355453.28	23383166.13
12577643.7	15637229.06	15734273.96	12701164.26	12286828.88	15284896.34	14272101.11
494621.1142	552240.8276	495204.7184	548043.9636	587517.2873	449204.1443	433306.6659
68883901.15	87698345.05	81464026.83	62166632.42	58477807.04	66334084.5	75021834.81
6199218.261	7848868.056	7068432.682	6331738.314	5791502.059	7498691.873	8524159.98
104951732.5	104605737.5	89684041.98	117471832.6	71343669.86	119966893.8	96183164.29

CAP-303	CAP-304	CAP-305	CAP-306	CAP-601	CAP-602	CAP-603
12888897.08	11819210	10947722.15	13406215.3	813713.5663	1538245.492	888081.52
3050846.011	2682522.8	3237764.582	2516074.074	1642641.053	2107274.385	1221861.8
9167710.895	9830716.2	7670372.975	9580210.834	13550080.13	13186586.92	11466828
9613790.707	2466352.9	12743795.83	9494324.6	1982395.406	1813994.125	1397254.9
2292497.273	2611196.2	1549441.661	2109937.498	4073792.142	3238171.228	2439924
3199435.476	3243815.2	2849757.876	2882560.608	5051447.695	5150305.956	5792399.2
2287652.442	2082286.6	2551768.522	2178190.838	1432270.799	1307223.778	1222042.8
1325852.247	1141168.7	1016142.689	1094566.71	1094619.992	682487.0842	889464.27
2533209.37	1733258	1438548.264	1954583.561	2760468.861	2668409.458	2179436.8
1621749.377	1894011.9	884549.0939	1845810.988	115149.9557	70266.01172	75605.598
121624667.1	117552048	104052063.2	105998778.7	86361136.52	81124457.96	90360857
8398921.317	9464093.8	6308217.672	6442298.574	6732647.169	5939265.932	4776410
4997616.094	4682051.1	4759561.852	4668024.788	6632673.556	6394527.431	6098417.8
2849739.896	3137901.2	2593109.34	3177900.269	3431394.691	2851199.978	3908286.2
4894868.958	5125362.7	4627995.334	3905747.262	3960340.476	3130251.996	3783990.5
4742708.207	5054663.3	4778735.481	4887618.351	6329100.947	5915205.318	4812746.5
1907054.687	2221566.7	1529374.896	1834529.457	2198279.317	1770264.386	1429484
3033044.54	2319449.7	1912812.503	3054107.254	227679.6222	193830.1603	553929.41
3825247.817	4168468.8	3554154.122	4186955.034	5031906.652	6444595.887	5549819.6
2189566.597	2775482.8	2889289.86	2093104.743	89674.06771	19015.55171	185391.85
24823740.45	25768142	22040090.49	21395186.63	10249522.45	10732075.73	13591159
11428221.26	12654085	11674533.78	6906404.267	16685129.77	13498890.38	12023829
1750002.901	3411506.2	1850290.269	2193069.661	1362847.198	968033.4707	769755.75
1663109.773	1499437.3	1360143.64	1775950.577	403833.479	182039.0191	489685.12
9474133.51	8911350.1	7995946.723	8898041.086	8280130.557	6554813.584	6711929.4
1018534.927	1381823.4	1917545.444	283762.5553	160043.6545	278515.6047	38115.228
1680038.767	807332.27	637056.2437	2462158.268	922277.2302	697098.0295	556107.55
2999468.837	2981778.7	3525337.514	3535014.754	7785724.097	7179868.26	6010620.8
4141201.802	5286950.2	4296893.478	4406078.865	5912474.596	6002039.137	5416953.1
5547632.041	6663419.6	5211127.265	5667142.42	6896295.808	6053547.907	6441302
3972247.974	6345896.1	9021096.634	912028.9753	551157.0219	1092599.229	347699.39
2761880.847	2263242.9	3449274.395	2115404.868	1395194.62	2113142.559	890901.26
7797413.932	12868498	18503499.14	1562718.478	686292.1334	2024502.629	520178.21
13362121.42	13402360	13174986.68	14927599.47	17829055.45	15721060.8	15206335
12786648.2	14257992	9830329.195	10788456.57	13939698.7	12831851.79	14040993
5956064.064	3842684.3	6197060.287	3898897.964	8230437.009	6050173.501	8063057.3
3458736.18	2786582.9	2465635.55	3122532.479	4544370.35	3611039.413	4134762.4
4783763.626	4804269	4604334.039	4877863.97	5814686.315	3661917.436	4025361.7
4279421.556	3853420	6420595.055	3247423.691	2392462.473	3152355.433	1390801.4
24879176.16	25332768	23913572.81	25018908.63	32323724.72	30795400.95	24295286
42378919.95	44343425	37228175.77	31220260.71	55416512.26	37295223.46	57112465
992885.5572	740973.38	3732358.716	1953821.677	1533156.469	1701723.28	1140550.6
4382106.662	4495744	4659640.95	4860444.599	6563424.767	6046031.428	5135633.2
5606600.99	6049000.9	5479206.008	6719620.745	7743374.771	7524993.496	6856831.4
958210.619	919602.77	1170494.934	862412.2857	35403441.93	10367275.5	10002357
3485151.145	3354154.9	3903584.019	3748564.188	3728049.957	2821801.612	3080420.2

6721395.952	5433661.4	7981051.91	6195171.017	4400865.874	4711543.25	2007231.9
10102683.05	8143712.4	11432978.12	7228583.37	5077840.595	7477855.09	2788670.2
11258322.44	9580406.6	13945624.72	9112318.794	6670232.782	9888988.154	3655395.3
700321.5394	660229.96	670942.6921	915193.497	5731865.38	4727800.039	3275742.1
2669666.976	3034806.8	2522383.239	2392553.016	25965290.62	4801179.965	4904833.2
10889079.12	11144554	12675848.11	9753292.516	13688284.28	11573794.47	16060106
6683717.743	4412792.8	6125943.505	4859391.14	5658777.933	5168959.639	6425482.9
47853419.93	48976831	38362647.5	51344124.99	73319133.25	54332335.7	52464058
12207176.37	11558238	13009512.17	12288630.22	20499756.32	9248714.47	9934397.4
14174424.58	13552713	27506551.34	20473485.77	16259392.21	13212645.86	20640599
15434136.82	16983342	12107295.63	15717449.53	21505320.09	17877549.58	15827820
538635.3088	442109.55	538406.8653	643274.1692	7798877.451	6452104.889	4752787.9
65174940.28	72698466	57865719.58	70106599.16	73766152.46	55695525.1	43884931
5567505.115	5418963.7	6907936.682	6677491.375	8925950.617	6759033.665	7849931.2
117275534.8	115418995	108132334.5	92506899.25	125297719.5	104864316.2	141518014

CAP-604	CAP-605	CAP-606
840853.9496	1029873.724	1666130.913
1352038.199	1535281.982	1919484.614
10797370.29	9892265.192	11520645.32
1281036.88	1458064.251	1186011.767
2960425.052	2382212.099	2622334.834
4766354.087	4421528.479	4195853.894
1059593.832	1141203.317	820518.0575
701686.3344	831819.7418	738222.9484
2054432.368	2000302.35	2583042.23
263568.0738	641726.8457	402698.2015
80057388.95	76391106.52	89824077.68
5127762.73	4642954.987	5577961.795
5253071.922	5452500.166	4640616.463
3323630.663	3519367.084	3091957.718
3379425.335	3017479.81	2844077.421
4472503.878	4926386.295	5418493.319
1558650.953	1541425.672	1840829.518
1543365.741	1423138.988	1823588.64
4206095.93	3972136.869	4633285.319
800272.9354	1565560.338	1451904.208
21039630.03	19119113.3	20765908.33
14120990.05	11963863.92	13108508.3
1619606.52	1780692.584	1938753.33
1317425.883	1334176.678	1344224.647
6578273.648	6102700.218	6749192.365
181672.094	126464.8183	191468.7448
728970.4278	644604.7077	666747.2202
3280497.937	3399824.973	3092130.811
4516146.997	4469180.973	4235643.875
5789701.633	5004835.328	5461989.417
619987.4659	493605.6994	711130.9761
1143313.467	1472803.861	1612067.408
831826.1941	663045.67	853882.7101
15352963.61	13647698.69	14418230.26
12499558.85	11047042.15	11995286.65
11521755.82	6700031.411	9150387.723
3258897.668	3117252.528	2843167.414
3689117.379	3605062.176	3781159.379
2032746.617	2270313.173	2942312.055
24020496.93	24397778.88	27737364.09
47200034.2	49317340.59	45249758.51
799763.4123	1037984.573	540813.1969
10316743.84	8616802.387	6091047.068
6930723.433	5873621.023	7202575.998
1153314.719	700088.1613	951081.9544
2719898.388	2619997.788	2858041.881

2938646.342	3574073.885	4995801.3
3750966.962	4863414.63	5500954.035
4932934.38	6312006.81	6523006.679
826408.9699	704587.1622	883101.5339
2033600.205	2280223.657	1891844.391
12344590.57	16029594.61	12256212.43
7656025.662	3707538.747	7531555.092
47312331.56	52848781.07	57604358.94
8202713.603	7636684.056	8526273.411
14797628.07	18573456.7	27500795.6
16980934.91	14902489.89	16955349
558112.4029	456268.8338	1300621.731
46239894.41	38135181.05	48326517.73
9381770.03	6993995.014	8670170.231
115519063.1	138406271.6	115481595.6
