# Supplementary material for: Replacement of Dietary Fishmeal with Clostridium autoethanogenum Protein on Lipidomics and Lipid Metabolism in Muscle of Pearl Gentian Grouper
Source: Aquac Nutr. 2023 Jun 30;2023:6723677. doi: 10.1155/2023/6723677 (PMC10328730; doi:10.1155/2023/6723677)
Supplement: Supplementary 9 — Receiver operating characteristic curve of fatty acids in CAP-0 and CAP-30 groups. [file 6723677.f9.pdf]

Table S8 Receiver operating characteristic curve of fatty acids in CAP-0 and CAP-3

Fatty acids	AUC	Ci1	Ci2	specificity	sensitivity
C18:1N9T	0.833	0.478	1	1	0.667
C20:5N3	0.819	0.451	1	0.833	0.833
C18:1N12	0.778	0.513	1	1	0.667
C22:1N9	0.778	0.346	1	0.667	0.833
C20:3N6	0.75	0.423	0.966	0.667	0.833
C8:0	0.722	0.489	0.966	0.667	0.833
C18:2N6T	0.722	0.423	0.91	0.833	0.667
C18:3N6	0.722	0.358	0.994	0.667	0.667
C20:1T	0.722	0.401	0.966	0.667	0.667
C18:3N3	0.722	0.417	1	0.667	0.833
C21:0	0.722	0.417	0.987	0.667	0.833
C20:3N3	0.708	0.417	1	0.667	0.833
C14:0	0.694	0.333	0.938	0.667	0.667
C16:1T	0.694	0.346	0.944	0.833	0.667
C16:1	0.694	0.417	0.944	0.667	0.833
C18:1N9C	0.694	0.312	0.944	0.667	0.833
C18:1N7	0.681	0.333	0.917	0.667	0.833
C20:1	0.681	0.29	0.917	0.667	0.833
C12:0	0.667	0.444	0.987	0.667	0.667
C19:1N9T	0.667	0.198	0.876	1	0.5
C18:2N6	0.667	0.417	0.972	0.667	0.833
C22:0	0.667	0.444	1	0.667	0.833
C20:4N6	0.667	0.179	1	0.667	0.833
C23:0	0.667	0.352	0.987	0.667	0.833
C22:4	0.667	0.29	0.966	1	0.5
C22:5N6	0.667	0.333	1	1	0.667
C22:6N3	0.667	0.352	0.938	0.833	0.667
C22:5N3	0.653	0.333	0.91	0.667	0.833
C14:1T	0.639	0.185	0.926	0.5	0.833
C15:1T	0.639	0.346	0.91	0.667	0.667
C6:0	0.625	0.241	0.966	0.667	0.667
C20:0	0.625	0.361	0.855	0.667	0.667
C20:2	0.625	0.25	0.91	0.5	0.833
C13:0	0.611	0.367	0.944	0.833	0.5
C15:1	0.611	0.185	0.876	1	0.5
C18:1N7T	0.611	0.29	0.833	0.833	0.667
C24:1	0.611	0.333	0.91	0.667	0.667
C11:0	0.583	0.256	0.883	0.833	0.667
C17:1T	0.583	0.284	0.919	0.833	0.5
C22:1N9T	0.583	0.284	0.966	0.667	0.667
C24:0	0.583	0.173	0.855	0.5	0.667
C15:0	0.556	0.278	0.883	0.5	0.667
C17:1	0.556	0.207	0.938	0.5	0.667
C10:0	0.542	0.179	0.772	0.833	0.5
C18:0	0.514	0.151	0.833	0.833	0.5
C22:2	0.5	0.179	0.833	0.667	0.5
C18:1N12T	0.486	0.102	0.833	0.5	0.667
C16:0	0.472	0.269	0.855	0.5	0.667
C14:1	0.465	0.25	0.966	0.667	0.667
C17:0	0.444	0.167	0.787	0.5	0.667

C19:1N12T	0.444	0.124	0.833	0.5	0.667

30 groups

threshold
2.11
73.7395
29.268
11.184
2.4305
0.1545
0.1345
0.52
4.2495
18.1745
0.714
8.298
15.1145
3.012
18.206
105.1405
25.9335
12.6675
0.541
1.869
119.4535
1.5105
13.752
0.475
0.917
10.1635
145.284
28.734
2.016
3.106
0.829
4.705
7.8355
0.5045
2.9775
21.8815
5.0895
0.0805
5.089
2.3695
1.543
6.163
4.1275
0.125
234.6925
1.2145
1.197
397.0905
11.338
11.54

2.3775
