# Supplementary material for: Replacement of Dietary Fishmeal with Clostridium autoethanogenum Protein on Lipidomics and Lipid Metabolism in Muscle of Pearl Gentian Grouper
Source: Aquac Nutr. 2023 Jun 30;2023:6723677. doi: 10.1155/2023/6723677 (PMC10328730; doi:10.1155/2023/6723677)
Supplement: Supplementary 10 — Receiver operating characteristic curve of fatty acids in CAP-30 and CAP-60 groups. [file 6723677.f10.pdf]

Table S9 Receiver operating characteristic curve of fatty acids in CAP-30 and CAP-

Fatty acids	AUC	Ci1	Ci2	specificity	sensitivity
C18:1N9T	0.944	0.778	1	0.833	0.833
C20:5N3	0.944	0.778	1	0.833	1
C18:1N12	0.889	0.583	1	0.667	1
C22:1N9	0.778	0.426	0.972	0.667	0.833
C20:3N6	0.778	0.506	0.966	0.667	0.833
C8:0	0.75	0.513	0.978	1	0.667
C18:2N6T	0.75	0.429	0.966	0.667	0.667
C18:3N6	0.736	0.451	0.889	0.667	0.833
C20:1T	0.736	0.383	0.889	0.667	0.667
C18:3N3	0.736	0.333	1	1	0.667
C21:0	0.722	0.346	0.91	1	0.5
C20:3N3	0.694	0.333	0.917	0.5	0.833
C14:0	0.681	0.367	0.944	0.667	0.667
C16:1T	0.667	0.444	0.91	0.667	0.833
C16:1	0.667	0.312	0.889	0.5	0.833
C18:1N9C	0.667	0.389	0.91	0.5	1
C18:1N7	0.667	0.346	0.972	0.667	0.667
C20:1	0.667	0.263	0.889	0.833	0.5
C12:0	0.667	0.346	0.861	0.5	0.833
C19:1N9T	0.667	0.201	0.944	0.667	0.667
C18:2N6	0.667	0.346	0.883	0.667	0.833
C22:0	0.653	0.34	0.889	0.5	0.833
C20:4N6	0.639	0.312	0.91	0.667	0.667
C23:0	0.639	0.389	0.987	0.5	0.833
C22:4	0.639	0.303	0.947	0.5	0.667
C22:5N6	0.611	0.312	0.917	0.667	0.833
C22:6N3	0.611	0.312	0.855	0.5	0.833
C22:5N3	0.611	0.312	0.926	0.667	0.667
C14:1T	0.611	0.374	0.944	0.5	0.833
C15:1T	0.611	0.269	0.917	0.5	0.833
C6:0	0.611	0.241	0.87	0.5	0.667
C20:0	0.597	0.34	0.904	0.5	0.667
C20:2	0.583	0.306	0.919	0.5	1
C13:0	0.569	0.228	0.861	1	0.5
C15:1	0.569	0.185	0.932	0.5	0.833
C18:1N7T	0.556	0.25	0.827	0.5	0.833
C24:1	0.556	0.25	0.91	0.5	0.833
C11:0	0.556	0.222	0.833	0.5	0.833
C17:1T	0.556	0.306	0.883	0.667	0.667
C22:1N9T	0.556	0.192	0.919	0.5	0.833
C24:0	0.556	0.194	0.91	0.5	0.833
C15:0	0.556	0.222	0.778	0.5	0.833
C17:1	0.556	0.167	0.855	0.833	0.5
C10:0	0.542	0.309	0.855	0.667	0.667
C18:0	0.528	0.228	0.833	0.5	0.667
C22:2	0.5	0.228	0.833	0.5	0.667
C18:1N12T	0.5	0.179	0.932	0.833	0.5
C16:0	0.5	0.228	0.876	0.5	0.833
C14:1	0.5	0.222	0.842	0.5	0.833
C17:0	0.431	0.145	0.737	0.5	0.667

C19:1N12T	0.361	0.111	0.71	0.667	0.5

60 groups

threshold
3.5905
1.9585
0.7405
1.493
1.1025
0.0795
35.205
0.137
5.577
3.0315
4.5465
0.462
16.5985
0.0805
31.2745
4.5365
23.4305
2.489
1.141
14.73
137.2125
2.4875
140.76
43.3225
7.3755
4.953
1.44
26.3765
2.7795
0.7945
1.2425
0.8595
3.69
6.7005
8.6955
7.319
32.8345
13.4675
2.743
206.8755
21.7895
1.9845
13.5165
0.111
6.8425
439.801
37.201
12.729
219.0315
261.9925

1.2285
